# Supplementary material for: Distributions and Trends of the Global Burden of Colorectal Cancer Attributable to Dietary Risk Factors over the Past 30 Years
Source: Nutrients. 2023 Dec 30;16(1):132. doi: 10.3390/nu16010132 (PMC10780867; doi:10.3390/nu16010132)
Supplement: Supplementary file 1 [file nutrients-16-00132-s001.zip › nutrients-2659614-supplementary.pdf]

Supplementary material

Distributions and trends of the global burden of colorectal cancer attributable to dietary risk factors over the past 30 years

Supplementary Figure S1. The global burden of CRC attributable to diet low in whole grains over the past 30 years.2
Supplementary Figure S2. The global burden of CRC attributable to diet low in milk over the past 30 years. 3
Supplementary Figure S3. The global burden of CRC attributable to diet low in fiber over the past 30 years. 4
Supplementary Figure S4. The global burden of CRC attributable to diet low in calcium over the past 30 years. 5
Supplementary Figure S5. The global burden of CRC attributable to diet high in red meat over the past 30 years. .. 6
Supplementary Figure S6. The global burden of CRC attributable to diet high in processed meat over the past 30 years. 7
Supplementary Figure S7. Flow chart of data selection in this study. 8
Supplementary Table S1. The definition and theoretical minimum risk exposure level for dietary risk factor in 2019. 9
Supplementary Table S2. The global death cases and age-standardized death rates of CRC in 204 countries and territories, and their temporal trends from 1990 to 2019. 10
Supplementary Table S3. The death cases and age-standardized death rates of CRC attributable to all dietary risks in 1990 and 2019, and their temporal trends from 1990 to 2019. 21
Supplementary Table S4. The death cases and age-standardized death rates of CRC attributable to all dietary risk factors in 204 countries and territories, and their temporal trends from 1990 to 2019. 23
Supplementary Table S5. The death cases and age-standardized death rates of CRC attributable to diet low in whole grains in 1990 and 2019, and their temporal trends from 1990 to 2019. 105
Supplementary Table S6. The death cases and age-standardized death rates of CRC attributable to diet low in milk in 1990 and 2019, and their temporal trends from 1990 to 2019. 107
Supplementary Table S7. The death cases and age-standardized death rates of CRC attributable to diet low in fiber in 1990 and 2019, and their temporal trends from 1990 to 2019. 109
Supplementary Table S8. The death cases and age-standardized death rates of CRC attributable to diet low in calcium in 1990 and 2019, and their temporal trends from 1990 to 2019. 111
Supplementary Table S9. The death cases and age-standardized death rates of CRC attributable to diet high in red meat in 1990 and 2019, and their temporal trends from 1990 to 2019. 113
Supplementary Table S10. The death cases and age-standardized death rates of CRC attributable to diet high in processed meat in 1990 and 2019, and their temporal trends from 1990 to 2019. 115

Supplementary Figure S1. The global burden of CRC attributable to diet low in whole grains over the past 30 years.

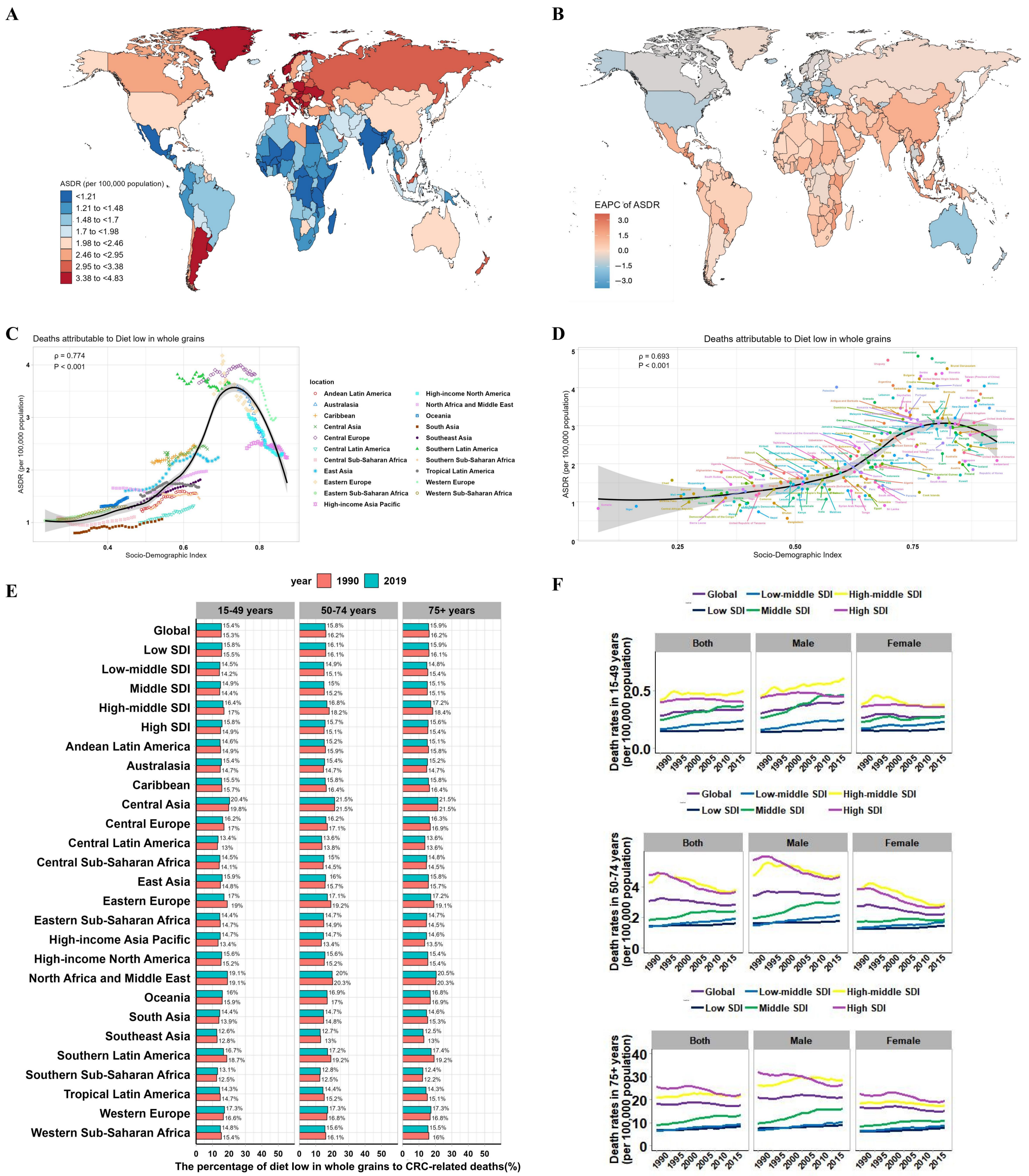

(A) The ASDR in 204 countries and territories in 2019. (B) The EAPC in ASDR in 204 countries and territories from 1990 to 2019. (C) Relationship between SDI and ASDR of CRC attributable to diet low in whole grains by regions in 2019. (D) Relationship between SDI and ASDR of CRC attributable to diet low in whole grains by nations in 2019. (E) The percentage of diet low in whole grains to CRC-related deaths between 1990 and 2019, in territories with low to high SDIs and in 21 GBD regions. (F) Deaths attributable to diet low in whole grains in CRC by different age and sex from 1990 to 2019.

Supplementary Figure S2. The global burden of CRC attributable to diet low in milk over the past 30 years.

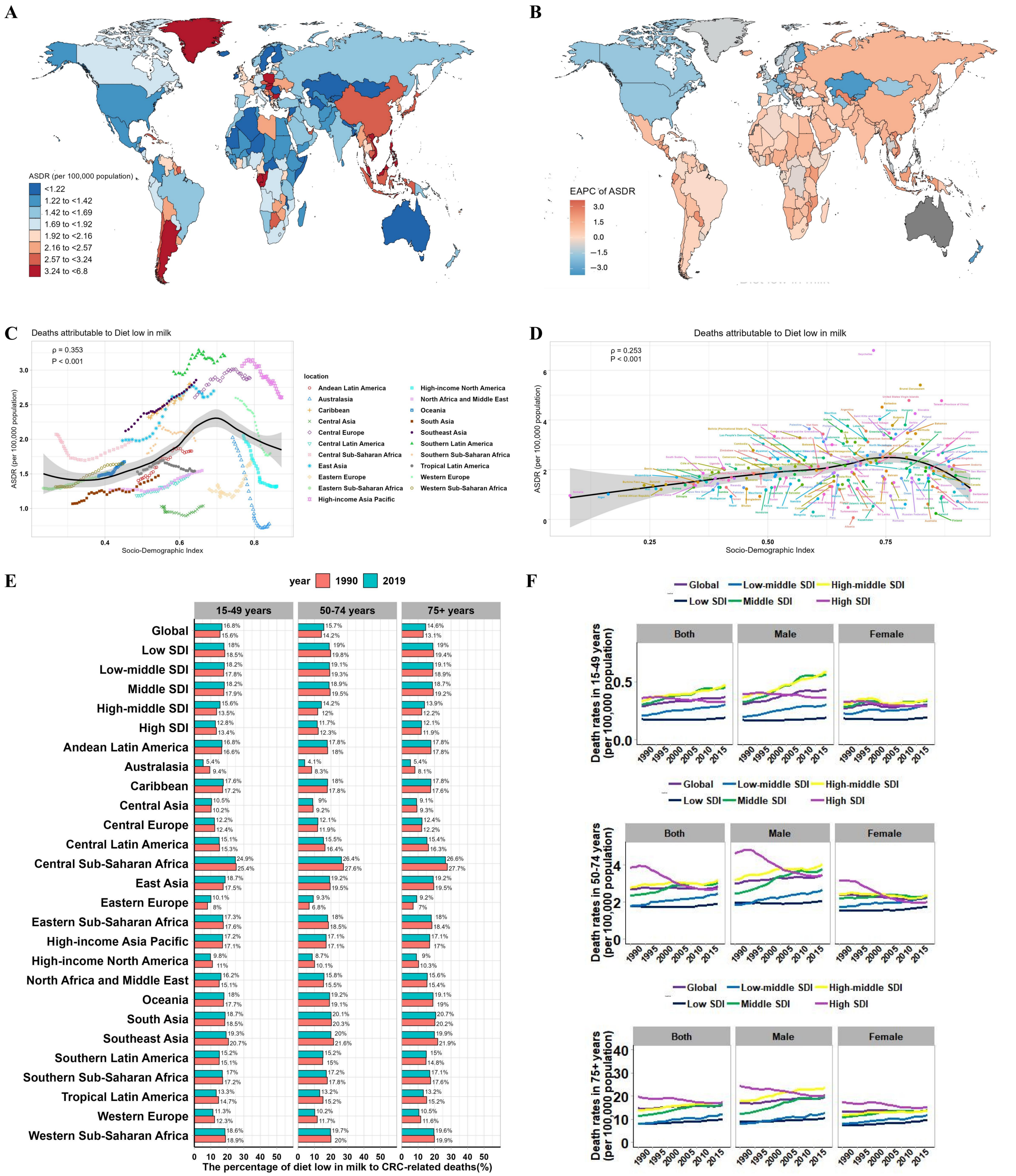

(A) The ASDR in 204 countries and territories in 2019. (B) The EAPC in ASDR in 204 countries and territories from 1990 to 2019. (C) Relationship between SDI and ASDR of CRC attributable to diet low in milk by regions in 2019. (D) Relationship between SDI and ASDR of CRC attributable to diet low in milk by nations in 2019. (E) The percentage of diet low in milk to CRC-related deaths between 1990 and 2019, in territories with low to high SDIs and in 21 GBD regions. (F) Deaths attributable to diet low in milk in CRC by different age and sex from 1990 to 2019.

Supplementary Figure S3. The global burden of CRC attributable to diet low in fiber over the past 30 years.

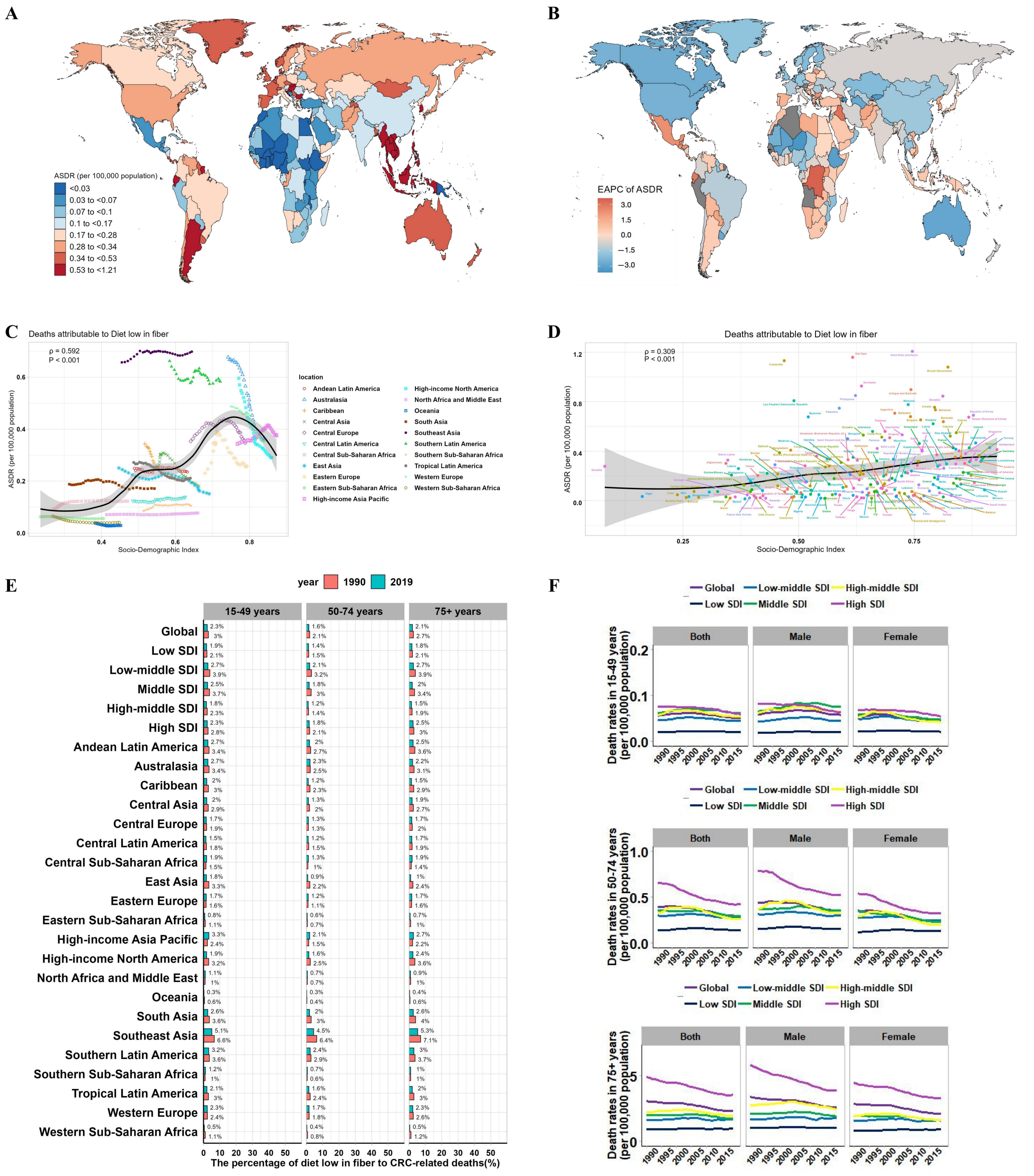

(A) The ASDR in 204 countries and territories in 2019. (B) The EAPC in ASDR in 204 countries and territories from 1990 to 2019. (C) Relationship between SDI and ASDR of CRC attributable to diet low in fiber by regions in 2019. (D) Relationship between SDI and ASDR of CRC attributable to diet low in fiber by nations in 2019. (E) The percentage of diet low in fiber to CRC-related deaths between 1990 and 2019, in territories with low to high SDIs and in 21 GBD regions. (F) Deaths attributable to diet low in fiber in CRC by different age and sex from 1990 to 2019.

Supplementary Figure S4. The global burden of CRC attributable to diet low in calcium over the past 30 years.

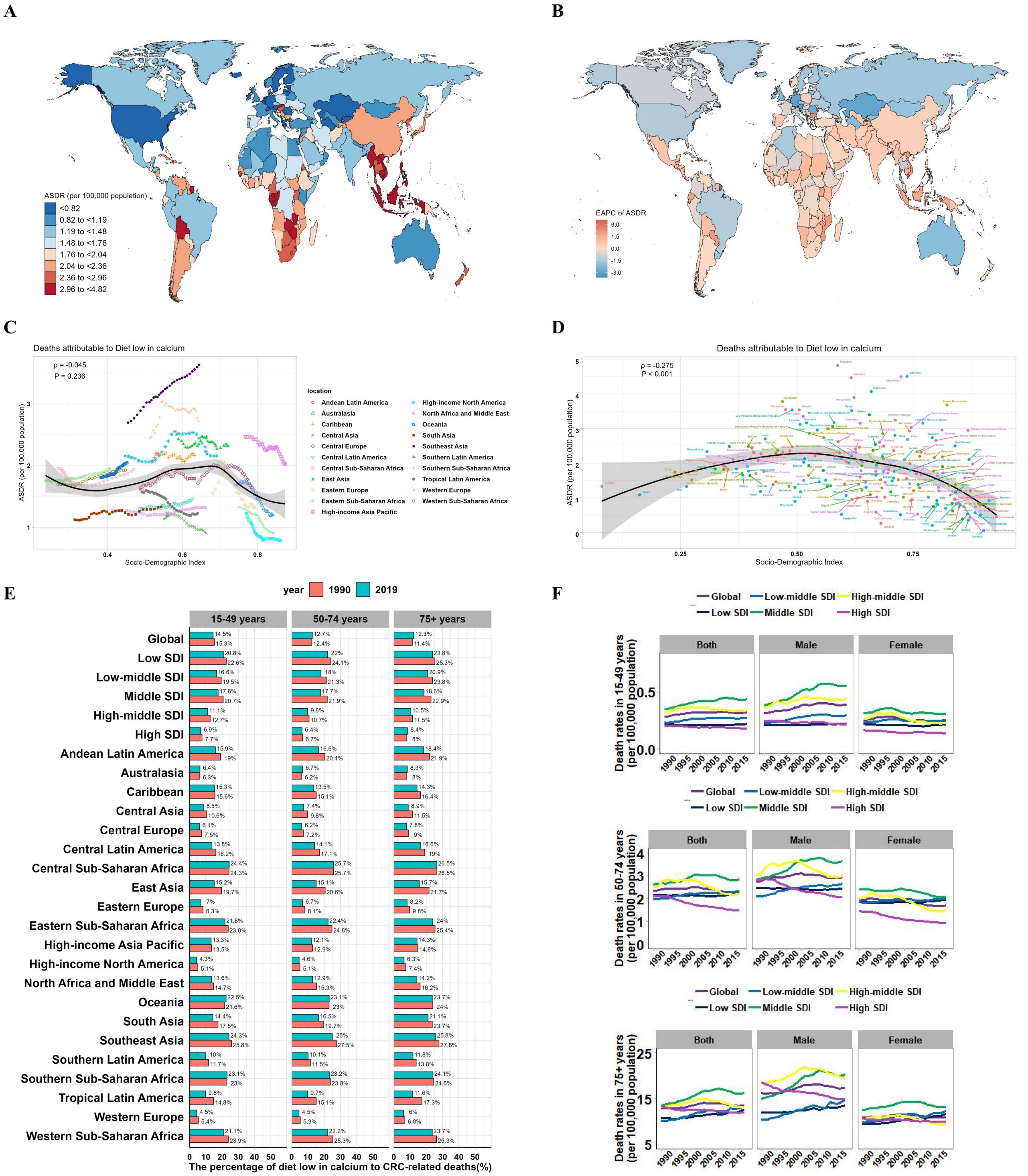

(A) The ASDR in 204 countries and territories in 2019. (B) The EAPC in ASDR in 204 countries and territories from 1990 to 2019. (C) Relationship between SDI and ASDR of CRC attributable to diet low in calcium by regions in 2019. (D) Relationship between SDI and ASDR of CRC attributable to diet low in calcium by nations in 2019. (E) The percentage of diet low in calcium to CRC-related deaths between 1990 and 2019, in territories with low to high SDIs and in 21 GBD regions. (F) Deaths attributable to diet low in calcium in CRC by different age and sex from 1990 to 2019.

Supplementary Figure S5. The global burden of CRC attributable to diet high in red meat over the past 30 years.

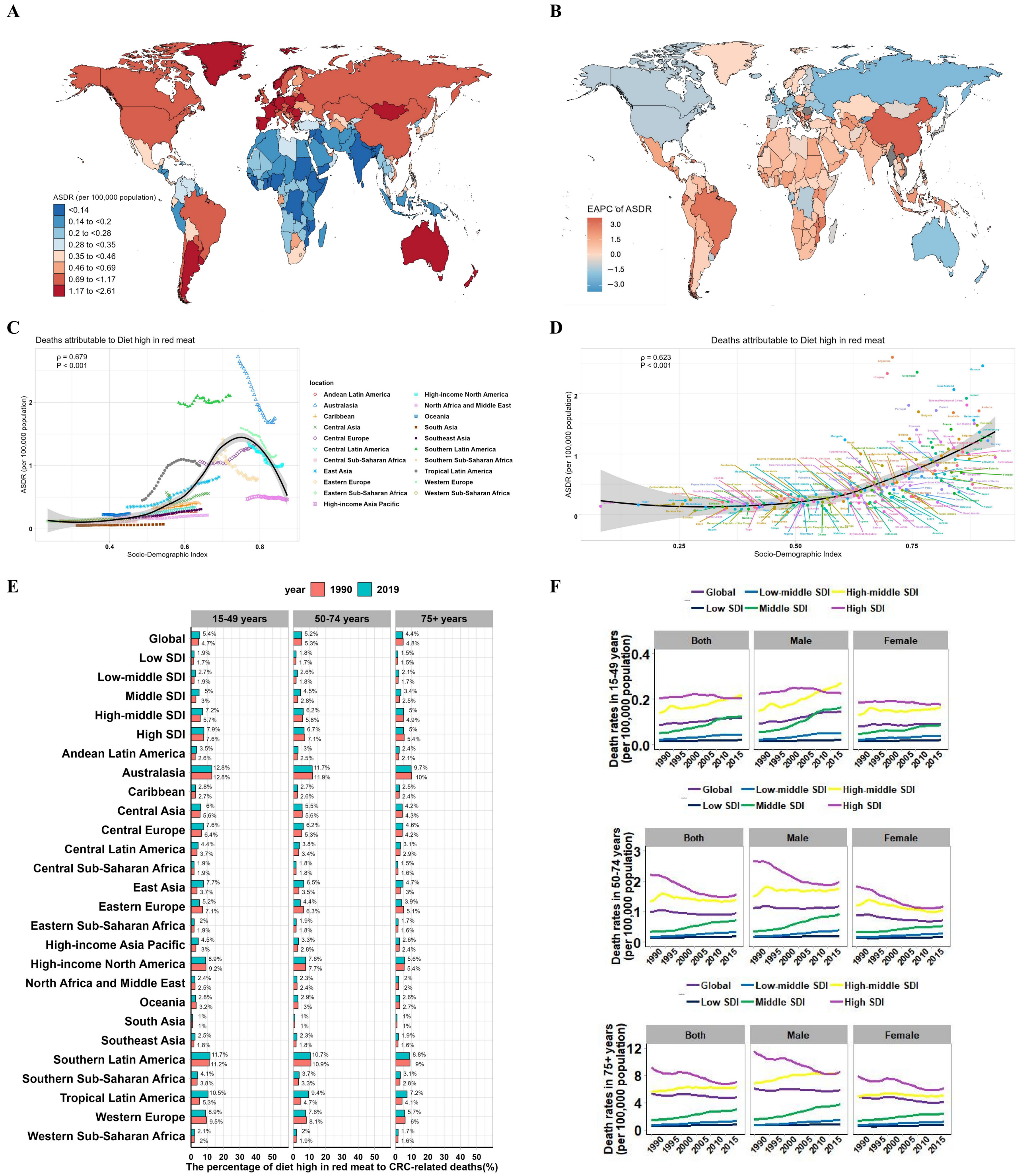

(A) The ASDR in 204 countries and territories in 2019. (B) The EAPC in ASDR in 204 countries and territories from 1990 to 2019. (C) Relationship between SDI and ASDR of CRC attributable to diet high in red meat by regions in 2019. (D) Relationship between SDI and ASDR of CRC attributable to diet high in red meat by nations in 2019. (E) The percentage of diet high in red meat to CRC-related deaths between 1990 and 2019, in territories with low to high SDIs and in 21 GBD regions. (F) Deaths attributable to diet high in red meat in CRC by different age and sex from 1990 to 2019.

Supplementary Figure S6. The global burden of CRC attributable to diet high in processed meat over the past 30 years.

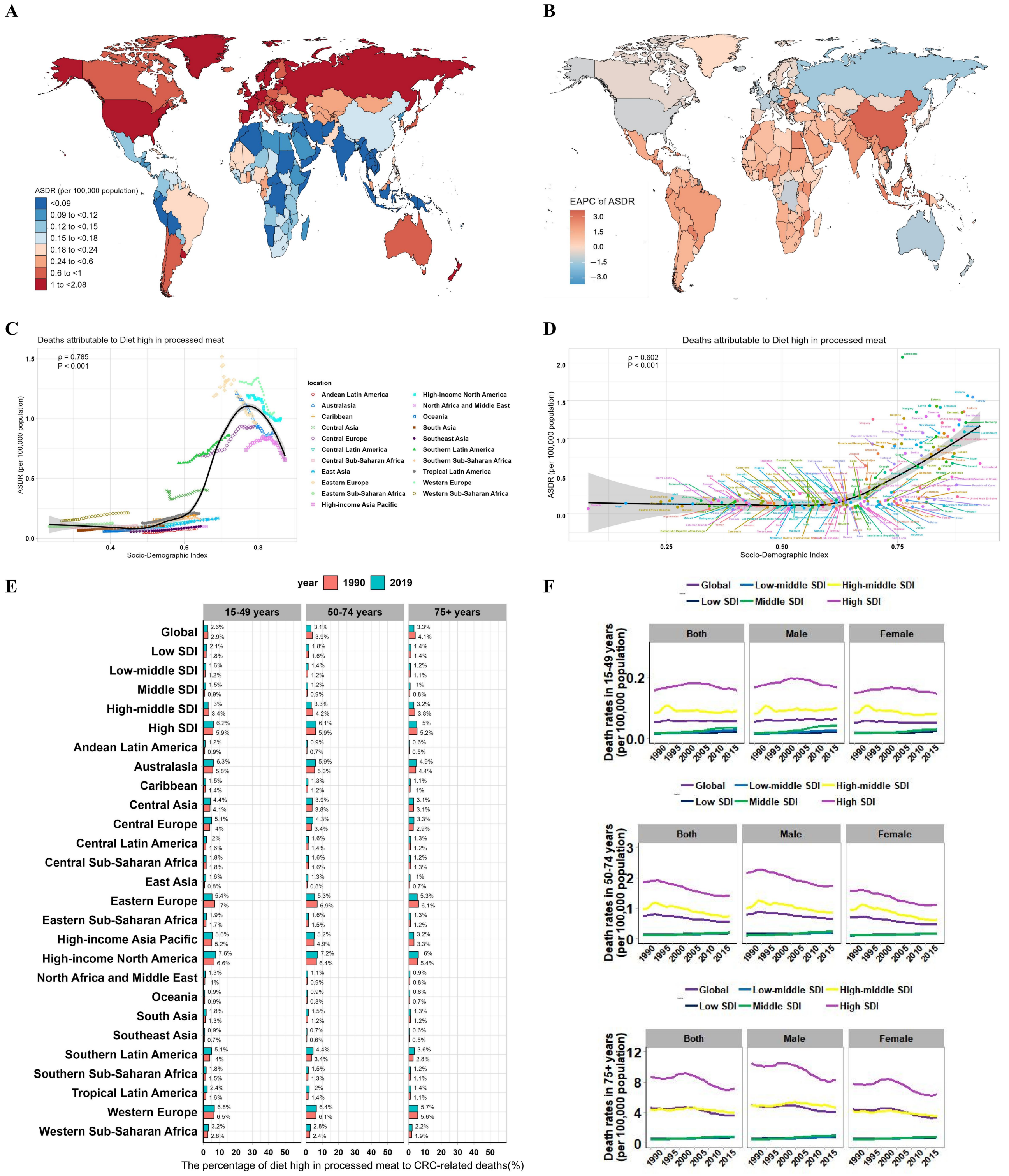

(A) The ASDR in 204 countries and territories in 2019. (B) The EAPC in ASDR in 204 countries and territories from 1990 to 2019. (C) Relationship between SDI and ASDR of CRC attributable to diet low in whole grains by regions in 2019. (D) Relationship between SDI and ASDR of CRC attributable to diet low in whole grains by nations in 2019. (E) The percentage of diet low in whole grains to CRC-related deaths between 1990 and 2019, in

territories with low to high SDIs and in 21 GBD regions. (F) Deaths attributable to diet low in whole grains in CRC by different age and sex from 1990 to 2019.

Supplementary Figure S7. Flow chart of data selection in this study.

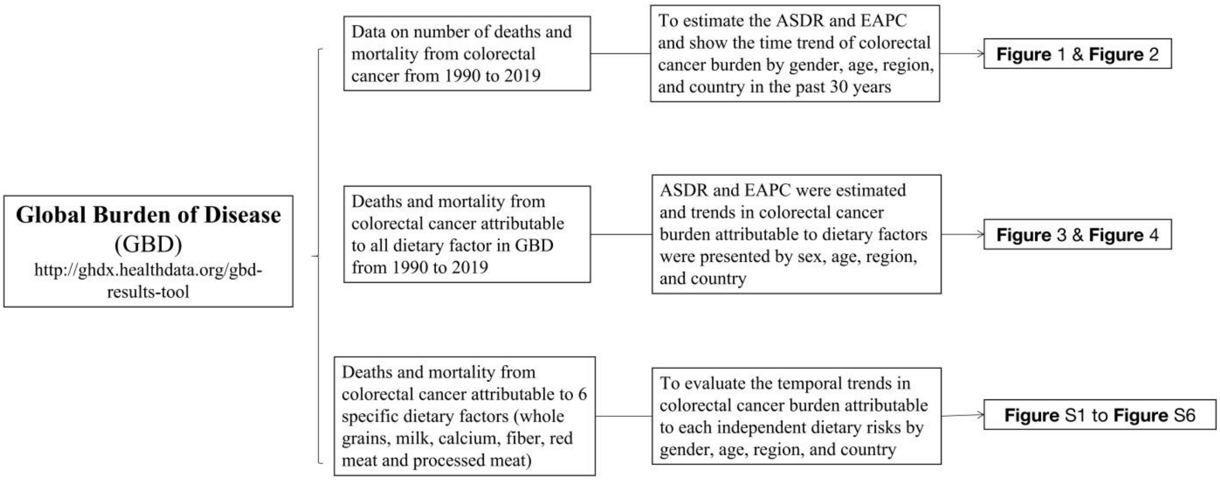

Supplementary Table S1. The definition and theoretical minimum risk exposure level for dietary risk factor in 2019.

Dietary risks	Definition	Theoretical minimum risk exposure level
Diet low in whole grains	Average daily consumption of whole grains (bran, germ, and endosperm in their natural proportion) from breakfast cereals, bread, rice, pasta, biscuits, muffins, tortillas, pancakes, and other sources	Consumption of whole grains 100-150 g per day
Diet low in milk	Average daily consumption of milk, including non-fat, low-fat, and full-fat milk, excluding soy milk and other plant derivatives	Consumption of milk 350-520 g per day
Diet low in fiber	Average daily intake of fibre from all sources including fruits, vegetables, grains, legumes, and pulses	Consumption of fibre 19-28 g per day
Diet low in calcium	Average daily intake of calcium from all sources, including milk, yogurt, and cheese	Consumption of calcium 1.0-1.5 g per day
Diet high in red meat	Average daily consumption of red meat (beef, pork, lamb, and goat but excluding poultry, fish, eggs, and all processed meats)	Consumption of red meat 18-27 g per day
Diet high in processed meat	Average daily consumption of meat preserved by smoking, curing, salting, or addition of chemical preservatives	Consumption of processed meat 0-4 g per day

Supplementary Table S2. The global death cases and age-standardized death rates of CRC in 204 countries and territories, and their temporal trends from 1990 to 2019.

Countries	1990		2019		1990-2019
	Number of deaths (95% UI)	ASDR per 100,000 (95% UI)	Number of deaths (95% UI)	ASDR per 100,000 (95% UI)	EAPC of ASDR (95% CI)
Afghanistan	539 (324 to 818)	7.87 (5 to 11.76)	1046 (686 to 1420)	8.43 (6.01 to 11.18)	0.45 (0.24 to 0.66)
Albania	170 (157 to 182)	8.63 (7.94 to 9.26)	385 (291 to 502)	9.15 (6.96 to 11.89)	0.59 (0.33 to 0.86)
Algeria	744 (604 to 901)	7.24 (6 to 8.56)	2384 (1889 to 2951)	8.05 (6.4 to 9.77)	0.44 (0.4 to 0.49)
American Samoa	3 (3 to 4)	16.3 (13.31 to 19.03)	7 (6 to 9)	16.55 (14.07 to 19.59)	0.14 (0.02 to 0.27)
Andorra	14 (11 to 19)	28.75 (22.82 to 37.96)	34 (26 to 42)	22.97 (17.88 to 28.86)	-0.82 (-0.87 to -0.77)
Angola	285 (201 to 412)	8.01 (5.61 to 11.18)	952 (738 to 1212)	9.65 (7.82 to 11.99)	0.74 (0.57 to 0.91)
Antigua and Barbuda	7 (6 to 7)	11.99 (10.88 to 13.04)	15 (13 to 17)	16.16 (14.01 to 18.45)	0.94 (0.79 to 1.08)
Argentina	6593 (6296 to 6834)	21.15 (20.15 to 21.96)	12886 (12008 to 13759)	23.59 (22 to 25.13)	0.26 (0.12 to 0.41)
Armenia	372 (354 to 391)	13.97 (13.3 to 14.72)	585 (492 to 681)	14.3 (12.07 to 16.63)	0.08 (-0.04 to 0.2)
Australia	4497 (4289 to 4642)	23.38 (22.22 to 24.16)	6734 (6065 to 7265)	15.49 (14.08 to 16.66)	-1.79 (-1.95 to -1.63)
Austria	3276 (3117 to 3388)	27.16 (25.84 to 28.08)	2603 (2355 to 2811)	13.54 (12.36 to 14.54)	-2.81 (-2.95 to -2.68)
Azerbaijan	541 (500 to 581)	10.55 (9.75 to 11.34)	1063 (875 to 1304)	12.2 (9.77 to 15.16)	1.09 (0.6 to 1.58)
Bahamas	25 (23 to 28)	17.23 (15.84 to 18.71)	68 (55 to 82)	18.41 (15.08 to 22.19)	0.49 (0.38 to 0.59)
Bahrain	19 (16 to 22)	12.44 (10.41 to 14.49)	84 (64 to 106)	11.42 (9.08 to 14.03)	-0.44 (-0.73 to -0.16)
Bangladesh	2047 (1502 to 2725)	4.62 (3.36 to 6.25)	6023 (4166 to 8611)	4.94 (3.4 to 7.07)	0.14 (0 to 0.29)
Barbados	52 (48 to 56)	17.46 (16.16 to 18.58)	118 (99 to 139)	23.96 (20.09 to 28.31)	1.08 (0.87 to 1.28)
Belarus	2246 (2157 to 2335)	17.47 (16.78 to 18.15)	2734 (2230 to 3445)	17.03 (13.86 to 21.48)	-0.85 (-1.14 to -0.56)
Belgium	3998 (3747 to 4183)	25.61 (23.99 to 26.79)	4325 (3880 to 4687)	17.1 (15.59 to 18.44)	-1.54 (-1.65 to -1.43)
Belize	6 (6 to 7)	7.02 (6.37 to 7.68)	28 (24 to 32)	10.42 (9.02 to 11.99)	1.23 (0.78 to 1.68)

Countries	1990		2019		1990-2019
	Number of deaths (95% UI)	ASDR per 100,000 (95% UI)	Number of deaths (95% UI)	ASDR per 100,000 (95% UI)	EAPC of ASDR (95% CI)
Benin	122 (103 to 143)	6.47 (5.5 to 7.59)	331 (261 to 423)	7.63 (6.19 to 9.53)	0.68 (0.63 to 0.72)
Bermuda	16 (15 to 17)	27.25 (24.93 to 29.34)	26 (21 to 31)	18.81 (15.73 to 22.69)	-1.26 (-1.37 to -1.15)
Bhutan	10 (6 to 15)	4.61 (2.64 to 6.5)	36 (22 to 49)	6.97 (4.39 to 9.42)	1.44 (1.42 to 1.46)
Bolivia (Plurinational State of)	261 (187 to 326)	8.85 (6.49 to 10.99)	992 (678 to 1314)	12.26 (8.43 to 16.2)	1.07 (1.03 to 1.11)
Bosnia and Herzegovina	544 (515 to 576)	14.4 (13.54 to 15.31)	1319 (1044 to 1639)	22.32 (17.81 to 27.66)	1.88 (1.6 to 2.17)
Botswana	54 (41 to 70)	10.69 (8.29 to 13.76)	189 (133 to 252)	15.75 (11.59 to 20.48)	1.06 (0.77 to 1.35)
Brazil	8340 (7992 to 8610)	10.21 (9.68 to 10.57)	27052 (25085 to 28403)	11.67 (10.81 to 12.28)	0.55 (0.4 to 0.71)
Brunei Darussalam	25 (21 to 30)	30.25 (25.59 to 35.26)	72 (64 to 82)	30.26 (26.58 to 34.1)	0.67 (0.4 to 0.94)
Bulgaria	2366 (2247 to 2484)	19.4 (18.4 to 20.31)	3672 (2991 to 4477)	25.11 (20.41 to 30.62)	2.15 (1.67 to 2.64)
Burkina Faso	228 (182 to 286)	5.87 (4.75 to 7.37)	585 (461 to 742)	7.27 (5.82 to 9.12)	0.77 (0.63 to 0.92)
Burundi	180 (143 to 231)	8.02 (6.44 to 10.12)	303 (216 to 438)	7.17 (5.21 to 10.11)	-0.54 (-0.63 to -0.45)
Cabo Verde	11 (9 to 12)	4.49 (4.04 to 4.97)	47 (38 to 56)	11.35 (8.97 to 13.38)	2.64 (2.29 to 2.98)
Cambodia	390 (303 to 506)	9.03 (7.24 to 11.72)	1561 (1240 to 1881)	14.02 (11.24 to 16.54)	1.58 (1.53 to 1.64)
Cameroon	364 (288 to 445)	9.19 (7.28 to 11.17)	1114 (838 to 1460)	10.64 (8.25 to 13.84)	0.58 (0.53 to 0.62)
Canada	6315 (5953 to 6544)	19.6 (18.42 to 20.3)	11616 (10433 to 12532)	16.22 (14.74 to 17.44)	-0.67 (-0.72 to -0.61)
Central African Republic	81 (60 to 104)	7.43 (5.78 to 9.42)	128 (92 to 178)	6.37 (4.71 to 8.79)	-0.44 (-0.5 to -0.38)
Chad	142 (110 to 177)	5.33 (4.13 to 6.66)	369 (285 to 480)	7.37 (5.77 to 9.35)	1.27 (1.2 to 1.34)
Chile	1167 (1105 to 1215)	12.69 (11.97 to 13.22)	3524 (3224 to 3795)	14.71 (13.46 to 15.85)	0.74 (0.65 to 0.83)
China	79322 (69661 to 89295)	10.18 (9.03 to 11.37)	261777 (224404 to 303318)	13.86 (11.92 to 16.01)	1.39 (1.14 to 1.63)

Countries	1990		2019		1990-2019
	Number of deaths (95% UI)	ASDR per 100,000 (95% UI)	Number of deaths (95% UI)	ASDR per 100,000 (95% UI)	EAPC of ASDR (95% CI)
Colombia	1544 (1469 to 1612)	9.43 (8.85 to 9.89)	5138 (3990 to 6532)	9.63 (7.48 to 12.24)	0.01 (-0.09 to 0.11)
Comoros	16 (10 to 22)	7.87 (5.04 to 10.42)	39 (29 to 50)	8.56 (6.39 to 10.8)	0.17 (0.07 to 0.27)
Congo	116 (77 to 154)	11.76 (8.29 to 15.34)	266 (197 to 352)	11.4 (8.76 to 14.52)	-0.17 (-0.36 to 0.02)
Cook Islands	1 (1 to 1)	8.39 (7.01 to 9.8)	2 (2 to 2)	7.77 (6.54 to 9.34)	-0.37 (-0.58 to -0.16)
Costa Rica	164 (152 to 174)	9.67 (8.94 to 10.28)	788 (622 to 986)	15.38 (12.15 to 19.28)	1.72 (1.6 to 1.84)
Côte d'Ivoire	307 (238 to 393)	8.89 (6.99 to 11.28)	850 (663 to 1066)	9.41 (7.62 to 11.47)	-0.02 (-0.12 to 0.08)
Croatia	1387 (1304 to 1472)	22.69 (21.28 to 24.04)	2308 (1843 to 2847)	25.28 (20.14 to 31.22)	0.73 (0.54 to 0.92)
Cuba	1571 (1485 to 1635)	15.5 (14.61 to 16.16)	3481 (2883 to 4186)	17.83 (14.73 to 21.48)	0.44 (0.31 to 0.57)
Cyprus	96 (84 to 109)	13.28 (11.7 to 15.1)	265 (230 to 301)	14.32 (12.4 to 16.32)	0.5 (0.28 to 0.73)
Czechia	4644 (4475 to 4785)	33.76 (32.41 to 34.8)	4678 (3864 to 5626)	21.58 (17.8 to 25.98)	-1.69 (-1.88 to -1.5)
Democratic People's Republic of Korea	1642 (1251 to 2130)	10.62 (8.4 to 13.46)	3442 (2576 to 4380)	10.89 (8.14 to 13.82)	0.19 (0.13 to 0.26)
Democratic Republic of the Congo	947 (738 to 1272)	6.87 (5.3 to 9.63)	1995 (1339 to 2956)	6.23 (4.15 to 9.51)	-0.49 (-0.81 to -0.17)
Denmark	2046 (1947 to 2122)	24.5 (23.43 to 25.37)	2647 (2396 to 2872)	21.75 (19.76 to 23.49)	-0.81 (-1.04 to -0.58)
Djibouti	11 (8 to 16)	9 (6.42 to 12.11)	59 (43 to 82)	11.19 (8.66 to 14.66)	0.78 (0.72 to 0.83)
Dominica	9 (8 to 10)	12.12 (10.75 to 13.52)	14 (11 to 16)	14.96 (12.42 to 18.09)	0.94 (0.87 to 1.01)
Dominican Republic	259 (228 to 291)	7.56 (6.6 to 8.51)	1115 (823 to 1448)	12.42 (9.23 to 16.14)	2.4 (2.19 to 2.61)
Ecuador	366 (343 to 388)	7.37 (6.85 to 7.83)	1603 (1285 to 2008)	11.34 (9.07 to 14.16)	2.09 (1.78 to 2.4)
Egypt	1552 (1423 to 1680)	5.46 (4.95 to 5.89)	4557 (3300 to 6275)	7.45 (5.4 to 10.11)	1.12 (1.04 to 1.19)
El Salvador	141 (130 to 151)	4.82 (4.4 to 5.18)	552 (426 to 710)	9.05 (6.98 to 11.68)	2.03 (1.71 to 2.35)

Countries	1990		2019		1990-2019
	Number of deaths (95% UI)	ASDR per 100,000 (95% UI)	Number of deaths (95% UI)	ASDR per 100,000 (95% UI)	EAPC of ASDR (95% CI)
Equatorial Guinea	12 (8 to 17)	6.52 (4.46 to 8.78)	61 (38 to 89)	14.24 (9.12 to 20.02)	3.51 (3.23 to 3.8)
Eritrea	62 (44 to 83)	6.55 (4.74 to 8.79)	247 (189 to 322)	9.97 (7.87 to 12.7)	1.19 (0.91 to 1.48)
Estonia	396 (378 to 414)	19.42 (18.49 to 20.27)	528 (425 to 660)	18.51 (14.81 to 23.22)	-0.1 (-0.31 to 0.11)
Eswatini	27 (20 to 34)	10.24 (7.87 to 13.05)	70 (47 to 96)	13.52 (9.32 to 18.23)	1.06 (0.71 to 1.42)
Ethiopia	1405 (1008 to 2166)	7.33 (5.07 to 11.43)	2855 (2128 to 4003)	7.33 (5.47 to 10.36)	-0.1 (-0.34 to 0.14)
Fiji	30 (24 to 36)	9.36 (7.69 to 11.17)	75 (59 to 93)	11.65 (9.51 to 14.2)	0.89 (0.71 to 1.07)
Finland	1050 (995 to 1093)	14.62 (13.81 to 15.22)	1616 (1455 to 1756)	12.33 (11.25 to 13.35)	-0.66 (-0.72 to -0.6)
France	20261 (19037 to 21246)	23.53 (22.15 to 24.58)	25497 (22330 to 27996)	16.41 (14.69 to 17.82)	-1.42 (-1.49 to -1.35)
Gabon	75 (50 to 111)	14.37 (9.71 to 21.11)	141 (103 to 177)	14.86 (11.25 to 18.24)	-0.01 (-0.08 to 0.06)
Gambia	15 (12 to 19)	4.72 (3.77 to 5.81)	58 (43 to 78)	6.56 (4.78 to 8.76)	0.99 (0.85 to 1.14)
Georgia	673 (619 to 731)	11.08 (10.21 to 12.04)	782 (659 to 913)	13.19 (11.08 to 15.44)	1.66 (1.11 to 2.2)
Germany	31780 (30043 to 33032)	24.4 (23.04 to 25.31)	37552 (34131 to 40326)	18.01 (16.54 to 19.26)	-1.45 (-2.06 to -0.84)
Ghana	354 (288 to 433)	6.28 (5.21 to 7.6)	1273 (1003 to 1615)	8.76 (6.96 to 10.92)	1.19 (1.16 to 1.22)
Greece	2132 (2017 to 2227)	14.19 (13.35 to 14.86)	4000 (3600 to 4305)	14.87 (13.67 to 15.87)	-0.36 (-0.58 to -0.13)
Greenland	9 (8 to 11)	29.76 (26.59 to 33.5)	20 (16 to 24)	31.38 (26.04 to 37.13)	-0.25 (-0.56 to 0.06)
Grenada	11 (10 to 12)	14.21 (12.93 to 15.52)	20 (18 to 22)	19.29 (17.44 to 21.14)	1.16 (1.03 to 1.28)
Guam	11 (9 to 12)	17.11 (13.91 to 20.21)	27 (22 to 31)	14.16 (12.02 to 16.74)	-0.64 (-1.02 to -0.26)
Guatemala	168 (149 to 187)	5.11 (4.57 to 5.65)	965 (771 to 1208)	9.09 (7.32 to 11.27)	1.87 (1.54 to 2.21)
Guinea	176 (148 to 205)	5.74 (4.81 to 6.67)	370 (282 to 480)	7.24 (5.51 to 9.24)	0.89 (0.86 to 0.92)
Guinea-Bissau	33 (22 to 41)	8.56 (6.18 to 10.77)	59 (44 to 74)	9.06 (6.93 to 11.29)	0.24 (0.2 to 0.28)

Countries	1990		2019		1990-2019
	Number of deaths (95% UI)	ASDR per 100,000 (95% UI)	Number of deaths (95% UI)	ASDR per 100,000 (95% UI)	EAPC of ASDR (95% CI)
Guyana	46 (40 to 52)	12.75 (11.2 to 14.37)	85 (68 to 107)	14.68 (11.87 to 18.25)	0.42 (0.27 to 0.58)
Haiti	301 (197 to 370)	10.03 (6.7 to 12.14)	671 (467 to 917)	10.53 (7.33 to 14.09)	0.35 (0.26 to 0.43)
Honduras	95 (76 to 111)	4.7 (3.72 to 5.53)	430 (303 to 605)	7.65 (5.34 to 10.67)	1.77 (1.64 to 1.91)
Hungary	4421 (4239 to 4587)	30.7 (29.38 to 31.89)	5646 (4699 to 6700)	28.56 (23.65 to 34.03)	-0.44 (-0.61 to -0.26)
Iceland	43 (40 to 46)	14.7 (13.54 to 15.74)	70 (61 to 78)	11.81 (10.53 to 13.16)	-0.93 (-1.11 to -0.76)
India	21971 (19162 to 25095)	5.47 (4.77 to 6.25)	79098 (67137 to 92724)	7.5 (6.35 to 8.76)	0.91 (0.77 to 1.06)
Indonesia	8267 (6315 to 9807)	8.94 (6.9 to 10.57)	30242 (20642 to 38807)	15.58 (10.54 to 19.86)	1.92 (1.83 to 2.02)
Iran (Islamic Republic of)	1665 (1369 to 1997)	7.17 (5.85 to 8.59)	6413 (5903 to 6908)	9.31 (8.53 to 10.08)	1.11 (0.95 to 1.27)
Iraq	541 (395 to 739)	7.2 (5.27 to 9.77)	1797 (1401 to 2258)	8.32 (6.58 to 10.16)	0.72 (0.52 to 0.93)
Ireland	1035 (991 to 1076)	25.68 (24.49 to 26.65)	1377 (1242 to 1498)	18.01 (16.26 to 19.51)	-1.38 (-1.44 to -1.32)
Israel	1036 (976 to 1085)	22.01 (20.69 to 23.07)	2008 (1797 to 2172)	16.56 (14.91 to 17.85)	-1.8 (-2.11 to -1.48)
Italy	17360 (16678 to 17759)	19.47 (18.63 to 19.95)	24877 (22016 to 26457)	15.83 (14.41 to 16.7)	-0.89 (-1 to -0.77)
Jamaica	193 (179 to 206)	10.65 (9.88 to 11.32)	501 (405 to 615)	16.52 (13.35 to 20.35)	1.64 (1.34 to 1.93)
Japan	30608 (29005 to 31368)	18.7 (17.61 to 19.23)	63742 (53256 to 69584)	15.85 (13.93 to 16.93)	-0.68 (-0.73 to -0.63)
Jordan	154 (125 to 188)	12.41 (9.99 to 15.13)	754 (628 to 901)	13.07 (10.93 to 15.46)	0.35 (0.24 to 0.45)
Kazakhstan	1881 (1788 to 1975)	15.15 (14.37 to 15.9)	2190 (1906 to 2491)	13.5 (11.79 to 15.28)	-0.17 (-0.32 to -0.02)
Kenya	392 (255 to 486)	5.13 (3.36 to 6.37)	1628 (1282 to 2038)	8.14 (6.52 to 10.04)	1.95 (1.77 to 2.14)
Kiribati	4 (3 to 5)	10.85 (8.88 to 12.99)	7 (5 to 9)	10.76 (8.26 to 14.05)	-0.2 (-0.26 to -0.14)
Kuwait	40 (36 to 44)	7.36 (6.44 to 8.29)	223 (187 to 265)	10.14 (8.41 to 12.04)	1.93 (1.46 to 2.41)
Kyrgyzstan	343 (321 to 366)	11.34 (10.63 to 12.12)	346 (304 to 394)	8.24 (7.25 to 9.34)	-1.17 (-1.38 to -0.96)

Countries	1990		2019		1990-2019
	Number of deaths (95% UI)	ASDR per 100,000 (95% UI)	Number of deaths (95% UI)	ASDR per 100,000 (95% UI)	EAPC of ASDR (95% CI)
Lao People's Democratic Republic	206 (139 to 274)	10.23 (7.16 to 13.55)	545 (389 to 721)	13.33 (9.6 to 17.23)	0.76 (0.7 to 0.82)
Latvia	643 (616 to 668)	17.98 (17.21 to 18.68)	746 (626 to 891)	17.63 (14.77 to 21.21)	-0.07 (-0.33 to 0.18)
Lebanon	275 (216 to 346)	13.42 (10.63 to 16.76)	910 (740 to 1199)	17.55 (14.26 to 23.15)	1.45 (1.27 to 1.63)
Lesotho	58 (45 to 83)	6.52 (5.12 to 9.38)	132 (96 to 174)	11.67 (8.66 to 15.1)	2.43 (2.25 to 2.61)
Liberia	70 (58 to 84)	6.81 (5.74 to 8.13)	120 (81 to 171)	6.68 (4.57 to 9.51)	0.43 (0.09 to 0.78)
Libya	216 (154 to 313)	11.93 (8.5 to 17.2)	620 (452 to 801)	12.45 (9.07 to 15.84)	0.37 (0.21 to 0.52)
Lithuania	746 (715 to 780)	16.62 (15.9 to 17.38)	1024 (857 to 1221)	16.9 (14.05 to 20.27)	0.09 (-0.11 to 0.29)
Luxembourg	147 (138 to 156)	27 (25.25 to 28.53)	170 (147 to 192)	15.97 (13.87 to 18.08)	-1.99 (-2.16 to -1.81)
Madagascar	333 (269 to 391)	6.82 (5.56 to 7.9)	714 (528 to 957)	7.11 (5.27 to 9.34)	0.08 (0.02 to 0.15)
Malawi	165 (139 to 191)	4.76 (4.03 to 5.41)	402 (308 to 507)	6.06 (4.74 to 7.49)	0.97 (0.82 to 1.12)
Malaysia	1368 (1200 to 1544)	16.26 (14.03 to 18.72)	4838 (3829 to 5962)	20.32 (16.15 to 24.85)	0.17 (-0.06 to 0.39)
Maldives	7 (5 to 9)	9.2 (6.96 to 11.44)	24 (19 to 28)	8.71 (7.13 to 10.38)	-0.66 (-0.83 to -0.48)
Mali	248 (210 to 290)	6.54 (5.56 to 7.59)	615 (487 to 773)	7.84 (6.29 to 9.73)	0.64 (0.59 to 0.69)
Malta	70 (65 to 75)	17 (15.79 to 18.12)	136 (120 to 154)	14.08 (12.38 to 15.89)	-0.58 (-0.68 to -0.48)
Marshall Islands	2 (1 to 2)	10.9 (8.99 to 13.23)	4 (3 to 5)	12.29 (9.55 to 15.44)	0.45 (0.4 to 0.51)
Mauritania	80 (60 to 98)	8.57 (6.45 to 10.46)	156 (119 to 195)	8.35 (6.53 to 10.34)	0.02 (-0.13 to 0.17)
Mauritius	56 (53 to 60)	8.21 (7.67 to 8.77)	215 (176 to 261)	12.77 (10.53 to 15.5)	1.53 (1.38 to 1.67)
Mexico	2600 (2496 to 2670)	6.52 (6.18 to 6.73)	10518 (9036 to 12022)	9.24 (7.95 to 10.52)	1.3 (1.21 to 1.39)
Micronesia (Federated States of)	5 (4 to 6)	11.29 (8.71 to 14.05)	8 (6 to 11)	13.08 (9.5 to 16.86)	0.44 (0.41 to 0.46)

Countries	1990		2019		1990-2019
	Number of deaths (95% UI)	ASDR per 100,000 (95% UI)	Number of deaths (95% UI)	ASDR per 100,000 (95% UI)	EAPC of ASDR (95% CI)
Monaco	17 (14 to 21)	22.69 (18.18 to 27.39)	25 (21 to 30)	24.27 (19.64 to 28.78)	0.35 (0.25 to 0.44)
Mongolia	89 (73 to 105)	8.76 (7.23 to 10.35)	193 (150 to 248)	9.31 (7.44 to 11.69)	-0.22 (-0.37 to -0.07)
Montenegro	86 (72 to 98)	14.21 (12.03 to 16.2)	168 (137 to 200)	17.23 (14.08 to 20.4)	1 (0.92 to 1.09)
Morocco	807 (643 to 943)	6.25 (4.88 to 7.34)	2482 (1856 to 3110)	8.48 (6.31 to 10.51)	1.01 (0.86 to 1.16)
Mozambique	266 (220 to 316)	5.15 (4.31 to 6)	832 (607 to 1092)	8.66 (6.46 to 11.2)	2.23 (2.09 to 2.37)
Myanmar	1989 (1548 to 2705)	8.91 (7.02 to 12.1)	5582 (4135 to 7152)	12.82 (9.6 to 16.23)	1.4 (1.31 to 1.49)
Namibia	38 (31 to 47)	5.74 (4.64 to 6.91)	113 (89 to 143)	8.68 (6.97 to 10.81)	1.57 (1.47 to 1.67)
Nauru	1 (0 to 1)	18.15 (12.23 to 25.69)	1 (0 to 1)	16.55 (11.75 to 21.15)	-0.48 (-0.72 to -0.25)
Nepal	313 (208 to 435)	3.62 (2.33 to 5.14)	1084 (781 to 1501)	5.4 (3.89 to 7.42)	1.42 (1.21 to 1.64)
Netherlands	4658 (4378 to 4846)	23.05 (21.68 to 23.95)	7774 (7036 to 8444)	21.75 (19.8 to 23.55)	-0.23 (-0.35 to -0.12)
New Zealand	1141 (1082 to 1191)	29.52 (27.87 to 30.79)	1648 (1481 to 1780)	20.26 (18.34 to 21.79)	-1.46 (-1.57 to -1.36)
Nicaragua	91 (76 to 106)	6.25 (5.12 to 7.49)	430 (362 to 502)	10.86 (9.23 to 12.5)	2.06 (1.81 to 2.31)
Niger	134 (102 to 176)	5.44 (4.17 to 7.06)	374 (276 to 510)	5.62 (4.24 to 7.51)	0.18 (0.09 to 0.28)
Nigeria	2486 (1798 to 3302)	6.39 (4.67 to 8.32)	6382 (4884 to 8138)	8.63 (6.75 to 10.78)	1.44 (1.26 to 1.63)
Niue	0 (0 to 0)	12.79 (10.66 to 15.3)	0 (0 to 0)	13.61 (10.77 to 17.01)	0.19 (0.15 to 0.22)
North Macedonia	270 (246 to 290)	15.05 (13.77 to 16.19)	683 (545 to 850)	22.13 (17.8 to 27.27)	1.48 (1.21 to 1.75)
Northern Mariana Islands	3 (2 to 4)	19.68 (15.87 to 23.99)	8 (7 to 9)	18.34 (15.88 to 20.86)	-0.23 (-0.36 to -0.1)
Norway	1611 (1519 to 1664)	22.69 (21.47 to 23.4)	2035 (1838 to 2176)	19.84 (18.04 to 21.11)	-0.46 (-0.59 to -0.32)
Oman	45 (31 to 62)	7.73 (5.36 to 10.43)	133 (108 to 167)	10.2 (8.48 to 12.04)	1.4 (1.19 to 1.62)
Pakistan	2968 (2546 to 3430)	5.43 (4.66 to 6.3)	8605 (6927 to 10776)	8.34 (6.72 to 10.41)	1.41 (1.17 to 1.64)

Countries	1990		2019		1990-2019
	Number of deaths (95% UI)	ASDR per 100,000 (95% UI)	Number of deaths (95% UI)	ASDR per 100,000 (95% UI)	EAPC of ASDR (95% CI)
Palau	1 (1 to 2)	13.78 (10.71 to 17.67)	2 (2 to 3)	13.69 (10.87 to 16.66)	-0.06 (-0.12 to 0.01)
Palestine	123 (84 to 169)	14.78 (10.08 to 20.25)	419 (358 to 487)	19.6 (16.69 to 22.68)	1.11 (0.88 to 1.35)
Panama	127 (118 to 136)	8.76 (8.06 to 9.36)	455 (357 to 576)	10.9 (8.54 to 13.81)	0.97 (0.89 to 1.05)
Papua New Guinea	106 (71 to 142)	6.31 (4.24 to 8.41)	318 (229 to 425)	7.46 (5.48 to 9.64)	0.5 (0.46 to 0.54)
Paraguay	135 (120 to 151)	6.34 (5.61 to 7.12)	652 (507 to 824)	12.16 (9.47 to 15.31)	2.57 (2.36 to 2.78)
Peru	896 (769 to 1036)	7.85 (6.73 to 9.07)	3035 (2291 to 3931)	9.44 (7.12 to 12.25)	0.8 (0.58 to 1.02)
Philippines	3762 (3351 to 4195)	13.14 (11.71 to 14.61)	11193 (9309 to 13497)	14.89 (12.46 to 17.81)	0.3 (0.03 to 0.57)
Poland	9180 (8821 to 9404)	21.44 (20.51 to 22)	17768 (15129 to 20758)	24.72 (20.98 to 28.96)	0.32 (0.16 to 0.48)
Portugal	2966 (2839 to 3080)	22.17 (21.13 to 23.03)	5189 (4700 to 5586)	20.11 (18.52 to 21.52)	-0.45 (-0.62 to -0.28)
Puerto Rico	487 (459 to 512)	13.69 (12.84 to 14.39)	1136 (892 to 1429)	15.44 (12.15 to 19.55)	0.31 (0.09 to 0.53)
Qatar	11 (9 to 15)	14.04 (10.44 to 17.84)	83 (61 to 110)	16.48 (12.93 to 20.54)	1.15 (0.84 to 1.45)
Republic of Korea	3204 (3051 to 3377)	11.81 (11.16 to 12.63)	12004 (10649 to 13220)	13.87 (12.22 to 15.3)	0.15 (-0.08 to 0.38)
Republic of Moldova	798 (766 to 834)	18.1 (17.38 to 18.9)	1028 (897 to 1164)	17.66 (15.45 to 20.01)	0.72 (0.16 to 1.28)
Romania	3548 (3418 to 3674)	12.86 (12.39 to 13.31)	7577 (6281 to 9022)	20.18 (16.61 to 24.11)	1.35 (1.16 to 1.55)
Russian Federation	29696 (28699 to 30771)	16.71 (16.11 to 17.33)	42834 (37637 to 48395)	18.17 (15.96 to 20.54)	0.03 (-0.24 to 0.3)
Rwanda	242 (174 to 307)	8.78 (6.5 to 11.05)	480 (377 to 608)	8.79 (7.08 to 10.83)	-0.34 (-0.55 to -0.13)
Saint Kitts and Nevis	7 (6 to 8)	18.91 (17.37 to 20.42)	11 (9 to 13)	18.32 (15.84 to 21.11)	0.06 (-0.09 to 0.22)
Saint Lucia	10 (9 to 11)	12.12 (11.23 to 12.99)	25 (21 to 29)	12.08 (10.24 to 14.11)	-0.39 (-0.67 to -0.11)
Saint Vincent and the Grenadines	8 (8 to 9)	11.83 (10.88 to 12.73)	19 (16 to 21)	14.42 (12.69 to 16.48)	0.64 (0.44 to 0.85)
Samoa	9 (7 to 10)	10.39 (8.79 to 12.45)	14 (11 to 17)	10.06 (8.18 to 12.34)	-0.18 (-0.22 to -0.13)

Countries	1990		2019		1990-2019
	Number of deaths (95% UI)	ASDR per 100,000 (95% UI)	Number of deaths (95% UI)	ASDR per 100,000 (95% UI)	EAPC of ASDR (95% CI)
San Marino	8 (7 to 9)	23.63 (20.36 to 27.36)	15 (11 to 21)	21.24 (14.31 to 29.61)	-0.16 (-0.25 to -0.07)
Sao Tome and Principe	6 (5 to 7)	9.79 (8.12 to 11.41)	14 (10 to 19)	15.18 (11.24 to 20.55)	1.58 (1.52 to 1.63)
Saudi Arabia	367 (251 to 509)	6.76 (4.56 to 9.32)	1629 (1263 to 2045)	9.66 (7.79 to 11.64)	1.13 (0.89 to 1.37)
Senegal	224 (180 to 266)	7.6 (6.21 to 8.98)	585 (467 to 731)	8.7 (7.13 to 10.77)	0.61 (0.45 to 0.77)
Serbia	2275 (1843 to 2539)	21.09 (16.95 to 23.71)	4018 (3269 to 4943)	25.38 (20.63 to 31.01)	0.83 (0.67 to 0.98)
Seychelles	9 (8 to 10)	16.5 (14.85 to 18.14)	26 (23 to 29)	25.33 (22.17 to 28.67)	1.12 (0.88 to 1.37)
Sierra Leone	111 (88 to 134)	6.17 (4.98 to 7.42)	225 (172 to 290)	6.96 (5.44 to 8.86)	0.64 (0.49 to 0.79)
Singapore	502 (479 to 522)	24.8 (23.48 to 25.94)	1111 (991 to 1197)	14.89 (13.22 to 16.12)	-2.06 (-2.19 to -1.94)
Slovakia	1411 (1340 to 1479)	23.75 (22.58 to 24.88)	2433 (1932 to 3038)	26.31 (20.96 to 32.8)	0.31 (0.13 to 0.49)
Slovenia	526 (413 to 662)	21.69 (17.07 to 27.36)	913 (720 to 1163)	19.84 (15.58 to 25.45)	-0.5 (-0.79 to -0.21)
Solomon Islands	11 (7 to 17)	8.48 (5.85 to 11.85)	31 (20 to 42)	10.43 (7.38 to 13.29)	0.6 (0.5 to 0.7)
Somalia	126 (86 to 191)	5.32 (3.71 to 8.01)	310 (197 to 580)	5.01 (3.17 to 9.27)	0 (-0.09 to 0.09)
South Africa	2030 (1721 to 2492)	10.39 (8.75 to 12.96)	4601 (4159 to 5198)	11.21 (10.13 to 12.6)	0.27 (0.03 to 0.51)
South Sudan	228 (137 to 371)	10.28 (6.28 to 16.58)	346 (223 to 528)	9.99 (6.57 to 14.78)	-0.03 (-0.11 to 0.05)
Spain	10285 (9693 to 10729)	18.95 (17.84 to 19.77)	20011 (17768 to 21746)	18.85 (17.05 to 20.21)	-0.01 (-0.06 to 0.05)
Sri Lanka	478 (433 to 525)	4.89 (4.41 to 5.37)	1510 (1129 to 1967)	6.41 (4.81 to 8.26)	1.32 (1.2 to 1.44)
Sudan	447 (340 to 664)	5.02 (3.85 to 7.52)	1256 (901 to 1851)	7.1 (5.26 to 10.59)	1.31 (1.21 to 1.41)
Suriname	31 (28 to 33)	12.33 (11.16 to 13.32)	94 (78 to 111)	16.26 (13.57 to 19.24)	1.06 (0.82 to 1.3)
Sweden	2854 (2672 to 2983)	18.2 (17.12 to 18.95)	3623 (3273 to 3863)	15.96 (14.67 to 16.96)	-0.54 (-0.68 to -0.4)
Switzerland	1513 (1424 to 1578)	14.06 (13.29 to 14.64)	2234 (1980 to 2431)	11.85 (10.7 to 12.84)	-0.85 (-1.03 to -0.67)

Countries	1990		2019		1990-2019
	Number of deaths (95% UI)	ASDR per 100,000 (95% UI)	Number of deaths (95% UI)	ASDR per 100,000 (95% UI)	EAPC of ASDR (95% CI)
Syrian Arab Republic	283 (219 to 360)	5.52 (4.27 to 6.93)	685 (499 to 908)	6.28 (4.7 to 8.15)	0.33 (0.12 to 0.54)
Taiwan (Province of China)	2343 (2258 to 2418)	15.54 (14.83 to 16.05)	10386 (8282 to 13153)	26.26 (20.88 to 33.29)	1.83 (1.61 to 2.04)
Tajikistan	260 (240 to 284)	8.87 (8.17 to 9.66)	428 (347 to 526)	10.22 (8.41 to 12.35)	1.13 (0.68 to 1.58)
Thailand	3572 (3165 to 3996)	10.78 (9.5 to 12.07)	10533 (7839 to 13572)	10.54 (7.88 to 13.56)	-0.62 (-0.89 to -0.35)
Timor-Leste	20 (15 to 27)	7.48 (5.61 to 9.89)	93 (64 to 119)	12.29 (8.48 to 15.79)	2.13 (1.88 to 2.38)
Togo	79 (66 to 94)	7.04 (5.94 to 8.35)	246 (181 to 320)	7.88 (5.9 to 10.04)	0.35 (0.31 to 0.38)
Tokelau	0 (0 to 0)	9.89 (7.94 to 12.21)	0 (0 to 0)	10.85 (8.14 to 13.95)	0.36 (0.33 to 0.39)
Tonga	3 (2 to 4)	6.04 (4.97 to 7.1)	5 (4 to 7)	6.91 (5.4 to 8.68)	0.37 (0.24 to 0.5)
Trinidad and Tobago	122 (114 to 128)	15.49 (14.48 to 16.34)	253 (194 to 326)	13.97 (10.74 to 17.96)	-0.53 (-0.65 to -0.4)
Tunisia	361 (292 to 431)	8.08 (6.56 to 9.66)	1154 (843 to 1546)	9.73 (7.17 to 12.98)	0.53 (0.45 to 0.61)
Turkey	4576 (3636 to 5557)	13.24 (10.44 to 16.04)	11194 (9055 to 13549)	13.11 (10.62 to 15.83)	0.26 (-0.21 to 0.72)
Turkmenistan	146 (139 to 154)	7.37 (7.01 to 7.76)	254 (207 to 314)	7.08 (5.81 to 8.71)	-0.38 (-0.81 to 0.05)
Tuvalu	1 (1 to 1)	10.08 (8.15 to 12.07)	1 (1 to 1)	10.9 (8.08 to 13.99)	0.12 (0.07 to 0.17)
Uganda	404 (330 to 474)	6.77 (5.61 to 7.86)	1519 (1194 to 1857)	11.55 (9.24 to 13.75)	1.84 (1.63 to 2.06)
Ukraine	15303 (14793 to 15836)	21.46 (20.74 to 22.22)	14582 (12548 to 16939)	19.18 (16.46 to 22.37)	-1.16 (-1.46 to -0.85)
United Arab Emirates	56 (37 to 77)	18.55 (11.88 to 24.75)	449 (306 to 608)	17.4 (11.94 to 24.35)	-0.31 (-0.73 to 0.12)
United Kingdom	22538 (21500 to 23104)	24.49 (23.35 to 25.09)	24220 (22210 to 25338)	18.09 (16.77 to 18.85)	-1.28 (-1.41 to -1.15)
United Republic of Tanzania	790 (655 to 965)	7.9 (6.61 to 9.52)	2180 (1732 to 2794)	9.51 (7.74 to 11.82)	0.71 (0.62 to 0.8)
United States of America	65582 (61888 to 67693)	20.05 (18.98 to 20.66)	84026 (77987 to 87516)	14.77 (13.86 to 15.32)	-1.29 (-1.39 to -1.18)
United States Virgin Islands	15 (13 to 17)	19.78 (16.9 to 22.59)	48 (40 to 55)	26.57 (22.3 to 31.01)	1.32 (1.11 to 1.53)

Countries	1990		2019		1990-2019
	Number of deaths (95% UI)	ASDR per 100,000 (95% UI)	Number of deaths (95% UI)	ASDR per 100,000 (95% UI)	EAPC of ASDR (95% CI)
Uruguay	1069 (1008 to 1114)	27.53 (25.97 to 28.72)	1520 (1389 to 1630)	26.12 (24.14 to 27.82)	-0.27 (-0.35 to -0.19)
Uzbekistan	818 (776 to 857)	7.04 (6.67 to 7.38)	1626 (1381 to 1901)	10.35 (9.07 to 11.79)	1.49 (1.37 to 1.62)
Vanuatu	5 (3 to 6)	7.69 (5.71 to 9.84)	15 (11 to 19)	9.29 (7.09 to 11.92)	0.5 (0.36 to 0.63)
Venezuela (Bolivarian Republic of)	800 (761 to 840)	8.65 (8.15 to 9.11)	3194 (2439 to 4131)	11.31 (8.72 to 14.59)	0.62 (0.48 to 0.76)
Viet Nam	3484 (2819 to 4170)	8.98 (7.27 to 10.68)	15557 (12175 to 18985)	17.5 (13.92 to 21.05)	2.64 (2.51 to 2.77)
Yemen	250 (161 to 364)	5.48 (3.53 to 7.97)	833 (626 to 1119)	6.66 (5.01 to 9.07)	1.01 (0.88 to 1.14)
Zambia	276 (208 to 350)	10.42 (8.01 to 13.09)	791 (581 to 1018)	12.62 (9.51 to 15.96)	0.6 (0.57 to 0.64)
Zimbabwe	362 (317 to 413)	10 (8.78 to 11.32)	818 (623 to 1031)	12.93 (9.93 to 16.2)	0.93 (0.71 to 1.16)

Abbreviations: ASDR, age-standardized death rate; EAPC, estimated annual percentage change; UI, uncertainty interval; CI, confidence

Supplementary Table S3. The death cases and age-standardized death rates of CRC attributable to all dietary risks in 1990 and 2019, and their temporal trends from 1990 to 2019.

Characteristics	1990		2019		1990-2019
	Number of deaths (95% UI)	ASDR per 100,000 (95% UI)	Number of deaths (95% UI)	ASDR per 100,000 (95% UI)	EAPC of ASDR (95% CI)
Global	179639 (138268 to 212910)	4.95 (3.82 to 5.88)	365752 (272831 to 442144)	4.61 (3.43 to 5.58)	-0.29 (-0.36 to -0.22)
Socio-demographic index					
Low SDI	5264 (4118 to 6470)	2.47 (1.93 to 3.02)	13113 (10344 to 15521)	2.8 (2.21 to 3.3)	0.44 (0.4 to 0.48)
Low-middle SDI	13450 (10851 to 16003)	2.48 (2.01 to 2.94)	40684 (31498 to 48681)	3.2 (2.48 to 3.82)	0.89 (0.84 to 0.94)
Middle SDI	31652 (25701 to 36920)	3.36 (2.74 to 3.91)	98821 (75848 to 119915)	4.22 (3.25 to 5.11)	0.96 (0.82 to 1.11)
High-middle SDI	57247 (42914 to 67890)	5.66 (4.23 to 6.74)	107586 (78263 to 131416)	5.35 (3.89 to 6.53)	-0.32 (-0.43 to -0.2)
High SDI	71931 (53299 to 88470)	6.86 (5.09 to 8.43)	105343 (76588 to 129174)	5.25 (3.83 to 6.41)	-1.04 (-1.11 to -0.98)
Region					
Andean Latin America	564 (449 to 676)	2.95 (2.35 to 3.53)	1898 (1353 to 2444)	3.48 (2.48 to 4.49)	0.74 (0.61 to 0.87)
Australasia	1964 (1470 to 2385)	8.5 (6.37 to 10.32)	2954 (2192 to 3624)	5.74 (4.3 to 7.02)	-1.66 (-1.77 to -1.54)
Caribbean	1111 (826 to 1321)	4.45 (3.31 to 5.3)	2502 (1734 to 3168)	4.84 (3.36 to 6.13)	0.25 (0.19 to 0.31)
Central Asia	1893 (1416 to 2257)	4.07 (3.04 to 4.86)	2610 (1915 to 3145)	3.91 (2.87 to 4.72)	0 (-0.11 to 0.12)
Central Europe	9516 (6656 to 11758)	6.65 (4.65 to 8.23)	15607 (10536 to 19885)	7.15 (4.84 to 9.09)	0.27 (0.16 to 0.38)
Central Latin America	1895 (1455 to 2278)	2.44 (1.88 to 2.94)	7018 (4940 to 8989)	3.04 (2.14 to 3.89)	0.73 (0.69 to 0.78)
Central Sub-Saharan Africa	591 (447 to 758)	2.98 (2.24 to 3.83)	1402 (1018 to 1854)	2.97 (2.14 to 4.03)	-0.1 (-0.31 to 0.12)
East Asia	31018 (24137 to 36894)	3.86 (3.03 to 4.6)	95246 (69351 to 119482)	4.85 (3.55 to 6.08)	1.15 (0.9 to 1.4)
Eastern Europe	17925 (13335 to 21588)	6.47 (4.83 to 7.8)	19858 (13871 to 25211)	5.72 (4 to 7.27)	-1.02 (-1.28 to -0.77)
Eastern Sub-Saharan	1878 (1470 to 2330)	2.71 (2.13 to 3.34)	4653 (3528 to 5753)	3.13 (2.4 to 3.85)	0.5 (0.44 to 0.56)

Africa					
High-income Asia Pacific	10886 (7945 to 13314)	5.68 (4.16 to 6.92)	25262 (18176 to 31456)	5.04 (3.64 to 6.2)	-0.42 (-0.51 to -0.33)
High-income North America	23253 (16965 to 28708)	6.48 (4.73 to 7.98)	30355 (21657 to 37839)	4.75 (3.43 to 5.9)	-1.22 (-1.31 to -1.12)
North Africa and Middle East	4572 (3322 to 5752)	2.89 (2.11 to 3.63)	13092 (9312 to 16064)	3.3 (2.37 to 4.05)	0.6 (0.43 to 0.77)
Oceania	79 (58 to 99)	3.01 (2.22 to 3.78)	211 (155 to 275)	3.39 (2.55 to 4.34)	0.4 (0.33 to 0.46)
South Asia	9650 (7685 to 11679)	1.96 (1.56 to 2.36)	31191 (23748 to 38373)	2.44 (1.87 to 3)	0.64 (0.53 to 0.76)
Southeast Asia	9939 (8112 to 11649)	4.16 (3.41 to 4.86)	32376 (24769 to 40353)	5.7 (4.37 to 7.08)	1 (0.94 to 1.06)
Southern Latin America	3569 (2757 to 4195)	8.07 (6.23 to 9.47)	6818 (5126 to 8144)	8.07 (6.06 to 9.62)	0.03 (-0.04 to 0.1)
Southern Sub-Saharan Africa	944 (734 to 1196)	3.75 (2.89 to 4.8)	2193 (1719 to 2668)	4.26 (3.35 to 5.17)	0.45 (0.23 to 0.66)
Tropical Latin America	2908 (2216 to 3505)	3.49 (2.67 to 4.21)	8984 (6693 to 11034)	3.78 (2.81 to 4.64)	0.31 (0.16 to 0.47)
Western Europe	43391 (31731 to 52971)	7.38 (5.41 to 9)	56341 (40795 to 69277)	5.67 (4.09 to 6.95)	-1.11 (-1.26 to -0.97)
Western Sub-Saharan Africa	2092 (1606 to 2643)	2.69 (2.08 to 3.38)	5181 (4002 to 6337)	3.2 (2.49 to 3.88)	0.77 (0.68 to 0.86)

Abbreviations: SDI, sociodemographic index; ASDR, age-standardized death rate; EAPC, estimated annual percentage change; UI, uncertainty interval; CI, confidence interval.

Supplementary Table S4. The death cases and age-standardized death rates of CRC attributable to all dietary risk factors in 204 countries and territories, and their temporal trends from 1990 to 2019.

Countries	Risk factors	1990		2019		1990-2019
		Number of deaths (95% UI)	ASDR per 100,000 (95% UI)	Number of deaths (95% UI)	ASDR per 100,000 (95% UI)	EAPC of ASDR (95% CI)
Afghanistan	All dietary risks	226 (133 to 344)	3.33 (2.06 to 4.96)	444 (283 to 623)	3.63 (2.53 to 4.93)	0.43 (0.29 to 0.57)
Afghanistan	Diet low in milk	113 (58 to 181)	1.67 (0.87 to 2.6)	218 (111 to 324)	1.78 (0.92 to 2.57)	0.43 (0.22 to 0.64)
Afghanistan	Diet low in fiber	10 (3 to 26)	0.16 (0.05 to 0.39)	37 (13 to 75)	0.3 (0.11 to 0.58)	1.74 (1.25 to 2.24)
Afghanistan	Diet low in calcium	119 (68 to 189)	1.77 (1.07 to 2.75)	232 (142 to 326)	1.92 (1.29 to 2.65)	0.34 (0.24 to 0.45)
Afghanistan	Diet high in red meat	16 (1 to 50)	0.23 (0.02 to 0.72)	25 (2 to 76)	0.2 (0.02 to 0.58)	-0.3 (-0.66 to 0.05)
Afghanistan	Diet high in processed meat	4 (1 to 9)	0.06 (0.02 to 0.13)	8 (2 to 16)	0.06 (0.02 to 0.12)	0.51 (0.2 to 0.83)
Afghanistan	Diet low in whole grains	113 (58 to 181)	1.67 (0.87 to 2.6)	218 (111 to 324)	1.78 (0.92 to 2.57)	0.43 (0.22 to 0.64)
Albania	All dietary risks	59 (43 to 72)	3.04 (2.23 to 3.71)	127 (82 to 180)	3.01 (1.94 to 4.27)	0.28 (0.03 to 0.54)
Albania	Diet low in milk	36 (18 to 45)	1.84 (0.92 to 2.29)	83 (40 to 119)	1.95 (0.95 to 2.8)	0.61 (0.34 to 0.88)
Albania	Diet low in fiber	3 (1 to 7)	0.18 (0.06 to 0.39)	4 (1 to 10)	0.1 (0.03 to 0.23)	-2.67 (-2.98 to -2.35)
Albania	Diet low in calcium	13 (6 to 22)	0.66 (0.33 to 1.16)	11 (5 to 19)	0.26 (0.12 to 0.45)	-3.45 (-3.74 to -3.17)
Albania	Diet high in red meat	5 (0 to 12)	0.23 (0.02 to 0.63)	18 (3 to 41)	0.42 (0.07 to 0.98)	2.88 (2.52 to 3.24)
Albania	Diet high in processed meat	11 (4 to 18)	0.57 (0.19 to 0.89)	27 (9 to 46)	0.64 (0.22 to 1.08)	0.82 (0.57 to 1.07)
Albania	Diet low in whole grains	36 (18 to 45)	1.84 (0.92 to 2.29)	83 (40 to 119)	1.95 (0.95 to 2.8)	0.61 (0.34 to 0.88)
Algeria	All dietary risks	268 (190 to 345)	2.67 (1.92 to 3.39)	745 (481 to 1001)	2.54 (1.63 to 3.39)	-0.26 (-0.29 to -0.22)
Algeria	Diet low in milk	149 (68 to 201)	1.46 (0.67 to 1.94)	475 (209 to 643)	1.6 (0.71 to 2.17)	0.39 (0.34 to 0.43)
Algeria	Diet low in fiber	14 (5 to 32)	0.15 (0.05 to 0.32)	17 (7 to 41)	0.06 (0.02 to 0.15)	-3.87 (-4.24 to -3.49)

Countries	Risk factors	1990 Number of deaths (95% UI)	1990 ASDR per 100,000 (95% UI)	2019 Number of deaths (95% UI)	2019 ASDR per 100,000 (95% UI)	1990-2019 EAPC of ASDR (95% CI)
Algeria	Diet low in calcium	120 (81 to 166)	1.23 (0.86 to 1.68)	255 (144 to 409)	0.9 (0.52 to 1.41)	-1.47 (-1.6 to -1.33)
Algeria	Diet high in red meat	15 (2 to 40)	0.14 (0.01 to 0.38)	52 (5 to 145)	0.17 (0.02 to 0.48)	0.83 (0.76 to 0.89)
Algeria	Diet high in processed meat	7 (2 to 12)	0.06 (0.02 to 0.11)	23 (5 to 45)	0.08 (0.02 to 0.14)	0.97 (0.87 to 1.06)
Algeria	Diet low in whole grains	149 (68 to 201)	1.46 (0.67 to 1.94)	475 (209 to 643)	1.6 (0.71 to 2.17)	0.39 (0.34 to 0.43)
American Samoa	All dietary risks	1 (1 to 1)	5.72 (4.1 to 7.18)	3 (2 to 3)	5.87 (4.33 to 7.4)	0.16 (0.03 to 0.29)
American Samoa	Diet low in milk	1 (0 to 1)	2.63 (1.01 to 3.54)	1 (0 to 2)	2.69 (1.01 to 3.69)	0.17 (0.04 to 0.3)
American Samoa	Diet low in fiber	0 (0 to 0)	0.04 (0.03 to 0.06)	0 (0 to 0)	0.04 (0.03 to 0.06)	-0.39 (-0.55 to -0.23)
American Samoa	Diet low in calcium	1 (0 to 1)	3.09 (2.22 to 4.08)	1 (1 to 2)	3.24 (2.4 to 4.26)	0.19 (0.07 to 0.32)
American Samoa	Diet high in red meat	0 (0 to 0)	0.55 (0.06 to 1.36)	0 (0 to 1)	0.52 (0.05 to 1.36)	-0.09 (-0.23 to 0.05)
American Samoa	Diet high in processed meat	0 (0 to 0)	0.15 (0.04 to 0.27)	0 (0 to 0)	0.15 (0.04 to 0.28)	0.29 (0.16 to 0.43)
American Samoa	Diet low in whole grains	1 (0 to 1)	2.63 (1.01 to 3.54)	1 (0 to 2)	2.69 (1.01 to 3.69)	0.17 (0.04 to 0.3)
Andorra	All dietary risks	4 (3 to 6)	8.9 (5.97 to 12.41)	10 (7 to 14)	7.09 (4.61 to 9.65)	-0.83 (-0.88 to -0.78)
Andorra	Diet low in milk	2 (1 to 4)	4.76 (1.73 to 7.06)	6 (2 to 8)	3.83 (1.38 to 5.37)	-0.79 (-0.85 to -0.74)
Andorra	Diet low in fiber	0 (0 to 0)	0.39 (0.13 to 0.92)	0 (0 to 1)	0.32 (0.1 to 0.72)	-0.93 (-1.08 to -0.78)
Andorra	Diet low in calcium	0 (0 to 1)	1 (0.44 to 1.79)	1 (0 to 2)	0.79 (0.34 to 1.39)	-1.05 (-1.18 to -0.91)
Andorra	Diet high in red meat	1 (0 to 2)	2.2 (0.69 to 4.12)	2 (1 to 5)	1.72 (0.45 to 3.26)	-0.86 (-0.93 to -0.79)
Andorra	Diet high in processed meat	1 (0 to 1)	1.68 (0.52 to 2.84)	2 (1 to 3)	1.35 (0.41 to 2.2)	-0.77 (-0.83 to -0.72)

Countries	Risk factors	1990 Number of deaths (95% UI)	1990 ASDR per 100,000 (95% UI)	2019 Number of deaths (95% UI)	2019 ASDR per 100,000 (95% UI)	1990-2019 EAPC of ASDR (95% CI)
Andorra	Diet low in whole grains	2 (1 to 4)	4.76 (1.73 to 7.06)	6 (2 to 8)	3.83 (1.38 to 5.37)	-0.79 (-0.85 to -0.74)
Angola	All dietary risks	118 (81 to 175)	3.36 (2.29 to 4.79)	363 (260 to 478)	3.72 (2.74 to 4.78)	0.35 (0.2 to 0.5)
Angola	Diet low in milk	47 (17 to 73)	1.32 (0.5 to 1.99)	146 (54 to 205)	1.49 (0.57 to 2.11)	0.45 (0.28 to 0.61)
Angola	Diet low in fiber	7 (2 to 15)	0.2 (0.06 to 0.45)	6 (3 to 15)	0.07 (0.03 to 0.17)	-4.78 (-5.23 to -4.32)
Angola	Diet low in calcium	74 (50 to 111)	2.11 (1.43 to 3.09)	224 (161 to 298)	2.32 (1.73 to 3.02)	0.29 (0.16 to 0.42)
Angola	Diet high in red meat	6 (1 to 18)	0.16 (0.02 to 0.47)	23 (2 to 63)	0.22 (0.02 to 0.61)	1.45 (1.22 to 1.67)
Angola	Diet high in processed meat	4 (1 to 9)	0.1 (0.02 to 0.22)	15 (2 to 31)	0.14 (0.02 to 0.28)	1.41 (1.21 to 1.6)
Angola	Diet low in whole grains	47 (17 to 73)	1.32 (0.5 to 1.99)	146 (54 to 205)	1.49 (0.57 to 2.11)	0.45 (0.28 to 0.61)
Antigua and Barbuda	All dietary risks	3 (2 to 3)	4.55 (3.44 to 5.43)	5 (4 to 7)	5.8 (4.17 to 7.19)	0.71 (0.57 to 0.84)
Antigua and Barbuda	Diet low in milk	1 (1 to 2)	2.49 (1.22 to 3.15)	3 (1 to 4)	3.22 (1.45 to 4.17)	0.76 (0.61 to 0.9)
Antigua and Barbuda	Diet low in fiber	0 (0 to 1)	0.85 (0.43 to 1.22)	1 (0 to 1)	0.9 (0.4 to 1.42)	-0.08 (-0.22 to 0.06)
Antigua and Barbuda	Diet low in calcium	1 (1 to 1)	1.53 (0.97 to 2.24)	2 (1 to 3)	1.99 (1.21 to 2.9)	0.79 (0.64 to 0.93)
Antigua and Barbuda	Diet high in red meat	0 (0 to 0)	0.3 (0.03 to 0.8)	0 (0 to 1)	0.39 (0.04 to 1.08)	0.9 (0.73 to 1.08)
Antigua and Barbuda	Diet high in processed meat	0 (0 to 0)	0.12 (0.03 to 0.21)	0 (0 to 0)	0.16 (0.04 to 0.3)	1.12 (0.96 to 1.27)
Antigua and Barbuda	Diet low in whole grains	1 (1 to 2)	2.49 (1.22 to 3.15)	3 (1 to 4)	3.22 (1.45 to 4.17)	0.76 (0.61 to 0.9)
Argentina	All dietary risks	2661 (2034 to 3139)	8.54 (6.55 to 10.08)	4879 (3646 to 5847)	8.93 (6.67 to 10.7)	0.14 (0.03 to 0.25)
Argentina	Diet low in milk	1278 (546 to 1613)	4.11 (1.75 to 5.19)	2218 (818 to 2874)	4.06 (1.5 to 5.25)	-0.12 (-0.24 to 0)
Argentina	Diet low in fiber	207 (71 to 392)	0.68 (0.24 to 1.28)	388 (139 to 738)	0.71 (0.25 to 1.35)	0.49 (0.32 to 0.66)

Countries	Risk factors	1990 Number of deaths (95% UI)	1990 ASDR per 100,000 (95% UI)	2019 Number of deaths (95% UI)	2019 ASDR per 100,000 (95% UI)	1990-2019 EAPC of ASDR (95% CI)
Argentina	Diet low in calcium	765 (472 to 1137)	2.48 (1.54 to 3.67)	1297 (734 to 2077)	2.36 (1.34 to 3.79)	-0.04 (-0.19 to 0.12)
Argentina	Diet high in red meat	742 (324 to 1129)	2.35 (1.02 to 3.58)	1417 (605 to 2160)	2.61 (1.12 to 3.97)	0.32 (0.21 to 0.42)
Argentina	Diet high in processed meat	175 (20 to 297)	0.56 (0.07 to 0.95)	431 (64 to 713)	0.8 (0.12 to 1.32)	1.24 (1.14 to 1.34)
Argentina	Diet low in whole grains	1278 (546 to 1613)	4.11 (1.75 to 5.19)	2218 (818 to 2874)	4.06 (1.5 to 5.25)	-0.12 (-0.24 to 0)
Armenia	All dietary risks	135 (98 to 161)	5.1 (3.69 to 6.11)	189 (128 to 245)	4.61 (3.14 to 5.96)	-0.59 (-0.69 to -0.48)
Armenia	Diet low in milk	79 (39 to 98)	2.98 (1.46 to 3.69)	126 (62 to 163)	3.06 (1.5 to 3.98)	0.11 (-0.01 to 0.23)
Armenia	Diet low in fiber	8 (3 to 18)	0.33 (0.11 to 0.7)	8 (3 to 20)	0.21 (0.07 to 0.5)	-2.64 (-3.1 to -2.18)
Armenia	Diet low in calcium	48 (30 to 69)	1.83 (1.18 to 2.62)	41 (21 to 71)	1.02 (0.52 to 1.75)	-2.91 (-3.21 to -2.61)
Armenia	Diet high in red meat	10 (1 to 29)	0.39 (0.03 to 1.06)	20 (2 to 51)	0.49 (0.05 to 1.25)	1.14 (0.86 to 1.43)
Armenia	Diet high in processed meat	9 (1 to 15)	0.31 (0.04 to 0.55)	14 (2 to 26)	0.35 (0.04 to 0.63)	0.58 (0.36 to 0.8)
Armenia	Diet low in whole grains	79 (39 to 98)	2.98 (1.46 to 3.69)	126 (62 to 163)	3.06 (1.5 to 3.98)	0.11 (-0.01 to 0.23)
Australia	All dietary risks	1562 (1171 to 1900)	8.12 (6.09 to 9.86)	2320 (1707 to 2869)	5.35 (3.99 to 6.58)	-1.8 (-1.94 to -1.66)
Australia	Diet low in milk	659 (249 to 885)	3.43 (1.29 to 4.61)	1023 (382 to 1379)	2.36 (0.88 to 3.16)	-1.67 (-1.82 to -1.53)
Australia	Diet low in fiber	134 (48 to 258)	0.69 (0.25 to 1.33)	153 (56 to 295)	0.35 (0.13 to 0.68)	-2.92 (-3.16 to -2.68)
Australia	Diet low in calcium	294 (142 to 519)	1.53 (0.74 to 2.71)	429 (206 to 764)	0.97 (0.46 to 1.73)	-2.03 (-2.2 to -1.86)
Australia	Diet high in red meat	506 (223 to 762)	2.62 (1.16 to 3.97)	722 (300 to 1124)	1.69 (0.72 to 2.61)	-1.81 (-1.99 to -1.64)
Australia	Diet high in processed meat	224 (56 to 350)	1.17 (0.29 to 1.83)	364 (98 to 565)	0.85 (0.23 to 1.31)	-1.32 (-1.45 to -1.18)
Australia	Diet low in whole grains	659 (249 to 885)	3.43 (1.29 to 4.61)	1023 (382 to 1379)	2.36 (0.88 to 3.16)	-1.67 (-1.82 to -1.53)

Countries	Risk factors	1990 Number of deaths (95% UI)	1990 ASDR per 100,000 (95% UI)	2019 Number of deaths (95% UI)	2019 ASDR per 100,000 (95% UI)	1990-2019 EAPC of ASDR (95% CI)
Austria	All dietary risks	1031 (754 to 1290)	8.58 (6.28 to 10.7)	787 (560 to 992)	4.11 (2.91 to 5.16)	-2.93 (-3.07 to -2.8)
Austria	Diet low in milk	472 (176 to 637)	3.92 (1.47 to 5.31)	390 (145 to 526)	2.03 (0.76 to 2.72)	-2.65 (-2.77 to -2.52)
Austria	Diet low in fiber	63 (21 to 140)	0.52 (0.18 to 1.13)	34 (12 to 80)	0.17 (0.06 to 0.4)	-4.26 (-4.51 to -4.01)
Austria	Diet low in calcium	202 (98 to 355)	1.64 (0.79 to 2.9)	145 (65 to 262)	0.74 (0.33 to 1.33)	-3.03 (-3.13 to -2.93)
Austria	Diet high in red meat	286 (91 to 472)	2.42 (0.79 to 3.95)	182 (48 to 329)	0.97 (0.27 to 1.73)	-3.71 (-3.98 to -3.44)
Austria	Diet high in processed meat	154 (29 to 243)	1.29 (0.25 to 2.03)	138 (34 to 215)	0.73 (0.19 to 1.13)	-2.28 (-2.44 to -2.12)
Austria	Diet low in whole grains	472 (176 to 637)	3.92 (1.47 to 5.31)	390 (145 to 526)	2.03 (0.76 to 2.72)	-2.65 (-2.77 to -2.52)
Azerbaijan	All dietary risks	210 (161 to 248)	4.15 (3.19 to 4.88)	368 (255 to 482)	4.24 (2.9 to 5.59)	0.41 (0.06 to 0.76)
Azerbaijan	Diet low in milk	113 (56 to 140)	2.23 (1.12 to 2.77)	227 (110 to 303)	2.6 (1.27 to 3.46)	1.15 (0.66 to 1.64)
Azerbaijan	Diet low in fiber	12 (4 to 26)	0.24 (0.08 to 0.52)	10 (3 to 24)	0.12 (0.04 to 0.31)	-3.48 (-3.87 to -3.08)
Azerbaijan	Diet low in calcium	88 (62 to 117)	1.75 (1.25 to 2.32)	107 (58 to 171)	1.28 (0.71 to 2.02)	-1.3 (-1.43 to -1.18)
Azerbaijan	Diet high in red meat	13 (1 to 35)	0.25 (0.02 to 0.68)	31 (3 to 86)	0.35 (0.03 to 0.96)	2.15 (1.39 to 2.92)
Azerbaijan	Diet high in processed meat	19 (3 to 31)	0.36 (0.06 to 0.61)	42 (8 to 73)	0.46 (0.08 to 0.81)	1.67 (1.02 to 2.32)
Azerbaijan	Diet low in whole grains	113 (56 to 140)	2.23 (1.12 to 2.77)	227 (110 to 303)	2.6 (1.27 to 3.46)	1.15 (0.66 to 1.64)
Bahamas	All dietary risks	9 (6 to 11)	5.84 (4.2 to 7.19)	23 (16 to 30)	6.26 (4.42 to 8.23)	0.45 (0.36 to 0.55)
Bahamas	Diet low in milk	4 (2 to 6)	2.99 (1.15 to 3.86)	12 (5 to 17)	3.37 (1.35 to 4.58)	0.67 (0.57 to 0.78)
Bahamas	Diet low in fiber	1 (0 to 2)	0.63 (0.23 to 1.13)	3 (1 to 5)	0.73 (0.27 to 1.28)	0.55 (0.41 to 0.68)
Bahamas	Diet low in calcium	3 (2 to 5)	2.22 (1.45 to 3.19)	8 (5 to 12)	2.16 (1.31 to 3.36)	0.02 (-0.1 to 0.13)

Countries	Risk factors	1990 Number of deaths (95% UI)	1990 ASDR per 100,000 (95% UI)	2019 Number of deaths (95% UI)	2019 ASDR per 100,000 (95% UI)	1990-2019 EAPC of ASDR (95% CI)
Bahamas	Diet high in red meat	1 (0 to 2)	0.63 (0.07 to 1.55)	3 (0 to 6)	0.67 (0.08 to 1.72)	0.61 (0.48 to 0.73)
Bahamas	Diet high in processed meat	0 (0 to 1)	0.19 (0.04 to 0.34)	1 (0 to 1)	0.2 (0.04 to 0.38)	0.5 (0.4 to 0.61)
Bahamas	Diet low in whole grains	4 (2 to 6)	2.99 (1.15 to 3.86)	12 (5 to 17)	3.37 (1.35 to 4.58)	0.67 (0.57 to 0.78)
Bahrain	All dietary risks	6 (4 to 8)	3.96 (2.69 to 5.08)	25 (15 to 34)	3.43 (2.16 to 4.6)	-0.66 (-0.93 to -0.39)
Bahrain	Diet low in milk	4 (2 to 5)	2.38 (1.05 to 3.18)	15 (6 to 22)	2.11 (0.83 to 2.95)	-0.56 (-0.84 to -0.28)
Bahrain	Diet low in fiber	0 (0 to 0)	0.07 (0.03 to 0.15)	0 (0 to 1)	0.05 (0.02 to 0.1)	-1.2 (-1.44 to -0.96)
Bahrain	Diet low in calcium	2 (1 to 3)	1.51 (0.91 to 2.26)	8 (5 to 14)	1.21 (0.7 to 1.96)	-0.94 (-1.2 to -0.68)
Bahrain	Diet high in red meat	1 (0 to 1)	0.33 (0.03 to 0.88)	2 (0 to 6)	0.29 (0.03 to 0.81)	-0.54 (-0.81 to -0.26)
Bahrain	Diet high in processed meat	0 (0 to 0)	0.12 (0.03 to 0.22)	1 (0 to 2)	0.12 (0.03 to 0.23)	-0.33 (-0.62 to -0.04)
Bahrain	Diet low in whole grains	4 (2 to 5)	2.38 (1.05 to 3.18)	15 (6 to 22)	2.11 (0.83 to 2.95)	-0.56 (-0.84 to -0.28)
Bangladesh	All dietary risks	910 (623 to 1232)	2.08 (1.4 to 2.83)	2568 (1730 to 3797)	2.11 (1.42 to 3.12)	-0.05 (-0.2 to 0.09)
Bangladesh	Diet low in milk	244 (95 to 395)	0.56 (0.22 to 0.9)	691 (242 to 1204)	0.57 (0.2 to 0.98)	-0.02 (-0.17 to 0.12)
Bangladesh	Diet low in fiber	199 (105 to 303)	0.46 (0.24 to 0.7)	493 (235 to 824)	0.41 (0.2 to 0.68)	-0.57 (-0.72 to -0.41)
Bangladesh	Diet low in calcium	574 (380 to 815)	1.31 (0.86 to 1.88)	1616 (1051 to 2412)	1.33 (0.87 to 1.99)	-0.06 (-0.21 to 0.09)
Bangladesh	Diet high in red meat	15 (4 to 34)	0.03 (0.01 to 0.08)	51 (11 to 131)	0.04 (0.01 to 0.11)	0.71 (0.57 to 0.84)
Bangladesh	Diet high in processed meat	50 (6 to 94)	0.11 (0.01 to 0.21)	167 (21 to 344)	0.13 (0.02 to 0.28)	0.69 (0.56 to 0.82)
Bangladesh	Diet low in whole grains	244 (95 to 395)	0.56 (0.22 to 0.9)	691 (242 to 1204)	0.57 (0.2 to 0.98)	-0.02 (-0.17 to 0.12)
Barbados	All dietary risks	17 (12 to 21)	5.68 (4.05 to 7.11)	37 (25 to 48)	7.54 (5.1 to 9.76)	0.89 (0.69 to 1.09)

Countries	Risk factors	1990 Number of deaths (95% UI)	1990 ASDR per 100,000 (95% UI)	2019 Number of deaths (95% UI)	2019 ASDR per 100,000 (95% UI)	1990-2019 EAPC of ASDR (95% CI)
Barbados	Diet low in milk	9 (3 to 11)	2.86 (1.08 to 3.69)	19 (7 to 26)	3.92 (1.47 to 5.36)	1.04 (0.83 to 1.26)
Barbados	Diet low in fiber	2 (1 to 3)	0.59 (0.21 to 1.07)	3 (1 to 7)	0.68 (0.24 to 1.33)	0.5 (0.38 to 0.62)
Barbados	Diet low in calcium	7 (4 to 10)	2.18 (1.37 to 3.19)	14 (9 to 21)	2.93 (1.82 to 4.34)	0.79 (0.55 to 1.04)
Barbados	Diet high in red meat	2 (0 to 5)	0.61 (0.06 to 1.52)	3 (0 to 9)	0.67 (0.06 to 1.87)	0.37 (0.25 to 0.5)
Barbados	Diet high in processed meat	1 (0 to 1)	0.22 (0.04 to 0.39)	1 (0 to 3)	0.29 (0.05 to 0.54)	1.05 (0.87 to 1.23)
Barbados	Diet low in whole grains	9 (3 to 11)	2.86 (1.08 to 3.69)	19 (7 to 26)	3.92 (1.47 to 5.36)	1.04 (0.83 to 1.26)
Belarus	All dietary risks	797 (590 to 956)	6.2 (4.59 to 7.44)	911 (628 to 1224)	5.67 (3.9 to 7.61)	-1.11 (-1.41 to -0.82)
Belarus	Diet low in milk	481 (237 to 598)	3.74 (1.84 to 4.64)	527 (221 to 732)	3.28 (1.38 to 4.55)	-1.31 (-1.63 to -1)
Belarus	Diet low in fiber	7 (5 to 13)	0.06 (0.04 to 0.11)	12 (6 to 27)	0.07 (0.04 to 0.17)	0.05 (-0.69 to 0.8)
Belarus	Diet low in calcium	125 (58 to 220)	0.98 (0.45 to 1.73)	148 (68 to 276)	0.92 (0.42 to 1.71)	-1.17 (-1.71 to -0.62)
Belarus	Diet high in red meat	164 (44 to 290)	1.27 (0.34 to 2.25)	199 (55 to 382)	1.25 (0.35 to 2.4)	-0.54 (-1.01 to -0.07)
Belarus	Diet high in processed meat	134 (41 to 202)	1.04 (0.32 to 1.56)	146 (42 to 241)	0.91 (0.26 to 1.5)	-1.26 (-1.57 to -0.95)
Belarus	Diet low in whole grains	481 (237 to 598)	3.74 (1.84 to 4.64)	527 (221 to 732)	3.28 (1.38 to 4.55)	-1.31 (-1.63 to -1)
Belgium	All dietary risks	1226 (864 to 1542)	7.86 (5.55 to 9.9)	1382 (976 to 1713)	5.5 (3.91 to 6.82)	-1.29 (-1.39 to -1.2)
Belgium	Diet low in milk	640 (238 to 837)	4.1 (1.53 to 5.36)	710 (263 to 928)	2.81 (1.04 to 3.66)	-1.41 (-1.53 to -1.28)
Belgium	Diet low in fiber	97 (34 to 198)	0.62 (0.21 to 1.26)	93 (32 to 198)	0.35 (0.12 to 0.75)	-1.9 (-2.04 to -1.76)
Belgium	Diet low in calcium	232 (111 to 405)	1.46 (0.7 to 2.57)	209 (97 to 364)	0.8 (0.37 to 1.39)	-2.04 (-2.31 to -1.76)
Belgium	Diet high in red meat	191 (35 to 415)	1.25 (0.23 to 2.7)	298 (83 to 540)	1.25 (0.36 to 2.22)	0 (-0.23 to 0.24)

Countries	Risk factors	1990 Number of deaths (95% UI)	1990 ASDR per 100,000 (95% UI)	2019 Number of deaths (95% UI)	2019 ASDR per 100,000 (95% UI)	1990-2019 EAPC of ASDR (95% CI)
Belgium	Diet high in processed meat	229 (66 to 346)	1.48 (0.44 to 2.23)	265 (82 to 409)	1.07 (0.33 to 1.63)	-1.25 (-1.33 to -1.16)
Belgium	Diet low in whole grains	640 (238 to 837)	4.1 (1.53 to 5.36)	710 (263 to 928)	2.81 (1.04 to 3.66)	-1.41 (-1.53 to -1.28)
Belize	All dietary risks	2 (1 to 2)	2.2 (1.53 to 2.71)	8 (6 to 10)	3.14 (2.13 to 3.98)	1.1 (0.67 to 1.53)
Belize	Diet low in milk	1 (0 to 1)	1.14 (0.44 to 1.51)	4 (2 to 6)	1.63 (0.61 to 2.22)	1.06 (0.62 to 1.51)
Belize	Diet low in fiber	0 (0 to 0)	0.11 (0.04 to 0.27)	0 (0 to 1)	0.11 (0.04 to 0.27)	-0.19 (-0.54 to 0.16)
Belize	Diet low in calcium	1 (1 to 1)	0.97 (0.63 to 1.39)	4 (2 to 5)	1.4 (0.91 to 2.02)	1.19 (0.79 to 1.6)
Belize	Diet high in red meat	0 (0 to 0)	0.15 (0.02 to 0.41)	1 (0 to 2)	0.21 (0.02 to 0.57)	0.93 (0.51 to 1.36)
Belize	Diet high in processed meat	0 (0 to 0)	0.07 (0.02 to 0.13)	0 (0 to 1)	0.12 (0.02 to 0.22)	1.59 (1.1 to 2.08)
Belize	Diet low in whole grains	1 (0 to 1)	1.14 (0.44 to 1.51)	4 (2 to 6)	1.63 (0.61 to 2.22)	1.06 (0.62 to 1.51)
Benin	All dietary risks	48 (38 to 59)	2.59 (2.06 to 3.17)	123 (90 to 163)	2.86 (2.14 to 3.73)	0.42 (0.38 to 0.46)
Benin	Diet low in milk	19 (7 to 26)	1.01 (0.38 to 1.37)	47 (17 to 69)	1.1 (0.41 to 1.6)	0.36 (0.33 to 0.4)
Benin	Diet low in fiber	0 (0 to 1)	0.02 (0.01 to 0.04)	1 (1 to 1)	0.02 (0.01 to 0.03)	0.19 (0 to 0.38)
Benin	Diet low in calcium	31 (24 to 40)	1.69 (1.29 to 2.13)	78 (58 to 105)	1.82 (1.37 to 2.42)	0.34 (0.29 to 0.38)
Benin	Diet high in red meat	2 (0 to 5)	0.1 (0.01 to 0.25)	5 (1 to 12)	0.1 (0.02 to 0.26)	0.33 (0.21 to 0.46)
Benin	Diet high in processed meat	3 (0 to 5)	0.14 (0.02 to 0.26)	8 (1 to 16)	0.18 (0.02 to 0.35)	1.17 (1.07 to 1.27)
Benin	Diet low in whole grains	19 (7 to 26)	1.01 (0.38 to 1.37)	47 (17 to 69)	1.1 (0.41 to 1.6)	0.36 (0.33 to 0.4)
Bermuda	All dietary risks	5 (3 to 6)	8.12 (5.52 to 10.41)	9 (6 to 11)	6.3 (4.39 to 8.12)	-0.82 (-0.88 to -0.77)
Bermuda	Diet low in milk	3 (1 to 3)	4.48 (1.69 to 5.81)	4 (2 to 6)	3.19 (1.21 to 4.42)	-1.13 (-1.24 to -1.03)

Countries	Risk factors	1990 Number of deaths (95% UI)	1990 ASDR per 100,000 (95% UI)	2019 Number of deaths (95% UI)	2019 ASDR per 100,000 (95% UI)	1990-2019 EAPC of ASDR (95% CI)
Bermuda	Diet low in fiber	0 (0 to 1)	0.45 (0.14 to 1.06)	1 (0 to 2)	0.64 (0.23 to 1.21)	1.31 (0.97 to 1.65)
Bermuda	Diet low in calcium	1 (1 to 2)	2.54 (1.36 to 4.2)	3 (2 to 5)	2.18 (1.29 to 3.34)	-0.55 (-0.65 to -0.46)
Bermuda	Diet high in red meat	1 (0 to 2)	1.28 (0.22 to 2.87)	1 (0 to 3)	0.97 (0.18 to 2.07)	-0.78 (-0.91 to -0.65)
Bermuda	Diet high in processed meat	0 (0 to 0)	0.31 (0.07 to 0.54)	0 (0 to 1)	0.21 (0.05 to 0.39)	-1.18 (-1.34 to -1.02)
Bermuda	Diet low in whole grains	3 (1 to 3)	4.48 (1.69 to 5.81)	4 (2 to 6)	3.19 (1.21 to 4.42)	-1.13 (-1.24 to -1.03)
Bhutan	All dietary risks	4 (2 to 6)	1.9 (1.07 to 2.74)	13 (8 to 19)	2.57 (1.62 to 3.66)	1.04 (1.02 to 1.05)
Bhutan	Diet low in milk	1 (1 to 2)	0.65 (0.25 to 1.07)	5 (2 to 8)	0.96 (0.36 to 1.49)	1.36 (1.34 to 1.38)
Bhutan	Diet low in fiber	1 (0 to 1)	0.24 (0.09 to 0.43)	1 (0 to 2)	0.22 (0.07 to 0.45)	-0.36 (-0.38 to -0.34)
Bhutan	Diet low in calcium	2 (1 to 4)	1.15 (0.65 to 1.71)	7 (4 to 11)	1.46 (0.89 to 2.13)	0.81 (0.79 to 0.82)
Bhutan	Diet high in red meat	0 (0 to 0)	0.06 (0.01 to 0.16)	1 (0 to 1)	0.1 (0.01 to 0.26)	1.7 (1.66 to 1.75)
Bhutan	Diet high in processed meat	0 (0 to 1)	0.12 (0.01 to 0.25)	1 (0 to 2)	0.21 (0.03 to 0.41)	1.98 (1.95 to 2.01)
Bhutan	Diet low in whole grains	1 (1 to 2)	0.65 (0.25 to 1.07)	5 (2 to 8)	0.96 (0.36 to 1.49)	1.36 (1.34 to 1.38)
Bolivia (Plurinational State of)	All dietary risks	109 (75 to 142)	3.73 (2.61 to 4.82)	398 (261 to 549)	4.94 (3.23 to 6.76)	0.9 (0.87 to 0.94)
Bolivia (Plurinational State of)	Diet low in milk	43 (17 to 61)	1.47 (0.58 to 2.06)	154 (59 to 232)	1.91 (0.72 to 2.87)	0.84 (0.79 to 0.89)
Bolivia (Plurinational State of)	Diet low in fiber	6 (2 to 13)	0.2 (0.07 to 0.45)	22 (7 to 51)	0.28 (0.1 to 0.65)	1.11 (0.89 to 1.33)
Bolivia (Plurinational State of)	Diet low in calcium	67 (45 to 92)	2.31 (1.58 to 3.1)	240 (152 to 334)	2.99 (1.89 to 4.13)	0.82 (0.78 to 0.85)

Countries	Risk factors	1990 Number of deaths (95% UI)	1990 ASDR per 100,000 (95% UI)	2019 Number of deaths (95% UI)	2019 ASDR per 100,000 (95% UI)	1990-2019 EAPC of ASDR (95% CI)
Bolivia (Plurinational State of)	Diet high in red meat	9 (1 to 23)	0.31 (0.03 to 0.79)	37 (4 to 96)	0.45 (0.05 to 1.17)	1.33 (1.24 to 1.43)
Bolivia (Plurinational State of)	Diet high in processed meat	2 (1 to 3)	0.05 (0.03 to 0.09)	7 (3 to 12)	0.08 (0.03 to 0.14)	1.44 (1.4 to 1.48)
Bolivia (Plurinational State of)	Diet low in whole grains	43 (17 to 61)	1.47 (0.58 to 2.06)	154 (59 to 232)	1.91 (0.72 to 2.87)	0.84 (0.79 to 0.89)
Bosnia and Herzegovina	All dietary risks	167 (119 to 205)	4.45 (3.19 to 5.46)	340 (202 to 467)	5.76 (3.42 to 7.89)	1.13 (0.91 to 1.35)
Bosnia and Herzegovina	Diet low in milk	86 (32 to 113)	2.28 (0.85 to 2.98)	196 (72 to 280)	3.31 (1.21 to 4.73)	1.59 (1.33 to 1.84)
Bosnia and Herzegovina	Diet low in fiber	2 (1 to 3)	0.05 (0.03 to 0.09)	4 (3 to 6)	0.06 (0.04 to 0.11)	0.96 (0.71 to 1.21)
Bosnia and Herzegovina	Diet low in calcium	69 (44 to 101)	1.87 (1.21 to 2.73)	89 (43 to 155)	1.52 (0.73 to 2.62)	-0.77 (-0.95 to -0.59)
Bosnia and Herzegovina	Diet high in red meat	10 (1 to 27)	0.27 (0.03 to 0.7)	31 (3 to 89)	0.53 (0.05 to 1.51)	3.03 (2.67 to 3.4)
Bosnia and Herzegovina	Diet high in processed meat	20 (3 to 33)	0.51 (0.08 to 0.84)	52 (10 to 92)	0.89 (0.17 to 1.55)	2.43 (2.09 to 2.76)
Bosnia and Herzegovina	Diet low in whole grains	86 (32 to 113)	2.28 (0.85 to 2.98)	196 (72 to 280)	3.31 (1.21 to 4.73)	1.59 (1.33 to 1.84)
Botswana	All dietary risks	18 (12 to 24)	3.66 (2.6 to 4.92)	60 (38 to 85)	5.04 (3.3 to 7.03)	0.82 (0.52 to 1.13)
Botswana	Diet low in milk	8 (3 to 12)	1.58 (0.59 to 2.34)	28 (10 to 42)	2.32 (0.84 to 3.41)	1.04 (0.73 to 1.34)
Botswana	Diet low in fiber	1 (0 to 2)	0.17 (0.06 to 0.4)	3 (1 to 7)	0.23 (0.08 to 0.55)	0.75 (0.15 to 1.35)
Botswana	Diet low in calcium	10 (7 to 13)	1.96 (1.39 to 2.69)	29 (18 to 43)	2.46 (1.55 to 3.63)	0.51 (0.21 to 0.81)
Botswana	Diet high in red meat	2 (0 to 4)	0.31 (0.02 to 0.84)	6 (0 to 16)	0.46 (0.04 to 1.29)	1.05 (0.82 to 1.29)
Botswana	Diet high in processed meat	1 (0 to 1)	0.11 (0.02 to 0.22)	2 (0 to 5)	0.18 (0.03 to 0.36)	1.35 (1.07 to 1.62)

Countries	Risk factors	1990 Number of deaths (95% UI)	1990 ASDR per 100,000 (95% UI)	2019 Number of deaths (95% UI)	2019 ASDR per 100,000 (95% UI)	1990-2019 EAPC of ASDR (95% CI)
Botswana	Diet low in whole grains	8 (3 to 12)	1.58 (0.59 to 2.34)	28 (10 to 42)	2.32 (0.84 to 3.41)	1.04 (0.73 to 1.34)
Brazil	All dietary risks	2858 (2180 to 3447)	3.53 (2.7 to 4.24)	8766 (6546 to 10783)	3.78 (2.8 to 4.64)	0.27 (0.12 to 0.43)
Brazil	Diet low in milk	1256 (468 to 1657)	1.54 (0.58 to 2.03)	3874 (1445 to 5213)	1.67 (0.62 to 2.25)	0.33 (0.18 to 0.47)
Brazil	Diet low in fiber	223 (87 to 420)	0.28 (0.11 to 0.52)	496 (197 to 1013)	0.21 (0.08 to 0.44)	-1.26 (-1.39 to -1.12)
Brazil	Diet low in calcium	1308 (943 to 1756)	1.64 (1.18 to 2.19)	2799 (1652 to 4311)	1.22 (0.72 to 1.87)	-1.3 (-1.43 to -1.17)
Brazil	Diet high in red meat	375 (73 to 814)	0.45 (0.09 to 0.99)	2355 (877 to 3841)	1 (0.37 to 1.64)	3.06 (2.44 to 3.69)
Brazil	Diet high in processed meat	110 (19 to 191)	0.13 (0.02 to 0.23)	492 (67 to 856)	0.21 (0.03 to 0.36)	2.03 (1.91 to 2.15)
Brazil	Diet low in whole grains	1256 (468 to 1657)	1.54 (0.58 to 2.03)	3874 (1445 to 5213)	1.67 (0.62 to 2.25)	0.33 (0.18 to 0.47)
Brunei Darussalam	All dietary risks	8 (6 to 10)	9.65 (6.83 to 12.49)	23 (16 to 29)	9.54 (6.65 to 11.98)	0.64 (0.35 to 0.92)
Brunei Darussalam	Diet low in milk	4 (1 to 5)	4.34 (1.62 to 6.06)	11 (4 to 15)	4.5 (1.68 to 6.19)	0.79 (0.53 to 1.05)
Brunei Darussalam	Diet low in fiber	1 (0 to 1)	0.69 (0.24 to 1.5)	3 (1 to 5)	1.08 (0.37 to 1.99)	2.54 (2.19 to 2.89)
Brunei Darussalam	Diet low in calcium	3 (2 to 5)	4.22 (2.65 to 6.09)	8 (5 to 13)	3.68 (2.28 to 5.47)	0.14 (-0.16 to 0.45)
Brunei Darussalam	Diet high in red meat	1 (0 to 2)	0.94 (0.08 to 2.51)	2 (0 to 5)	0.76 (0.07 to 2.1)	-0.09 (-0.41 to 0.22)
Brunei Darussalam	Diet high in processed meat	1 (0 to 1)	0.75 (0.1 to 1.35)	2 (0 to 4)	0.79 (0.1 to 1.41)	0.94 (0.64 to 1.23)
Brunei Darussalam	Diet low in whole grains	4 (1 to 5)	4.34 (1.62 to 6.06)	11 (4 to 15)	4.5 (1.68 to 6.19)	0.79 (0.53 to 1.05)
Bulgaria	All dietary risks	793 (560 to 978)	6.5 (4.61 to 8.02)	1266 (869 to 1715)	8.68 (5.94 to 11.8)	2.29 (1.8 to 2.78)
Bulgaria	Diet low in milk	434 (173 to 555)	3.55 (1.41 to 4.55)	619 (227 to 871)	4.23 (1.57 to 5.97)	1.83 (1.36 to 2.31)

Countries	Risk factors	1990 Number of deaths (95% UI)	1990 ASDR per 100,000 (95% UI)	2019 Number of deaths (95% UI)	2019 ASDR per 100,000 (95% UI)	1990-2019 EAPC of ASDR (95% CI)
Bulgaria	Diet low in fiber	53 (18 to 114)	0.44 (0.15 to 0.95)	96 (33 to 198)	0.65 (0.22 to 1.34)	2.27 (1.68 to 2.86)
Bulgaria	Diet low in calcium	184 (95 to 319)	1.52 (0.78 to 2.63)	326 (176 to 545)	2.2 (1.18 to 3.66)	2.34 (1.83 to 2.85)
Bulgaria	Diet high in red meat	137 (29 to 275)	1.12 (0.25 to 2.24)	241 (62 to 471)	1.7 (0.47 to 3.32)	3.25 (2.52 to 3.98)
Bulgaria	Diet high in processed meat	98 (17 to 159)	0.8 (0.14 to 1.3)	181 (43 to 301)	1.26 (0.31 to 2.08)	3.3 (2.6 to 3.99)
Bulgaria	Diet low in whole grains	434 (173 to 555)	3.55 (1.41 to 4.55)	619 (227 to 871)	4.23 (1.57 to 5.97)	1.83 (1.36 to 2.31)
Burkina Faso	All dietary risks	92 (69 to 120)	2.38 (1.81 to 3.09)	225 (168 to 298)	2.82 (2.14 to 3.66)	0.6 (0.45 to 0.75)
Burkina Faso	Diet low in milk	37 (14 to 53)	0.95 (0.36 to 1.36)	91 (34 to 130)	1.14 (0.43 to 1.62)	0.65 (0.5 to 0.8)
Burkina Faso	Diet low in fiber	1 (1 to 3)	0.04 (0.02 to 0.09)	2 (1 to 4)	0.03 (0.01 to 0.05)	-1.34 (-1.75 to -0.93)
Burkina Faso	Diet low in calcium	58 (43 to 76)	1.51 (1.13 to 1.98)	136 (99 to 181)	1.72 (1.27 to 2.28)	0.45 (0.3 to 0.6)
Burkina Faso	Diet high in red meat	5 (0 to 13)	0.12 (0.01 to 0.33)	14 (1 to 39)	0.17 (0.01 to 0.47)	1.23 (1.17 to 1.29)
Burkina Faso	Diet high in processed meat	5 (1 to 9)	0.12 (0.01 to 0.23)	15 (2 to 27)	0.17 (0.02 to 0.32)	1.41 (1.31 to 1.5)
Burkina Faso	Diet low in whole grains	37 (14 to 53)	0.95 (0.36 to 1.36)	91 (34 to 130)	1.14 (0.43 to 1.62)	0.65 (0.5 to 0.8)
Burundi	All dietary risks	65 (49 to 85)	2.93 (2.2 to 3.78)	117 (79 to 172)	2.8 (1.93 to 4.05)	-0.32 (-0.39 to -0.25)
Burundi	Diet low in milk	29 (11 to 41)	1.29 (0.47 to 1.82)	51 (18 to 81)	1.21 (0.44 to 1.9)	-0.38 (-0.46 to -0.31)
Burundi	Diet low in fiber	0 (0 to 1)	0.02 (0.01 to 0.03)	1 (1 to 2)	0.03 (0.01 to 0.05)	1.25 (1.15 to 1.35)
Burundi	Diet low in calcium	40 (28 to 52)	1.8 (1.32 to 2.33)	74 (50 to 110)	1.78 (1.21 to 2.61)	-0.2 (-0.28 to -0.13)
Burundi	Diet high in red meat	2 (0 to 4)	0.07 (0.02 to 0.17)	2 (1 to 6)	0.06 (0.02 to 0.14)	-0.99 (-1.22 to -0.77)
Burundi	Diet high in processed meat	3 (0 to 5)	0.11 (0.02 to 0.22)	4 (1 to 9)	0.1 (0.01 to 0.21)	-0.62 (-0.73 to -0.5)

Countries	Risk factors	1990 Number of deaths (95% UI)	1990 ASDR per 100,000 (95% UI)	2019 Number of deaths (95% UI)	2019 ASDR per 100,000 (95% UI)	1990-2019 EAPC of ASDR (95% CI)
Burundi	Diet low in whole grains	29 (11 to 41)	1.29 (0.47 to 1.82)	51 (18 to 81)	1.21 (0.44 to 1.9)	-0.38 (-0.46 to -0.31)
Cabo Verde	All dietary risks	4 (3 to 4)	1.59 (1.23 to 1.91)	14 (10 to 19)	3.46 (2.35 to 4.52)	1.98 (1.62 to 2.35)
Cabo Verde	Diet low in milk	2 (1 to 2)	0.64 (0.24 to 0.88)	7 (2 to 10)	1.61 (0.6 to 2.28)	2.56 (2.21 to 2.91)
Cabo Verde	Diet low in fiber	0 (0 to 0)	0.06 (0.02 to 0.15)	1 (0 to 1)	0.12 (0.04 to 0.3)	1.26 (0.7 to 1.81)
Cabo Verde	Diet low in calcium	2 (2 to 3)	0.91 (0.7 to 1.17)	7 (4 to 10)	1.65 (1.04 to 2.34)	1.21 (0.82 to 1.61)
Cabo Verde	Diet high in red meat	0 (0 to 1)	0.09 (0.01 to 0.24)	1 (0 to 3)	0.24 (0.03 to 0.65)	2.6 (2.21 to 2.99)
Cabo Verde	Diet high in processed meat	0 (0 to 0)	0.1 (0.01 to 0.17)	1 (0 to 2)	0.25 (0.03 to 0.46)	2.85 (2.57 to 3.14)
Cabo Verde	Diet low in whole grains	2 (1 to 2)	0.64 (0.24 to 0.88)	7 (2 to 10)	1.61 (0.6 to 2.28)	2.56 (2.21 to 2.91)
Cambodia	All dietary risks	173 (128 to 233)	4.05 (3.05 to 5.47)	649 (481 to 816)	5.86 (4.39 to 7.28)	1.29 (1.25 to 1.33)
Cambodia	Diet low in milk	45 (18 to 72)	1.06 (0.43 to 1.66)	170 (64 to 258)	1.52 (0.58 to 2.3)	1.28 (1.24 to 1.31)
Cambodia	Diet low in fiber	39 (21 to 59)	0.91 (0.5 to 1.38)	125 (66 to 182)	1.13 (0.61 to 1.65)	0.66 (0.62 to 0.7)
Cambodia	Diet low in calcium	111 (77 to 156)	2.59 (1.81 to 3.64)	418 (310 to 536)	3.78 (2.82 to 4.81)	1.32 (1.27 to 1.36)
Cambodia	Diet high in red meat	8 (1 to 22)	0.18 (0.02 to 0.5)	35 (3 to 97)	0.31 (0.03 to 0.84)	1.79 (1.76 to 1.82)
Cambodia	Diet high in processed meat	2 (1 to 3)	0.05 (0.03 to 0.08)	10 (5 to 16)	0.09 (0.04 to 0.14)	2.16 (2.08 to 2.24)
Cambodia	Diet low in whole grains	45 (18 to 72)	1.06 (0.43 to 1.66)	170 (64 to 258)	1.52 (0.58 to 2.3)	1.28 (1.24 to 1.31)
Cameroon	All dietary risks	144 (106 to 184)	3.66 (2.73 to 4.67)	410 (291 to 569)	3.97 (2.89 to 5.32)	0.27 (0.24 to 0.3)
Cameroon	Diet low in milk	55 (21 to 77)	1.39 (0.52 to 1.94)	158 (57 to 237)	1.52 (0.55 to 2.26)	0.34 (0.32 to 0.37)
Cameroon	Diet low in fiber	2 (1 to 4)	0.04 (0.02 to 0.11)	3 (2 to 6)	0.03 (0.02 to 0.06)	-1.77 (-2.05 to -1.49)

Countries	Risk factors	1990 Number of deaths (95% UI)	1990 ASDR per 100,000 (95% UI)	2019 Number of deaths (95% UI)	2019 ASDR per 100,000 (95% UI)	1990-2019 EAPC of ASDR (95% CI)
Cameroon	Diet low in calcium	92 (68 to 119)	2.35 (1.74 to 3.03)	253 (179 to 354)	2.47 (1.77 to 3.39)	0.13 (0.09 to 0.17)
Cameroon	Diet high in red meat	8 (1 to 23)	0.2 (0.02 to 0.57)	23 (2 to 64)	0.22 (0.02 to 0.59)	0.3 (0.25 to 0.36)
Cameroon	Diet high in processed meat	9 (1 to 17)	0.21 (0.02 to 0.4)	29 (4 to 56)	0.26 (0.03 to 0.5)	0.9 (0.82 to 0.97)
Cameroon	Diet low in whole grains	55 (21 to 77)	1.39 (0.52 to 1.94)	158 (57 to 237)	1.52 (0.55 to 2.26)	0.34 (0.32 to 0.37)
Canada	All dietary risks	2051 (1504 to 2552)	6.38 (4.68 to 7.93)	3525 (2515 to 4452)	4.94 (3.55 to 6.22)	-0.88 (-0.97 to -0.8)
Canada	Diet low in milk	976 (367 to 1291)	3.03 (1.14 to 4.01)	1763 (660 to 2370)	2.46 (0.92 to 3.31)	-0.73 (-0.79 to -0.67)
Canada	Diet low in fiber	144 (50 to 302)	0.45 (0.16 to 0.94)	149 (50 to 363)	0.2 (0.07 to 0.5)	-2.64 (-2.89 to -2.38)
Canada	Diet low in calcium	537 (285 to 906)	1.67 (0.89 to 2.81)	863 (420 to 1512)	1.2 (0.58 to 2.1)	-1.01 (-1.18 to -0.84)
Canada	Diet high in red meat	405 (97 to 767)	1.26 (0.3 to 2.38)	646 (130 to 1313)	0.92 (0.19 to 1.84)	-1.23 (-1.35 to -1.1)
Canada	Diet high in processed meat	288 (74 to 461)	0.9 (0.23 to 1.44)	563 (150 to 881)	0.8 (0.23 to 1.25)	-0.46 (-0.52 to -0.4)
Canada	Diet low in whole grains	976 (367 to 1291)	3.03 (1.14 to 4.01)	1763 (660 to 2370)	2.46 (0.92 to 3.31)	-0.73 (-0.79 to -0.67)
Central African Republic	All dietary risks	32 (22 to 42)	2.93 (2.18 to 3.85)	50 (34 to 73)	2.52 (1.78 to 3.57)	-0.44 (-0.5 to -0.39)
Central African Republic	Diet low in milk	13 (5 to 18)	1.16 (0.44 to 1.68)	20 (7 to 30)	0.99 (0.36 to 1.51)	-0.47 (-0.52 to -0.41)
Central African Republic	Diet low in fiber	1 (0 to 2)	0.09 (0.03 to 0.22)	1 (0 to 3)	0.07 (0.03 to 0.17)	-0.55 (-0.91 to -0.19)
Central African Republic	Diet low in calcium	19 (13 to 26)	1.79 (1.28 to 2.37)	30 (20 to 43)	1.51 (1.07 to 2.17)	-0.53 (-0.6 to -0.45)
Central African Republic	Diet high in red meat	3 (0 to 7)	0.23 (0.02 to 0.66)	5 (1 to 13)	0.24 (0.03 to 0.62)	0.36 (0.18 to 0.55)
Central African Republic	Diet high in processed meat	1 (0 to 2)	0.1 (0.02 to 0.2)	2 (0 to 4)	0.09 (0.01 to 0.18)	-0.3 (-0.39 to -0.2)
Central African Republic	Diet low in whole grains	13 (5 to 18)	1.16 (0.44 to 1.68)	20 (7 to 30)	0.99 (0.36 to 1.51)	-0.47 (-0.52 to -0.41)

Countries	Risk factors	1990 Number of deaths (95% UI)	1990 ASDR per 100,000 (95% UI)	2019 Number of deaths (95% UI)	2019 ASDR per 100,000 (95% UI)	1990-2019 EAPC of ASDR (95% CI)
Chad	All dietary risks	62 (46 to 80)	2.36 (1.76 to 3.03)	156 (115 to 210)	3.16 (2.34 to 4.2)	1.14 (1.08 to 1.2)
Chad	Diet low in milk	30 (15 to 41)	1.14 (0.57 to 1.53)	78 (38 to 109)	1.57 (0.78 to 2.18)	1.25 (1.18 to 1.32)
Chad	Diet low in fiber	2 (1 to 5)	0.07 (0.02 to 0.17)	2 (1 to 6)	0.05 (0.02 to 0.12)	-1.7 (-1.99 to -1.41)
Chad	Diet low in calcium	35 (26 to 46)	1.33 (0.98 to 1.76)	85 (61 to 117)	1.74 (1.28 to 2.33)	1.05 (0.99 to 1.1)
Chad	Diet high in red meat	4 (0 to 10)	0.13 (0.01 to 0.37)	9 (1 to 25)	0.17 (0.01 to 0.48)	1.02 (0.96 to 1.08)
Chad	Diet high in processed meat	3 (0 to 5)	0.1 (0.01 to 0.2)	8 (1 to 16)	0.16 (0.02 to 0.31)	1.67 (1.58 to 1.76)
Chad	Diet low in whole grains	30 (15 to 41)	1.14 (0.57 to 1.53)	78 (38 to 109)	1.57 (0.78 to 2.18)	1.25 (1.18 to 1.32)
Chile	All dietary risks	484 (379 to 565)	5.29 (4.15 to 6.16)	1404 (1087 to 1674)	5.86 (4.54 to 6.99)	0.69 (0.57 to 0.8)
Chile	Diet low in milk	220 (90 to 281)	2.39 (0.97 to 3.06)	597 (219 to 774)	2.49 (0.91 to 3.23)	0.4 (0.28 to 0.52)
Chile	Diet low in fiber	39 (14 to 72)	0.44 (0.16 to 0.79)	76 (25 to 165)	0.32 (0.11 to 0.69)	-0.83 (-0.97 to -0.7)
Chile	Diet low in calcium	215 (164 to 275)	2.37 (1.78 to 3.02)	557 (390 to 762)	2.33 (1.63 to 3.18)	0.39 (0.24 to 0.55)
Chile	Diet high in red meat	41 (4 to 104)	0.45 (0.05 to 1.12)	218 (54 to 417)	0.91 (0.22 to 1.74)	2.81 (2.67 to 2.96)
Chile	Diet high in processed meat	61 (14 to 93)	0.66 (0.15 to 1.01)	223 (72 to 337)	0.93 (0.3 to 1.41)	1.52 (1.42 to 1.61)
Chile	Diet low in whole grains	220 (90 to 281)	2.39 (0.97 to 3.06)	597 (219 to 774)	2.49 (0.91 to 3.23)	0.4 (0.28 to 0.52)
China	All dietary risks	29656 (23146 to 35344)	3.84 (3.01 to 4.57)	90407 (65691 to 114669)	4.76 (3.48 to 6.01)	1.11 (0.85 to 1.37)
China	Diet low in milk	12319 (4612 to 16523)	1.59 (0.6 to 2.13)	41733 (15441 to 57649)	2.2 (0.82 to 3.04)	1.45 (1.19 to 1.71)
China	Diet low in fiber	1914 (748 to 3591)	0.25 (0.1 to 0.46)	2548 (1057 to 5592)	0.14 (0.06 to 0.3)	-1.63 (-1.96 to -1.3)
China	Diet low in calcium	16556 (13032 to 21225)	2.16 (1.71 to 2.77)	40118 (27305 to 55646)	2.13 (1.45 to 2.94)	0.33 (0.03 to 0.63)

Countries	Risk factors	1990 Number of deaths (95% UI)	1990 ASDR per 100,000 (95% UI)	2019 Number of deaths (95% UI)	2019 ASDR per 100,000 (95% UI)	1990-2019 EAPC of ASDR (95% CI)
China	Diet high in red meat	2722 (392 to 6670)	0.34 (0.05 to 0.84)	15719 (3800 to 30286)	0.81 (0.2 to 1.57)	3.6 (3.33 to 3.86)
China	Diet high in processed meat	573 (264 to 906)	0.07 (0.03 to 0.11)	3151 (666 to 5697)	0.16 (0.04 to 0.29)	3.47 (3.17 to 3.76)
China	Diet low in whole grains	12319 (4612 to 16523)	1.59 (0.6 to 2.13)	41733 (15441 to 57649)	2.2 (0.82 to 3.04)	1.45 (1.19 to 1.71)
Colombia	All dietary risks	543 (417 to 644)	3.36 (2.6 to 3.97)	1643 (1082 to 2256)	3.08 (2.03 to 4.23)	-0.24 (-0.35 to -0.12)
Colombia	Diet low in milk	228 (85 to 304)	1.4 (0.53 to 1.87)	762 (279 to 1100)	1.43 (0.52 to 2.06)	0 (-0.1 to 0.1)
Colombia	Diet low in fiber	47 (17 to 90)	0.29 (0.11 to 0.55)	111 (37 to 244)	0.21 (0.07 to 0.46)	-0.67 (-0.97 to -0.38)
Colombia	Diet low in calcium	282 (213 to 361)	1.76 (1.32 to 2.25)	744 (464 to 1078)	1.39 (0.87 to 2.02)	-0.64 (-0.8 to -0.49)
Colombia	Diet high in red meat	48 (4 to 127)	0.29 (0.02 to 0.76)	165 (16 to 448)	0.31 (0.03 to 0.85)	0.22 (0.12 to 0.32)
Colombia	Diet high in processed meat	17 (3 to 29)	0.1 (0.02 to 0.17)	72 (11 to 141)	0.14 (0.02 to 0.26)	1.18 (1.06 to 1.31)
Colombia	Diet low in whole grains	228 (85 to 304)	1.4 (0.53 to 1.87)	762 (279 to 1100)	1.43 (0.52 to 2.06)	0 (-0.1 to 0.1)
Comoros	All dietary risks	7 (4 to 9)	3.16 (2.03 to 4.27)	16 (11 to 21)	3.5 (2.41 to 4.59)	0.24 (0.14 to 0.33)
Comoros	Diet low in milk	2 (1 to 4)	1.19 (0.44 to 1.79)	6 (2 to 9)	1.33 (0.46 to 1.92)	0.27 (0.17 to 0.36)
Comoros	Diet low in fiber	0 (0 to 0)	0.08 (0.03 to 0.23)	1 (0 to 1)	0.12 (0.04 to 0.28)	1.46 (1.33 to 1.58)
Comoros	Diet low in calcium	4 (3 to 6)	2.09 (1.36 to 2.9)	11 (7 to 14)	2.29 (1.55 to 3.08)	0.21 (0.11 to 0.31)
Comoros	Diet high in red meat	0 (0 to 1)	0.12 (0.02 to 0.33)	1 (0 to 1)	0.12 (0.02 to 0.31)	-0.03 (-0.17 to 0.1)
Comoros	Diet high in processed meat	0 (0 to 1)	0.13 (0.02 to 0.26)	1 (0 to 1)	0.14 (0.02 to 0.27)	0.16 (0.05 to 0.28)
Comoros	Diet low in whole grains	2 (1 to 4)	1.19 (0.44 to 1.79)	6 (2 to 9)	1.33 (0.46 to 1.92)	0.27 (0.17 to 0.36)
Congo	All dietary risks	48 (31 to 66)	4.88 (3.34 to 6.56)	109 (76 to 148)	4.7 (3.38 to 6.15)	-0.21 (-0.4 to -0.03)

Countries	Risk factors	1990 Number of deaths (95% UI)	1990 ASDR per 100,000 (95% UI)	2019 Number of deaths (95% UI)	2019 ASDR per 100,000 (95% UI)	1990-2019 EAPC of ASDR (95% CI)
Congo	Diet low in milk	17 (7 to 27)	1.75 (0.68 to 2.63)	40 (14 to 59)	1.7 (0.62 to 2.48)	-0.21 (-0.38 to -0.03)
Congo	Diet low in fiber	3 (1 to 6)	0.3 (0.1 to 0.65)	8 (3 to 15)	0.34 (0.12 to 0.67)	0.26 (0.13 to 0.39)
Congo	Diet low in calcium	31 (19 to 44)	3.18 (2.1 to 4.41)	69 (48 to 96)	2.99 (2.15 to 4.01)	-0.31 (-0.51 to -0.11)
Congo	Diet high in red meat	2 (0 to 7)	0.24 (0.02 to 0.68)	6 (0 to 18)	0.25 (0.02 to 0.71)	0.3 (0.09 to 0.52)
Congo	Diet high in processed meat	2 (0 to 4)	0.17 (0.03 to 0.35)	4 (1 to 9)	0.17 (0.02 to 0.33)	-0.05 (-0.23 to 0.13)
Congo	Diet low in whole grains	17 (7 to 27)	1.75 (0.68 to 2.63)	40 (14 to 59)	1.7 (0.62 to 2.48)	-0.21 (-0.38 to -0.03)
Cook Islands	All dietary risks	0 (0 to 0)	3.05 (2.29 to 3.8)	1 (0 to 1)	2.58 (1.8 to 3.37)	-0.66 (-0.87 to -0.45)
Cook Islands	Diet low in milk	0 (0 to 0)	1.37 (0.52 to 1.88)	0 (0 to 0)	1.25 (0.45 to 1.73)	-0.42 (-0.64 to -0.21)
Cook Islands	Diet low in fiber	0 (0 to 0)	0.02 (0.02 to 0.03)	0 (0 to 0)	0.02 (0.01 to 0.02)	-0.68 (-0.91 to -0.45)
Cook Islands	Diet low in calcium	0 (0 to 0)	1.73 (1.29 to 2.23)	0 (0 to 0)	1.29 (0.89 to 1.79)	-1.05 (-1.26 to -0.84)
Cook Islands	Diet high in red meat	0 (0 to 0)	0.26 (0.03 to 0.67)	0 (0 to 0)	0.27 (0.03 to 0.68)	-0.13 (-0.35 to 0.09)
Cook Islands	Diet high in processed meat	0 (0 to 0)	0.07 (0.02 to 0.14)	0 (0 to 0)	0.08 (0.02 to 0.14)	0.03 (-0.19 to 0.24)
Cook Islands	Diet low in whole grains	0 (0 to 0)	1.37 (0.52 to 1.88)	0 (0 to 0)	1.25 (0.45 to 1.73)	-0.42 (-0.64 to -0.21)
Costa Rica	All dietary risks	54 (39 to 66)	3.19 (2.3 to 3.94)	237 (151 to 323)	4.63 (2.96 to 6.31)	1.43 (1.3 to 1.55)
Costa Rica	Diet low in milk	26 (10 to 34)	1.56 (0.59 to 2.04)	128 (49 to 181)	2.51 (0.95 to 3.53)	1.76 (1.64 to 1.89)
Costa Rica	Diet low in fiber	6 (2 to 11)	0.36 (0.14 to 0.65)	26 (9 to 51)	0.51 (0.18 to 1)	1.38 (1.21 to 1.55)
Costa Rica	Diet low in calcium	22 (14 to 31)	1.29 (0.84 to 1.84)	77 (42 to 128)	1.52 (0.82 to 2.51)	0.74 (0.58 to 0.89)
Costa Rica	Diet high in red meat	5 (0 to 13)	0.29 (0.03 to 0.78)	23 (2 to 63)	0.44 (0.04 to 1.23)	1.48 (1.32 to 1.64)

Countries	Risk factors	1990		2019		1990-2019
		Number of deaths (95% UI)	ASDR per 100,000 (95% UI)	Number of deaths (95% UI)	ASDR per 100,000 (95% UI)	EAPC of ASDR (95% CI)
Costa Rica	Diet high in processed meat	2 (0 to 4)	0.13 (0.02 to 0.23)	11 (2 to 22)	0.21 (0.03 to 0.42)	1.97 (1.85 to 2.1)
Costa Rica	Diet low in whole grains	26 (10 to 34)	1.56 (0.59 to 2.04)	128 (49 to 181)	2.51 (0.95 to 3.53)	1.76 (1.64 to 1.89)
Côte d'Ivoire	All dietary risks	124 (89 to 167)	3.63 (2.69 to 4.82)	334 (242 to 437)	3.73 (2.79 to 4.74)	-0.14 (-0.25 to -0.04)
Côte d'Ivoire	Diet low in milk	49 (18 to 72)	1.44 (0.52 to 2.09)	134 (50 to 191)	1.49 (0.55 to 2.09)	-0.12 (-0.23 to -0.01)
Côte d'Ivoire	Diet low in fiber	1 (1 to 1)	0.02 (0.02 to 0.04)	2 (1 to 3)	0.02 (0.02 to 0.03)	-0.56 (-0.69 to -0.44)
Côte d'Ivoire	Diet low in calcium	77 (55 to 106)	2.28 (1.65 to 3.1)	204 (147 to 271)	2.3 (1.7 to 3.01)	-0.21 (-0.32 to -0.1)
Côte d'Ivoire	Diet high in red meat	7 (1 to 21)	0.21 (0.02 to 0.59)	19 (2 to 54)	0.21 (0.02 to 0.58)	-0.17 (-0.25 to -0.08)
Côte d'Ivoire	Diet high in processed meat	8 (1 to 15)	0.22 (0.03 to 0.42)	24 (3 to 44)	0.24 (0.03 to 0.45)	0.15 (0.06 to 0.25)
Côte d'Ivoire	Diet low in whole grains	49 (18 to 72)	1.44 (0.52 to 2.09)	134 (50 to 191)	1.49 (0.55 to 2.09)	-0.12 (-0.23 to -0.01)
Croatia	All dietary risks	406 (268 to 510)	6.67 (4.4 to 8.36)	647 (411 to 879)	7.06 (4.46 to 9.64)	0.44 (0.27 to 0.62)
Croatia	Diet low in milk	230 (85 to 299)	3.76 (1.39 to 4.88)	376 (137 to 526)	4.11 (1.5 to 5.74)	0.66 (0.47 to 0.85)
Croatia	Diet low in fiber	41 (14 to 78)	0.68 (0.23 to 1.29)	70 (24 to 140)	0.75 (0.25 to 1.51)	0.53 (0.34 to 0.72)
Croatia	Diet low in calcium	128 (70 to 207)	2.12 (1.16 to 3.41)	165 (76 to 289)	1.77 (0.81 to 3.11)	-0.8 (-0.99 to -0.61)
Croatia	Diet high in red meat	38 (3 to 103)	0.62 (0.05 to 1.68)	79 (8 to 211)	0.88 (0.1 to 2.34)	2.01 (1.65 to 2.37)
Croatia	Diet high in processed meat	15 (3 to 28)	0.25 (0.05 to 0.45)	26 (5 to 50)	0.29 (0.05 to 0.56)	0.99 (0.69 to 1.28)
Croatia	Diet low in whole grains	230 (85 to 299)	3.76 (1.39 to 4.88)	376 (137 to 526)	4.11 (1.5 to 5.74)	0.66 (0.47 to 0.85)
Cuba	All dietary risks	510 (367 to 622)	5.04 (3.61 to 6.15)	996 (663 to 1340)	5.09 (3.4 to 6.86)	-0.22 (-0.38 to -0.06)
Cuba	Diet low in milk	254 (95 to 329)	2.51 (0.94 to 3.25)	534 (194 to 741)	2.73 (0.99 to 3.81)	0.19 (0.04 to 0.34)

Countries	Risk factors	1990 Number of deaths (95% UI)	1990 ASDR per 100,000 (95% UI)	2019 Number of deaths (95% UI)	2019 ASDR per 100,000 (95% UI)	1990-2019 EAPC of ASDR (95% CI)
Cuba	Diet low in fiber	44 (15 to 88)	0.43 (0.15 to 0.86)	16 (8 to 34)	0.08 (0.04 to 0.17)	-6.9 (-7.85 to -5.93)
Cuba	Diet low in calcium	215 (142 to 305)	2.12 (1.4 to 3.02)	402 (237 to 624)	2.05 (1.2 to 3.19)	-0.75 (-0.98 to -0.52)
Cuba	Diet high in red meat	44 (4 to 121)	0.44 (0.04 to 1.19)	102 (9 to 276)	0.53 (0.04 to 1.43)	0.88 (0.64 to 1.12)
Cuba	Diet high in processed meat	19 (3 to 35)	0.19 (0.03 to 0.34)	47 (8 to 88)	0.24 (0.04 to 0.46)	1.01 (0.88 to 1.15)
Cuba	Diet low in whole grains	254 (95 to 329)	2.51 (0.94 to 3.25)	534 (194 to 741)	2.73 (0.99 to 3.81)	0.19 (0.04 to 0.34)
Cyprus	All dietary risks	33 (24 to 42)	4.63 (3.34 to 5.86)	91 (64 to 116)	4.94 (3.47 to 6.29)	0.49 (0.26 to 0.72)
Cyprus	Diet low in milk	17 (7 to 23)	2.38 (0.95 to 3.14)	48 (19 to 63)	2.58 (1.02 to 3.43)	0.51 (0.29 to 0.74)
Cyprus	Diet low in fiber	3 (1 to 6)	0.44 (0.16 to 0.83)	10 (4 to 17)	0.55 (0.21 to 0.95)	1.19 (0.9 to 1.48)
Cyprus	Diet low in calcium	9 (5 to 15)	1.3 (0.72 to 2.07)	25 (14 to 42)	1.38 (0.75 to 2.24)	0.6 (0.35 to 0.86)
Cyprus	Diet high in red meat	5 (1 to 10)	0.65 (0.12 to 1.42)	11 (2 to 26)	0.59 (0.09 to 1.39)	-0.22 (-0.5 to 0.06)
Cyprus	Diet high in processed meat	4 (1 to 7)	0.58 (0.12 to 0.94)	11 (2 to 18)	0.6 (0.12 to 0.99)	0.28 (0.08 to 0.47)
Cyprus	Diet low in whole grains	17 (7 to 23)	2.38 (0.95 to 3.14)	48 (19 to 63)	2.58 (1.02 to 3.43)	0.51 (0.29 to 0.74)
Czechia	All dietary risks	1364 (962 to 1733)	9.93 (7.01 to 12.63)	1334 (883 to 1812)	6.17 (4.1 to 8.38)	-1.77 (-1.95 to -1.59)
Czechia	Diet low in milk	648 (245 to 895)	4.71 (1.79 to 6.51)	667 (246 to 953)	3.08 (1.14 to 4.4)	-1.58 (-1.76 to -1.4)
Czechia	Diet low in fiber	109 (37 to 226)	0.8 (0.27 to 1.64)	114 (38 to 238)	0.52 (0.18 to 1.1)	-1.46 (-1.77 to -1.15)
Czechia	Diet low in calcium	327 (164 to 573)	2.37 (1.19 to 4.17)	298 (135 to 531)	1.36 (0.61 to 2.42)	-1.98 (-2.2 to -1.76)
Czechia	Diet high in red meat	275 (62 to 544)	2.01 (0.45 to 3.94)	230 (41 to 503)	1.09 (0.21 to 2.34)	-2.46 (-2.62 to -2.29)
Czechia	Diet high in processed meat	181 (32 to 298)	1.32 (0.24 to 2.15)	189 (37 to 323)	0.88 (0.18 to 1.5)	-1.49 (-1.68 to -1.31)

Countries	Risk factors	1990 Number of deaths (95% UI)	1990 ASDR per 100,000 (95% UI)	2019 Number of deaths (95% UI)	2019 ASDR per 100,000 (95% UI)	1990-2019 EAPC of ASDR (95% CI)
Czechia	Diet low in whole grains	648 (245 to 895)	4.71 (1.79 to 6.51)	667 (246 to 953)	3.08 (1.14 to 4.4)	-1.58 (-1.76 to -1.4)
Democratic People's Republic of Korea	All dietary risks	605 (435 to 818)	3.94 (2.9 to 5.18)	1354 (966 to 1786)	4.28 (3.01 to 5.61)	0.42 (0.35 to 0.48)
Democratic People's Republic of Korea	Diet low in milk	231 (84 to 346)	1.49 (0.55 to 2.21)	498 (180 to 730)	1.57 (0.57 to 2.29)	0.31 (0.25 to 0.38)
Democratic People's Republic of Korea	Diet low in fiber	27 (9 to 63)	0.19 (0.06 to 0.42)	85 (29 to 180)	0.27 (0.09 to 0.57)	1.51 (1.36 to 1.66)
Democratic People's Republic of Korea	Diet low in calcium	378 (273 to 513)	2.48 (1.83 to 3.3)	866 (610 to 1176)	2.74 (1.91 to 3.69)	0.47 (0.4 to 0.53)
Democratic People's Republic of Korea	Diet high in red meat	33 (3 to 89)	0.21 (0.02 to 0.56)	65 (7 to 181)	0.21 (0.02 to 0.57)	0.16 (0.04 to 0.29)
Democratic People's Republic of Korea	Diet high in processed meat	16 (3 to 31)	0.1 (0.02 to 0.19)	34 (7 to 66)	0.11 (0.02 to 0.2)	0.37 (0.28 to 0.46)
Democratic People's Republic of Korea	Diet low in whole grains	231 (84 to 346)	1.49 (0.55 to 2.21)	498 (180 to 730)	1.57 (0.57 to 2.29)	0.31 (0.25 to 0.38)
Democratic Republic of the Congo	All dietary risks	358 (266 to 480)	2.63 (1.93 to 3.67)	806 (528 to 1210)	2.53 (1.62 to 3.92)	-0.27 (-0.53 to -0.01)
Democratic Republic of the Congo	Diet low in milk	130 (50 to 200)	0.95 (0.36 to 1.48)	292 (99 to 480)	0.92 (0.3 to 1.56)	-0.3 (-0.56 to -0.03)
Democratic Republic of the Congo	Diet low in fiber	5 (2 to 13)	0.05 (0.02 to 0.11)	39 (12 to 88)	0.13 (0.04 to 0.29)	3.55 (2.91 to 4.18)
Democratic Republic of the Congo	Diet low in calcium	241 (174 to 333)	1.78 (1.25 to 2.55)	541 (340 to 827)	1.71 (1.06 to 2.68)	-0.31 (-0.57 to -0.04)
Democratic Republic of the Congo	Diet high in red meat	11 (2 to 26)	0.08 (0.01 to 0.19)	18 (4 to 46)	0.06 (0.01 to 0.14)	-1.24 (-1.77 to -0.71)

Countries	Risk factors	1990 Number of deaths (95% UI)	1990 ASDR per 100,000 (95% UI)	2019 Number of deaths (95% UI)	2019 ASDR per 100,000 (95% UI)	1990-2019 EAPC of ASDR (95% CI)
Democratic Republic of the Congo	Diet high in processed meat	16 (2 to 32)	0.11 (0.02 to 0.21)	29 (4 to 62)	0.09 (0.01 to 0.19)	-0.99 (-1.43 to -0.54)
Democratic Republic of the Congo	Diet low in whole grains	130 (50 to 200)	0.95 (0.36 to 1.48)	292 (99 to 480)	0.92 (0.3 to 1.56)	-0.3 (-0.56 to -0.03)
Denmark	All dietary risks	641 (467 to 801)	7.68 (5.61 to 9.57)	826 (579 to 1039)	6.8 (4.79 to 8.56)	-0.83 (-1.04 to -0.61)
Denmark	Diet low in milk	296 (113 to 396)	3.53 (1.35 to 4.72)	446 (168 to 583)	3.66 (1.38 to 4.77)	-0.19 (-0.46 to 0.07)
Denmark	Diet low in fiber	42 (14 to 91)	0.49 (0.17 to 1.07)	45 (15 to 105)	0.36 (0.12 to 0.84)	-1.47 (-1.65 to -1.29)
Denmark	Diet low in calcium	124 (60 to 217)	1.45 (0.7 to 2.52)	106 (49 to 183)	0.84 (0.38 to 1.44)	-2.4 (-2.63 to -2.17)
Denmark	Diet high in red meat	150 (42 to 265)	1.86 (0.53 to 3.22)	175 (44 to 328)	1.48 (0.38 to 2.72)	-1.38 (-1.61 to -1.15)
Denmark	Diet high in processed meat	119 (34 to 180)	1.44 (0.42 to 2.18)	162 (51 to 248)	1.34 (0.42 to 2.05)	-0.63 (-0.88 to -0.38)
Denmark	Diet low in whole grains	296 (113 to 396)	3.53 (1.35 to 4.72)	446 (168 to 583)	3.66 (1.38 to 4.77)	-0.19 (-0.46 to 0.07)
Djibouti	All dietary risks	5 (3 to 7)	3.9 (2.71 to 5.35)	23 (16 to 33)	4.41 (3.17 to 6)	0.37 (0.32 to 0.42)
Djibouti	Diet low in milk	2 (1 to 3)	1.54 (0.58 to 2.23)	10 (4 to 16)	1.91 (0.7 to 2.79)	0.76 (0.71 to 0.81)
Djibouti	Diet low in fiber	1 (0 to 1)	0.62 (0.28 to 1.03)	2 (1 to 4)	0.39 (0.13 to 0.71)	-2.14 (-2.32 to -1.96)
Djibouti	Diet low in calcium	3 (2 to 4)	2.14 (1.47 to 2.98)	12 (8 to 18)	2.37 (1.72 to 3.25)	0.27 (0.22 to 0.32)
Djibouti	Diet high in red meat	0 (0 to 1)	0.22 (0.02 to 0.62)	2 (0 to 5)	0.29 (0.02 to 0.84)	1.16 (1.08 to 1.24)
Djibouti	Diet high in processed meat	0 (0 to 0)	0.11 (0.02 to 0.24)	1 (0 to 2)	0.16 (0.02 to 0.32)	1.4 (1.31 to 1.48)
Djibouti	Diet low in whole grains	2 (1 to 3)	1.54 (0.58 to 2.23)	10 (4 to 16)	1.91 (0.7 to 2.79)	0.76 (0.71 to 0.81)
Dominica	All dietary risks	3 (2 to 3)	3.81 (2.58 to 4.8)	4 (3 to 5)	4.43 (2.8 to 5.91)	0.74 (0.67 to 0.82)

Countries	Risk factors	1990 Number of deaths (95% UI)	1990 ASDR per 100,000 (95% UI)	2019 Number of deaths (95% UI)	2019 ASDR per 100,000 (95% UI)	1990-2019 EAPC of ASDR (95% CI)
Dominica	Diet low in milk	2 (1 to 2)	2.54 (1.25 to 3.21)	3 (1 to 4)	3.17 (1.54 to 4.26)	0.97 (0.9 to 1.04)
Dominica	Diet low in fiber	0 (0 to 0)	0.08 (0.03 to 0.2)	0 (0 to 0)	0.06 (0.03 to 0.13)	-0.91 (-1.31 to -0.51)
Dominica	Diet low in calcium	1 (0 to 1)	1.13 (0.6 to 1.89)	1 (0 to 2)	1.02 (0.44 to 1.9)	-0.18 (-0.42 to 0.06)
Dominica	Diet high in red meat	0 (0 to 1)	0.31 (0.03 to 0.84)	0 (0 to 1)	0.4 (0.03 to 1.13)	1.06 (0.97 to 1.14)
Dominica	Diet high in processed meat	0 (0 to 0)	0.14 (0.03 to 0.26)	0 (0 to 0)	0.18 (0.03 to 0.35)	1.11 (1.03 to 1.19)
Dominica	Diet low in whole grains	2 (1 to 2)	2.54 (1.25 to 3.21)	3 (1 to 4)	3.17 (1.54 to 4.26)	0.97 (0.9 to 1.04)
Dominican Republic	All dietary risks	98 (77 to 118)	2.9 (2.28 to 3.5)	389 (263 to 531)	4.34 (2.94 to 5.92)	2.01 (1.83 to 2.19)
Dominican Republic	Diet low in milk	40 (15 to 54)	1.19 (0.45 to 1.58)	168 (62 to 247)	1.87 (0.69 to 2.76)	2.22 (2.01 to 2.43)
Dominican Republic	Diet low in fiber	9 (3 to 17)	0.28 (0.11 to 0.5)	27 (9 to 59)	0.31 (0.11 to 0.66)	0.67 (0.47 to 0.86)
Dominican Republic	Diet low in calcium	55 (42 to 69)	1.63 (1.25 to 2.07)	205 (134 to 288)	2.3 (1.51 to 3.2)	1.78 (1.6 to 1.97)
Dominican Republic	Diet high in red meat	6 (1 to 15)	0.16 (0.02 to 0.43)	28 (2 to 79)	0.31 (0.03 to 0.86)	2.93 (2.73 to 3.14)
Dominican Republic	Diet high in processed meat	3 (1 to 5)	0.08 (0.02 to 0.14)	13 (2 to 27)	0.15 (0.03 to 0.29)	2.9 (2.66 to 3.15)
Dominican Republic	Diet low in whole grains	40 (15 to 54)	1.19 (0.45 to 1.58)	168 (62 to 247)	1.87 (0.69 to 2.76)	2.22 (2.01 to 2.43)
Ecuador	All dietary risks	132 (101 to 156)	2.67 (2.06 to 3.18)	577 (415 to 771)	4.09 (2.97 to 5.43)	2.1 (1.77 to 2.43)
Ecuador	Diet low in milk	59 (22 to 77)	1.19 (0.45 to 1.55)	251 (89 to 359)	1.78 (0.64 to 2.54)	1.97 (1.66 to 2.28)
Ecuador	Diet low in fiber	14 (5 to 25)	0.28 (0.11 to 0.5)	75 (29 to 125)	0.53 (0.2 to 0.89)	2.9 (2.48 to 3.32)
Ecuador	Diet low in calcium	66 (49 to 85)	1.34 (1 to 1.73)	269 (182 to 376)	1.92 (1.32 to 2.67)	1.89 (1.55 to 2.24)
Ecuador	Diet high in red meat	9 (1 to 26)	0.19 (0.02 to 0.51)	54 (6 to 143)	0.38 (0.04 to 0.99)	3.06 (2.74 to 3.38)

Countries	Risk factors	1990 Number of deaths (95% UI)	1990 ASDR per 100,000 (95% UI)	2019 Number of deaths (95% UI)	2019 ASDR per 100,000 (95% UI)	1990-2019 EAPC of ASDR (95% CI)
Ecuador	Diet high in processed meat	4 (1 to 6)	0.07 (0.02 to 0.11)	17 (5 to 32)	0.12 (0.03 to 0.21)	2.5 (2.18 to 2.81)
Ecuador	Diet low in whole grains	59 (22 to 77)	1.19 (0.45 to 1.55)	251 (89 to 359)	1.78 (0.64 to 2.54)	1.97 (1.66 to 2.28)
Egypt	All dietary risks	551 (429 to 646)	1.97 (1.53 to 2.3)	1385 (870 to 2004)	2.29 (1.49 to 3.24)	0.52 (0.41 to 0.63)
Egypt	Diet low in milk	246 (91 to 320)	0.87 (0.32 to 1.14)	708 (248 to 1083)	1.16 (0.41 to 1.76)	1.03 (0.95 to 1.11)
Egypt	Diet low in fiber	5 (3 to 11)	0.02 (0.01 to 0.04)	12 (8 to 19)	0.02 (0.01 to 0.03)	-0.02 (-0.28 to 0.24)
Egypt	Diet low in calcium	323 (260 to 402)	1.16 (0.93 to 1.43)	643 (396 to 984)	1.09 (0.68 to 1.66)	-0.32 (-0.48 to -0.17)
Egypt	Diet high in red meat	33 (3 to 88)	0.11 (0.01 to 0.3)	112 (9 to 330)	0.18 (0.02 to 0.51)	1.64 (1.57 to 1.71)
Egypt	Diet high in processed meat	15 (3 to 26)	0.05 (0.01 to 0.09)	64 (9 to 134)	0.1 (0.02 to 0.21)	2.57 (2.47 to 2.68)
Egypt	Diet low in whole grains	246 (91 to 320)	0.87 (0.32 to 1.14)	708 (248 to 1083)	1.16 (0.41 to 1.76)	1.03 (0.95 to 1.11)
El Salvador	All dietary risks	49 (38 to 57)	1.67 (1.31 to 1.98)	165 (109 to 226)	2.7 (1.77 to 3.7)	1.47 (1.2 to 1.74)
El Salvador	Diet low in milk	20 (8 to 27)	0.7 (0.26 to 0.94)	80 (30 to 117)	1.32 (0.49 to 1.93)	2.09 (1.77 to 2.41)
El Salvador	Diet low in fiber	1 (0 to 2)	0.03 (0.01 to 0.07)	3 (1 to 7)	0.05 (0.02 to 0.11)	1.88 (1.69 to 2.07)
El Salvador	Diet low in calcium	30 (24 to 37)	1.02 (0.82 to 1.28)	83 (52 to 120)	1.34 (0.84 to 1.97)	0.68 (0.44 to 0.91)
El Salvador	Diet high in red meat	2 (0 to 5)	0.07 (0.01 to 0.17)	10 (1 to 26)	0.16 (0.02 to 0.43)	2.7 (2.26 to 3.15)
El Salvador	Diet high in processed meat	2 (0 to 3)	0.06 (0.01 to 0.1)	7 (1 to 14)	0.12 (0.02 to 0.24)	2.38 (2.03 to 2.73)
El Salvador	Diet low in whole grains	20 (8 to 27)	0.7 (0.26 to 0.94)	80 (30 to 117)	1.32 (0.49 to 1.93)	2.09 (1.77 to 2.41)
Equatorial Guinea	All dietary risks	5 (3 to 7)	2.7 (1.8 to 3.71)	21 (12 to 31)	4.93 (3.03 to 7.14)	2.62 (2.43 to 2.8)
Equatorial Guinea	Diet low in milk	2 (1 to 3)	1.01 (0.39 to 1.49)	8 (3 to 14)	2 (0.71 to 3.21)	3.05 (2.82 to 3.28)

Countries	Risk factors	1990 Number of deaths (95% UI)	1990 ASDR per 100,000 (95% UI)	2019 Number of deaths (95% UI)	2019 ASDR per 100,000 (95% UI)	1990-2019 EAPC of ASDR (95% CI)
Equatorial Guinea	Diet low in fiber	0 (0 to 1)	0.14 (0.04 to 0.32)	0 (0 to 1)	0.07 (0.03 to 0.16)	-3.81 (-4.43 to -3.18)
Equatorial Guinea	Diet low in calcium	3 (2 to 5)	1.74 (1.15 to 2.42)	12 (7 to 18)	2.85 (1.77 to 4.21)	2.03 (1.9 to 2.16)
Equatorial Guinea	Diet high in red meat	0 (0 to 1)	0.14 (0.01 to 0.41)	2 (0 to 5)	0.39 (0.03 to 1.13)	4.71 (4.27 to 5.14)
Equatorial Guinea	Diet high in processed meat	0 (0 to 0)	0.09 (0.01 to 0.18)	1 (0 to 3)	0.26 (0.03 to 0.55)	5 (4.51 to 5.48)
Equatorial Guinea	Diet low in whole grains	2 (1 to 3)	1.01 (0.39 to 1.49)	8 (3 to 14)	2 (0.71 to 3.21)	3.05 (2.82 to 3.28)
Eritrea	All dietary risks	24 (17 to 34)	2.59 (1.83 to 3.6)	95 (68 to 127)	3.85 (2.85 to 5.09)	1.12 (0.85 to 1.38)
Eritrea	Diet low in milk	9 (4 to 14)	0.98 (0.38 to 1.52)	37 (13 to 55)	1.49 (0.54 to 2.17)	1.19 (0.92 to 1.47)
Eritrea	Diet low in fiber	1 (0 to 2)	0.07 (0.02 to 0.17)	2 (1 to 4)	0.07 (0.03 to 0.17)	0.3 (0.26 to 0.35)
Eritrea	Diet low in calcium	16 (11 to 23)	1.7 (1.15 to 2.4)	61 (44 to 82)	2.48 (1.84 to 3.3)	1.06 (0.8 to 1.32)
Eritrea	Diet high in red meat	1 (0 to 3)	0.11 (0.01 to 0.31)	5 (0 to 12)	0.18 (0.02 to 0.48)	1.31 (0.99 to 1.64)
Eritrea	Diet high in processed meat	1 (0 to 2)	0.09 (0.01 to 0.19)	4 (1 to 8)	0.15 (0.02 to 0.3)	1.39 (1.06 to 1.72)
Eritrea	Diet low in whole grains	9 (4 to 14)	0.98 (0.38 to 1.52)	37 (13 to 55)	1.49 (0.54 to 2.17)	1.19 (0.92 to 1.47)
Estonia	All dietary risks	122 (84 to 152)	5.97 (4.11 to 7.47)	150 (96 to 205)	5.25 (3.41 to 7.15)	-0.44 (-0.64 to -0.24)
Estonia	Diet low in milk	71 (27 to 90)	3.45 (1.35 to 4.42)	84 (31 to 117)	2.95 (1.08 to 4.1)	-0.54 (-0.74 to -0.35)
Estonia	Diet low in fiber	4 (1 to 11)	0.22 (0.07 to 0.54)	4 (2 to 10)	0.14 (0.05 to 0.34)	-1.91 (-2.15 to -1.67)
Estonia	Diet low in calcium	16 (7 to 27)	0.78 (0.36 to 1.34)	18 (8 to 32)	0.62 (0.27 to 1.1)	-0.97 (-1.2 to -0.73)
Estonia	Diet high in red meat	15 (2 to 37)	0.75 (0.09 to 1.8)	19 (2 to 49)	0.7 (0.08 to 1.76)	-0.16 (-0.42 to 0.11)
Estonia	Diet high in processed meat	30 (11 to 51)	1.49 (0.56 to 2.53)	40 (15 to 68)	1.42 (0.53 to 2.46)	-0.09 (-0.31 to 0.14)

Countries	Risk factors	1990 Number of deaths (95% UI)	1990 ASDR per 100,000 (95% UI)	2019 Number of deaths (95% UI)	2019 ASDR per 100,000 (95% UI)	1990-2019 EAPC of ASDR (95% CI)
Estonia	Diet low in whole grains	71 (27 to 90)	3.45 (1.35 to 4.42)	84 (31 to 117)	2.95 (1.08 to 4.1)	-0.54 (-0.74 to -0.35)
Eswatini	All dietary risks	10 (7 to 13)	3.89 (2.81 to 5.16)	25 (16 to 35)	4.89 (3.17 to 6.8)	0.89 (0.57 to 1.22)
Eswatini	Diet low in milk	4 (1 to 6)	1.43 (0.54 to 2.18)	9 (3 to 14)	1.72 (0.62 to 2.7)	0.78 (0.49 to 1.07)
Eswatini	Diet low in fiber	0 (0 to 1)	0.11 (0.04 to 0.27)	1 (0 to 2)	0.18 (0.06 to 0.44)	2.09 (1.62 to 2.56)
Eswatini	Diet low in calcium	6 (4 to 8)	2.43 (1.73 to 3.27)	15 (10 to 22)	3.06 (2.02 to 4.26)	0.87 (0.54 to 1.21)
Eswatini	Diet high in red meat	1 (0 to 2)	0.34 (0.03 to 0.92)	2 (0 to 6)	0.41 (0.03 to 1.14)	0.67 (0.38 to 0.97)
Eswatini	Diet high in processed meat	0 (0 to 1)	0.12 (0.02 to 0.24)	1 (0 to 2)	0.16 (0.03 to 0.33)	1.17 (0.82 to 1.52)
Eswatini	Diet low in whole grains	4 (1 to 6)	1.43 (0.54 to 2.18)	9 (3 to 14)	1.72 (0.62 to 2.7)	0.78 (0.49 to 1.07)
Ethiopia	All dietary risks	583 (406 to 913)	3.07 (2.07 to 4.84)	1116 (774 to 1637)	2.89 (2.01 to 4.26)	-0.33 (-0.56 to -0.1)
Ethiopia	Diet low in milk	240 (97 to 394)	1.26 (0.52 to 2.07)	472 (175 to 738)	1.22 (0.45 to 1.94)	-0.22 (-0.45 to 0.01)
Ethiopia	Diet low in fiber	11 (4 to 28)	0.06 (0.02 to 0.15)	11 (6 to 23)	0.03 (0.02 to 0.06)	-3.14 (-3.4 to -2.89)
Ethiopia	Diet low in calcium	376 (254 to 597)	1.99 (1.29 to 3.18)	697 (478 to 1055)	1.82 (1.25 to 2.77)	-0.46 (-0.68 to -0.24)
Ethiopia	Diet high in red meat	23 (3 to 66)	0.12 (0.01 to 0.33)	50 (5 to 143)	0.13 (0.01 to 0.36)	0.29 (0 to 0.57)
Ethiopia	Diet high in processed meat	19 (3 to 40)	0.09 (0.02 to 0.2)	41 (7 to 87)	0.1 (0.02 to 0.21)	0.26 (-0.03 to 0.55)
Ethiopia	Diet low in whole grains	240 (97 to 394)	1.26 (0.52 to 2.07)	472 (175 to 738)	1.22 (0.45 to 1.94)	-0.22 (-0.45 to 0.01)
Fiji	All dietary risks	11 (8 to 15)	3.6 (2.71 to 4.53)	27 (20 to 36)	4.27 (3.09 to 5.57)	0.76 (0.55 to 0.96)
Fiji	Diet low in milk	5 (2 to 7)	1.55 (0.58 to 2.12)	12 (4 to 17)	1.91 (0.71 to 2.68)	0.87 (0.69 to 1.04)

Countries	Risk factors	1990 Number of deaths (95% UI)	1990 ASDR per 100,000 (95% UI)	2019 Number of deaths (95% UI)	2019 ASDR per 100,000 (95% UI)	1990-2019 EAPC of ASDR (95% CI)
Fiji	Diet low in fiber	0 (0 to 1)	0.14 (0.05 to 0.33)	0 (0 to 1)	0.07 (0.03 to 0.17)	-2.01 (-2.65 to -1.36)
Fiji	Diet low in calcium	6 (5 to 8)	2.03 (1.54 to 2.62)	15 (11 to 20)	2.37 (1.74 to 3.16)	0.79 (0.58 to 1.01)
Fiji	Diet high in red meat	1 (0 to 3)	0.36 (0.04 to 0.9)	3 (0 to 7)	0.4 (0.04 to 1.06)	0.2 (-0.01 to 0.41)
Fiji	Diet high in processed meat	0 (0 to 0)	0.08 (0.02 to 0.14)	1 (0 to 1)	0.1 (0.03 to 0.19)	1.16 (1 to 1.32)
Fiji	Diet low in whole grains	5 (2 to 7)	1.55 (0.58 to 2.12)	12 (4 to 17)	1.91 (0.71 to 2.68)	0.87 (0.69 to 1.04)
Finland	All dietary risks	315 (222 to 393)	4.39 (3.1 to 5.48)	466 (316 to 592)	3.56 (2.44 to 4.52)	-0.8 (-0.87 to -0.72)
Finland	Diet low in milk	169 (63 to 221)	2.35 (0.88 to 3.07)	260 (96 to 345)	1.98 (0.73 to 2.62)	-0.64 (-0.71 to -0.57)
Finland	Diet low in fiber	27 (10 to 56)	0.38 (0.13 to 0.77)	32 (11 to 67)	0.23 (0.08 to 0.49)	-2.14 (-2.29 to -1.99)
Finland	Diet low in calcium	39 (18 to 67)	0.54 (0.25 to 0.93)	53 (26 to 91)	0.38 (0.19 to 0.65)	-1.44 (-1.58 to -1.3)
Finland	Diet high in red meat	64 (15 to 125)	0.91 (0.22 to 1.74)	87 (18 to 178)	0.69 (0.15 to 1.41)	-0.93 (-1.22 to -0.63)
Finland	Diet high in processed meat	55 (14 to 85)	0.77 (0.2 to 1.19)	89 (26 to 138)	0.69 (0.2 to 1.06)	-0.37 (-0.51 to -0.23)
Finland	Diet low in whole grains	169 (63 to 221)	2.35 (0.88 to 3.07)	260 (96 to 345)	1.98 (0.73 to 2.62)	-0.64 (-0.71 to -0.57)
France	All dietary risks	7083 (5300 to 8567)	8.24 (6.17 to 9.94)	8886 (6399 to 10934)	5.76 (4.18 to 7.05)	-1.38 (-1.44 to -1.32)
France	Diet low in milk	3563 (1340 to 4615)	4.14 (1.55 to 5.35)	4591 (1795 to 6014)	2.97 (1.15 to 3.86)	-1.31 (-1.38 to -1.24)
France	Diet low in fiber	542 (196 to 1084)	0.62 (0.22 to 1.24)	682 (230 to 1384)	0.43 (0.15 to 0.87)	-1.31 (-1.36 to -1.26)
France	Diet low in calcium	912 (430 to 1590)	1.03 (0.48 to 1.79)	1344 (607 to 2431)	0.84 (0.38 to 1.51)	-0.63 (-0.72 to -0.55)
France	Diet high in red meat	1982 (719 to 3166)	2.35 (0.87 to 3.73)	2092 (651 to 3629)	1.41 (0.46 to 2.4)	-2 (-2.12 to -1.88)

Countries	Risk factors	1990 Number of deaths (95% UI)	1990 ASDR per 100,000 (95% UI)	2019 Number of deaths (95% UI)	2019 ASDR per 100,000 (95% UI)	1990-2019 EAPC of ASDR (95% CI)
France	Diet high in processed meat	1191 (358 to 1798)	1.4 (0.42 to 2.1)	1559 (482 to 2393)	1.02 (0.33 to 1.56)	-1.22 (-1.29 to -1.15)
France	Diet low in whole grains	3563 (1340 to 4615)	4.14 (1.55 to 5.35)	4591 (1795 to 6014)	2.97 (1.15 to 3.86)	-1.31 (-1.38 to -1.24)
Gabon	All dietary risks	31 (19 to 47)	5.87 (3.73 to 8.82)	54 (36 to 71)	5.65 (3.96 to 7.37)	-0.28 (-0.34 to -0.21)
Gabon	Diet low in milk	10 (4 to 18)	1.97 (0.71 to 3.32)	19 (7 to 29)	2.02 (0.74 to 3.02)	-0.05 (-0.13 to 0.03)
Gabon	Diet low in fiber	1 (0 to 2)	0.16 (0.05 to 0.44)	1 (0 to 3)	0.13 (0.05 to 0.32)	-0.89 (-1 to -0.79)
Gabon	Diet low in calcium	18 (12 to 28)	3.52 (2.27 to 5.37)	32 (22 to 43)	3.4 (2.37 to 4.47)	-0.26 (-0.32 to -0.2)
Gabon	Diet high in red meat	5 (1 to 11)	0.98 (0.24 to 2.12)	7 (1 to 15)	0.69 (0.11 to 1.59)	-1.49 (-1.73 to -1.24)
Gabon	Diet high in processed meat	1 (0 to 3)	0.23 (0.03 to 0.5)	2 (0 to 5)	0.25 (0.03 to 0.49)	0.19 (0.1 to 0.28)
Gabon	Diet low in whole grains	10 (4 to 18)	1.97 (0.71 to 3.32)	19 (7 to 29)	2.02 (0.74 to 3.02)	-0.05 (-0.13 to 0.03)
Gambia	All dietary risks	6 (5 to 8)	2 (1.5 to 2.58)	23 (16 to 31)	2.58 (1.78 to 3.53)	0.67 (0.53 to 0.82)
Gambia	Diet low in milk	2 (1 to 3)	0.69 (0.25 to 1)	9 (3 to 13)	0.97 (0.36 to 1.47)	0.98 (0.82 to 1.15)
Gambia	Diet low in fiber	1 (0 to 1)	0.2 (0.07 to 0.36)	1 (0 to 2)	0.09 (0.03 to 0.2)	-3.74 (-4.23 to -3.25)
Gambia	Diet low in calcium	4 (3 to 5)	1.26 (0.93 to 1.65)	14 (10 to 20)	1.64 (1.13 to 2.26)	0.7 (0.55 to 0.85)
Gambia	Diet high in red meat	0 (0 to 1)	0.07 (0.01 to 0.19)	1 (0 to 2)	0.09 (0.01 to 0.23)	0.55 (0.4 to 0.7)
Gambia	Diet high in processed meat	0 (0 to 1)	0.11 (0.01 to 0.21)	1 (0 to 3)	0.16 (0.02 to 0.32)	1.14 (0.98 to 1.3)
Gambia	Diet low in whole grains	2 (1 to 3)	0.69 (0.25 to 1)	9 (3 to 13)	0.97 (0.36 to 1.47)	0.98 (0.82 to 1.15)
Georgia	All dietary risks	229 (162 to 283)	3.77 (2.66 to 4.66)	264 (185 to 336)	4.43 (3.12 to 5.65)	1.46 (0.99 to 1.92)
Georgia	Diet low in milk	144 (71 to 178)	2.37 (1.17 to 2.93)	168 (82 to 220)	2.82 (1.37 to 3.68)	1.66 (1.12 to 2.21)

Countries	Risk factors	1990 Number of deaths (95% UI)	1990 ASDR per 100,000 (95% UI)	2019 Number of deaths (95% UI)	2019 ASDR per 100,000 (95% UI)	1990-2019 EAPC of ASDR (95% CI)
Georgia	Diet low in fiber	6 (2 to 16)	0.11 (0.04 to 0.28)	11 (4 to 26)	0.18 (0.06 to 0.43)	2.01 (1.48 to 2.54)
Georgia	Diet low in calcium	64 (34 to 106)	1.07 (0.58 to 1.76)	70 (37 to 117)	1.17 (0.61 to 1.95)	0.7 (0.39 to 1)
Georgia	Diet high in red meat	20 (2 to 53)	0.32 (0.03 to 0.87)	20 (2 to 56)	0.35 (0.03 to 0.94)	1.48 (0.81 to 2.16)
Georgia	Diet high in processed meat	24 (4 to 40)	0.39 (0.06 to 0.66)	28 (5 to 49)	0.47 (0.08 to 0.83)	1.84 (1.22 to 2.47)
Georgia	Diet low in whole grains	144 (71 to 178)	2.37 (1.17 to 2.93)	168 (82 to 220)	2.82 (1.37 to 3.68)	1.66 (1.12 to 2.21)
Germany	All dietary risks	10116 (7460 to 12662)	7.78 (5.73 to 9.72)	11624 (8408 to 14622)	5.59 (4.06 to 7)	-1.56 (-2.15 to -0.96)
Germany	Diet low in milk	4449 (1662 to 6079)	3.42 (1.28 to 4.66)	5750 (2126 to 7714)	2.76 (1.02 to 3.7)	-1.12 (-1.75 to -0.49)
Germany	Diet low in fiber	614 (209 to 1340)	0.46 (0.16 to 1.02)	742 (259 to 1592)	0.34 (0.12 to 0.74)	-1.75 (-2.48 to -1.01)
Germany	Diet low in calcium	2079 (1007 to 3719)	1.57 (0.75 to 2.8)	1783 (841 to 3113)	0.81 (0.38 to 1.41)	-3.02 (-3.71 to -2.34)
Germany	Diet high in red meat	2408 (709 to 4183)	1.89 (0.57 to 3.23)	2433 (588 to 4583)	1.23 (0.33 to 2.26)	-1.74 (-2.12 to -1.37)
Germany	Diet high in processed meat	2041 (654 to 3102)	1.57 (0.51 to 2.4)	2491 (844 to 3835)	1.21 (0.42 to 1.87)	-1.25 (-1.89 to -0.61)
Germany	Diet low in whole grains	4449 (1662 to 6079)	3.42 (1.28 to 4.66)	5750 (2126 to 7714)	2.76 (1.02 to 3.7)	-1.12 (-1.75 to -0.49)
Ghana	All dietary risks	140 (107 to 177)	2.54 (1.97 to 3.16)	450 (317 to 593)	3.15 (2.25 to 4.1)	0.72 (0.69 to 0.75)
Ghana	Diet low in milk	58 (21 to 80)	1.04 (0.38 to 1.44)	197 (71 to 281)	1.38 (0.5 to 1.95)	0.99 (0.96 to 1.01)
Ghana	Diet low in fiber	1 (1 to 2)	0.02 (0.01 to 0.04)	3 (2 to 3)	0.02 (0.01 to 0.02)	-0.1 (-0.4 to 0.2)
Ghana	Diet low in calcium	89 (68 to 114)	1.62 (1.25 to 2.08)	253 (179 to 341)	1.79 (1.28 to 2.41)	0.26 (0.22 to 0.3)
Ghana	Diet high in red meat	5 (1 to 13)	0.09 (0.01 to 0.23)	21 (2 to 55)	0.14 (0.02 to 0.37)	1.65 (1.54 to 1.75)
Ghana	Diet high in processed meat	8 (1 to 15)	0.14 (0.02 to 0.26)	37 (5 to 68)	0.25 (0.03 to 0.45)	2.1 (2.01 to 2.19)

Countries	Risk factors	1990 Number of deaths (95% UI)	1990 ASDR per 100,000 (95% UI)	2019 Number of deaths (95% UI)	2019 ASDR per 100,000 (95% UI)	1990-2019 EAPC of ASDR (95% CI)
Ghana	Diet low in whole grains	58 (21 to 80)	1.04 (0.38 to 1.44)	197 (71 to 281)	1.38 (0.5 to 1.95)	0.99 (0.96 to 1.01)
Greece	All dietary risks	711 (517 to 864)	4.74 (3.44 to 5.74)	1362 (983 to 1653)	5.1 (3.66 to 6.18)	-0.21 (-0.43 to 0)
Greece	Diet low in milk	457 (225 to 565)	3.04 (1.49 to 3.74)	837 (407 to 1046)	3.11 (1.51 to 3.91)	-0.45 (-0.68 to -0.22)
Greece	Diet low in fiber	17 (7 to 44)	0.12 (0.05 to 0.29)	53 (18 to 125)	0.19 (0.06 to 0.44)	1.25 (1.07 to 1.43)
Greece	Diet low in calcium	111 (52 to 198)	0.73 (0.34 to 1.3)	216 (107 to 381)	0.75 (0.36 to 1.31)	-0.33 (-0.57 to -0.08)
Greece	Diet high in red meat	132 (30 to 254)	0.89 (0.21 to 1.7)	278 (74 to 505)	1.11 (0.31 to 1.96)	0.43 (0.07 to 0.79)
Greece	Diet high in processed meat	82 (13 to 135)	0.55 (0.09 to 0.9)	159 (28 to 264)	0.63 (0.12 to 1.02)	0.15 (-0.01 to 0.3)
Greece	Diet low in whole grains	457 (225 to 565)	3.04 (1.49 to 3.74)	837 (407 to 1046)	3.11 (1.51 to 3.91)	-0.45 (-0.68 to -0.22)
Greenland	All dietary risks	3 (2 to 4)	9.6 (6.93 to 12)	6 (4 to 8)	9.78 (6.83 to 12.82)	-0.38 (-0.7 to -0.07)
Greenland	Diet low in milk	1 (1 to 2)	4.62 (1.73 to 6.19)	3 (1 to 4)	4.83 (1.81 to 6.6)	-0.29 (-0.6 to 0.02)
Greenland	Diet low in fiber	0 (0 to 0)	0.65 (0.23 to 1.28)	0 (0 to 1)	0.46 (0.16 to 1.02)	-1.84 (-2.25 to -1.43)
Greenland	Diet low in calcium	1 (0 to 1)	1.76 (0.87 to 3.06)	1 (0 to 1)	1.38 (0.65 to 2.37)	-1.44 (-1.86 to -1.03)
Greenland	Diet high in red meat	1 (0 to 1)	2.1 (0.61 to 3.89)	2 (0 to 3)	2.36 (0.69 to 4.36)	0.08 (-0.16 to 0.33)
Greenland	Diet high in processed meat	1 (0 to 1)	1.85 (0.59 to 2.83)	1 (0 to 2)	2.08 (0.7 to 3.32)	0 (-0.31 to 0.31)
Greenland	Diet low in whole grains	1 (1 to 2)	4.62 (1.73 to 6.19)	3 (1 to 4)	4.83 (1.81 to 6.6)	-0.29 (-0.6 to 0.02)
Grenada	All dietary risks	4 (3 to 5)	4.99 (3.65 to 6)	6 (4 to 8)	6.3 (4.39 to 7.72)	0.89 (0.77 to 1.01)
Grenada	Diet low in milk	2 (1 to 3)	2.79 (1.28 to 3.51)	4 (2 to 5)	3.61 (1.53 to 4.66)	0.96 (0.84 to 1.09)
Grenada	Diet low in fiber	0 (0 to 1)	0.3 (0.1 to 0.67)	0 (0 to 1)	0.32 (0.1 to 0.76)	0.18 (0.06 to 0.29)

Countries	Risk factors	1990 Number of deaths (95% UI)	1990 ASDR per 100,000 (95% UI)	2019 Number of deaths (95% UI)	2019 ASDR per 100,000 (95% UI)	1990-2019 EAPC of ASDR (95% CI)
Grenada	Diet low in calcium	2 (1 to 2)	2.19 (1.51 to 2.99)	3 (2 to 4)	2.54 (1.63 to 3.62)	0.63 (0.5 to 0.76)
Grenada	Diet high in red meat	0 (0 to 0)	0.22 (0.03 to 0.54)	0 (0 to 1)	0.37 (0.04 to 0.97)	1.97 (1.84 to 2.1)
Grenada	Diet high in processed meat	0 (0 to 0)	0.15 (0.03 to 0.27)	0 (0 to 0)	0.21 (0.04 to 0.37)	1.32 (1.17 to 1.47)
Grenada	Diet low in whole grains	2 (1 to 3)	2.79 (1.28 to 3.51)	4 (2 to 5)	3.61 (1.53 to 4.66)	0.96 (0.84 to 1.09)
Guam	All dietary risks	3 (2 to 4)	5.54 (3.77 to 7.22)	8 (6 to 11)	4.39 (3 to 5.72)	-0.82 (-1.2 to -0.44)
Guam	Diet low in milk	2 (1 to 2)	2.74 (1.04 to 3.73)	4 (2 to 6)	2.27 (0.83 to 3.13)	-0.64 (-1.02 to -0.25)
Guam	Diet low in fiber	0 (0 to 0)	0.04 (0.03 to 0.04)	0 (0 to 0)	0.03 (0.03 to 0.04)	-0.67 (-1.05 to -0.29)
Guam	Diet low in calcium	2 (1 to 2)	2.62 (1.7 to 3.71)	4 (2 to 5)	1.9 (1.21 to 2.75)	-1.2 (-1.57 to -0.83)
Guam	Diet high in red meat	0 (0 to 1)	0.65 (0.08 to 1.58)	1 (0 to 2)	0.56 (0.08 to 1.3)	-0.44 (-0.84 to -0.03)
Guam	Diet high in processed meat	0 (0 to 0)	0.17 (0.04 to 0.31)	0 (0 to 1)	0.15 (0.03 to 0.28)	-0.24 (-0.61 to 0.14)
Guam	Diet low in whole grains	2 (1 to 2)	2.74 (1.04 to 3.73)	4 (2 to 6)	2.27 (0.83 to 3.13)	-0.64 (-1.02 to -0.25)
Guatemala	All dietary risks	60 (48 to 72)	1.87 (1.49 to 2.2)	336 (242 to 435)	3.19 (2.33 to 4.09)	1.66 (1.32 to 2.01)
Guatemala	Diet low in milk	23 (8 to 32)	0.7 (0.26 to 0.98)	141 (52 to 203)	1.33 (0.49 to 1.92)	2.11 (1.74 to 2.48)
Guatemala	Diet low in fiber	1 (0 to 1)	0.02 (0.01 to 0.05)	5 (2 to 13)	0.05 (0.02 to 0.12)	2.26 (1.58 to 2.94)
Guatemala	Diet low in calcium	40 (32 to 49)	1.24 (0.99 to 1.52)	202 (145 to 269)	1.93 (1.39 to 2.55)	1.28 (0.95 to 1.61)
Guatemala	Diet high in red meat	2 (0 to 6)	0.07 (0.01 to 0.18)	16 (2 to 43)	0.15 (0.02 to 0.4)	2.47 (2.03 to 2.91)
Guatemala	Diet high in processed meat	2 (0 to 4)	0.06 (0.01 to 0.11)	12 (2 to 24)	0.11 (0.02 to 0.22)	2.3 (1.99 to 2.62)
Guatemala	Diet low in whole grains	23 (8 to 32)	0.7 (0.26 to 0.98)	141 (52 to 203)	1.33 (0.49 to 1.92)	2.11 (1.74 to 2.48)

Countries	Risk factors	1990 Number of deaths (95% UI)	1990 ASDR per 100,000 (95% UI)	2019 Number of deaths (95% UI)	2019 ASDR per 100,000 (95% UI)	1990-2019 EAPC of ASDR (95% CI)
Guinea	All dietary risks	70 (56 to 86)	2.3 (1.83 to 2.8)	143 (101 to 191)	2.83 (2.01 to 3.76)	0.79 (0.75 to 0.82)
Guinea	Diet low in milk	26 (10 to 36)	0.84 (0.32 to 1.18)	52 (18 to 78)	1.03 (0.37 to 1.51)	0.77 (0.74 to 0.81)
Guinea	Diet low in fiber	2 (1 to 5)	0.07 (0.02 to 0.16)	4 (1 to 9)	0.07 (0.02 to 0.18)	0.34 (0.15 to 0.54)
Guinea	Diet low in calcium	46 (36 to 59)	1.51 (1.17 to 1.94)	92 (65 to 125)	1.83 (1.29 to 2.47)	0.7 (0.66 to 0.74)
Guinea	Diet high in red meat	2 (0 to 5)	0.07 (0.01 to 0.17)	6 (1 to 16)	0.12 (0.01 to 0.31)	2.12 (2.01 to 2.24)
Guinea	Diet high in processed meat	4 (0 to 8)	0.13 (0.02 to 0.24)	9 (1 to 18)	0.17 (0.02 to 0.34)	1.1 (1.06 to 1.15)
Guinea	Diet low in whole grains	26 (10 to 36)	0.84 (0.32 to 1.18)	52 (18 to 78)	1.03 (0.37 to 1.51)	0.77 (0.74 to 0.81)
Guinea-Bissau	All dietary risks	14 (10 to 19)	3.73 (2.63 to 4.88)	24 (17 to 32)	3.76 (2.73 to 4.87)	0.04 (0 to 0.08)
Guinea-Bissau	Diet low in milk	4 (2 to 6)	1.14 (0.44 to 1.74)	8 (3 to 12)	1.24 (0.46 to 1.86)	0.38 (0.33 to 0.42)
Guinea-Bissau	Diet low in fiber	2 (1 to 3)	0.47 (0.2 to 0.78)	1 (0 to 3)	0.21 (0.07 to 0.43)	-3.38 (-3.61 to -3.14)
Guinea-Bissau	Diet low in calcium	9 (6 to 12)	2.4 (1.62 to 3.23)	16 (11 to 22)	2.48 (1.76 to 3.31)	0.15 (0.11 to 0.18)
Guinea-Bissau	Diet high in red meat	1 (0 to 2)	0.19 (0.02 to 0.56)	1 (0 to 4)	0.21 (0.02 to 0.59)	0.29 (0.22 to 0.37)
Guinea-Bissau	Diet high in processed meat	1 (0 to 1)	0.18 (0.02 to 0.36)	1 (0 to 3)	0.2 (0.02 to 0.39)	0.3 (0.25 to 0.34)
Guinea-Bissau	Diet low in whole grains	4 (2 to 6)	1.14 (0.44 to 1.74)	8 (3 to 12)	1.24 (0.46 to 1.86)	0.38 (0.33 to 0.42)
Guyana	All dietary risks	17 (13 to 21)	4.84 (3.83 to 5.82)	26 (18 to 35)	4.58 (3.16 to 6.03)	-0.28 (-0.42 to -0.14)
Guyana	Diet low in milk	7 (2 to 9)	1.87 (0.69 to 2.54)	13 (5 to 18)	2.2 (0.81 to 3.1)	0.48 (0.31 to 0.65)
Guyana	Diet low in fiber	2 (1 to 4)	0.58 (0.23 to 0.99)	2 (1 to 4)	0.35 (0.12 to 0.71)	-1.66 (-1.98 to -1.34)
Guyana	Diet low in calcium	10 (8 to 13)	2.82 (2.2 to 3.54)	12 (8 to 18)	2.21 (1.45 to 3.16)	-0.97 (-1.14 to -0.8)

Countries	Risk factors	1990 Number of deaths (95% UI)	1990 ASDR per 100,000 (95% UI)	2019 Number of deaths (95% UI)	2019 ASDR per 100,000 (95% UI)	1990-2019 EAPC of ASDR (95% CI)
Guyana	Diet high in red meat	1 (0 to 1)	0.16 (0.03 to 0.38)	1 (0 to 3)	0.19 (0.03 to 0.46)	0.29 (0.1 to 0.49)
Guyana	Diet high in processed meat	1 (0 to 1)	0.14 (0.03 to 0.26)	1 (0 to 2)	0.18 (0.03 to 0.35)	0.8 (0.62 to 0.99)
Guyana	Diet low in whole grains	7 (2 to 9)	1.87 (0.69 to 2.54)	13 (5 to 18)	2.2 (0.81 to 3.1)	0.48 (0.31 to 0.65)
Haiti	All dietary risks	122 (80 to 157)	4.11 (2.73 to 5.23)	270 (179 to 376)	4.27 (2.88 to 5.84)	0.26 (0.2 to 0.33)
Haiti	Diet low in milk	50 (20 to 70)	1.67 (0.67 to 2.34)	107 (38 to 163)	1.69 (0.6 to 2.54)	0.2 (0.1 to 0.29)
Haiti	Diet low in fiber	4 (1 to 10)	0.15 (0.05 to 0.36)	14 (4 to 28)	0.22 (0.07 to 0.45)	0.97 (0.67 to 1.26)
Haiti	Diet low in calcium	78 (50 to 104)	2.63 (1.71 to 3.48)	170 (114 to 239)	2.7 (1.81 to 3.78)	0.21 (0.15 to 0.28)
Haiti	Diet high in red meat	5 (1 to 14)	0.16 (0.02 to 0.44)	12 (1 to 33)	0.19 (0.02 to 0.51)	0.61 (0.5 to 0.73)
Haiti	Diet high in processed meat	3 (1 to 6)	0.09 (0.02 to 0.18)	7 (1 to 14)	0.1 (0.02 to 0.21)	0.6 (0.49 to 0.71)
Haiti	Diet low in whole grains	50 (20 to 70)	1.67 (0.67 to 2.34)	107 (38 to 163)	1.69 (0.6 to 2.54)	0.2 (0.1 to 0.29)
Honduras	All dietary risks	33 (25 to 41)	1.66 (1.23 to 2.06)	135 (85 to 202)	2.43 (1.55 to 3.64)	1.37 (1.24 to 1.51)
Honduras	Diet low in milk	13 (5 to 19)	0.66 (0.25 to 0.94)	61 (22 to 99)	1.1 (0.39 to 1.74)	1.83 (1.69 to 1.97)
Honduras	Diet low in fiber	1 (0 to 2)	0.04 (0.01 to 0.09)	4 (1 to 10)	0.07 (0.02 to 0.18)	2.25 (1.93 to 2.58)
Honduras	Diet low in calcium	20 (15 to 26)	1.03 (0.76 to 1.32)	71 (43 to 111)	1.3 (0.81 to 1.99)	0.82 (0.66 to 0.99)
Honduras	Diet high in red meat	2 (0 to 5)	0.09 (0.01 to 0.23)	9 (1 to 25)	0.15 (0.02 to 0.43)	1.95 (1.81 to 2.09)
Honduras	Diet high in processed meat	1 (0 to 2)	0.06 (0.01 to 0.1)	6 (1 to 12)	0.1 (0.02 to 0.21)	2.11 (1.97 to 2.24)
Honduras	Diet low in whole grains	13 (5 to 19)	0.66 (0.25 to 0.94)	61 (22 to 99)	1.1 (0.39 to 1.74)	1.83 (1.69 to 1.97)

Countries	Risk factors	1990		2019		1990-2019
		Number of deaths (95% UI)	ASDR per 100,000 (95% UI)	Number of deaths (95% UI)	ASDR per 100,000 (95% UI)	EAPC of ASDR (95% CI)
Hungary	All dietary risks	1386 (972 to 1730)	9.65 (6.76 to 12.02)	1846 (1251 to 2435)	9.35 (6.29 to 12.34)	-0.29 (-0.5 to -0.08)
Hungary	Diet low in milk	720 (269 to 935)	5 (1.86 to 6.52)	942 (346 to 1297)	4.76 (1.76 to 6.57)	-0.35 (-0.53 to -0.17)
Hungary	Diet low in fiber	84 (28 to 187)	0.59 (0.2 to 1.31)	124 (41 to 269)	0.62 (0.2 to 1.36)	-0.24 (-0.64 to 0.17)
Hungary	Diet low in calcium	409 (227 to 673)	2.85 (1.58 to 4.67)	548 (295 to 885)	2.74 (1.47 to 4.46)	-0.5 (-0.85 to -0.15)
Hungary	Diet high in red meat	190 (30 to 435)	1.32 (0.21 to 3.02)	231 (30 to 543)	1.2 (0.17 to 2.78)	-0.25 (-0.35 to -0.15)
Hungary	Diet high in processed meat	166 (26 to 268)	1.16 (0.19 to 1.88)	261 (56 to 435)	1.35 (0.3 to 2.24)	0.7 (0.54 to 0.87)
Hungary	Diet low in whole grains	720 (269 to 935)	5 (1.86 to 6.52)	942 (346 to 1297)	4.76 (1.76 to 6.57)	-0.35 (-0.53 to -0.17)
Iceland	All dietary risks	15 (11 to 18)	5.2 (3.89 to 6.28)	23 (16 to 29)	3.88 (2.72 to 4.87)	-1.22 (-1.39 to -1.04)
Iceland	Diet low in milk	8 (3 to 10)	2.64 (1.02 to 3.38)	12 (4 to 16)	1.97 (0.73 to 2.61)	-1.22 (-1.38 to -1.06)
Iceland	Diet low in fiber	2 (1 to 3)	0.61 (0.24 to 1.04)	3 (1 to 5)	0.43 (0.16 to 0.77)	-1.47 (-1.76 to -1.19)
Iceland	Diet low in calcium	1 (1 to 2)	0.47 (0.21 to 0.81)	2 (1 to 4)	0.35 (0.17 to 0.58)	-1.56 (-1.99 to -1.14)
Iceland	Diet high in red meat	4 (2 to 7)	1.46 (0.53 to 2.29)	6 (2 to 10)	1.02 (0.33 to 1.69)	-1.35 (-1.45 to -1.24)
Iceland	Diet high in processed meat	2 (1 to 4)	0.85 (0.26 to 1.31)	4 (1 to 6)	0.67 (0.19 to 1.05)	-0.95 (-1.08 to -0.82)
Iceland	Diet low in whole grains	8 (3 to 10)	2.64 (1.02 to 3.38)	12 (4 to 16)	1.97 (0.73 to 2.61)	-1.22 (-1.38 to -1.06)
India	All dietary risks	7349 (5771 to 8966)	1.91 (1.51 to 2.31)	24864 (18529 to 31324)	2.41 (1.81 to 3.02)	0.67 (0.55 to 0.8)
India	Diet low in milk	3116 (1186 to 4259)	0.79 (0.3 to 1.08)	11220 (4244 to 15602)	1.07 (0.4 to 1.48)	0.9 (0.76 to 1.04)
India	Diet low in fiber	573 (212 to 1129)	0.15 (0.06 to 0.29)	1356 (499 to 2983)	0.13 (0.05 to 0.29)	-0.62 (-0.9 to -0.33)

Countries	Risk factors	1990 Number of deaths (95% UI)	1990 ASDR per 100,000 (95% UI)	2019 Number of deaths (95% UI)	2019 ASDR per 100,000 (95% UI)	1990-2019 EAPC of ASDR (95% CI)
India	Diet low in calcium	4194 (3254 to 5326)	1.12 (0.88 to 1.41)	13505 (9962 to 17712)	1.33 (0.99 to 1.74)	0.51 (0.38 to 0.64)
India	Diet high in red meat	182 (58 to 375)	0.05 (0.01 to 0.1)	673 (191 to 1464)	0.06 (0.02 to 0.14)	0.96 (0.78 to 1.14)
India	Diet high in processed meat	196 (135 to 304)	0.05 (0.03 to 0.07)	957 (635 to 1398)	0.09 (0.06 to 0.13)	2.16 (2.04 to 2.28)
India	Diet low in whole grains	3116 (1186 to 4259)	0.79 (0.3 to 1.08)	11220 (4244 to 15602)	1.07 (0.4 to 1.48)	0.9 (0.76 to 1.04)
Indonesia	All dietary risks	3347 (2432 to 4228)	3.66 (2.7 to 4.57)	11599 (7639 to 15679)	6 (3.9 to 8.09)	1.72 (1.61 to 1.83)
Indonesia	Diet low in milk	1003 (389 to 1535)	1.09 (0.42 to 1.64)	3710 (1425 to 6007)	1.9 (0.73 to 3.08)	1.95 (1.82 to 2.08)
Indonesia	Diet low in fiber	429 (185 to 703)	0.47 (0.2 to 0.77)	1054 (417 to 1995)	0.56 (0.22 to 1.05)	0.59 (0.44 to 0.74)
Indonesia	Diet low in calcium	2274 (1621 to 2962)	2.49 (1.78 to 3.22)	7835 (5026 to 10740)	4.07 (2.57 to 5.58)	1.69 (1.58 to 1.8)
Indonesia	Diet high in red meat	98 (17 to 244)	0.1 (0.02 to 0.25)	396 (59 to 1039)	0.2 (0.03 to 0.52)	2.15 (2.06 to 2.24)
Indonesia	Diet high in processed meat	34 (24 to 44)	0.04 (0.03 to 0.05)	156 (87 to 238)	0.08 (0.05 to 0.12)	2.78 (2.72 to 2.83)
Indonesia	Diet low in whole grains	1003 (389 to 1535)	1.09 (0.42 to 1.64)	3710 (1425 to 6007)	1.9 (0.73 to 3.08)	1.95 (1.82 to 2.08)
Iran（Islamic Republic of)	All dietary risks	658 (503 to 826)	2.89 (2.19 to 3.63)	2408 (1816 to 2840)	3.51 (2.65 to 4.14)	0.9 (0.72 to 1.08)
Iran（Islamic Republic of)	Diet low in milk	347 (171 to 462)	1.51 (0.75 to 2.01)	1366 (672 to 1711)	1.98 (0.98 to 2.49)	1.14 (0.99 to 1.29)
Iran（Islamic Republic of)	Diet low in fiber	21 (8 to 49)	0.1 (0.04 to 0.22)	47 (21 to 102)	0.07 (0.03 to 0.15)	-0.78 (-1.17 to -0.39)
Iran（Islamic Republic of)	Diet low in calcium	336 (247 to 445)	1.49 (1.09 to 1.98)	1125 (825 to 1472)	1.65 (1.21 to 2.16)	0.61 (0.41 to 0.82)

Countries	Risk factors	1990 Number of deaths (95% UI)	1990 ASDR per 100,000 (95% UI)	2019 Number of deaths (95% UI)	2019 ASDR per 100,000 (95% UI)	1990-2019 EAPC of ASDR (95% CI)
Iran（Islamic Republic of)	Diet high in red meat	40 (4 to 110)	0.17 (0.02 to 0.46)	136 (14 to 356)	0.2 (0.02 to 0.5)	0.63 (0.43 to 0.84)
Iran（Islamic Republic of)	Diet high in processed meat	16 (5 to 30)	0.07 (0.02 to 0.12)	65 (18 to 112)	0.09 (0.03 to 0.16)	1.26 (1.13 to 1.4)
Iran（Islamic Republic of)	Diet low in whole grains	347 (171 to 462)	1.51 (0.75 to 2.01)	1366 (672 to 1711)	1.98 (0.98 to 2.49)	1.14 (0.99 to 1.29)
Iraq	All dietary risks	209 (144 to 292)	2.81 (1.95 to 3.88)	710 (518 to 915)	3.32 (2.45 to 4.18)	0.7 (0.56 to 0.83)
Iraq	Diet low in milk	104 (45 to 151)	1.4 (0.6 to 2.04)	333 (135 to 474)	1.55 (0.64 to 2.17)	0.56 (0.35 to 0.77)
Iraq	Diet low in fiber	4 (1 to 9)	0.05 (0.02 to 0.13)	29 (9 to 67)	0.14 (0.04 to 0.31)	3.13 (2.48 to 3.79)
Iraq	Diet low in calcium	117 (79 to 167)	1.58 (1.08 to 2.25)	412 (296 to 549)	1.94 (1.41 to 2.52)	0.71 (0.63 to 0.78)
Iraq	Diet high in red meat	9 (1 to 25)	0.12 (0.01 to 0.33)	24 (4 to 57)	0.11 (0.02 to 0.26)	0.17 (-0.4 to 0.74)
Iraq	Diet high in processed meat	5 (1 to 10)	0.06 (0.02 to 0.13)	16 (4 to 29)	0.07 (0.02 to 0.13)	0.79 (0.45 to 1.13)
Iraq	Diet low in whole grains	104 (45 to 151)	1.4 (0.6 to 2.04)	333 (135 to 474)	1.55 (0.64 to 2.17)	0.56 (0.35 to 0.77)
Ireland	All dietary risks	312 (225 to 390)	7.73 (5.62 to 9.68)	416 (295 to 528)	5.45 (3.88 to 6.91)	-1.26 (-1.3 to -1.21)
Ireland	Diet low in milk	151 (56 to 203)	3.74 (1.4 to 5.05)	191 (72 to 267)	2.5 (0.94 to 3.49)	-1.45 (-1.57 to -1.33)
Ireland	Diet low in fiber	14 (5 to 33)	0.34 (0.12 to 0.83)	17 (6 to 41)	0.22 (0.08 to 0.53)	-1.06 (-1.23 to -0.9)
Ireland	Diet low in calcium	45 (21 to 80)	1.11 (0.52 to 1.96)	41 (18 to 70)	0.53 (0.23 to 0.9)	-2.24 (-2.42 to -2.06)
Ireland	Diet high in red meat	92 (31 to 152)	2.3 (0.77 to 3.77)	146 (60 to 229)	1.93 (0.8 to 3)	-0.73 (-0.83 to -0.62)
Ireland	Diet high in processed meat	50 (11 to 79)	1.25 (0.28 to 1.96)	74 (19 to 115)	0.97 (0.25 to 1.5)	-0.93 (-1.07 to -0.79)

Countries	Risk factors	1990 Number of deaths (95% UI)	1990 ASDR per 100,000 (95% UI)	2019 Number of deaths (95% UI)	2019 ASDR per 100,000 (95% UI)	1990-2019 EAPC of ASDR (95% CI)
Ireland	Diet low in whole grains	151 (56 to 203)	3.74 (1.4 to 5.05)	191 (72 to 267)	2.5 (0.94 to 3.49)	-1.45 (-1.57 to -1.33)
Israel	All dietary risks	309 (205 to 386)	6.57 (4.38 to 8.23)	587 (388 to 734)	4.84 (3.2 to 6.05)	-1.82 (-2.1 to -1.53)
Israel	Diet low in milk	205 (91 to 258)	4.33 (1.93 to 5.48)	388 (167 to 496)	3.2 (1.38 to 4.08)	-1.84 (-2.15 to -1.53)
Israel	Diet low in fiber	7 (3 to 16)	0.15 (0.06 to 0.36)	13 (6 to 32)	0.1 (0.05 to 0.26)	-1.7 (-1.84 to -1.55)
Israel	Diet low in calcium	68 (33 to 120)	1.43 (0.7 to 2.52)	99 (45 to 174)	0.8 (0.37 to 1.42)	-2.61 (-2.8 to -2.43)
Israel	Diet high in red meat	30 (2 to 80)	0.64 (0.05 to 1.72)	73 (9 to 181)	0.61 (0.08 to 1.5)	-0.97 (-1.34 to -0.6)
Israel	Diet high in processed meat	32 (4 to 55)	0.7 (0.09 to 1.19)	73 (10 to 120)	0.61 (0.09 to 1)	-1.18 (-1.45 to -0.91)
Israel	Diet low in whole grains	205 (91 to 258)	4.33 (1.93 to 5.48)	388 (167 to 496)	3.2 (1.38 to 4.08)	-1.84 (-2.15 to -1.53)
Italy	All dietary risks	6314 (4725 to 7510)	7.08 (5.3 to 8.45)	8868 (6524 to 10794)	5.65 (4.13 to 6.84)	-0.95 (-1.04 to -0.86)
Italy	Diet low in milk	3730 (1827 to 4619)	4.18 (2.04 to 5.18)	5328 (2609 to 6695)	3.38 (1.65 to 4.23)	-0.9 (-1.02 to -0.79)
Italy	Diet low in fiber	290 (105 to 625)	0.33 (0.12 to 0.7)	450 (170 to 945)	0.28 (0.11 to 0.58)	-0.58 (-0.78 to -0.39)
Italy	Diet low in calcium	1140 (581 to 1973)	1.26 (0.64 to 2.19)	1504 (749 to 2610)	0.92 (0.46 to 1.59)	-1.18 (-1.28 to -1.08)
Italy	Diet high in red meat	1199 (340 to 2142)	1.36 (0.39 to 2.41)	1522 (404 to 2885)	1.01 (0.28 to 1.87)	-1.33 (-1.48 to -1.18)
Italy	Diet high in processed meat	911 (268 to 1389)	1.03 (0.3 to 1.57)	1355 (425 to 2090)	0.89 (0.29 to 1.35)	-0.63 (-0.77 to -0.5)
Italy	Diet low in whole grains	3730 (1827 to 4619)	4.18 (2.04 to 5.18)	5328 (2609 to 6695)	3.38 (1.65 to 4.23)	-0.9 (-1.02 to -0.79)
Jamaica	All dietary risks	63 (45 to 76)	3.45 (2.47 to 4.2)	156 (105 to 205)	5.14 (3.46 to 6.77)	1.59 (1.31 to 1.87)
Jamaica	Diet low in milk	32 (12 to 42)	1.79 (0.69 to 2.31)	85 (32 to 119)	2.8 (1.07 to 3.93)	1.68 (1.38 to 1.98)
Jamaica	Diet low in fiber	2 (1 to 5)	0.12 (0.04 to 0.29)	6 (2 to 14)	0.2 (0.07 to 0.47)	2.17 (1.91 to 2.44)

Countries	Risk factors	1990 Number of deaths (95% UI)	1990 ASDR per 100,000 (95% UI)	2019 Number of deaths (95% UI)	2019 ASDR per 100,000 (95% UI)	1990-2019 EAPC of ASDR (95% CI)
Jamaica	Diet low in calcium	29 (20 to 40)	1.59 (1.09 to 2.17)	67 (41 to 99)	2.19 (1.35 to 3.24)	1.46 (1.15 to 1.77)
Jamaica	Diet high in red meat	4 (0 to 11)	0.23 (0.02 to 0.62)	10 (1 to 28)	0.34 (0.03 to 0.93)	1.2 (0.87 to 1.54)
Jamaica	Diet high in processed meat	2 (0 to 4)	0.12 (0.02 to 0.23)	6 (1 to 12)	0.2 (0.03 to 0.39)	1.71 (1.39 to 2.04)
Jamaica	Diet low in whole grains	32 (12 to 42)	1.79 (0.69 to 2.31)	85 (32 to 119)	2.8 (1.07 to 3.93)	1.68 (1.38 to 1.98)
Japan	All dietary risks	9693 (7087 to 11881)	5.91 (4.32 to 7.24)	20829 (14658 to 26068)	5.24 (3.8 to 6.46)	-0.4 (-0.46 to -0.34)
Japan	Diet low in milk	4172 (1565 to 5755)	2.55 (0.96 to 3.52)	9488 (3559 to 12779)	2.38 (0.89 to 3.17)	-0.29 (-0.35 to -0.23)
Japan	Diet low in fiber	521 (198 to 1095)	0.33 (0.12 to 0.68)	1296 (517 to 2630)	0.32 (0.12 to 0.64)	0.15 (0.02 to 0.28)
Japan	Diet low in calcium	4101 (2661 to 5820)	2.51 (1.63 to 3.57)	8636 (5464 to 12519)	2.11 (1.32 to 3.06)	-0.42 (-0.52 to -0.33)
Japan	Diet high in red meat	805 (77 to 2136)	0.49 (0.05 to 1.3)	1639 (164 to 4326)	0.43 (0.04 to 1.11)	-0.77 (-0.86 to -0.67)
Japan	Diet high in processed meat	1396 (374 to 2174)	0.82 (0.22 to 1.28)	2639 (705 to 4168)	0.76 (0.22 to 1.18)	-0.39 (-0.6 to -0.18)
Japan	Diet low in whole grains	4172 (1565 to 5755)	2.55 (0.96 to 3.52)	9488 (3559 to 12779)	2.38 (0.89 to 3.17)	-0.29 (-0.35 to -0.23)
Jordan	All dietary risks	60 (44 to 77)	4.92 (3.55 to 6.27)	277 (197 to 354)	4.86 (3.48 to 6.12)	0.02 (-0.07 to 0.1)
Jordan	Diet low in milk	32 (16 to 44)	2.65 (1.32 to 3.57)	161 (78 to 211)	2.79 (1.37 to 3.66)	0.35 (0.25 to 0.45)
Jordan	Diet low in fiber	3 (1 to 7)	0.29 (0.1 to 0.61)	13 (4 to 29)	0.23 (0.08 to 0.5)	-1.2 (-1.34 to -1.06)
Jordan	Diet low in calcium	28 (20 to 38)	2.35 (1.69 to 3.18)	117 (78 to 166)	2.09 (1.41 to 2.96)	-0.52 (-0.6 to -0.43)
Jordan	Diet high in red meat	4 (0 to 10)	0.28 (0.03 to 0.77)	17 (2 to 47)	0.29 (0.03 to 0.8)	0.48 (0.31 to 0.64)
Jordan	Diet high in processed meat	1 (0 to 3)	0.11 (0.03 to 0.2)	8 (2 to 14)	0.12 (0.03 to 0.23)	0.72 (0.58 to 0.85)
Jordan	Diet low in whole grains	32 (16 to 44)	2.65 (1.32 to 3.57)	161 (78 to 211)	2.79 (1.37 to 3.66)	0.35 (0.25 to 0.45)

Countries	Risk factors	1990 Number of deaths (95% UI)	1990 ASDR per 100,000 (95% UI)	2019 Number of deaths (95% UI)	2019 ASDR per 100,000 (95% UI)	1990-2019 EAPC of ASDR (95% CI)
Kazakhstan	All dietary risks	693 (519 to 832)	5.57 (4.16 to 6.72)	747 (534 to 947)	4.59 (3.28 to 5.8)	-0.5 (-0.63 to -0.37)
Kazakhstan	Diet low in milk	401 (198 to 497)	3.24 (1.6 to 4)	471 (239 to 609)	2.9 (1.47 to 3.73)	-0.14 (-0.29 to 0.01)
Kazakhstan	Diet low in fiber	39 (13 to 87)	0.32 (0.11 to 0.7)	31 (10 to 73)	0.2 (0.07 to 0.47)	-2.15 (-2.74 to -1.55)
Kazakhstan	Diet low in calcium	131 (61 to 237)	1.07 (0.5 to 1.93)	78 (38 to 134)	0.51 (0.25 to 0.88)	-2.95 (-3.37 to -2.53)
Kazakhstan	Diet high in red meat	152 (45 to 259)	1.18 (0.34 to 2.05)	171 (50 to 299)	1.02 (0.29 to 1.81)	0.11 (-0.47 to 0.7)
Kazakhstan	Diet high in processed meat	79 (15 to 128)	0.62 (0.12 to 1.02)	91 (15 to 152)	0.55 (0.09 to 0.91)	-0.17 (-0.39 to 0.06)
Kazakhstan	Diet low in whole grains	401 (198 to 497)	3.24 (1.6 to 4)	471 (239 to 609)	2.9 (1.47 to 3.73)	-0.14 (-0.29 to 0.01)
Kenya	All dietary risks	127 (82 to 171)	1.69 (1.1 to 2.28)	483 (323 to 659)	2.45 (1.67 to 3.28)	1.55 (1.39 to 1.71)
Kenya	Diet low in milk	55 (22 to 82)	0.72 (0.29 to 1.08)	237 (90 to 342)	1.19 (0.45 to 1.72)	2.05 (1.86 to 2.24)
Kenya	Diet low in fiber	3 (1 to 7)	0.04 (0.02 to 0.1)	9 (4 to 19)	0.05 (0.02 to 0.1)	0.52 (0.39 to 0.65)
Kenya	Diet low in calcium	70 (42 to 98)	0.94 (0.56 to 1.31)	224 (142 to 338)	1.17 (0.76 to 1.73)	0.92 (0.79 to 1.04)
Kenya	Diet high in red meat	9 (1 to 26)	0.12 (0.01 to 0.33)	39 (4 to 107)	0.19 (0.02 to 0.52)	2.08 (1.87 to 2.3)
Kenya	Diet high in processed meat	6 (1 to 12)	0.08 (0.01 to 0.15)	28 (5 to 53)	0.13 (0.02 to 0.25)	2.28 (2.07 to 2.49)
Kenya	Diet low in whole grains	55 (22 to 82)	0.72 (0.29 to 1.08)	237 (90 to 342)	1.19 (0.45 to 1.72)	2.05 (1.86 to 2.24)
Kiribati	All dietary risks	2 (1 to 2)	4.56 (3.49 to 5.69)	3 (2 to 4)	4.42 (3.21 to 5.91)	-0.29 (-0.36 to -0.23)
Kiribati	Diet low in milk	1 (0 to 1)	1.84 (0.7 to 2.56)	1 (0 to 2)	1.8 (0.63 to 2.59)	-0.26 (-0.32 to -0.2)
Kiribati	Diet low in fiber	0 (0 to 0)	0.1 (0.04 to 0.26)	0 (0 to 0)	0.08 (0.03 to 0.21)	-1.13 (-1.3 to -0.97)
Kiribati	Diet low in calcium	1 (1 to 1)	2.98 (2.27 to 3.86)	2 (1 to 2)	2.86 (2.04 to 3.88)	-0.33 (-0.39 to -0.26)

Countries	Risk factors	1990 Number of deaths (95% UI)	1990 ASDR per 100,000 (95% UI)	2019 Number of deaths (95% UI)	2019 ASDR per 100,000 (95% UI)	1990-2019 EAPC of ASDR (95% CI)
Kiribati	Diet high in red meat	0 (0 to 0)	0.23 (0.02 to 0.64)	0 (0 to 0)	0.23 (0.02 to 0.63)	-0.21 (-0.28 to -0.13)
Kiribati	Diet high in processed meat	0 (0 to 0)	0.08 (0.03 to 0.15)	0 (0 to 0)	0.09 (0.03 to 0.17)	0.11 (0.06 to 0.16)
Kiribati	Diet low in whole grains	1 (0 to 1)	1.84 (0.7 to 2.56)	1 (0 to 2)	1.8 (0.63 to 2.59)	-0.26 (-0.32 to -0.2)
Kuwait	All dietary risks	12 (8 to 15)	2.19 (1.49 to 2.81)	62 (40 to 83)	2.82 (1.81 to 3.78)	1.68 (1.28 to 2.07)
Kuwait	Diet low in milk	7 (2 to 9)	1.25 (0.46 to 1.66)	37 (14 to 51)	1.7 (0.63 to 2.35)	1.88 (1.42 to 2.34)
Kuwait	Diet low in fiber	1 (0 to 2)	0.14 (0.05 to 0.32)	3 (1 to 7)	0.13 (0.04 to 0.31)	0.42 (0.04 to 0.81)
Kuwait	Diet low in calcium	4 (2 to 6)	0.69 (0.38 to 1.12)	17 (8 to 30)	0.8 (0.4 to 1.4)	1.18 (0.84 to 1.51)
Kuwait	Diet high in red meat	2 (0 to 4)	0.28 (0.03 to 0.68)	9 (1 to 21)	0.37 (0.04 to 0.94)	1.75 (1.15 to 2.36)
Kuwait	Diet high in processed meat	0 (0 to 1)	0.07 (0.02 to 0.12)	2 (1 to 5)	0.11 (0.02 to 0.2)	2.31 (1.71 to 2.9)
Kuwait	Diet low in whole grains	7 (2 to 9)	1.25 (0.46 to 1.66)	37 (14 to 51)	1.7 (0.63 to 2.35)	1.88 (1.42 to 2.34)
Kyrgyzstan	All dietary risks	120 (87 to 147)	3.97 (2.9 to 4.89)	113 (79 to 143)	2.7 (1.9 to 3.42)	-1.5 (-1.7 to -1.29)
Kyrgyzstan	Diet low in milk	72 (36 to 89)	2.4 (1.19 to 2.96)	74 (37 to 95)	1.76 (0.89 to 2.28)	-1.14 (-1.35 to -0.92)
Kyrgyzstan	Diet low in fiber	6 (2 to 15)	0.21 (0.07 to 0.5)	4 (1 to 10)	0.11 (0.04 to 0.24)	-3.22 (-3.5 to -2.94)
Kyrgyzstan	Diet low in calcium	24 (11 to 44)	0.79 (0.37 to 1.46)	21 (10 to 37)	0.52 (0.25 to 0.91)	-1.97 (-2.19 to -1.76)
Kyrgyzstan	Diet high in red meat	21 (5 to 41)	0.7 (0.16 to 1.35)	15 (2 to 33)	0.34 (0.05 to 0.78)	-2.69 (-3.18 to -2.2)
Kyrgyzstan	Diet high in processed meat	13 (2 to 22)	0.43 (0.07 to 0.71)	13 (2 to 22)	0.3 (0.04 to 0.51)	-1.32 (-1.62 to -1.01)
Kyrgyzstan	Diet low in whole grains	72 (36 to 89)	2.4 (1.19 to 2.96)	74 (37 to 95)	1.76 (0.89 to 2.28)	-1.14 (-1.35 to -0.92)

Countries	Risk factors	1990 Number of deaths (95% UI)	1990 ASDR per 100,000 (95% UI)	2019 Number of deaths (95% UI)	2019 ASDR per 100,000 (95% UI)	1990-2019 EAPC of ASDR (95% CI)
Lao People's Democratic Republic	All dietary risks	90 (59 to 125)	4.48 (3.06 to 6.17)	220 (153 to 300)	5.41 (3.75 to 7.23)	0.46 (0.39 to 0.53)
Lao People's Democratic Republic	Diet low in milk	24 (10 to 40)	1.21 (0.47 to 1.99)	61 (24 to 99)	1.5 (0.58 to 2.43)	0.58 (0.51 to 0.65)
Lao People's Democratic Republic	Diet low in fiber	18 (9 to 29)	0.9 (0.46 to 1.42)	32 (15 to 53)	0.81 (0.38 to 1.3)	-0.71 (-0.82 to -0.6)
Lao People's Democratic Republic	Diet low in calcium	58 (37 to 83)	2.91 (1.92 to 4.15)	143 (98 to 195)	3.54 (2.42 to 4.77)	0.49 (0.42 to 0.56)
Lao People's Democratic Republic	Diet high in red meat	4 (0 to 12)	0.19 (0.02 to 0.56)	14 (1 to 40)	0.33 (0.03 to 0.94)	1.75 (1.7 to 1.8)
Lao People's Democratic Republic	Diet high in processed meat	1 (1 to 2)	0.06 (0.03 to 0.09)	4 (2 to 6)	0.08 (0.04 to 0.14)	1.33 (1.27 to 1.39)
Lao People's Democratic Republic	Diet low in whole grains	24 (10 to 40)	1.21 (0.47 to 1.99)	61 (24 to 99)	1.5 (0.58 to 2.43)	0.58 (0.51 to 0.65)
Latvia	All dietary risks	220 (159 to 272)	6.16 (4.45 to 7.6)	230 (154 to 304)	5.42 (3.61 to 7.19)	-0.53 (-0.78 to -0.28)
Latvia	Diet low in milk	131 (60 to 164)	3.66 (1.69 to 4.57)	128 (47 to 174)	3.01 (1.1 to 4.12)	-0.77 (-1 to -0.53)
Latvia	Diet low in fiber	11 (4 to 26)	0.31 (0.1 to 0.71)	13 (4 to 28)	0.3 (0.1 to 0.65)	-0.55 (-1.02 to -0.07)
Latvia	Diet low in calcium	27 (13 to 46)	0.75 (0.35 to 1.29)	31 (13 to 55)	0.7 (0.3 to 1.24)	-0.78 (-1.36 to -0.19)
Latvia	Diet high in red meat	29 (5 to 65)	0.81 (0.13 to 1.82)	27 (3 to 68)	0.67 (0.08 to 1.62)	-0.44 (-0.81 to -0.07)
Latvia	Diet high in processed meat	53 (19 to 95)	1.48 (0.54 to 2.64)	60 (23 to 105)	1.43 (0.54 to 2.58)	-0.11 (-0.39 to 0.17)
Latvia	Diet low in whole grains	131 (60 to 164)	3.66 (1.69 to 4.57)	128 (47 to 174)	3.01 (1.1 to 4.12)	-0.77 (-1 to -0.53)
Lebanon	All dietary risks	86 (57 to 115)	4.26 (2.84 to 5.68)	285 (185 to 390)	5.5 (3.57 to 7.55)	1.46 (1.25 to 1.66)

Countries	Risk factors	1990 Number of deaths (95% UI)	1990 ASDR per 100,000 (95% UI)	2019 Number of deaths (95% UI)	2019 ASDR per 100,000 (95% UI)	1990-2019 EAPC of ASDR (95% CI)
Lebanon	Diet low in milk	58 (29 to 80)	2.85 (1.43 to 3.89)	194 (90 to 272)	3.73 (1.73 to 5.25)	1.45 (1.27 to 1.64)
Lebanon	Diet low in fiber	1 (1 to 2)	0.05 (0.03 to 0.12)	5 (2 to 12)	0.09 (0.04 to 0.22)	2.84 (2.43 to 3.24)
Lebanon	Diet low in calcium	26 (14 to 45)	1.33 (0.72 to 2.25)	81 (42 to 140)	1.57 (0.82 to 2.71)	1.33 (1.02 to 1.64)
Lebanon	Diet high in red meat	7 (1 to 20)	0.35 (0.03 to 0.99)	26 (2 to 71)	0.5 (0.04 to 1.37)	1.8 (1.59 to 2)
Lebanon	Diet high in processed meat	3 (1 to 5)	0.12 (0.03 to 0.23)	9 (2 to 16)	0.16 (0.04 to 0.31)	1.46 (1.29 to 1.62)
Lebanon	Diet low in whole grains	58 (29 to 80)	2.85 (1.43 to 3.89)	194 (90 to 272)	3.73 (1.73 to 5.25)	1.45 (1.27 to 1.64)
Lesotho	All dietary risks	22 (16 to 33)	2.53 (1.85 to 3.75)	50 (35 to 69)	4.43 (3.12 to 6.06)	2.34 (2.16 to 2.51)
Lesotho	Diet low in milk	7 (3 to 12)	0.82 (0.31 to 1.36)	16 (6 to 25)	1.38 (0.49 to 2.2)	2.14 (2 to 2.3)
Lesotho	Diet low in fiber	0 (0 to 0)	0.02 (0.01 to 0.03)	0 (0 to 0)	0.03 (0.02 to 0.04)	1.79 (1.65 to 1.92)
Lesotho	Diet low in calcium	16 (11 to 23)	1.77 (1.25 to 2.6)	35 (24 to 48)	3.12 (2.15 to 4.23)	2.36 (2.18 to 2.55)
Lesotho	Diet high in red meat	1 (0 to 4)	0.17 (0.01 to 0.48)	4 (0 to 10)	0.3 (0.02 to 0.87)	2.54 (2.38 to 2.71)
Lesotho	Diet high in processed meat	1 (0 to 1)	0.08 (0.01 to 0.16)	2 (0 to 4)	0.16 (0.02 to 0.32)	2.93 (2.7 to 3.15)
Lesotho	Diet low in whole grains	7 (3 to 12)	0.82 (0.31 to 1.36)	16 (6 to 25)	1.38 (0.49 to 2.2)	2.14 (2 to 2.3)
Liberia	All dietary risks	29 (22 to 36)	2.8 (2.18 to 3.48)	51 (34 to 74)	2.86 (1.89 to 4.2)	0.5 (0.2 to 0.81)
Liberia	Diet low in milk	10 (4 to 14)	0.98 (0.38 to 1.39)	18 (7 to 29)	1 (0.37 to 1.6)	0.5 (0.21 to 0.8)
Liberia	Diet low in fiber	2 (1 to 4)	0.19 (0.06 to 0.36)	5 (2 to 9)	0.26 (0.09 to 0.49)	1.08 (0.91 to 1.26)
Liberia	Diet low in calcium	18 (14 to 24)	1.81 (1.38 to 2.29)	33 (21 to 49)	1.83 (1.16 to 2.74)	0.48 (0.17 to 0.79)
Liberia	Diet high in red meat	1 (0 to 3)	0.1 (0.01 to 0.24)	2 (0 to 4)	0.08 (0.01 to 0.23)	0.29 (-0.23 to 0.8)

Countries	Risk factors	1990		2019		1990-2019
		Number of deaths (95% UI)	ASDR per 100,000 (95% UI)	Number of deaths (95% UI)	ASDR per 100,000 (95% UI)	EAPC of ASDR (95% CI)
Liberia	Diet high in processed meat	2 (0 to 3)	0.16 (0.02 to 0.3)	3 (0 to 6)	0.15 (0.02 to 0.31)	0.48 (0.04 to 0.93)
Liberia	Diet low in whole grains	10 (4 to 14)	0.98 (0.38 to 1.39)	18 (7 to 29)	1 (0.37 to 1.6)	0.5 (0.21 to 0.8)
Libya	All dietary risks	72 (46 to 106)	4.01 (2.59 to 5.88)	212 (134 to 294)	4.28 (2.73 to 5.86)	0.3 (0.19 to 0.42)
Libya	Diet low in milk	44 (20 to 66)	2.42 (1.13 to 3.65)	123 (54 to 180)	2.48 (1.07 to 3.53)	0.3 (0.16 to 0.44)
Libya	Diet low in fiber	2 (1 to 4)	0.09 (0.03 to 0.25)	8 (3 to 19)	0.17 (0.05 to 0.38)	1.53 (1.24 to 1.83)
Libya	Diet low in calcium	27 (15 to 45)	1.51 (0.85 to 2.53)	86 (52 to 131)	1.76 (1.08 to 2.65)	0.3 (0.13 to 0.48)
Libya	Diet high in red meat	6 (0 to 18)	0.34 (0.02 to 0.99)	15 (1 to 41)	0.29 (0.03 to 0.81)	-0.26 (-0.46 to -0.06)
Libya	Diet high in processed meat	2 (0 to 5)	0.12 (0.03 to 0.24)	6 (1 to 12)	0.12 (0.03 to 0.23)	0.28 (0.07 to 0.49)
Libya	Diet low in whole grains	44 (20 to 66)	2.42 (1.13 to 3.65)	123 (54 to 180)	2.48 (1.07 to 3.53)	0.3 (0.16 to 0.44)
Lithuania	All dietary risks	265 (191 to 324)	5.91 (4.25 to 7.21)	322 (209 to 421)	5.32 (3.45 to 7)	-0.36 (-0.56 to -0.16)
Lithuania	Diet low in milk	143 (60 to 180)	3.18 (1.34 to 4.02)	168 (62 to 228)	2.76 (1.01 to 3.76)	-0.5 (-0.67 to -0.33)
Lithuania	Diet low in fiber	3 (2 to 8)	0.08 (0.04 to 0.18)	7 (3 to 18)	0.12 (0.05 to 0.29)	1.8 (1.21 to 2.39)
Lithuania	Diet low in calcium	56 (28 to 99)	1.24 (0.62 to 2.21)	52 (23 to 92)	0.82 (0.37 to 1.45)	-1.68 (-1.97 to -1.38)
Lithuania	Diet high in red meat	42 (9 to 85)	0.94 (0.2 to 1.9)	53 (10 to 115)	0.91 (0.18 to 1.92)	0.24 (-0.06 to 0.54)
Lithuania	Diet high in processed meat	62 (23 to 112)	1.38 (0.51 to 2.49)	84 (29 to 150)	1.41 (0.48 to 2.53)	0.13 (-0.1 to 0.37)
Lithuania	Diet low in whole grains	143 (60 to 180)	3.18 (1.34 to 4.02)	168 (62 to 228)	2.76 (1.01 to 3.76)	-0.5 (-0.67 to -0.33)
Luxembourg	All dietary risks	48 (34 to 59)	8.74 (6.4 to 10.76)	54 (38 to 69)	5.07 (3.62 to 6.47)	-2.06 (-2.26 to -1.86)
Luxembourg	Diet low in milk	23 (9 to 30)	4.26 (1.59 to 5.58)	27 (10 to 36)	2.49 (0.93 to 3.38)	-2 (-2.15 to -1.86)

Countries	Risk factors	1990		2019		1990-2019
		Number of deaths (95% UI)	ASDR per 100,000 (95% UI)	Number of deaths (95% UI)	ASDR per 100,000 (95% UI)	EAPC of ASDR (95% CI)
Luxembourg	Diet low in fiber	4 (1 to 7)	0.67 (0.23 to 1.36)	4 (1 to 8)	0.32 (0.11 to 0.69)	-2.52 (-2.8 to -2.25)
Luxembourg	Diet low in calcium	6 (3 to 11)	1.11 (0.51 to 1.92)	6 (3 to 9)	0.51 (0.25 to 0.85)	-2.69 (-2.92 to -2.47)
Luxembourg	Diet high in red meat	12 (3 to 20)	2.14 (0.62 to 3.65)	14 (4 to 24)	1.36 (0.43 to 2.34)	-1.94 (-2.22 to -1.65)
Luxembourg	Diet high in processed meat	10 (4 to 15)	1.82 (0.66 to 2.83)	11 (4 to 18)	1.09 (0.38 to 1.73)	-1.94 (-2.17 to -1.71)
Luxembourg	Diet low in whole grains	23 (9 to 30)	4.26 (1.59 to 5.58)	27 (10 to 36)	2.49 (0.93 to 3.38)	-2 (-2.15 to -1.86)
Madagascar	All dietary risks	123 (93 to 155)	2.57 (1.97 to 3.21)	273 (187 to 383)	2.74 (1.9 to 3.79)	0.12 (0.04 to 0.2)
Madagascar	Diet low in milk	44 (17 to 64)	0.92 (0.34 to 1.32)	94 (34 to 152)	0.94 (0.36 to 1.49)	-0.01 (-0.09 to 0.07)
Madagascar	Diet low in fiber	7 (2 to 15)	0.15 (0.05 to 0.32)	15 (5 to 34)	0.16 (0.05 to 0.35)	-0.07 (-0.29 to 0.15)
Madagascar	Diet low in calcium	76 (58 to 97)	1.59 (1.22 to 2.02)	175 (121 to 244)	1.77 (1.23 to 2.46)	0.27 (0.18 to 0.36)
Madagascar	Diet high in red meat	9 (1 to 24)	0.18 (0.01 to 0.48)	15 (1 to 44)	0.15 (0.01 to 0.41)	-0.72 (-0.86 to -0.59)
Madagascar	Diet high in processed meat	5 (1 to 10)	0.1 (0.02 to 0.19)	11 (2 to 23)	0.11 (0.01 to 0.21)	0.11 (0.04 to 0.18)
Madagascar	Diet low in whole grains	44 (17 to 64)	0.92 (0.34 to 1.32)	94 (34 to 152)	0.94 (0.36 to 1.49)	-0.01 (-0.09 to 0.07)
Malawi	All dietary risks	63 (49 to 78)	1.84 (1.42 to 2.25)	153 (110 to 197)	2.33 (1.71 to 2.97)	0.92 (0.78 to 1.07)
Malawi	Diet low in milk	20 (8 to 30)	0.59 (0.22 to 0.86)	50 (19 to 77)	0.76 (0.28 to 1.16)	0.99 (0.85 to 1.14)
Malawi	Diet low in fiber	0 (0 to 1)	0.01 (0.01 to 0.02)	1 (1 to 1)	0.01 (0.01 to 0.02)	0.23 (0.16 to 0.29)
Malawi	Diet low in calcium	46 (34 to 58)	1.34 (1.01 to 1.69)	108 (77 to 141)	1.65 (1.19 to 2.14)	0.82 (0.68 to 0.96)
Malawi	Diet high in red meat	2 (0 to 4)	0.05 (0.01 to 0.11)	6 (1 to 15)	0.09 (0.01 to 0.22)	2.57 (2.36 to 2.79)
Malawi	Diet high in processed meat	2 (0 to 5)	0.07 (0.01 to 0.13)	7 (1 to 13)	0.1 (0.01 to 0.18)	1.41 (1.22 to 1.59)

Countries	Risk factors	1990 Number of deaths (95% UI)	1990 ASDR per 100,000 (95% UI)	2019 Number of deaths (95% UI)	2019 ASDR per 100,000 (95% UI)	1990-2019 EAPC of ASDR (95% CI)
Malawi	Diet low in whole grains	20 (8 to 30)	0.59 (0.22 to 0.86)	50 (19 to 77)	0.76 (0.28 to 1.16)	0.99 (0.85 to 1.14)
Malaysia	All dietary risks	564 (448 to 681)	6.74 (5.28 to 8.23)	1857 (1370 to 2408)	7.84 (5.82 to 10.13)	-0.03 (-0.24 to 0.18)
Malaysia	Diet low in milk	206 (78 to 283)	2.45 (0.93 to 3.42)	734 (273 to 1043)	3.08 (1.13 to 4.38)	0.2 (-0.03 to 0.43)
Malaysia	Diet low in fiber	79 (35 to 123)	0.94 (0.42 to 1.49)	180 (69 to 342)	0.78 (0.3 to 1.46)	-1.13 (-1.33 to -0.94)
Malaysia	Diet low in calcium	331 (258 to 419)	3.97 (3.06 to 5.06)	1060 (763 to 1414)	4.51 (3.26 to 6.02)	-0.08 (-0.29 to 0.12)
Malaysia	Diet high in red meat	30 (3 to 82)	0.35 (0.03 to 0.95)	97 (10 to 263)	0.4 (0.04 to 1.09)	-0.37 (-0.65 to -0.1)
Malaysia	Diet high in processed meat	15 (3 to 27)	0.17 (0.03 to 0.31)	70 (10 to 136)	0.28 (0.04 to 0.54)	1.34 (1.16 to 1.52)
Malaysia	Diet low in whole grains	206 (78 to 283)	2.45 (0.93 to 3.42)	734 (273 to 1043)	3.08 (1.13 to 4.38)	0.2 (-0.03 to 0.43)
Maldives	All dietary risks	3 (2 to 4)	3.7 (2.68 to 4.79)	8 (6 to 10)	3.04 (2.15 to 3.85)	-1.21 (-1.4 to -1.02)
Maldives	Diet low in milk	1 (0 to 2)	1.58 (0.6 to 2.21)	4 (1 to 5)	1.45 (0.53 to 1.98)	-0.75 (-0.92 to -0.58)
Maldives	Diet low in fiber	0 (0 to 0)	0.35 (0.13 to 0.65)	1 (0 to 2)	0.34 (0.13 to 0.62)	-0.73 (-0.99 to -0.47)
Maldives	Diet low in calcium	2 (1 to 2)	2.21 (1.54 to 2.95)	4 (3 to 5)	1.51 (1.05 to 2.06)	-1.95 (-2.19 to -1.71)
Maldives	Diet high in red meat	0 (0 to 0)	0.06 (0.02 to 0.12)	0 (0 to 1)	0.1 (0.02 to 0.22)	1.42 (1.3 to 1.54)
Maldives	Diet high in processed meat	0 (0 to 0)	0.05 (0.03 to 0.08)	0 (0 to 0)	0.06 (0.03 to 0.09)	-0.23 (-0.41 to -0.05)
Maldives	Diet low in whole grains	1 (0 to 2)	1.58 (0.6 to 2.21)	4 (1 to 5)	1.45 (0.53 to 1.98)	-0.75 (-0.92 to -0.58)
Mali	All dietary risks	98 (76 to 122)	2.6 (2.03 to 3.22)	204 (142 to 275)	2.64 (1.85 to 3.5)	-0.07 (-0.16 to 0.02)
Mali	Diet low in milk	42 (15 to 57)	1.1 (0.42 to 1.5)	93 (33 to 133)	1.2 (0.43 to 1.68)	0.24 (0.17 to 0.31)
Mali	Diet low in fiber	3 (1 to 6)	0.07 (0.03 to 0.17)	2 (1 to 5)	0.03 (0.02 to 0.07)	-3.05 (-3.59 to -2.5)

Countries	Risk factors	1990 Number of deaths (95% UI)	1990 ASDR per 100,000 (95% UI)	2019 Number of deaths (95% UI)	2019 ASDR per 100,000 (95% UI)	1990-2019 EAPC of ASDR (95% CI)
Mali	Diet low in calcium	56 (44 to 72)	1.51 (1.19 to 1.93)	100 (67 to 142)	1.32 (0.91 to 1.85)	-0.75 (-0.91 to -0.6)
Mali	Diet high in red meat	7 (1 to 18)	0.17 (0.01 to 0.47)	18 (2 to 51)	0.23 (0.02 to 0.63)	1.23 (1.14 to 1.31)
Mali	Diet high in processed meat	6 (1 to 11)	0.15 (0.02 to 0.27)	17 (2 to 31)	0.2 (0.03 to 0.38)	1.17 (1.1 to 1.24)
Mali	Diet low in whole grains	42 (15 to 57)	1.1 (0.42 to 1.5)	93 (33 to 133)	1.2 (0.43 to 1.68)	0.24 (0.17 to 0.31)
Malta	All dietary risks	25 (18 to 31)	6.05 (4.45 to 7.38)	47 (33 to 59)	4.84 (3.39 to 6.12)	-0.63 (-0.74 to -0.52)
Malta	Diet low in milk	14 (6 to 18)	3.4 (1.52 to 4.25)	28 (14 to 37)	2.91 (1.41 to 3.78)	-0.43 (-0.56 to -0.31)
Malta	Diet low in fiber	1 (0 to 3)	0.34 (0.11 to 0.75)	2 (1 to 4)	0.17 (0.06 to 0.42)	-1.52 (-1.94 to -1.09)
Malta	Diet low in calcium	5 (3 to 9)	1.3 (0.66 to 2.26)	8 (4 to 15)	0.83 (0.39 to 1.47)	-1.07 (-1.25 to -0.88)
Malta	Diet high in red meat	3 (1 to 7)	0.81 (0.13 to 1.79)	6 (1 to 14)	0.66 (0.12 to 1.47)	-0.9 (-1.06 to -0.73)
Malta	Diet high in processed meat	5 (2 to 7)	1.12 (0.38 to 1.73)	9 (3 to 14)	0.94 (0.33 to 1.49)	-0.56 (-0.71 to -0.41)
Malta	Diet low in whole grains	14 (6 to 18)	3.4 (1.52 to 4.25)	28 (14 to 37)	2.91 (1.41 to 3.78)	-0.43 (-0.56 to -0.31)
Marshall Islands	All dietary risks	1 (1 to 1)	4.33 (3.39 to 5.42)	1 (1 to 2)	4.79 (3.47 to 6.23)	0.4 (0.34 to 0.46)
Marshall Islands	Diet low in milk	0 (0 to 0)	1.8 (0.69 to 2.47)	1 (0 to 1)	2.03 (0.75 to 2.84)	0.46 (0.4 to 0.52)
Marshall Islands	Diet low in fiber	0 (0 to 0)	0.05 (0.03 to 0.13)	0 (0 to 0)	0.05 (0.03 to 0.11)	-0.19 (-0.25 to -0.14)
Marshall Islands	Diet low in calcium	0 (0 to 1)	2.71 (2.04 to 3.51)	1 (1 to 1)	2.95 (2.17 to 3.92)	0.35 (0.29 to 0.4)
Marshall Islands	Diet high in red meat	0 (0 to 0)	0.29 (0.03 to 0.78)	0 (0 to 0)	0.33 (0.03 to 0.92)	0.43 (0.37 to 0.49)
Marshall Islands	Diet high in processed meat	0 (0 to 0)	0.09 (0.03 to 0.15)	0 (0 to 0)	0.1 (0.03 to 0.19)	0.68 (0.61 to 0.74)
Marshall Islands	Diet low in whole grains	0 (0 to 0)	1.8 (0.69 to 2.47)	1 (0 to 1)	2.03 (0.75 to 2.84)	0.46 (0.4 to 0.52)

Countries	Risk factors	1990 Number of deaths (95% UI)	1990 ASDR per 100,000 (95% UI)	2019 Number of deaths (95% UI)	2019 ASDR per 100,000 (95% UI)	1990-2019 EAPC of ASDR (95% CI)
Mauritania	All dietary risks	25 (16 to 34)	2.7 (1.77 to 3.62)	48 (31 to 66)	2.59 (1.68 to 3.58)	-0.06 (-0.19 to 0.07)
Mauritania	Diet low in milk	14 (5 to 19)	1.48 (0.57 to 2.07)	27 (10 to 39)	1.46 (0.53 to 2.08)	0.05 (-0.08 to 0.18)
Mauritania	Diet low in fiber	1 (0 to 3)	0.16 (0.05 to 0.36)	1 (0 to 3)	0.07 (0.03 to 0.18)	-2.52 (-2.84 to -2.21)
Mauritania	Diet low in calcium	8 (5 to 14)	0.94 (0.52 to 1.49)	16 (9 to 27)	0.9 (0.5 to 1.44)	-0.17 (-0.26 to -0.07)
Mauritania	Diet high in red meat	2 (0 to 7)	0.25 (0.02 to 0.7)	5 (0 to 13)	0.24 (0.02 to 0.67)	0.09 (-0.07 to 0.24)
Mauritania	Diet high in processed meat	2 (0 to 4)	0.22 (0.03 to 0.42)	4 (1 to 8)	0.23 (0.03 to 0.41)	0.3 (0.17 to 0.43)
Mauritania	Diet low in whole grains	14 (5 to 19)	1.48 (0.57 to 2.07)	27 (10 to 39)	1.46 (0.53 to 2.08)	0.05 (-0.08 to 0.18)
Mauritius	All dietary risks	20 (15 to 24)	2.95 (2.27 to 3.5)	63 (41 to 83)	3.78 (2.49 to 4.96)	0.83 (0.73 to 0.94)
Mauritius	Diet low in milk	9 (3 to 12)	1.28 (0.47 to 1.7)	34 (12 to 48)	2.01 (0.74 to 2.84)	1.58 (1.41 to 1.75)
Mauritius	Diet low in fiber	3 (1 to 5)	0.47 (0.21 to 0.7)	5 (2 to 11)	0.33 (0.12 to 0.67)	-1.33 (-1.54 to -1.12)
Mauritius	Diet low in calcium	9 (7 to 13)	1.42 (1.04 to 1.86)	24 (14 to 37)	1.48 (0.88 to 2.25)	0.11 (0.01 to 0.2)
Mauritius	Diet high in red meat	1 (0 to 3)	0.18 (0.02 to 0.48)	5 (0 to 13)	0.28 (0.03 to 0.79)	1.42 (1.24 to 1.6)
Mauritius	Diet high in processed meat	0 (0 to 1)	0.06 (0.03 to 0.09)	2 (1 to 3)	0.1 (0.04 to 0.17)	1.92 (1.76 to 2.08)
Mauritius	Diet low in whole grains	9 (3 to 12)	1.28 (0.47 to 1.7)	34 (12 to 48)	2.01 (0.74 to 2.84)	1.58 (1.41 to 1.75)
Mexico	All dietary risks	796 (596 to 975)	2.02 (1.51 to 2.47)	3112 (2137 to 4058)	2.74 (1.88 to 3.56)	1.15 (1.02 to 1.28)
Mexico	Diet low in milk	323 (122 to 444)	0.81 (0.31 to 1.12)	1323 (493 to 1886)	1.16 (0.43 to 1.65)	1.32 (1.22 to 1.43)
Mexico	Diet low in fiber	10 (6 to 20)	0.03 (0.02 to 0.05)	51 (26 to 110)	0.05 (0.02 to 0.1)	2.37 (2.1 to 2.63)
Mexico	Diet low in calcium	417 (301 to 554)	1.07 (0.77 to 1.42)	1464 (974 to 2092)	1.3 (0.87 to 1.85)	0.73 (0.55 to 0.92)

Countries	Risk factors	1990		2019		1990-2019
		Number of deaths (95% UI)	ASDR per 100,000 (95% UI)	Number of deaths (95% UI)	ASDR per 100,000 (95% UI)	EAPC of ASDR (95% CI)
Mexico	Diet high in red meat	101 (16 to 231)	0.25 (0.04 to 0.58)	463 (89 to 1017)	0.4 (0.07 to 0.89)	1.84 (1.76 to 1.92)
Mexico	Diet high in processed meat	41 (8 to 68)	0.1 (0.02 to 0.17)	174 (37 to 302)	0.15 (0.03 to 0.26)	1.62 (1.54 to 1.69)
Mexico	Diet low in whole grains	323 (122 to 444)	0.81 (0.31 to 1.12)	1323 (493 to 1886)	1.16 (0.43 to 1.65)	1.32 (1.22 to 1.43)
Micronesia (Federated States of)	All dietary risks	2 (1 to 3)	4.5 (3.37 to 5.85)	3 (2 to 4)	5.12 (3.53 to 6.81)	0.38 (0.35 to 0.41)
Micronesia (Federated States of)	Diet low in milk	1 (0 to 1)	1.87 (0.71 to 2.64)	1 (0 to 2)	2.16 (0.8 to 3.12)	0.44 (0.41 to 0.47)
Micronesia (Federated States of)	Diet low in fiber	0 (0 to 0)	0.06 (0.03 to 0.14)	0 (0 to 0)	0.05 (0.03 to 0.12)	-0.21 (-0.26 to -0.15)
Micronesia (Federated States of)	Diet low in calcium	1 (1 to 2)	2.82 (2.09 to 3.74)	2 (1 to 3)	3.17 (2.17 to 4.28)	0.34 (0.31 to 0.37)
Micronesia (Federated States of)	Diet high in red meat	0 (0 to 0)	0.3 (0.03 to 0.85)	0 (0 to 1)	0.35 (0.03 to 0.97)	0.37 (0.33 to 0.4)
Micronesia (Federated States of)	Diet high in processed meat	0 (0 to 0)	0.09 (0.03 to 0.16)	0 (0 to 0)	0.11 (0.03 to 0.2)	0.66 (0.62 to 0.7)
Micronesia (Federated States of)	Diet low in whole grains	1 (0 to 1)	1.87 (0.71 to 2.64)	1 (0 to 2)	2.16 (0.8 to 3.12)	0.44 (0.41 to 0.47)
Monaco	All dietary risks	5 (3 to 7)	6.83 (4.49 to 9.11)	8 (5 to 10)	7.36 (5.02 to 9.79)	0.37 (0.28 to 0.45)
Monaco	Diet low in milk	3 (1 to 4)	3.79 (1.45 to 5.33)	4 (2 to 6)	4.04 (1.5 to 5.57)	0.34 (0.24 to 0.44)
Monaco	Diet low in fiber	0 (0 to 0)	0.1 (0.05 to 0.26)	0 (0 to 0)	0.09 (0.05 to 0.19)	-0.19 (-0.4 to 0.02)
Monaco	Diet low in calcium	0 (0 to 0)	0.05 (0.04 to 0.07)	0 (0 to 0)	0.05 (0.04 to 0.06)	0.23 (0.05 to 0.41)

Countries	Risk factors	1990 Number of deaths (95% UI)	1990 ASDR per 100,000 (95% UI)	2019 Number of deaths (95% UI)	2019 ASDR per 100,000 (95% UI)	1990-2019 EAPC of ASDR (95% CI)
Monaco	Diet high in red meat	2 (1 to 3)	2.21 (0.84 to 3.73)	3 (1 to 4)	2.46 (0.99 to 4.11)	0.43 (0.37 to 0.5)
Monaco	Diet high in processed meat	1 (0 to 2)	1.44 (0.48 to 2.34)	2 (1 to 3)	1.56 (0.56 to 2.56)	0.38 (0.3 to 0.47)
Monaco	Diet low in whole grains	3 (1 to 4)	3.79 (1.45 to 5.33)	4 (2 to 6)	4.04 (1.5 to 5.57)	0.34 (0.24 to 0.44)
Mongolia	All dietary risks	44 (34 to 55)	4.38 (3.41 to 5.39)	86 (63 to 115)	4.17 (3.09 to 5.46)	-0.68 (-0.84 to -0.51)
Mongolia	Diet low in milk	19 (9 to 25)	1.87 (0.92 to 2.46)	41 (19 to 56)	1.99 (0.94 to 2.68)	-0.22 (-0.37 to -0.08)
Mongolia	Diet low in fiber	7 (3 to 10)	0.66 (0.33 to 0.98)	10 (4 to 17)	0.5 (0.21 to 0.81)	-1.6 (-1.84 to -1.36)
Mongolia	Diet low in calcium	15 (10 to 20)	1.49 (1.04 to 2.03)	21 (12 to 34)	1.06 (0.62 to 1.68)	-1.78 (-2.02 to -1.55)
Mongolia	Diet high in red meat	13 (7 to 19)	1.25 (0.64 to 1.89)	27 (13 to 43)	1.24 (0.6 to 1.96)	-0.53 (-0.75 to -0.32)
Mongolia	Diet high in processed meat	3 (0 to 6)	0.3 (0.04 to 0.54)	7 (1 to 13)	0.32 (0.05 to 0.57)	-0.23 (-0.44 to -0.02)
Mongolia	Diet low in whole grains	19 (9 to 25)	1.87 (0.92 to 2.46)	41 (19 to 56)	1.99 (0.94 to 2.68)	-0.22 (-0.37 to -0.08)
Montenegro	All dietary risks	24 (15 to 31)	3.9 (2.52 to 5.11)	46 (28 to 62)	4.71 (2.85 to 6.35)	0.89 (0.83 to 0.96)
Montenegro	Diet low in milk	15 (6 to 20)	2.47 (0.93 to 3.32)	29 (11 to 40)	3 (1.11 to 4.13)	1 (0.91 to 1.08)
Montenegro	Diet low in fiber	1 (0 to 2)	0.12 (0.04 to 0.32)	1 (0 to 3)	0.13 (0.05 to 0.33)	-0.49 (-0.99 to 0)
Montenegro	Diet low in calcium	2 (1 to 3)	0.31 (0.12 to 0.54)	2 (1 to 3)	0.2 (0.08 to 0.36)	-2.98 (-3.64 to -2.32)
Montenegro	Diet high in red meat	4 (1 to 9)	0.64 (0.1 to 1.48)	9 (2 to 18)	0.9 (0.17 to 1.88)	2.08 (1.75 to 2.4)
Montenegro	Diet high in processed meat	5 (1 to 7)	0.74 (0.2 to 1.21)	9 (2 to 15)	0.91 (0.24 to 1.5)	1.14 (1 to 1.27)
Montenegro	Diet low in whole grains	15 (6 to 20)	2.47 (0.93 to 3.32)	29 (11 to 40)	3 (1.11 to 4.13)	1 (0.91 to 1.08)
Morocco	All dietary risks	328 (246 to 401)	2.56 (1.91 to 3.12)	913 (618 to 1237)	3.14 (2.1 to 4.2)	0.6 (0.45 to 0.76)

Countries	Risk factors	1990 Number of deaths (95% UI)	1990 ASDR per 100,000 (95% UI)	2019 Number of deaths (95% UI)	2019 ASDR per 100,000 (95% UI)	1990-2019 EAPC of ASDR (95% CI)
Morocco	Diet low in milk	171 (87 to 223)	1.33 (0.68 to 1.74)	526 (251 to 738)	1.8 (0.87 to 2.52)	0.99 (0.83 to 1.14)
Morocco	Diet low in fiber	2 (1 to 3)	0.02 (0.01 to 0.02)	6 (4 to 8)	0.02 (0.01 to 0.03)	0.7 (0.51 to 0.89)
Morocco	Diet low in calcium	180 (135 to 229)	1.41 (1.04 to 1.79)	419 (271 to 591)	1.46 (0.94 to 2.05)	-0.1 (-0.25 to 0.05)
Morocco	Diet high in red meat	17 (2 to 47)	0.13 (0.01 to 0.35)	60 (5 to 169)	0.2 (0.02 to 0.56)	1.53 (1.36 to 1.7)
Morocco	Diet high in processed meat	5 (2 to 8)	0.04 (0.02 to 0.06)	23 (5 to 44)	0.07 (0.02 to 0.14)	2.59 (2.29 to 2.9)
Morocco	Diet low in whole grains	171 (87 to 223)	1.33 (0.68 to 1.74)	526 (251 to 738)	1.8 (0.87 to 2.52)	0.99 (0.83 to 1.14)
Mozambique	All dietary risks	113 (88 to 139)	2.2 (1.73 to 2.7)	336 (238 to 449)	3.52 (2.48 to 4.66)	2.02 (1.88 to 2.16)
Mozambique	Diet low in milk	44 (16 to 61)	0.85 (0.32 to 1.17)	130 (47 to 192)	1.36 (0.5 to 1.98)	2.02 (1.87 to 2.16)
Mozambique	Diet low in fiber	6 (2 to 12)	0.11 (0.04 to 0.25)	6 (2 to 15)	0.07 (0.03 to 0.17)	-1.9 (-2.28 to -1.53)
Mozambique	Diet low in calcium	74 (56 to 96)	1.45 (1.1 to 1.86)	222 (151 to 303)	2.33 (1.6 to 3.15)	2.04 (1.9 to 2.17)
Mozambique	Diet high in red meat	3 (1 to 7)	0.06 (0.01 to 0.13)	12 (2 to 31)	0.12 (0.02 to 0.3)	2.96 (2.82 to 3.11)
Mozambique	Diet high in processed meat	4 (1 to 7)	0.07 (0.01 to 0.13)	13 (2 to 28)	0.13 (0.02 to 0.27)	2.89 (2.71 to 3.07)
Mozambique	Diet low in whole grains	44 (16 to 61)	0.85 (0.32 to 1.17)	130 (47 to 192)	1.36 (0.5 to 1.98)	2.02 (1.87 to 2.16)
Myanmar	All dietary risks	879 (650 to 1234)	3.97 (2.99 to 5.59)	2206 (1559 to 2907)	5.09 (3.6 to 6.68)	0.92 (0.83 to 1.01)
Myanmar	Diet low in milk	270 (103 to 415)	1.22 (0.47 to 1.84)	737 (271 to 1148)	1.69 (0.62 to 2.6)	1.27 (1.18 to 1.35)
Myanmar	Diet low in fiber	178 (93 to 276)	0.8 (0.43 to 1.25)	288 (124 to 483)	0.68 (0.29 to 1.13)	-0.86 (-1.01 to -0.71)
Myanmar	Diet low in calcium	557 (392 to 800)	2.52 (1.81 to 3.61)	1364 (952 to 1858)	3.16 (2.23 to 4.3)	0.82 (0.73 to 0.91)
Myanmar	Diet high in red meat	23 (4 to 57)	0.1 (0.02 to 0.25)	124 (11 to 352)	0.28 (0.03 to 0.78)	4.29 (4.01 to 4.57)

Countries	Risk factors	1990 Number of deaths (95% UI)	1990 ASDR per 100,000 (95% UI)	2019 Number of deaths (95% UI)	2019 ASDR per 100,000 (95% UI)	1990-2019 EAPC of ASDR (95% CI)
Myanmar	Diet high in processed meat	11 (6 to 17)	0.05 (0.03 to 0.07)	36 (16 to 63)	0.08 (0.04 to 0.14)	2.19 (2.07 to 2.31)
Myanmar	Diet low in whole grains	270 (103 to 415)	1.22 (0.47 to 1.84)	737 (271 to 1148)	1.69 (0.62 to 2.6)	1.27 (1.18 to 1.35)
Namibia	All dietary risks	16 (12 to 21)	2.42 (1.84 to 3.06)	43 (32 to 57)	3.36 (2.53 to 4.32)	1.25 (1.16 to 1.33)
Namibia	Diet low in milk	6 (3 to 9)	0.97 (0.38 to 1.32)	17 (6 to 24)	1.29 (0.47 to 1.87)	1.05 (0.96 to 1.14)
Namibia	Diet low in fiber	1 (0 to 2)	0.16 (0.06 to 0.32)	3 (1 to 5)	0.21 (0.08 to 0.42)	1.19 (1.1 to 1.27)
Namibia	Diet low in calcium	10 (7 to 13)	1.44 (1.07 to 1.89)	26 (19 to 34)	2 (1.5 to 2.63)	1.25 (1.17 to 1.33)
Namibia	Diet high in red meat	1 (0 to 3)	0.19 (0.02 to 0.51)	4 (0 to 10)	0.27 (0.02 to 0.73)	1.35 (1.25 to 1.44)
Namibia	Diet high in processed meat	0 (0 to 1)	0.06 (0.01 to 0.12)	1 (0 to 2)	0.09 (0.02 to 0.18)	1.58 (1.48 to 1.69)
Namibia	Diet low in whole grains	6 (3 to 9)	0.97 (0.38 to 1.32)	17 (6 to 24)	1.29 (0.47 to 1.87)	1.05 (0.96 to 1.14)
Nauru	All dietary risks	0 (0 to 0)	6.5 (4.11 to 9.41)	0 (0 to 0)	6.09 (4.08 to 8.23)	-0.38 (-0.53 to -0.24)
Nauru	Diet low in milk	0 (0 to 0)	2.96 (1.1 to 4.66)	0 (0 to 0)	2.71 (1.01 to 3.94)	-0.47 (-0.69 to -0.26)
Nauru	Diet low in fiber	0 (0 to 0)	0.04 (0.03 to 0.06)	0 (0 to 0)	0.04 (0.03 to 0.07)	0.28 (-0.18 to 0.75)
Nauru	Diet low in calcium	0 (0 to 0)	3.6 (2.25 to 5.43)	0 (0 to 0)	3.52 (2.35 to 4.97)	-0.24 (-0.3 to -0.17)
Nauru	Diet high in red meat	0 (0 to 0)	0.59 (0.06 to 1.62)	0 (0 to 0)	0.49 (0.05 to 1.31)	-0.85 (-1.27 to -0.44)
Nauru	Diet high in processed meat	0 (0 to 0)	0.16 (0.04 to 0.34)	0 (0 to 0)	0.15 (0.04 to 0.29)	-0.47 (-0.84 to -0.11)
Nauru	Diet low in whole grains	0 (0 to 0)	2.96 (1.1 to 4.66)	0 (0 to 0)	2.71 (1.01 to 3.94)	-0.47 (-0.69 to -0.26)
Nepal	All dietary risks	126 (81 to 180)	1.47 (0.93 to 2.14)	398 (276 to 573)	1.99 (1.39 to 2.85)	1.05 (0.84 to 1.27)
Nepal	Diet low in milk	42 (16 to 67)	0.49 (0.19 to 0.8)	148 (57 to 236)	0.74 (0.28 to 1.17)	1.49 (1.29 to 1.69)

Countries	Risk factors	1990 Number of deaths (95% UI)	1990 ASDR per 100,000 (95% UI)	2019 Number of deaths (95% UI)	2019 ASDR per 100,000 (95% UI)	1990-2019 EAPC of ASDR (95% CI)
Nepal	Diet low in fiber	11 (4 to 22)	0.13 (0.04 to 0.26)	15 (5 to 36)	0.08 (0.03 to 0.19)	-1.98 (-2.35 to -1.6)
Nepal	Diet low in calcium	78 (50 to 113)	0.92 (0.57 to 1.35)	234 (161 to 334)	1.18 (0.82 to 1.69)	0.83 (0.63 to 1.02)
Nepal	Diet high in red meat	7 (1 to 19)	0.08 (0.01 to 0.21)	24 (2 to 69)	0.12 (0.01 to 0.34)	1.61 (1.35 to 1.87)
Nepal	Diet high in processed meat	8 (1 to 17)	0.09 (0.01 to 0.18)	33 (5 to 63)	0.16 (0.02 to 0.3)	2.03 (1.82 to 2.24)
Nepal	Diet low in whole grains	42 (16 to 67)	0.49 (0.19 to 0.8)	148 (57 to 236)	0.74 (0.28 to 1.17)	1.49 (1.29 to 1.69)
Netherlands	All dietary risks	1362 (958 to 1704)	6.75 (4.77 to 8.44)	2319 (1627 to 2922)	6.5 (4.58 to 8.15)	-0.13 (-0.25 to -0.01)
Netherlands	Diet low in milk	727 (271 to 962)	3.6 (1.34 to 4.74)	1262 (478 to 1660)	3.52 (1.33 to 4.63)	-0.1 (-0.23 to 0.04)
Netherlands	Diet low in fiber	149 (56 to 278)	0.73 (0.27 to 1.36)	171 (59 to 360)	0.47 (0.16 to 1)	-1.7 (-1.82 to -1.58)
Netherlands	Diet low in calcium	128 (61 to 218)	0.62 (0.3 to 1.06)	209 (96 to 355)	0.57 (0.26 to 0.96)	0.12 (-0.12 to 0.37)
Netherlands	Diet high in red meat	297 (69 to 560)	1.49 (0.36 to 2.79)	544 (151 to 987)	1.56 (0.44 to 2.8)	0.12 (0.01 to 0.24)
Netherlands	Diet high in processed meat	227 (51 to 355)	1.13 (0.26 to 1.76)	416 (103 to 655)	1.18 (0.31 to 1.84)	0.15 (0.02 to 0.28)
Netherlands	Diet low in whole grains	727 (271 to 962)	3.6 (1.34 to 4.74)	1262 (478 to 1660)	3.52 (1.33 to 4.63)	-0.1 (-0.23 to 0.04)
New Zealand	All dietary risks	402 (302 to 487)	10.4 (7.83 to 12.58)	634 (479 to 769)	7.8 (5.91 to 9.47)	-1.06 (-1.13 to -0.98)
New Zealand	Diet low in milk	168 (63 to 226)	4.35 (1.63 to 5.86)	260 (98 to 340)	3.2 (1.2 to 4.18)	-1.22 (-1.3 to -1.14)
New Zealand	Diet low in fiber	23 (8 to 51)	0.59 (0.2 to 1.31)	34 (12 to 74)	0.42 (0.15 to 0.9)	-0.64 (-0.89 to -0.38)
New Zealand	Diet low in calcium	98 (52 to 165)	2.52 (1.34 to 4.28)	205 (124 to 300)	2.5 (1.51 to 3.65)	0.03 (-0.22 to 0.29)
New Zealand	Diet high in red meat	125 (52 to 192)	3.22 (1.34 to 4.93)	166 (62 to 262)	2.07 (0.79 to 3.24)	-1.69 (-1.85 to -1.53)
New Zealand	Diet high in processed meat	55 (13 to 86)	1.43 (0.35 to 2.22)	86 (22 to 133)	1.07 (0.28 to 1.65)	-1.05 (-1.18 to -0.92)

Countries	Risk factors	1990		2019		1990-2019
		Number of deaths (95% UI)	ASDR per 100,000 (95% UI)	Number of deaths (95% UI)	ASDR per 100,000 (95% UI)	EAPC of ASDR (95% CI)
New Zealand	Diet low in whole grains	168 (63 to 226)	4.35 (1.63 to 5.86)	260 (98 to 340)	3.2 (1.2 to 4.18)	-1.22 (-1.3 to -1.14)
Nicaragua	All dietary risks	34 (26 to 42)	2.37 (1.78 to 3.02)	144 (107 to 180)	3.66 (2.71 to 4.53)	1.52 (1.28 to 1.75)
Nicaragua	Diet low in milk	13 (5 to 18)	0.93 (0.35 to 1.3)	60 (22 to 84)	1.52 (0.55 to 2.11)	1.8 (1.57 to 2.04)
Nicaragua	Diet low in fiber	2 (1 to 4)	0.14 (0.05 to 0.29)	6 (2 to 13)	0.15 (0.05 to 0.35)	-0.41 (-0.82 to 0)
Nicaragua	Diet low in calcium	21 (16 to 27)	1.46 (1.1 to 1.92)	83 (61 to 109)	2.13 (1.59 to 2.78)	1.28 (1.03 to 1.52)
Nicaragua	Diet high in red meat	2 (0 to 4)	0.11 (0.01 to 0.29)	7 (1 to 17)	0.17 (0.02 to 0.43)	1.8 (1.63 to 1.97)
Nicaragua	Diet high in processed meat	1 (0 to 2)	0.07 (0.01 to 0.13)	5 (1 to 10)	0.13 (0.02 to 0.25)	2.61 (2.35 to 2.87)
Nicaragua	Diet low in whole grains	13 (5 to 18)	0.93 (0.35 to 1.3)	60 (22 to 84)	1.52 (0.55 to 2.11)	1.8 (1.57 to 2.04)
Niger	All dietary risks	52 (38 to 71)	2.16 (1.58 to 2.9)	131 (91 to 181)	2.01 (1.42 to 2.75)	-0.26 (-0.39 to -0.14)
Niger	Diet low in milk	21 (8 to 31)	0.87 (0.33 to 1.27)	58 (21 to 87)	0.88 (0.34 to 1.31)	0.1 (0 to 0.19)
Niger	Diet low in fiber	2 (1 to 4)	0.07 (0.02 to 0.17)	2 (1 to 5)	0.03 (0.01 to 0.08)	-3.47 (-3.89 to -3.05)
Niger	Diet low in calcium	31 (22 to 42)	1.27 (0.92 to 1.73)	69 (46 to 100)	1.08 (0.74 to 1.55)	-0.68 (-0.85 to -0.51)
Niger	Diet high in red meat	4 (0 to 11)	0.15 (0.01 to 0.43)	11 (1 to 30)	0.16 (0.01 to 0.44)	0.26 (0.19 to 0.33)
Niger	Diet high in processed meat	3 (0 to 6)	0.12 (0.01 to 0.24)	10 (1 to 19)	0.14 (0.02 to 0.27)	0.58 (0.49 to 0.67)
Niger	Diet low in whole grains	21 (8 to 31)	0.87 (0.33 to 1.27)	58 (21 to 87)	0.88 (0.34 to 1.31)	0.1 (0 to 0.19)
Nigeria	All dietary risks	1006 (710 to 1394)	2.6 (1.86 to 3.53)	2425 (1751 to 3204)	3.3 (2.44 to 4.31)	1.19 (1.02 to 1.37)
Nigeria	Diet low in milk	394 (153 to 600)	1.02 (0.39 to 1.54)	982 (380 to 1428)	1.33 (0.51 to 1.92)	1.34 (1.16 to 1.52)
Nigeria	Diet low in fiber	17 (7 to 41)	0.05 (0.02 to 0.11)	21 (13 to 39)	0.03 (0.02 to 0.05)	-1.51 (-1.87 to -1.15)

Countries	Risk factors	1990 Number of deaths (95% UI)	1990 ASDR per 100,000 (95% UI)	2019 Number of deaths (95% UI)	2019 ASDR per 100,000 (95% UI)	1990-2019 EAPC of ASDR (95% CI)
Nigeria	Diet low in calcium	647 (450 to 912)	1.68 (1.18 to 2.33)	1477 (1064 to 2007)	2.03 (1.48 to 2.71)	0.98 (0.82 to 1.14)
Nigeria	Diet high in red meat	38 (5 to 104)	0.09 (0.01 to 0.26)	110 (13 to 295)	0.14 (0.02 to 0.38)	2 (1.77 to 2.22)
Nigeria	Diet high in processed meat	57 (8 to 114)	0.14 (0.02 to 0.28)	174 (27 to 322)	0.22 (0.03 to 0.41)	2.05 (1.86 to 2.25)
Nigeria	Diet low in whole grains	394 (153 to 600)	1.02 (0.39 to 1.54)	982 (380 to 1428)	1.33 (0.51 to 1.92)	1.34 (1.16 to 1.52)
Niue	All dietary risks	0 (0 to 0)	4.83 (3.67 to 6.21)	0 (0 to 0)	4.93 (3.48 to 6.55)	0.02 (-0.01 to 0.05)
Niue	Diet low in milk	0 (0 to 0)	2.11 (0.78 to 2.94)	0 (0 to 0)	2.22 (0.78 to 3.16)	0.14 (0.11 to 0.18)
Niue	Diet low in fiber	0 (0 to 0)	0.04 (0.02 to 0.06)	0 (0 to 0)	0.03 (0.02 to 0.05)	-0.5 (-0.56 to -0.45)
Niue	Diet low in calcium	0 (0 to 0)	2.86 (2.21 to 3.73)	0 (0 to 0)	2.8 (1.99 to 3.85)	-0.15 (-0.19 to -0.12)
Niue	Diet high in red meat	0 (0 to 0)	0.38 (0.04 to 1)	0 (0 to 0)	0.41 (0.04 to 1.09)	0.28 (0.23 to 0.34)
Niue	Diet high in processed meat	0 (0 to 0)	0.11 (0.03 to 0.19)	0 (0 to 0)	0.12 (0.03 to 0.24)	0.52 (0.46 to 0.57)
Niue	Diet low in whole grains	0 (0 to 0)	2.11 (0.78 to 2.94)	0 (0 to 0)	2.22 (0.78 to 3.16)	0.14 (0.11 to 0.18)
North Macedonia	All dietary risks	90 (63 to 109)	5.02 (3.54 to 6.14)	208 (133 to 286)	6.77 (4.33 to 9.21)	1.06 (0.79 to 1.34)
North Macedonia	Diet low in milk	49 (20 to 64)	2.76 (1.11 to 3.56)	124 (49 to 177)	4.03 (1.57 to 5.72)	1.45 (1.18 to 1.71)
North Macedonia	Diet low in fiber	3 (1 to 8)	0.19 (0.06 to 0.46)	7 (2 to 18)	0.24 (0.08 to 0.59)	0.68 (0.24 to 1.13)
North Macedonia	Diet low in calcium	31 (19 to 48)	1.79 (1.1 to 2.69)	54 (27 to 94)	1.8 (0.92 to 3.06)	-0.29 (-0.65 to 0.07)
North Macedonia	Diet high in red meat	7 (1 to 20)	0.4 (0.03 to 1.08)	18 (2 to 53)	0.59 (0.05 to 1.69)	1.6 (1.37 to 1.84)
North Macedonia	Diet high in processed meat	10 (2 to 17)	0.56 (0.1 to 0.93)	28 (6 to 50)	0.9 (0.18 to 1.58)	1.93 (1.68 to 2.18)
North Macedonia	Diet low in whole grains	49 (20 to 64)	2.76 (1.11 to 3.56)	124 (49 to 177)	4.03 (1.57 to 5.72)	1.45 (1.18 to 1.71)

Countries	Risk factors	1990 Number of deaths (95% UI)	1990 ASDR per 100,000 (95% UI)	2019 Number of deaths (95% UI)	2019 ASDR per 100,000 (95% UI)	1990-2019 EAPC of ASDR (95% CI)
Northern Mariana Islands	All dietary risks	1 (1 to 1)	6.42 (4.43 to 8.28)	3 (2 to 3)	6.2 (4.54 to 7.77)	-0.05 (-0.19 to 0.09)
Northern Mariana Islands	Diet low in milk	0 (0 to 1)	3.15 (1.2 to 4.43)	1 (0 to 2)	2.98 (1.12 to 4.02)	-0.15 (-0.29 to -0.02)
Northern Mariana Islands	Diet low in fiber	0 (0 to 0)	0.04 (0.03 to 0.05)	0 (0 to 0)	0.04 (0.03 to 0.05)	-0.15 (-0.28 to -0.03)
Northern Mariana Islands	Diet low in calcium	0 (0 to 1)	3.07 (2.02 to 4.24)	1 (1 to 2)	3.15 (2.27 to 4.24)	0.23 (0.06 to 0.39)
Northern Mariana Islands	Diet high in red meat	0 (0 to 0)	0.75 (0.1 to 1.77)	0 (0 to 1)	0.63 (0.07 to 1.58)	-0.65 (-0.79 to -0.51)
Northern Mariana Islands	Diet high in processed meat	0 (0 to 0)	0.19 (0.04 to 0.36)	0 (0 to 0)	0.18 (0.04 to 0.32)	-0.29 (-0.42 to -0.16)
Northern Mariana Islands	Diet low in whole grains	0 (0 to 1)	3.15 (1.2 to 4.43)	1 (0 to 2)	2.98 (1.12 to 4.02)	-0.15 (-0.29 to -0.02)
Norway	All dietary risks	536 (386 to 660)	7.55 (5.47 to 9.3)	678 (492 to 839)	6.61 (4.81 to 8.17)	-0.43 (-0.55 to -0.3)
Norway	Diet low in milk	291 (119 to 371)	4.1 (1.67 to 5.21)	356 (140 to 458)	3.46 (1.35 to 4.45)	-0.62 (-0.73 to -0.51)
Norway	Diet low in fiber	48 (18 to 89)	0.66 (0.25 to 1.25)	46 (18 to 90)	0.43 (0.16 to 0.85)	-1.6 (-1.78 to -1.41)
Norway	Diet low in calcium	74 (35 to 127)	1.01 (0.48 to 1.71)	96 (50 to 161)	0.91 (0.47 to 1.53)	0.16 (-0.04 to 0.36)
Norway	Diet high in red meat	79 (16 to 166)	1.14 (0.24 to 2.38)	122 (32 to 232)	1.22 (0.33 to 2.3)	0.25 (0.04 to 0.45)
Norway	Diet high in processed meat	121 (46 to 205)	1.72 (0.65 to 2.94)	157 (60 to 265)	1.55 (0.59 to 2.66)	-0.35 (-0.51 to -0.19)
Norway	Diet low in whole grains	291 (119 to 371)	4.1 (1.67 to 5.21)	356 (140 to 458)	3.46 (1.35 to 4.45)	-0.62 (-0.73 to -0.51)
Oman	All dietary risks	15 (9 to 22)	2.67 (1.68 to 3.76)	37 (23 to 51)	2.9 (1.9 to 3.86)	0.64 (0.51 to 0.77)
Oman	Diet low in milk	8 (3 to 12)	1.35 (0.53 to 1.97)	22 (8 to 31)	1.69 (0.61 to 2.34)	1.2 (1.01 to 1.39)
Oman	Diet low in fiber	1 (0 to 2)	0.13 (0.04 to 0.32)	1 (0 to 2)	0.08 (0.03 to 0.19)	-1.9 (-2.45 to -1.33)
Oman	Diet low in calcium	7 (4 to 11)	1.26 (0.76 to 1.88)	12 (6 to 21)	1.02 (0.58 to 1.66)	-0.67 (-0.83 to -0.51)

Countries	Risk factors	1990 Number of deaths (95% UI)	1990 ASDR per 100,000 (95% UI)	2019 Number of deaths (95% UI)	2019 ASDR per 100,000 (95% UI)	1990-2019 EAPC of ASDR (95% CI)
Oman	Diet high in red meat	1 (0 to 4)	0.22 (0.02 to 0.62)	5 (0 to 12)	0.32 (0.03 to 0.87)	2.06 (1.74 to 2.38)
Oman	Diet high in processed meat	0 (0 to 1)	0.06 (0.02 to 0.12)	1 (0 to 2)	0.09 (0.02 to 0.17)	1.93 (1.63 to 2.22)
Oman	Diet low in whole grains	8 (3 to 12)	1.35 (0.53 to 1.97)	22 (8 to 31)	1.69 (0.61 to 2.34)	1.2 (1.01 to 1.39)
Pakistan	All dietary risks	1260 (996 to 1523)	2.32 (1.83 to 2.81)	3348 (2405 to 4339)	3.29 (2.35 to 4.23)	1.15 (0.92 to 1.37)
Pakistan	Diet low in milk	607 (288 to 781)	1.12 (0.53 to 1.45)	1780 (813 to 2436)	1.74 (0.79 to 2.36)	1.41 (1.14 to 1.69)
Pakistan	Diet low in fiber	118 (47 to 201)	0.22 (0.09 to 0.38)	292 (110 to 545)	0.29 (0.11 to 0.53)	1.13 (0.87 to 1.4)
Pakistan	Diet low in calcium	620 (487 to 795)	1.15 (0.9 to 1.46)	1372 (914 to 1952)	1.38 (0.94 to 1.95)	0.63 (0.47 to 0.79)
Pakistan	Diet high in red meat	64 (6 to 173)	0.12 (0.01 to 0.31)	198 (18 to 551)	0.19 (0.02 to 0.52)	1.53 (1.33 to 1.73)
Pakistan	Diet high in processed meat	84 (13 to 147)	0.15 (0.02 to 0.26)	262 (41 to 483)	0.24 (0.04 to 0.44)	1.49 (1.24 to 1.75)
Pakistan	Diet low in whole grains	607 (288 to 781)	1.12 (0.53 to 1.45)	1780 (813 to 2436)	1.74 (0.79 to 2.36)	1.41 (1.14 to 1.69)
Palau	All dietary risks	0 (0 to 1)	5.05 (3.65 to 6.69)	1 (1 to 1)	4.85 (3.46 to 6.39)	-0.17 (-0.24 to -0.09)
Palau	Diet low in milk	0 (0 to 0)	2.25 (0.86 to 3.2)	0 (0 to 1)	2.22 (0.8 to 3.12)	-0.08 (-0.15 to -0.01)
Palau	Diet low in fiber	0 (0 to 0)	0.03 (0.02 to 0.06)	0 (0 to 0)	0.03 (0.02 to 0.04)	-0.33 (-0.41 to -0.24)
Palau	Diet low in calcium	0 (0 to 0)	2.89 (2.11 to 3.95)	0 (0 to 1)	2.68 (1.93 to 3.67)	-0.28 (-0.36 to -0.2)
Palau	Diet high in red meat	0 (0 to 0)	0.42 (0.04 to 1.1)	0 (0 to 0)	0.42 (0.04 to 1.1)	-0.1 (-0.17 to -0.03)
Palau	Diet high in processed meat	0 (0 to 0)	0.12 (0.03 to 0.22)	0 (0 to 0)	0.13 (0.03 to 0.24)	0.24 (0.16 to 0.32)
Palau	Diet low in whole grains	0 (0 to 0)	2.25 (0.86 to 3.2)	0 (0 to 1)	2.22 (0.8 to 3.12)	-0.08 (-0.15 to -0.01)
Palestine	All dietary risks	53 (36 to 74)	6.49 (4.41 to 8.99)	182 (144 to 221)	8.56 (6.8 to 10.32)	1.13 (0.9 to 1.37)

Countries	Risk factors	1990 Number of deaths (95% UI)	1990 ASDR per 100,000 (95% UI)	2019 Number of deaths (95% UI)	2019 ASDR per 100,000 (95% UI)	1990-2019 EAPC of ASDR (95% CI)
Palestine	Diet low in milk	25 (13 to 37)	3.1 (1.53 to 4.43)	85 (41 to 112)	4.01 (1.95 to 5.21)	1.04 (0.8 to 1.29)
Palestine	Diet low in fiber	4 (2 to 8)	0.54 (0.2 to 1.02)	16 (6 to 27)	0.75 (0.29 to 1.29)	1.52 (1.31 to 1.72)
Palestine	Diet low in calcium	30 (19 to 42)	3.61 (2.37 to 5.13)	102 (79 to 129)	4.82 (3.76 to 6.11)	1.2 (0.97 to 1.42)
Palestine	Diet high in red meat	2 (0 to 6)	0.27 (0.03 to 0.77)	7 (1 to 19)	0.33 (0.04 to 0.85)	0.54 (0.27 to 0.81)
Palestine	Diet high in processed meat	1 (0 to 2)	0.11 (0.04 to 0.2)	3 (1 to 6)	0.15 (0.05 to 0.25)	1.07 (0.81 to 1.33)
Palestine	Diet low in whole grains	25 (13 to 37)	3.1 (1.53 to 4.43)	85 (41 to 112)	4.01 (1.95 to 5.21)	1.04 (0.8 to 1.29)
Panama	All dietary risks	50 (41 to 59)	3.46 (2.81 to 4.07)	153 (106 to 206)	3.66 (2.54 to 4.94)	0.34 (0.25 to 0.43)
Panama	Diet low in milk	18 (7 to 25)	1.26 (0.47 to 1.71)	60 (22 to 89)	1.43 (0.53 to 2.13)	0.6 (0.52 to 0.67)
Panama	Diet low in fiber	7 (3 to 10)	0.46 (0.19 to 0.71)	20 (8 to 36)	0.49 (0.19 to 0.86)	0.38 (0.14 to 0.62)
Panama	Diet low in calcium	28 (22 to 35)	1.93 (1.54 to 2.39)	74 (48 to 105)	1.76 (1.14 to 2.51)	-0.24 (-0.36 to -0.13)
Panama	Diet high in red meat	4 (0 to 11)	0.29 (0.03 to 0.74)	16 (2 to 41)	0.38 (0.04 to 0.98)	1.19 (1.04 to 1.34)
Panama	Diet high in processed meat	1 (0 to 3)	0.1 (0.02 to 0.17)	6 (1 to 11)	0.13 (0.02 to 0.26)	1.37 (1.27 to 1.47)
Panama	Diet low in whole grains	18 (7 to 25)	1.26 (0.47 to 1.71)	60 (22 to 89)	1.43 (0.53 to 2.13)	0.6 (0.52 to 0.67)
Papua New Guinea	All dietary risks	42 (27 to 58)	2.52 (1.63 to 3.49)	127 (87 to 176)	3.01 (2.12 to 4.09)	0.56 (0.53 to 0.59)
Papua New Guinea	Diet low in milk	18 (7 to 27)	1.08 (0.42 to 1.59)	54 (20 to 78)	1.27 (0.49 to 1.86)	0.51 (0.47 to 0.55)
Papua New Guinea	Diet low in fiber	0 (0 to 1)	0.02 (0.01 to 0.04)	1 (1 to 1)	0.02 (0.01 to 0.04)	0.33 (0.25 to 0.42)
Papua New Guinea	Diet low in calcium	26 (16 to 36)	1.57 (1.01 to 2.2)	80 (55 to 111)	1.9 (1.32 to 2.6)	0.63 (0.6 to 0.66)
Papua New Guinea	Diet high in red meat	3 (0 to 8)	0.16 (0.01 to 0.47)	8 (1 to 23)	0.18 (0.02 to 0.51)	0.22 (0.13 to 0.32)

Countries	Risk factors	1990 Number of deaths (95% UI)	1990 ASDR per 100,000 (95% UI)	2019 Number of deaths (95% UI)	2019 ASDR per 100,000 (95% UI)	1990-2019 EAPC of ASDR (95% CI)
Papua New Guinea	Diet high in processed meat	1 (0 to 2)	0.05 (0.02 to 0.09)	3 (1 to 5)	0.06 (0.02 to 0.1)	0.58 (0.52 to 0.65)
Papua New Guinea	Diet low in whole grains	18 (7 to 27)	1.08 (0.42 to 1.59)	54 (20 to 78)	1.27 (0.49 to 1.86)	0.51 (0.47 to 0.55)
Paraguay	All dietary risks	50 (40 to 61)	2.38 (1.87 to 2.9)	218 (144 to 297)	4.08 (2.7 to 5.52)	2.15 (1.98 to 2.32)
Paraguay	Diet low in milk	20 (7 to 27)	0.95 (0.36 to 1.28)	95 (35 to 137)	1.77 (0.66 to 2.56)	2.54 (2.35 to 2.73)
Paraguay	Diet low in fiber	1 (0 to 2)	0.04 (0.02 to 0.1)	4 (2 to 8)	0.07 (0.03 to 0.16)	1.76 (1.47 to 2.05)
Paraguay	Diet low in calcium	23 (17 to 31)	1.12 (0.82 to 1.46)	94 (59 to 140)	1.78 (1.11 to 2.62)	2.01 (1.82 to 2.2)
Paraguay	Diet high in red meat	12 (4 to 20)	0.57 (0.19 to 0.95)	48 (13 to 91)	0.88 (0.23 to 1.68)	1.25 (1.07 to 1.44)
Paraguay	Diet high in processed meat	2 (0 to 3)	0.08 (0.01 to 0.14)	8 (1 to 17)	0.15 (0.03 to 0.31)	2.76 (2.57 to 2.96)
Paraguay	Diet low in whole grains	20 (7 to 27)	0.95 (0.36 to 1.28)	95 (35 to 137)	1.77 (0.66 to 2.56)	2.54 (2.35 to 2.73)
Peru	All dietary risks	324 (250 to 398)	2.87 (2.22 to 3.54)	923 (615 to 1273)	2.87 (1.91 to 3.97)	0.03 (-0.19 to 0.25)
Peru	Diet low in milk	137 (52 to 187)	1.21 (0.47 to 1.65)	444 (164 to 659)	1.38 (0.51 to 2.06)	0.59 (0.37 to 0.82)
Peru	Diet low in fiber	28 (10 to 54)	0.25 (0.09 to 0.49)	31 (11 to 77)	0.1 (0.04 to 0.24)	-3.85 (-4.12 to -3.59)
Peru	Diet low in calcium	183 (137 to 235)	1.62 (1.22 to 2.09)	466 (291 to 682)	1.45 (0.91 to 2.13)	-0.47 (-0.72 to -0.22)
Peru	Diet high in red meat	17 (2 to 45)	0.15 (0.02 to 0.39)	63 (6 to 175)	0.19 (0.02 to 0.55)	1.23 (1.03 to 1.43)
Peru	Diet high in processed meat	5 (3 to 7)	0.04 (0.03 to 0.06)	20 (8 to 36)	0.06 (0.03 to 0.11)	1.88 (1.72 to 2.05)
Peru	Diet low in whole grains	137 (52 to 187)	1.21 (0.47 to 1.65)	444 (164 to 659)	1.38 (0.51 to 2.06)	0.59 (0.37 to 0.82)
Philippines	All dietary risks	1599 (1296 to 1894)	5.65 (4.59 to 6.7)	4672 (3592 to 5908)	6.24 (4.84 to 7.83)	0.19 (-0.07 to 0.45)

Countries	Risk factors	1990 Number of deaths (95% UI)	1990 ASDR per 100,000 (95% UI)	2019 Number of deaths (95% UI)	2019 ASDR per 100,000 (95% UI)	1990-2019 EAPC of ASDR (95% CI)
Philippines	Diet low in milk	515 (194 to 728)	1.81 (0.69 to 2.57)	1440 (552 to 2138)	1.92 (0.74 to 2.84)	0 (-0.28 to 0.27)
Philippines	Diet low in fiber	225 (104 to 336)	0.8 (0.37 to 1.2)	633 (287 to 992)	0.85 (0.39 to 1.33)	0.05 (-0.13 to 0.24)
Philippines	Diet low in calcium	1010 (763 to 1254)	3.59 (2.72 to 4.44)	2905 (2180 to 3856)	3.89 (2.95 to 5.1)	0.14 (-0.13 to 0.42)
Philippines	Diet high in red meat	85 (8 to 226)	0.29 (0.03 to 0.77)	330 (34 to 860)	0.43 (0.04 to 1.12)	1.12 (0.91 to 1.33)
Philippines	Diet high in processed meat	42 (9 to 75)	0.14 (0.03 to 0.25)	171 (26 to 319)	0.22 (0.03 to 0.4)	1.53 (1.18 to 1.88)
Philippines	Diet low in whole grains	515 (194 to 728)	1.81 (0.69 to 2.57)	1440 (552 to 2138)	1.92 (0.74 to 2.84)	0 (-0.28 to 0.27)
Poland	All dietary risks	2867 (2010 to 3549)	6.7 (4.7 to 8.27)	5348 (3597 to 6924)	7.46 (5.01 to 9.7)	0.26 (0.1 to 0.41)
Poland	Diet low in milk	1724 (718 to 2187)	4.02 (1.68 to 5.1)	2914 (1101 to 3990)	4.05 (1.53 to 5.53)	-0.19 (-0.31 to -0.07)
Poland	Diet low in fiber	74 (31 to 169)	0.18 (0.07 to 0.41)	141 (58 to 319)	0.2 (0.08 to 0.44)	0.35 (-0.01 to 0.71)
Poland	Diet low in calcium	612 (309 to 1064)	1.44 (0.72 to 2.5)	1181 (563 to 2126)	1.63 (0.77 to 2.92)	0.33 (-0.08 to 0.74)
Poland	Diet high in red meat	581 (153 to 1087)	1.35 (0.35 to 2.53)	1216 (364 to 2265)	1.72 (0.52 to 3.18)	0.9 (0.82 to 0.99)
Poland	Diet high in processed meat	217 (28 to 372)	0.51 (0.07 to 0.87)	546 (84 to 950)	0.77 (0.12 to 1.34)	1.67 (1.36 to 1.97)
Poland	Diet low in whole grains	1724 (718 to 2187)	4.02 (1.68 to 5.1)	2914 (1101 to 3990)	4.05 (1.53 to 5.53)	-0.19 (-0.31 to -0.07)
Portugal	All dietary risks	919 (637 to 1144)	6.89 (4.82 to 8.56)	1692 (1188 to 2095)	6.59 (4.69 to 8.13)	-0.14 (-0.35 to 0.07)
Portugal	Diet low in milk	486 (180 to 633)	3.63 (1.35 to 4.73)	865 (324 to 1125)	3.35 (1.25 to 4.36)	-0.36 (-0.53 to -0.19)
Portugal	Diet low in fiber	39 (13 to 95)	0.3 (0.1 to 0.74)	82 (27 to 189)	0.3 (0.1 to 0.71)	0.45 (0.2 to 0.69)
Portugal	Diet low in calcium	293 (166 to 473)	2.18 (1.23 to 3.52)	377 (193 to 663)	1.4 (0.72 to 2.47)	-1.38 (-1.48 to -1.28)

Countries	Risk factors	1990		2019		1990-2019
		Number of deaths (95% UI)	ASDR per 100,000 (95% UI)	Number of deaths (95% UI)	ASDR per 100,000 (95% UI)	EAPC of ASDR (95% CI)
Portugal	Diet high in red meat	136 (21 to 302)	1.03 (0.17 to 2.27)	441 (148 to 745)	1.81 (0.64 to 3)	1.81 (1.15 to 2.47)
Portugal	Diet high in processed meat	82 (9 to 140)	0.62 (0.07 to 1.06)	164 (21 to 280)	0.66 (0.09 to 1.12)	0.24 (-0.06 to 0.54)
Portugal	Diet low in whole grains	486 (180 to 633)	3.63 (1.35 to 4.73)	865 (324 to 1125)	3.35 (1.25 to 4.36)	-0.36 (-0.53 to -0.19)
Puerto Rico	All dietary risks	156 (111 to 190)	4.38 (3.14 to 5.32)	341 (216 to 463)	4.61 (2.93 to 6.3)	0.06 (-0.13 to 0.26)
Puerto Rico	Diet low in milk	81 (30 to 103)	2.27 (0.85 to 2.91)	187 (68 to 266)	2.54 (0.93 to 3.64)	0.29 (0.07 to 0.51)
Puerto Rico	Diet low in fiber	11 (4 to 22)	0.31 (0.11 to 0.62)	20 (7 to 43)	0.26 (0.09 to 0.58)	-0.72 (-0.84 to -0.61)
Puerto Rico	Diet low in calcium	65 (43 to 94)	1.84 (1.21 to 2.65)	129 (75 to 203)	1.72 (0.99 to 2.73)	-0.38 (-0.53 to -0.23)
Puerto Rico	Diet high in red meat	12 (1 to 33)	0.35 (0.03 to 0.92)	30 (3 to 84)	0.41 (0.04 to 1.15)	0.5 (0.26 to 0.75)
Puerto Rico	Diet high in processed meat	6 (1 to 10)	0.16 (0.03 to 0.29)	14 (2 to 28)	0.2 (0.03 to 0.39)	0.62 (0.38 to 0.85)
Puerto Rico	Diet low in whole grains	81 (30 to 103)	2.27 (0.85 to 2.91)	187 (68 to 266)	2.54 (0.93 to 3.64)	0.29 (0.07 to 0.51)
Qatar	All dietary risks	3 (2 to 4)	3.95 (2.48 to 5.55)	22 (12 to 31)	4.38 (2.55 to 5.99)	0.81 (0.54 to 1.08)
Qatar	Diet low in milk	2 (1 to 3)	2.6 (1.08 to 3.65)	15 (5 to 21)	2.96 (1.15 to 4.11)	1.01 (0.72 to 1.3)
Qatar	Diet low in fiber	0 (0 to 0)	0.04 (0.03 to 0.08)	0 (0 to 0)	0.04 (0.03 to 0.07)	0.08 (-0.15 to 0.32)
Qatar	Diet low in calcium	1 (0 to 1)	1.11 (0.54 to 1.99)	5 (2 to 8)	1.08 (0.54 to 1.93)	-0.1 (-0.32 to 0.12)
Qatar	Diet high in red meat	0 (0 to 1)	0.4 (0.04 to 1.11)	3 (0 to 7)	0.47 (0.04 to 1.28)	1.29 (0.96 to 1.63)
Qatar	Diet high in processed meat	0 (0 to 0)	0.15 (0.03 to 0.3)	1 (0 to 2)	0.19 (0.04 to 0.36)	1.43 (1.08 to 1.77)
Qatar	Diet low in whole grains	2 (1 to 3)	2.6 (1.08 to 3.65)	15 (5 to 21)	2.96 (1.15 to 4.11)	1.01 (0.72 to 1.3)
Republic of Korea	All dietary risks	1025 (775 to 1255)	3.88 (2.97 to 4.71)	4078 (3036 to 5052)	4.74 (3.52 to 5.88)	0.35 (0.07 to 0.64)

Countries	Risk factors	1990 Number of deaths (95% UI)	1990 ASDR per 100,000 (95% UI)	2019 Number of deaths (95% UI)	2019 ASDR per 100,000 (95% UI)	1990-2019 EAPC of ASDR (95% CI)
Republic of Korea	Diet low in milk	373 (145 to 534)	1.4 (0.55 to 2)	1591 (614 to 2228)	1.85 (0.71 to 2.58)	0.57 (0.29 to 0.86)
Republic of Korea	Diet low in fiber	100 (37 to 187)	0.41 (0.16 to 0.74)	586 (249 to 951)	0.69 (0.3 to 1.12)	1.57 (1.21 to 1.93)
Republic of Korea	Diet low in calcium	527 (386 to 697)	2.02 (1.49 to 2.66)	1688 (1116 to 2422)	1.97 (1.3 to 2.8)	-0.44 (-0.68 to -0.2)
Republic of Korea	Diet high in red meat	86 (7 to 235)	0.31 (0.03 to 0.85)	503 (83 to 1112)	0.58 (0.1 to 1.27)	1.82 (1.29 to 2.36)
Republic of Korea	Diet high in processed meat	76 (9 to 133)	0.26 (0.03 to 0.46)	336 (43 to 580)	0.38 (0.05 to 0.66)	1.1 (0.57 to 1.63)
Republic of Korea	Diet low in whole grains	373 (145 to 534)	1.4 (0.55 to 2)	1591 (614 to 2228)	1.85 (0.71 to 2.58)	0.57 (0.29 to 0.86)
Republic of Moldova	All dietary risks	248 (173 to 307)	5.63 (3.92 to 6.99)	313 (218 to 405)	5.37 (3.75 to 6.93)	0.52 (0.04 to 1.01)
Republic of Moldova	Diet low in milk	131 (49 to 169)	2.96 (1.11 to 3.83)	163 (61 to 218)	2.79 (1.04 to 3.75)	0.55 (0.01 to 1.1)
Republic of Moldova	Diet low in fiber	4 (2 to 11)	0.11 (0.05 to 0.26)	10 (3 to 25)	0.17 (0.06 to 0.42)	2.2 (1.68 to 2.72)
Republic of Moldova	Diet low in calcium	76 (41 to 124)	1.75 (0.96 to 2.84)	100 (55 to 161)	1.71 (0.94 to 2.76)	0.36 (-0.01 to 0.73)
Republic of Moldova	Diet high in red meat	23 (2 to 62)	0.51 (0.04 to 1.39)	26 (2 to 72)	0.45 (0.04 to 1.23)	0.43 (-0.28 to 1.14)
Republic of Moldova	Diet high in processed meat	46 (13 to 69)	1.03 (0.3 to 1.56)	54 (14 to 87)	0.92 (0.24 to 1.5)	0.4 (-0.18 to 0.98)
Republic of Moldova	Diet low in whole grains	131 (49 to 169)	2.96 (1.11 to 3.83)	163 (61 to 218)	2.79 (1.04 to 3.75)	0.55 (0.01 to 1.1)
Romania	All dietary risks	1023 (689 to 1296)	3.71 (2.5 to 4.7)	2107 (1366 to 2788)	5.64 (3.68 to 7.47)	1.26 (1.05 to 1.48)
Romania	Diet low in milk	597 (218 to 770)	2.16 (0.8 to 2.78)	1187 (437 to 1649)	3.16 (1.17 to 4.37)	1.04 (0.84 to 1.25)
Romania	Diet low in fiber	43 (15 to 106)	0.16 (0.05 to 0.39)	49 (20 to 117)	0.13 (0.05 to 0.31)	-1.5 (-1.85 to -1.15)
Romania	Diet low in calcium	246 (119 to 435)	0.9 (0.44 to 1.6)	206 (94 to 359)	0.53 (0.24 to 0.92)	-2.62 (-3.01 to -2.23)
Romania	Diet high in red meat	135 (16 to 331)	0.49 (0.06 to 1.2)	508 (123 to 968)	1.4 (0.35 to 2.62)	4.1 (3.85 to 4.34)

Countries	Risk factors	1990 Number of deaths (95% UI)	1990 ASDR per 100,000 (95% UI)	2019 Number of deaths (95% UI)	2019 ASDR per 100,000 (95% UI)	1990-2019 EAPC of ASDR (95% CI)
Romania	Diet high in processed meat	116 (16 to 195)	0.42 (0.06 to 0.71)	384 (96 to 628)	1.04 (0.27 to 1.71)	3.56 (3.31 to 3.81)
Romania	Diet low in whole grains	597 (218 to 770)	2.16 (0.8 to 2.78)	1187 (437 to 1649)	3.16 (1.17 to 4.37)	1.04 (0.84 to 1.25)
Russian Federation	All dietary risks	11290 (8473 to 13664)	6.34 (4.76 to 7.65)	13572 (9453 to 17522)	5.76 (4.02 to 7.42)	-0.87 (-1.2 to -0.55)
Russian Federation	Diet low in milk	5313 (2093 to 6762)	2.99 (1.17 to 3.8)	7217 (2748 to 9557)	3.06 (1.17 to 4.05)	-0.23 (-0.49 to 0.04)
Russian Federation	Diet low in fiber	455 (165 to 1014)	0.26 (0.1 to 0.58)	676 (254 to 1462)	0.29 (0.11 to 0.62)	-0.7 (-1.35 to -0.04)
Russian Federation	Diet low in calcium	2821 (1603 to 4530)	1.6 (0.91 to 2.56)	3070 (1589 to 5314)	1.3 (0.67 to 2.25)	-1.61 (-2.08 to -1.14)
Russian Federation	Diet high in red meat	2102 (615 to 3771)	1.17 (0.34 to 2.1)	1906 (341 to 4184)	0.81 (0.15 to 1.78)	-2.23 (-2.6 to -1.86)
Russian Federation	Diet high in processed meat	2628 (912 to 4926)	1.46 (0.51 to 2.73)	2529 (789 to 3917)	1.07 (0.33 to 1.66)	-1.58 (-1.95 to -1.21)
Russian Federation	Diet low in whole grains	5313 (2093 to 6762)	2.99 (1.17 to 3.8)	7217 (2748 to 9557)	3.06 (1.17 to 4.05)	-0.23 (-0.49 to 0.04)
Rwanda	All dietary risks	88 (60 to 116)	3.25 (2.28 to 4.22)	166 (117 to 219)	3.07 (2.19 to 3.95)	-0.63 (-0.83 to -0.43)
Rwanda	Diet low in milk	39 (15 to 57)	1.43 (0.54 to 2.04)	77 (28 to 112)	1.41 (0.53 to 2.02)	-0.4 (-0.61 to -0.19)
Rwanda	Diet low in fiber	1 (0 to 1)	0.02 (0.01 to 0.02)	1 (1 to 1)	0.02 (0.02 to 0.02)	-0.45 (-0.64 to -0.26)
Rwanda	Diet low in calcium	54 (37 to 72)	1.99 (1.4 to 2.65)	93 (67 to 127)	1.76 (1.29 to 2.31)	-0.97 (-1.17 to -0.77)
Rwanda	Diet high in red meat	3 (0 to 7)	0.1 (0.02 to 0.25)	7 (1 to 19)	0.13 (0.02 to 0.33)	0.83 (0.57 to 1.09)
Rwanda	Diet high in processed meat	4 (0 to 7)	0.12 (0.02 to 0.25)	8 (1 to 15)	0.13 (0.02 to 0.25)	-0.04 (-0.29 to 0.22)
Rwanda	Diet low in whole grains	39 (15 to 57)	1.43 (0.54 to 2.04)	77 (28 to 112)	1.41 (0.53 to 2.02)	-0.4 (-0.61 to -0.19)
Saint Kitts and Nevis	All dietary risks	3 (2 to 3)	7.5 (5.9 to 8.8)	4 (3 to 5)	6.94 (5.24 to 8.51)	-0.08 (-0.23 to 0.07)
Saint Kitts and Nevis	Diet low in milk	1 (0 to 2)	3.29 (1.29 to 4.26)	2 (1 to 3)	3.17 (1.21 to 4.22)	0.03 (-0.13 to 0.18)

Countries	Risk factors	1990 Number of deaths (95% UI)	1990 ASDR per 100,000 (95% UI)	2019 Number of deaths (95% UI)	2019 ASDR per 100,000 (95% UI)	1990-2019 EAPC of ASDR (95% CI)
Saint Kitts and Nevis	Diet low in fiber	0 (0 to 1)	1.27 (0.63 to 1.81)	1 (0 to 1)	1.21 (0.62 to 1.78)	0.1 (-0.03 to 0.24)
Saint Kitts and Nevis	Diet low in calcium	1 (1 to 2)	3.56 (2.76 to 4.56)	2 (1 to 2)	3.03 (2.13 to 4.07)	-0.31 (-0.46 to -0.16)
Saint Kitts and Nevis	Diet high in red meat	0 (0 to 0)	0.47 (0.04 to 1.29)	0 (0 to 1)	0.48 (0.04 to 1.32)	0.02 (-0.14 to 0.19)
Saint Kitts and Nevis	Diet high in processed meat	0 (0 to 0)	0.21 (0.04 to 0.37)	0 (0 to 0)	0.2 (0.04 to 0.37)	-0.03 (-0.22 to 0.15)
Saint Kitts and Nevis	Diet low in whole grains	1 (0 to 2)	3.29 (1.29 to 4.26)	2 (1 to 3)	3.17 (1.21 to 4.22)	0.03 (-0.13 to 0.18)
Saint Lucia	All dietary risks	4 (3 to 4)	4.53 (3.41 to 5.42)	9 (6 to 11)	4.28 (3.07 to 5.43)	-0.5 (-0.78 to -0.22)
Saint Lucia	Diet low in milk	2 (1 to 2)	2.38 (1.08 to 2.99)	5 (2 to 6)	2.22 (0.92 to 3.01)	-0.63 (-0.92 to -0.35)
Saint Lucia	Diet low in fiber	0 (0 to 0)	0.29 (0.1 to 0.6)	1 (0 to 2)	0.42 (0.16 to 0.76)	1.33 (1.16 to 1.5)
Saint Lucia	Diet low in calcium	2 (1 to 2)	2.03 (1.45 to 2.68)	4 (2 to 5)	1.76 (1.19 to 2.5)	-0.64 (-0.97 to -0.3)
Saint Lucia	Diet high in red meat	0 (0 to 1)	0.38 (0.03 to 1.01)	1 (0 to 2)	0.37 (0.03 to 1)	-0.8 (-1.11 to -0.49)
Saint Lucia	Diet high in processed meat	0 (0 to 0)	0.13 (0.03 to 0.23)	0 (0 to 1)	0.13 (0.03 to 0.25)	-0.34 (-0.63 to -0.06)
Saint Lucia	Diet low in whole grains	2 (1 to 2)	2.38 (1.08 to 2.99)	5 (2 to 6)	2.22 (0.92 to 3.01)	-0.63 (-0.92 to -0.35)
Saint Vincent and the Grenadines	All dietary risks	3 (3 to 4)	4.78 (3.8 to 5.54)	6 (5 to 8)	4.93 (3.54 to 6.1)	-0.08 (-0.33 to 0.17)
Saint Vincent and the Grenadines	Diet low in milk	2 (1 to 2)	2.21 (0.94 to 2.79)	3 (1 to 4)	2.46 (0.94 to 3.29)	0.27 (0.04 to 0.5)
Saint Vincent and the Grenadines	Diet low in fiber	0 (0 to 0)	0.33 (0.11 to 0.67)	0 (0 to 1)	0.33 (0.11 to 0.66)	-0.54 (-0.76 to -0.31)
Saint Vincent and the Grenadines	Diet low in calcium	2 (1 to 2)	2.64 (2.12 to 3.28)	3 (2 to 4)	2.31 (1.61 to 3.17)	-0.81 (-1.12 to -0.5)
Saint Vincent and the Grenadines	Diet high in red meat	0 (0 to 0)	0.23 (0.03 to 0.59)	0 (0 to 1)	0.33 (0.03 to 0.91)	1.29 (1.15 to 1.43)
Saint Vincent and the Grenadines	Diet high in processed meat	0 (0 to 0)	0.12 (0.03 to 0.22)	0 (0 to 0)	0.17 (0.03 to 0.31)	1.2 (1.01 to 1.38)

Countries	Risk factors	1990 Number of deaths (95% UI)	1990 ASDR per 100,000 (95% UI)	2019 Number of deaths (95% UI)	2019 ASDR per 100,000 (95% UI)	1990-2019 EAPC of ASDR (95% CI)
Saint Vincent and the Grenadines	Diet low in whole grains	2 (1 to 2)	2.21 (0.94 to 2.79)	3 (1 to 4)	2.46 (0.94 to 3.29)	0.27 (0.04 to 0.5)
Samoa	All dietary risks	3 (3 to 4)	4.02 (3.08 to 5.03)	5 (4 to 7)	3.72 (2.67 to 4.81)	-0.4 (-0.46 to -0.34)
Samoa	Diet low in milk	1 (1 to 2)	1.8 (0.67 to 2.46)	2 (1 to 3)	1.76 (0.66 to 2.49)	-0.1 (-0.15 to -0.05)
Samoa	Diet low in fiber	0 (0 to 0)	0.02 (0.02 to 0.03)	0 (0 to 0)	0.02 (0.02 to 0.03)	-0.27 (-0.32 to -0.22)
Samoa	Diet low in calcium	2 (1 to 3)	2.36 (1.83 to 3.06)	3 (2 to 4)	2.03 (1.48 to 2.69)	-0.74 (-0.84 to -0.65)
Samoa	Diet high in red meat	0 (0 to 1)	0.31 (0.03 to 0.86)	0 (0 to 1)	0.31 (0.03 to 0.83)	-0.05 (-0.09 to -0.01)
Samoa	Diet high in processed meat	0 (0 to 0)	0.09 (0.03 to 0.16)	0 (0 to 0)	0.09 (0.02 to 0.18)	0.2 (0.15 to 0.25)
Samoa	Diet low in whole grains	1 (1 to 2)	1.8 (0.67 to 2.46)	2 (1 to 3)	1.76 (0.66 to 2.49)	-0.1 (-0.15 to -0.05)
San Marino	All dietary risks	2 (2 to 3)	7.34 (5.07 to 9.38)	5 (3 to 7)	6.6 (3.89 to 9.95)	-0.18 (-0.27 to -0.09)
San Marino	Diet low in milk	1 (1 to 2)	4.03 (1.55 to 5.39)	3 (1 to 4)	3.63 (1.32 to 5.7)	-0.16 (-0.25 to -0.07)
San Marino	Diet low in fiber	0 (0 to 0)	0.36 (0.12 to 0.84)	0 (0 to 1)	0.33 (0.1 to 0.79)	-0.28 (-0.68 to 0.13)
San Marino	Diet low in calcium	0 (0 to 0)	0.79 (0.3 to 1.38)	1 (0 to 1)	0.74 (0.28 to 1.38)	-0.47 (-1.25 to 0.32)
San Marino	Diet high in red meat	1 (0 to 1)	1.71 (0.49 to 3.12)	1 (0 to 2)	1.5 (0.38 to 3.06)	-0.18 (-0.33 to -0.03)
San Marino	Diet high in processed meat	0 (0 to 1)	1.38 (0.42 to 2.19)	1 (0 to 2)	1.24 (0.37 to 2.23)	-0.14 (-0.25 to -0.03)
San Marino	Diet low in whole grains	1 (1 to 2)	4.03 (1.55 to 5.39)	3 (1 to 4)	3.63 (1.32 to 5.7)	-0.16 (-0.25 to -0.07)
Sao Tome and Principe	All dietary risks	2 (2 to 3)	4.01 (3.07 to 4.87)	5 (4 to 8)	6 (4.06 to 8.41)	1.45 (1.39 to 1.52)
Sao Tome and Principe	Diet low in milk	1 (0 to 1)	1.66 (0.63 to 2.23)	2 (1 to 4)	2.58 (0.9 to 3.93)	1.63 (1.57 to 1.68)
Sao Tome and Principe	Diet low in fiber	0 (0 to 0)	0.03 (0.02 to 0.04)	0 (0 to 0)	0.03 (0.02 to 0.05)	1.25 (1.12 to 1.38)

Countries	Risk factors	1990 Number of deaths (95% UI)	1990 ASDR per 100,000 (95% UI)	2019 Number of deaths (95% UI)	2019 ASDR per 100,000 (95% UI)	1990-2019 EAPC of ASDR (95% CI)
Sao Tome and Principe	Diet low in calcium	2 (1 to 2)	2.59 (1.95 to 3.22)	3 (2 to 5)	3.71 (2.53 to 5.33)	1.29 (1.21 to 1.36)
Sao Tome and Principe	Diet high in red meat	0 (0 to 0)	0.09 (0.03 to 0.18)	0 (0 to 0)	0.14 (0.04 to 0.34)	1.94 (1.81 to 2.08)
Sao Tome and Principe	Diet high in processed meat	0 (0 to 0)	0.23 (0.03 to 0.41)	0 (0 to 1)	0.37 (0.04 to 0.75)	1.74 (1.68 to 1.79)
Sao Tome and Principe	Diet low in whole grains	1 (0 to 1)	1.66 (0.63 to 2.23)	2 (1 to 4)	2.58 (0.9 to 3.93)	1.63 (1.57 to 1.68)
Saudi Arabia	All dietary risks	118 (74 to 169)	2.21 (1.38 to 3.1)	479 (301 to 664)	2.88 (1.83 to 3.85)	0.8 (0.57 to 1.02)
Saudi Arabia	Diet low in milk	62 (23 to 93)	1.15 (0.44 to 1.72)	274 (102 to 390)	1.63 (0.6 to 2.25)	1.11 (0.89 to 1.34)
Saudi Arabia	Diet low in fiber	3 (1 to 8)	0.06 (0.02 to 0.15)	11 (4 to 25)	0.06 (0.03 to 0.15)	0.67 (0.52 to 0.82)
Saudi Arabia	Diet low in calcium	56 (32 to 86)	1.06 (0.61 to 1.62)	190 (111 to 296)	1.19 (0.73 to 1.83)	0.23 (-0.02 to 0.48)
Saudi Arabia	Diet high in red meat	8 (1 to 24)	0.14 (0.01 to 0.43)	36 (3 to 99)	0.2 (0.02 to 0.54)	1.01 (0.87 to 1.15)
Saudi Arabia	Diet high in processed meat	3 (1 to 6)	0.06 (0.01 to 0.11)	18 (3 to 35)	0.1 (0.02 to 0.18)	1.82 (1.65 to 1.99)
Saudi Arabia	Diet low in whole grains	62 (23 to 93)	1.15 (0.44 to 1.72)	274 (102 to 390)	1.63 (0.6 to 2.25)	1.11 (0.89 to 1.34)
Senegal	All dietary risks	93 (71 to 116)	3.19 (2.45 to 3.93)	224 (165 to 290)	3.36 (2.52 to 4.29)	0.26 (0.11 to 0.42)
Senegal	Diet low in milk	36 (13 to 49)	1.23 (0.45 to 1.66)	89 (33 to 128)	1.33 (0.49 to 1.9)	0.34 (0.19 to 0.5)
Senegal	Diet low in fiber	5 (2 to 11)	0.18 (0.06 to 0.38)	3 (1 to 6)	0.05 (0.02 to 0.1)	-5.22 (-5.85 to -4.58)
Senegal	Diet low in calcium	57 (43 to 73)	1.98 (1.5 to 2.51)	137 (98 to 180)	2.07 (1.52 to 2.68)	0.23 (0.08 to 0.37)
Senegal	Diet high in red meat	4 (0 to 12)	0.15 (0.02 to 0.39)	12 (1 to 32)	0.17 (0.02 to 0.46)	0.69 (0.48 to 0.9)
Senegal	Diet high in processed meat	5 (1 to 10)	0.18 (0.02 to 0.33)	15 (2 to 28)	0.21 (0.03 to 0.4)	0.9 (0.68 to 1.12)
Senegal	Diet low in whole grains	36 (13 to 49)	1.23 (0.45 to 1.66)	89 (33 to 128)	1.33 (0.49 to 1.9)	0.34 (0.19 to 0.5)

Countries	Risk factors	1990 Number of deaths (95% UI)	1990 ASDR per 100,000 (95% UI)	2019 Number of deaths (95% UI)	2019 ASDR per 100,000 (95% UI)	1990-2019 EAPC of ASDR (95% CI)
Serbia	All dietary risks	694 (466 to 890)	6.45 (4.34 to 8.31)	1213 (778 to 1637)	7.68 (4.96 to 10.33)	0.71 (0.55 to 0.88)
Serbia	Diet low in milk	386 (149 to 510)	3.58 (1.37 to 4.78)	682 (259 to 948)	4.3 (1.62 to 5.97)	0.82 (0.67 to 0.98)
Serbia	Diet low in fiber	38 (13 to 88)	0.36 (0.12 to 0.84)	63 (21 to 144)	0.41 (0.14 to 0.93)	0.05 (-0.31 to 0.42)
Serbia	Diet low in calcium	208 (108 to 347)	1.97 (1.03 to 3.25)	367 (194 to 613)	2.32 (1.22 to 3.86)	0.47 (0.24 to 0.71)
Serbia	Diet high in red meat	69 (6 to 186)	0.64 (0.05 to 1.72)	120 (11 to 329)	0.77 (0.07 to 2.11)	1.03 (0.82 to 1.24)
Serbia	Diet high in processed meat	76 (11 to 132)	0.69 (0.1 to 1.2)	125 (17 to 220)	0.8 (0.11 to 1.42)	0.73 (0.6 to 0.86)
Serbia	Diet low in whole grains	386 (149 to 510)	3.58 (1.37 to 4.78)	682 (259 to 948)	4.3 (1.62 to 5.97)	0.82 (0.67 to 0.98)
Seychelles	All dietary risks	3 (3 to 4)	5.81 (4.57 to 6.99)	8 (6 to 10)	8.14 (6.09 to 10.13)	0.85 (0.64 to 1.07)
Seychelles	Diet low in milk	1 (0 to 2)	2.2 (0.83 to 3.11)	4 (1 to 5)	3.46 (1.29 to 4.95)	1.22 (0.96 to 1.47)
Seychelles	Diet low in fiber	0 (0 to 1)	0.6 (0.23 to 1.09)	1 (0 to 1)	0.53 (0.17 to 1.14)	-0.66 (-0.76 to -0.57)
Seychelles	Diet low in calcium	2 (2 to 2)	3.41 (2.7 to 4.29)	5 (3 to 6)	4.49 (3.24 to 5.89)	0.66 (0.46 to 0.87)
Seychelles	Diet high in red meat	0 (0 to 0)	0.26 (0.04 to 0.66)	0 (0 to 1)	0.44 (0.06 to 1.11)	1.41 (1.16 to 1.66)
Seychelles	Diet high in processed meat	0 (0 to 0)	0.1 (0.06 to 0.15)	0 (0 to 0)	0.17 (0.09 to 0.28)	1.66 (1.42 to 1.89)
Seychelles	Diet low in whole grains	1 (0 to 2)	2.2 (0.83 to 3.11)	4 (1 to 5)	3.46 (1.29 to 4.95)	1.22 (0.96 to 1.47)
Sierra Leone	All dietary risks	49 (37 to 62)	2.75 (2.1 to 3.43)	94 (67 to 126)	2.95 (2.15 to 3.86)	0.44 (0.31 to 0.58)
Sierra Leone	Diet low in milk	16 (6 to 23)	0.9 (0.33 to 1.3)	32 (12 to 48)	1.01 (0.37 to 1.5)	0.61 (0.47 to 0.74)
Sierra Leone	Diet low in fiber	8 (4 to 12)	0.45 (0.22 to 0.66)	10 (4 to 18)	0.33 (0.14 to 0.56)	-1.13 (-1.24 to -1.03)
Sierra Leone	Diet low in calcium	31 (22 to 40)	1.73 (1.26 to 2.22)	60 (42 to 81)	1.89 (1.34 to 2.53)	0.51 (0.38 to 0.65)

Countries	Risk factors	1990 Number of deaths (95% UI)	1990 ASDR per 100,000 (95% UI)	2019 Number of deaths (95% UI)	2019 ASDR per 100,000 (95% UI)	1990-2019 EAPC of ASDR (95% CI)
Sierra Leone	Diet high in red meat	1 (0 to 2)	0.06 (0.01 to 0.13)	3 (0 to 6)	0.08 (0.01 to 0.19)	1.47 (1.21 to 1.73)
Sierra Leone	Diet high in processed meat	2 (0 to 4)	0.12 (0.01 to 0.23)	5 (1 to 10)	0.15 (0.02 to 0.29)	0.89 (0.7 to 1.07)
Sierra Leone	Diet low in whole grains	16 (6 to 23)	0.9 (0.33 to 1.3)	32 (12 to 48)	1.01 (0.37 to 1.5)	0.61 (0.47 to 0.74)
Singapore	All dietary risks	160 (118 to 199)	7.98 (5.91 to 9.86)	332 (233 to 424)	4.45 (3.12 to 5.67)	-2.25 (-2.36 to -2.15)
Singapore	Diet low in milk	69 (26 to 95)	3.39 (1.26 to 4.69)	155 (59 to 212)	2.07 (0.78 to 2.84)	-1.99 (-2.12 to -1.87)
Singapore	Diet low in fiber	16 (6 to 30)	0.82 (0.32 to 1.52)	28 (10 to 55)	0.38 (0.14 to 0.75)	-2.8 (-2.91 to -2.7)
Singapore	Diet low in calcium	71 (48 to 99)	3.6 (2.44 to 4.97)	116 (66 to 180)	1.57 (0.9 to 2.43)	-3.08 (-3.18 to -2.98)
Singapore	Diet high in red meat	18 (2 to 44)	0.86 (0.09 to 2.14)	52 (9 to 114)	0.68 (0.12 to 1.52)	-1.03 (-1.12 to -0.93)
Singapore	Diet high in processed meat	8 (1 to 14)	0.38 (0.05 to 0.68)	25 (3 to 44)	0.33 (0.04 to 0.58)	-0.61 (-0.7 to -0.53)
Singapore	Diet low in whole grains	69 (26 to 95)	3.39 (1.26 to 4.69)	155 (59 to 212)	2.07 (0.78 to 2.84)	-1.99 (-2.12 to -1.87)
Slovakia	All dietary risks	476 (347 to 584)	8.03 (5.87 to 9.84)	851 (575 to 1131)	9.21 (6.21 to 12.24)	0.49 (0.31 to 0.67)
Slovakia	Diet low in milk	230 (86 to 300)	3.88 (1.45 to 5.04)	405 (150 to 566)	4.38 (1.63 to 6.11)	0.39 (0.21 to 0.57)
Slovakia	Diet low in fiber	35 (12 to 72)	0.6 (0.21 to 1.22)	78 (27 to 150)	0.84 (0.29 to 1.64)	1.36 (0.96 to 1.77)
Slovakia	Diet low in calcium	172 (108 to 255)	2.9 (1.82 to 4.29)	273 (157 to 428)	2.96 (1.7 to 4.62)	0.04 (-0.19 to 0.28)
Slovakia	Diet high in red meat	56 (7 to 133)	0.94 (0.12 to 2.23)	110 (18 to 255)	1.2 (0.19 to 2.76)	0.88 (0.64 to 1.12)
Slovakia	Diet high in processed meat	52 (9 to 87)	0.88 (0.15 to 1.46)	119 (28 to 202)	1.29 (0.31 to 2.18)	1.62 (1.41 to 1.83)
Slovakia	Diet low in whole grains	230 (86 to 300)	3.88 (1.45 to 5.04)	405 (150 to 566)	4.38 (1.63 to 6.11)	0.39 (0.21 to 0.57)
Slovenia	All dietary risks	167 (111 to 227)	6.89 (4.59 to 9.35)	273 (184 to 371)	5.93 (4.01 to 8.09)	-0.74 (-1.03 to -0.45)

Countries	Risk factors	1990 Number of deaths (95% UI)	1990 ASDR per 100,000 (95% UI)	2019 Number of deaths (95% UI)	2019 ASDR per 100,000 (95% UI)	1990-2019 EAPC of ASDR (95% CI)
Slovenia	Diet low in milk	82 (30 to 115)	3.37 (1.23 to 4.78)	140 (51 to 201)	3.05 (1.11 to 4.4)	-0.55 (-0.84 to -0.26)
Slovenia	Diet low in fiber	9 (3 to 22)	0.39 (0.12 to 0.91)	14 (5 to 32)	0.3 (0.1 to 0.67)	-1.25 (-1.41 to -1.09)
Slovenia	Diet low in calcium	34 (15 to 62)	1.41 (0.64 to 2.56)	45 (20 to 81)	0.94 (0.42 to 1.74)	-1.77 (-1.95 to -1.6)
Slovenia	Diet high in red meat	32 (7 to 64)	1.31 (0.3 to 2.64)	49 (9 to 108)	1.1 (0.21 to 2.37)	-0.83 (-1.25 to -0.41)
Slovenia	Diet high in processed meat	33 (11 to 54)	1.37 (0.45 to 2.24)	59 (19 to 97)	1.3 (0.44 to 2.13)	-0.36 (-0.72 to 0)
Slovenia	Diet low in whole grains	82 (30 to 115)	3.37 (1.23 to 4.78)	140 (51 to 201)	3.05 (1.11 to 4.4)	-0.55 (-0.84 to -0.26)
Solomon Islands	All dietary risks	4 (3 to 6)	3.19 (2.12 to 4.53)	11 (7 to 16)	3.78 (2.59 to 5.11)	0.48 (0.39 to 0.57)
Solomon Islands	Diet low in milk	2 (1 to 3)	1.4 (0.55 to 2.16)	5 (2 to 7)	1.65 (0.6 to 2.39)	0.45 (0.35 to 0.55)
Solomon Islands	Diet low in fiber	0 (0 to 0)	0.02 (0.01 to 0.04)	0 (0 to 0)	0.03 (0.02 to 0.04)	0.47 (0.39 to 0.56)
Solomon Islands	Diet low in calcium	3 (2 to 4)	1.96 (1.29 to 2.84)	7 (4 to 10)	2.31 (1.56 to 3.16)	0.47 (0.39 to 0.55)
Solomon Islands	Diet high in red meat	0 (0 to 1)	0.16 (0.02 to 0.48)	1 (0 to 2)	0.18 (0.02 to 0.51)	0.36 (0.16 to 0.55)
Solomon Islands	Diet high in processed meat	0 (0 to 0)	0.06 (0.02 to 0.12)	0 (0 to 1)	0.08 (0.03 to 0.15)	0.93 (0.83 to 1.02)
Solomon Islands	Diet low in whole grains	2 (1 to 3)	1.4 (0.55 to 2.16)	5 (2 to 7)	1.65 (0.6 to 2.39)	0.45 (0.35 to 0.55)
Somalia	All dietary risks	54 (37 to 84)	2.3 (1.59 to 3.58)	136 (83 to 261)	2.22 (1.35 to 4.22)	0.04 (-0.03 to 0.1)
Somalia	Diet low in milk	20 (7 to 32)	0.85 (0.31 to 1.36)	51 (17 to 99)	0.82 (0.28 to 1.61)	0.09 (0.02 to 0.16)
Somalia	Diet low in fiber	6 (2 to 10)	0.24 (0.09 to 0.44)	17 (6 to 35)	0.28 (0.1 to 0.57)	0.43 (0.23 to 0.62)
Somalia	Diet low in calcium	33 (22 to 50)	1.39 (0.95 to 2.17)	81 (49 to 156)	1.33 (0.8 to 2.55)	0.01 (-0.07 to 0.08)
Somalia	Diet high in red meat	4 (0 to 12)	0.16 (0.01 to 0.47)	8 (1 to 29)	0.13 (0.01 to 0.45)	-0.45 (-0.54 to -0.36)

Countries	Risk factors	1990 Number of deaths (95% UI)	1990 ASDR per 100,000 (95% UI)	2019 Number of deaths (95% UI)	2019 ASDR per 100,000 (95% UI)	1990-2019 EAPC of ASDR (95% CI)
Somalia	Diet high in processed meat	2 (0 to 4)	0.07 (0.01 to 0.16)	4 (1 to 11)	0.07 (0.01 to 0.17)	-0.01 (-0.15 to 0.13)
Somalia	Diet low in whole grains	20 (7 to 32)	0.85 (0.31 to 1.36)	51 (17 to 99)	0.82 (0.28 to 1.61)	0.09 (0.02 to 0.16)
South Africa	All dietary risks	736 (559 to 952)	3.78 (2.85 to 4.96)	1693 (1327 to 2070)	4.12 (3.2 to 5.02)	0.3 (0.06 to 0.54)
South Africa	Diet low in milk	243 (92 to 368)	1.24 (0.47 to 1.91)	572 (214 to 826)	1.39 (0.52 to 2)	0.41 (0.16 to 0.65)
South Africa	Diet low in fiber	16 (7 to 35)	0.08 (0.03 to 0.18)	41 (17 to 90)	0.1 (0.04 to 0.22)	0.66 (0.44 to 0.88)
South Africa	Diet low in calcium	476 (361 to 621)	2.46 (1.84 to 3.25)	1063 (851 to 1321)	2.6 (2.08 to 3.23)	0.14 (-0.11 to 0.4)
South Africa	Diet high in red meat	70 (9 to 180)	0.35 (0.04 to 0.92)	178 (27 to 419)	0.42 (0.06 to 1)	0.9 (0.71 to 1.08)
South Africa	Diet high in processed meat	26 (5 to 49)	0.13 (0.02 to 0.25)	70 (11 to 125)	0.16 (0.03 to 0.29)	1 (0.8 to 1.2)
South Africa	Diet low in whole grains	243 (92 to 368)	1.24 (0.47 to 1.91)	572 (214 to 826)	1.39 (0.52 to 2)	0.41 (0.16 to 0.65)
South Sudan	All dietary risks	81 (46 to 135)	3.67 (2.12 to 6.09)	121 (76 to 187)	3.53 (2.24 to 5.4)	-0.12 (-0.18 to -0.05)
South Sudan	Diet low in milk	33 (12 to 60)	1.49 (0.52 to 2.7)	50 (19 to 83)	1.46 (0.54 to 2.39)	-0.03 (-0.1 to 0.04)
South Sudan	Diet low in fiber	1 (0 to 2)	0.03 (0.02 to 0.08)	1 (1 to 2)	0.03 (0.02 to 0.06)	-0.4 (-0.46 to -0.35)
South Sudan	Diet low in calcium	49 (27 to 84)	2.23 (1.27 to 3.81)	72 (44 to 111)	2.11 (1.32 to 3.22)	-0.21 (-0.27 to -0.16)
South Sudan	Diet high in red meat	5 (0 to 15)	0.21 (0.02 to 0.67)	7 (1 to 22)	0.21 (0.02 to 0.61)	0.05 (-0.05 to 0.15)
South Sudan	Diet high in processed meat	4 (0 to 9)	0.17 (0.02 to 0.41)	6 (1 to 14)	0.17 (0.02 to 0.36)	0.09 (-0.02 to 0.2)
South Sudan	Diet low in whole grains	33 (12 to 60)	1.49 (0.52 to 2.7)	50 (19 to 83)	1.46 (0.54 to 2.39)	-0.03 (-0.1 to 0.04)
Spain	All dietary risks	3563 (2644 to 4334)	6.57 (4.89 to 7.98)	6817 (4968 to 8519)	6.43 (4.71 to 8.02)	-0.05 (-0.11 to 0)

Countries	Risk factors	1990 Number of deaths (95% UI)	1990 ASDR per 100,000 (95% UI)	2019 Number of deaths (95% UI)	2019 ASDR per 100,000 (95% UI)	1990-2019 EAPC of ASDR (95% CI)
Spain	Diet low in milk	1730 (656 to 2218)	3.19 (1.21 to 4.08)	3228 (1228 to 4234)	3.03 (1.15 to 3.95)	-0.18 (-0.23 to -0.12)
Spain	Diet low in fiber	170 (58 to 389)	0.32 (0.11 to 0.72)	438 (154 to 929)	0.4 (0.14 to 0.85)	1.25 (1.06 to 1.45)
Spain	Diet low in calcium	861 (453 to 1475)	1.58 (0.83 to 2.72)	1810 (974 to 3015)	1.67 (0.89 to 2.81)	0.55 (0.42 to 0.69)
Spain	Diet high in red meat	687 (173 to 1275)	1.28 (0.33 to 2.37)	1214 (275 to 2359)	1.2 (0.29 to 2.26)	-0.73 (-0.88 to -0.58)
Spain	Diet high in processed meat	675 (231 to 1047)	1.24 (0.43 to 1.93)	1186 (335 to 1803)	1.14 (0.35 to 1.73)	-0.26 (-0.37 to -0.16)
Spain	Diet low in whole grains	1730 (656 to 2218)	3.19 (1.21 to 4.08)	3228 (1228 to 4234)	3.03 (1.15 to 3.95)	-0.18 (-0.23 to -0.12)
Sri Lanka	All dietary risks	183 (148 to 217)	1.89 (1.53 to 2.23)	529 (370 to 717)	2.25 (1.59 to 3)	0.99 (0.86 to 1.12)
Sri Lanka	Diet low in milk	71 (27 to 96)	0.72 (0.27 to 0.98)	213 (78 to 318)	0.9 (0.33 to 1.35)	1.11 (0.99 to 1.23)
Sri Lanka	Diet low in fiber	9 (3 to 21)	0.1 (0.04 to 0.22)	18 (6 to 42)	0.08 (0.03 to 0.19)	-0.45 (-0.78 to -0.13)
Sri Lanka	Diet low in calcium	120 (96 to 148)	1.24 (0.98 to 1.52)	337 (234 to 468)	1.44 (1.01 to 1.98)	0.9 (0.76 to 1.04)
Sri Lanka	Diet high in red meat	3 (1 to 7)	0.03 (0.01 to 0.07)	11 (3 to 24)	0.05 (0.01 to 0.1)	1.4 (1.29 to 1.51)
Sri Lanka	Diet high in processed meat	3 (2 to 5)	0.03 (0.02 to 0.04)	11 (4 to 18)	0.04 (0.02 to 0.08)	1.78 (1.66 to 1.9)
Sri Lanka	Diet low in whole grains	71 (27 to 96)	0.72 (0.27 to 0.98)	213 (78 to 318)	0.9 (0.33 to 1.35)	1.11 (0.99 to 1.23)
Sudan	All dietary risks	188 (134 to 286)	2.13 (1.55 to 3.28)	503 (345 to 766)	2.87 (1.98 to 4.41)	1.11 (1.03 to 1.19)
Sudan	Diet low in milk	92 (45 to 144)	1.04 (0.5 to 1.63)	258 (121 to 412)	1.47 (0.69 to 2.34)	1.29 (1.2 to 1.39)
Sudan	Diet low in fiber	12 (5 to 25)	0.14 (0.05 to 0.29)	26 (9 to 55)	0.15 (0.05 to 0.31)	0.09 (0.03 to 0.15)
Sudan	Diet low in calcium	102 (71 to 156)	1.16 (0.83 to 1.79)	261 (175 to 405)	1.5 (1.03 to 2.31)	0.92 (0.85 to 0.99)
Sudan	Diet high in red meat	9 (1 to 25)	0.1 (0.01 to 0.28)	26 (3 to 77)	0.14 (0.01 to 0.42)	1.41 (1.29 to 1.54)

Countries	Risk factors	1990		2019		1990-2019
		Number of deaths (95% UI)	ASDR per 100,000 (95% UI)	Number of deaths (95% UI)	ASDR per 100,000 (95% UI)	EAPC of ASDR (95% CI)
Sudan	Diet high in processed meat	4 (1 to 7)	0.04 (0.01 to 0.07)	11 (3 to 22)	0.06 (0.02 to 0.12)	1.6 (1.48 to 1.72)
Sudan	Diet low in whole grains	92 (45 to 144)	1.04 (0.5 to 1.63)	258 (121 to 412)	1.47 (0.69 to 2.34)	1.29 (1.2 to 1.39)
Suriname	All dietary risks	11 (9 to 13)	4.52 (3.57 to 5.39)	35 (26 to 44)	6.03 (4.54 to 7.64)	1.05 (0.81 to 1.3)
Suriname	Diet low in milk	4 (2 to 6)	1.8 (0.67 to 2.46)	14 (5 to 19)	2.41 (0.89 to 3.35)	1.09 (0.85 to 1.34)
Suriname	Diet low in fiber	2 (1 to 3)	0.75 (0.34 to 1.12)	5 (2 to 8)	0.93 (0.41 to 1.48)	0.73 (0.47 to 0.99)
Suriname	Diet low in calcium	6 (4 to 7)	2.32 (1.75 to 2.99)	18 (13 to 24)	3.15 (2.29 to 4.14)	1.08 (0.81 to 1.34)
Suriname	Diet high in red meat	1 (0 to 1)	0.21 (0.03 to 0.52)	2 (0 to 4)	0.28 (0.04 to 0.7)	1.22 (0.92 to 1.52)
Suriname	Diet high in processed meat	0 (0 to 1)	0.14 (0.03 to 0.26)	1 (0 to 2)	0.2 (0.04 to 0.38)	1.33 (1.08 to 1.58)
Suriname	Diet low in whole grains	4 (2 to 6)	1.8 (0.67 to 2.46)	14 (5 to 19)	2.41 (0.89 to 3.35)	1.09 (0.85 to 1.34)
Sweden	All dietary risks	901 (630 to 1111)	5.74 (4.06 to 7.06)	1128 (800 to 1400)	4.98 (3.53 to 6.19)	-0.61 (-0.73 to -0.48)
Sweden	Diet low in milk	497 (197 to 638)	3.17 (1.25 to 4.05)	627 (245 to 811)	2.76 (1.07 to 3.56)	-0.59 (-0.73 to -0.45)
Sweden	Diet low in fiber	89 (32 to 165)	0.56 (0.2 to 1.04)	77 (28 to 161)	0.33 (0.12 to 0.68)	-2.15 (-2.29 to -2.01)
Sweden	Diet low in calcium	106 (53 to 179)	0.64 (0.32 to 1.08)	104 (50 to 174)	0.44 (0.21 to 0.73)	-1.61 (-1.71 to -1.5)
Sweden	Diet high in red meat	143 (29 to 302)	0.95 (0.2 to 1.96)	215 (53 to 418)	1 (0.26 to 1.88)	0.09 (-0.02 to 0.2)
Sweden	Diet high in processed meat	184 (62 to 283)	1.18 (0.4 to 1.83)	248 (89 to 389)	1.11 (0.41 to 1.76)	-0.27 (-0.41 to -0.13)
Sweden	Diet low in whole grains	497 (197 to 638)	3.17 (1.25 to 4.05)	627 (245 to 811)	2.76 (1.07 to 3.56)	-0.59 (-0.73 to -0.45)
Switzerland	All dietary risks	496 (360 to 604)	4.62 (3.37 to 5.61)	747 (534 to 924)	3.98 (2.88 to 4.9)	-0.72 (-0.9 to -0.55)
Switzerland	Diet low in milk	256 (95 to 331)	2.38 (0.88 to 3.08)	393 (151 to 516)	2.08 (0.8 to 2.73)	-0.71 (-0.91 to -0.52)

Countries	Risk factors	1990 Number of deaths (95% UI)	1990 ASDR per 100,000 (95% UI)	2019 Number of deaths (95% UI)	2019 ASDR per 100,000 (95% UI)	1990-2019 EAPC of ASDR (95% CI)
Switzerland	Diet low in fiber	50 (19 to 93)	0.46 (0.17 to 0.85)	79 (30 to 146)	0.41 (0.16 to 0.76)	-0.52 (-0.8 to -0.24)
Switzerland	Diet low in calcium	52 (23 to 90)	0.46 (0.2 to 0.79)	90 (40 to 158)	0.45 (0.2 to 0.8)	-0.17 (-0.42 to 0.08)
Switzerland	Diet high in red meat	130 (40 to 217)	1.24 (0.39 to 2.04)	171 (51 to 303)	0.95 (0.29 to 1.64)	-1.09 (-1.19 to -0.99)
Switzerland	Diet high in processed meat	78 (19 to 121)	0.74 (0.18 to 1.13)	123 (34 to 191)	0.67 (0.18 to 1.03)	-0.54 (-0.7 to -0.37)
Switzerland	Diet low in whole grains	256 (95 to 331)	2.38 (0.88 to 3.08)	393 (151 to 516)	2.08 (0.8 to 2.73)	-0.71 (-0.91 to -0.52)
Syrian Arab Republic	All dietary risks	97 (67 to 128)	1.95 (1.36 to 2.59)	241 (156 to 342)	2.24 (1.49 to 3.1)	0.29 (0.07 to 0.51)
Syrian Arab Republic	Diet low in milk	54 (25 to 76)	1.09 (0.5 to 1.48)	140 (64 to 199)	1.29 (0.59 to 1.81)	0.49 (0.29 to 0.7)
Syrian Arab Republic	Diet low in fiber	4 (1 to 9)	0.08 (0.03 to 0.18)	10 (3 to 23)	0.1 (0.03 to 0.23)	-0.05 (-0.41 to 0.32)
Syrian Arab Republic	Diet low in calcium	42 (27 to 60)	0.86 (0.57 to 1.21)	100 (61 to 154)	0.96 (0.61 to 1.43)	0.03 (-0.25 to 0.32)
Syrian Arab Republic	Diet high in red meat	7 (1 to 20)	0.13 (0.01 to 0.38)	16 (1 to 45)	0.14 (0.01 to 0.4)	0.06 (-0.15 to 0.28)
Syrian Arab Republic	Diet high in processed meat	3 (1 to 5)	0.05 (0.01 to 0.1)	6 (2 to 13)	0.06 (0.01 to 0.11)	0.3 (0.09 to 0.52)
Syrian Arab Republic	Diet low in whole grains	54 (25 to 76)	1.09 (0.5 to 1.48)	140 (64 to 199)	1.29 (0.59 to 1.81)	0.49 (0.29 to 0.7)
Taiwan (Province of China)	All dietary risks	757 (549 to 933)	5.07 (3.68 to 6.23)	3485 (2400 to 4805)	8.82 (6.08 to 12.15)	2.12 (1.88 to 2.36)
Taiwan (Province of China)	Diet low in milk	362 (135 to 477)	2.4 (0.9 to 3.16)	1658 (589 to 2387)	4.19 (1.49 to 6.04)	1.93 (1.69 to 2.16)
Taiwan (Province of China)	Diet low in fiber	84 (31 to 150)	0.57 (0.21 to 1)	265 (90 to 532)	0.67 (0.23 to 1.35)	1.22 (0.93 to 1.52)
Taiwan (Province of China)	Diet low in calcium	288 (185 to 419)	1.97 (1.26 to 2.84)	1167 (666 to 1880)	2.95 (1.68 to 4.76)	1.86 (1.58 to 2.15)

Countries	Risk factors	1990 Number of deaths (95% UI)	1990 ASDR per 100,000 (95% UI)	2019 Number of deaths (95% UI)	2019 ASDR per 100,000 (95% UI)	1990-2019 EAPC of ASDR (95% CI)
Taiwan (Province of China)	Diet high in red meat	95 (13 to 225)	0.62 (0.08 to 1.49)	713 (189 to 1384)	1.81 (0.48 to 3.52)	3.86 (3.49 to 4.24)
Taiwan (Province of China)	Diet high in processed meat	33 (5 to 59)	0.21 (0.03 to 0.38)	192 (23 to 367)	0.49 (0.06 to 0.93)	3.09 (2.87 to 3.31)
Taiwan (Province of China)	Diet low in whole grains	362 (135 to 477)	2.4 (0.9 to 3.16)	1658 (589 to 2387)	4.19 (1.49 to 6.04)	1.93 (1.69 to 2.16)
Tajikistan	All dietary risks	109 (87 to 128)	3.77 (3.02 to 4.43)	182 (137 to 233)	4.41 (3.36 to 5.57)	1.12 (0.74 to 1.51)
Tajikistan	Diet low in milk	54 (27 to 67)	1.88 (0.93 to 2.34)	90 (43 to 121)	2.17 (1.04 to 2.91)	1.16 (0.71 to 1.62)
Tajikistan	Diet low in fiber	10 (4 to 18)	0.34 (0.12 to 0.61)	14 (5 to 26)	0.36 (0.13 to 0.65)	0.32 (0.16 to 0.47)
Tajikistan	Diet low in calcium	52 (41 to 65)	1.8 (1.41 to 2.25)	90 (67 to 119)	2.22 (1.67 to 2.89)	1.23 (0.93 to 1.54)
Tajikistan	Diet high in red meat	6 (1 to 17)	0.21 (0.02 to 0.57)	9 (1 to 25)	0.21 (0.02 to 0.56)	0.84 (0.13 to 1.56)
Tajikistan	Diet high in processed meat	8 (1 to 13)	0.26 (0.03 to 0.44)	12 (2 to 22)	0.27 (0.03 to 0.49)	0.78 (0.21 to 1.36)
Tajikistan	Diet low in whole grains	54 (27 to 67)	1.88 (0.93 to 2.34)	90 (43 to 121)	2.17 (1.04 to 2.91)	1.16 (0.71 to 1.62)
Thailand	All dietary risks	1517 (1230 to 1800)	4.6 (3.72 to 5.48)	4069 (2857 to 5580)	4.07 (2.87 to 5.57)	-0.96 (-1.2 to -0.71)
Thailand	Diet low in milk	472 (175 to 672)	1.43 (0.54 to 2.02)	1329 (490 to 2057)	1.33 (0.49 to 2.06)	-0.79 (-1.06 to -0.53)
Thailand	Diet low in fiber	262 (134 to 375)	0.8 (0.41 to 1.14)	557 (242 to 960)	0.56 (0.24 to 0.96)	-1.74 (-1.94 to -1.55)
Thailand	Diet low in calcium	953 (733 to 1177)	2.9 (2.23 to 3.59)	2491 (1727 to 3403)	2.49 (1.73 to 3.4)	-1.05 (-1.3 to -0.8)
Thailand	Diet high in red meat	74 (8 to 197)	0.22 (0.02 to 0.57)	242 (22 to 697)	0.24 (0.02 to 0.69)	-0.2 (-0.46 to 0.06)
Thailand	Diet high in processed meat	17 (11 to 23)	0.05 (0.03 to 0.07)	66 (30 to 114)	0.07 (0.03 to 0.11)	0.48 (0.27 to 0.7)

Countries	Risk factors	1990 Number of deaths (95% UI)	1990 ASDR per 100,000 (95% UI)	2019 Number of deaths (95% UI)	2019 ASDR per 100,000 (95% UI)	1990-2019 EAPC of ASDR (95% CI)
Thailand	Diet low in whole grains	472 (175 to 672)	1.43 (0.54 to 2.02)	1329 (490 to 2057)	1.33 (0.49 to 2.06)	-0.79 (-1.06 to -0.53)
Timor-Leste	All dietary risks	8 (6 to 11)	3.02 (2.17 to 4.1)	37 (23 to 50)	4.9 (3.16 to 6.62)	2.05 (1.81 to 2.28)
Timor-Leste	Diet low in milk	3 (1 to 4)	1 (0.39 to 1.55)	12 (5 to 19)	1.63 (0.61 to 2.53)	2.07 (1.84 to 2.3)
Timor-Leste	Diet low in fiber	0 (0 to 1)	0.19 (0.06 to 0.41)	2 (1 to 4)	0.28 (0.1 to 0.62)	1.4 (1.27 to 1.53)
Timor-Leste	Diet low in calcium	5 (4 to 8)	2.08 (1.46 to 2.9)	25 (16 to 34)	3.35 (2.12 to 4.58)	2.04 (1.8 to 2.27)
Timor-Leste	Diet high in red meat	0 (0 to 1)	0.13 (0.01 to 0.36)	2 (0 to 5)	0.24 (0.02 to 0.67)	2.39 (2.11 to 2.67)
Timor-Leste	Diet high in processed meat	0 (0 to 0)	0.04 (0.02 to 0.06)	1 (0 to 1)	0.07 (0.04 to 0.11)	2.53 (2.21 to 2.85)
Timor-Leste	Diet low in whole grains	3 (1 to 4)	1 (0.39 to 1.55)	12 (5 to 19)	1.63 (0.61 to 2.53)	2.07 (1.84 to 2.3)
Togo	All dietary risks	32 (25 to 40)	2.89 (2.26 to 3.59)	97 (67 to 132)	3.14 (2.2 to 4.16)	0.23 (0.19 to 0.27)
Togo	Diet low in milk	12 (4 to 17)	1.1 (0.41 to 1.54)	36 (13 to 54)	1.17 (0.43 to 1.7)	0.15 (0.1 to 0.2)
Togo	Diet low in fiber	0 (0 to 1)	0.03 (0.02 to 0.05)	1 (0 to 1)	0.02 (0.01 to 0.04)	-0.63 (-0.87 to -0.39)
Togo	Diet low in calcium	21 (16 to 27)	1.93 (1.46 to 2.46)	64 (44 to 88)	2.08 (1.43 to 2.81)	0.2 (0.16 to 0.24)
Togo	Diet high in red meat	1 (0 to 3)	0.09 (0.01 to 0.23)	3 (0 to 9)	0.1 (0.02 to 0.27)	0.48 (0.39 to 0.58)
Togo	Diet high in processed meat	2 (0 to 3)	0.14 (0.02 to 0.27)	6 (1 to 12)	0.18 (0.02 to 0.36)	0.83 (0.8 to 0.87)
Togo	Diet low in whole grains	12 (4 to 17)	1.1 (0.41 to 1.54)	36 (13 to 54)	1.17 (0.43 to 1.7)	0.15 (0.1 to 0.2)
Tokelau	All dietary risks	0 (0 to 0)	3.9 (2.91 to 4.97)	0 (0 to 0)	4.07 (2.84 to 5.45)	0.17 (0.15 to 0.19)
Tokelau	Diet low in milk	0 (0 to 0)	1.65 (0.61 to 2.3)	0 (0 to 0)	1.78 (0.63 to 2.65)	0.3 (0.27 to 0.34)
Tokelau	Diet low in fiber	0 (0 to 0)	0.04 (0.02 to 0.1)	0 (0 to 0)	0.03 (0.02 to 0.06)	-1.43 (-1.49 to -1.37)

Countries	Risk factors	1990 Number of deaths (95% UI)	1990 ASDR per 100,000 (95% UI)	2019 Number of deaths (95% UI)	2019 ASDR per 100,000 (95% UI)	1990-2019 EAPC of ASDR (95% CI)
Tokelau	Diet low in calcium	0 (0 to 0)	2.41 (1.8 to 3.12)	0 (0 to 0)	2.41 (1.71 to 3.27)	0 (-0.01 to 0.01)
Tokelau	Diet high in red meat	0 (0 to 0)	0.27 (0.02 to 0.76)	0 (0 to 0)	0.31 (0.03 to 0.84)	0.51 (0.45 to 0.57)
Tokelau	Diet high in processed meat	0 (0 to 0)	0.08 (0.03 to 0.14)	0 (0 to 0)	0.1 (0.03 to 0.18)	0.74 (0.68 to 0.79)
Tokelau	Diet low in whole grains	0 (0 to 0)	1.65 (0.61 to 2.3)	0 (0 to 0)	1.78 (0.63 to 2.65)	0.3 (0.27 to 0.34)
Tonga	All dietary risks	1 (1 to 1)	2.38 (1.83 to 2.94)	2 (1 to 3)	2.64 (1.92 to 3.45)	0.28 (0.15 to 0.41)
Tonga	Diet low in milk	0 (0 to 1)	1 (0.37 to 1.37)	1 (0 to 1)	1.14 (0.4 to 1.63)	0.36 (0.23 to 0.48)
Tonga	Diet low in fiber	0 (0 to 0)	0.03 (0.01 to 0.06)	0 (0 to 0)	0.02 (0.01 to 0.04)	-0.52 (-0.64 to -0.39)
Tonga	Diet low in calcium	1 (1 to 1)	1.48 (1.13 to 1.88)	1 (1 to 2)	1.6 (1.17 to 2.16)	0.2 (0.07 to 0.33)
Tonga	Diet high in red meat	0 (0 to 0)	0.16 (0.01 to 0.43)	0 (0 to 0)	0.19 (0.02 to 0.52)	0.37 (0.24 to 0.5)
Tonga	Diet high in processed meat	0 (0 to 0)	0.05 (0.02 to 0.08)	0 (0 to 0)	0.06 (0.02 to 0.11)	0.67 (0.56 to 0.78)
Tonga	Diet low in whole grains	0 (0 to 1)	1 (0.37 to 1.37)	1 (0 to 1)	1.14 (0.4 to 1.63)	0.36 (0.23 to 0.48)
Trinidad and Tobago	All dietary risks	41 (29 to 49)	5.22 (3.79 to 6.28)	79 (51 to 108)	4.39 (2.83 to 5.98)	-0.98 (-1.13 to -0.82)
Trinidad and Tobago	Diet low in milk	21 (8 to 27)	2.65 (1.03 to 3.45)	44 (17 to 65)	2.45 (0.95 to 3.58)	-0.48 (-0.61 to -0.35)
Trinidad and Tobago	Diet low in fiber	4 (1 to 7)	0.48 (0.16 to 0.9)	5 (2 to 11)	0.29 (0.1 to 0.63)	-2.73 (-3.05 to -2.41)
Trinidad and Tobago	Diet low in calcium	18 (12 to 25)	2.3 (1.6 to 3.19)	30 (17 to 48)	1.69 (0.97 to 2.63)	-1.71 (-1.95 to -1.48)
Trinidad and Tobago	Diet high in red meat	2 (0 to 6)	0.3 (0.03 to 0.79)	6 (1 to 16)	0.31 (0.03 to 0.88)	0.24 (0.06 to 0.42)
Trinidad and Tobago	Diet high in processed meat	1 (0 to 3)	0.19 (0.03 to 0.33)	3 (1 to 7)	0.18 (0.03 to 0.35)	-0.13 (-0.24 to -0.01)
Trinidad and Tobago	Diet low in whole grains	21 (8 to 27)	2.65 (1.03 to 3.45)	44 (17 to 65)	2.45 (0.95 to 3.58)	-0.48 (-0.61 to -0.35)

Countries	Risk factors	1990		2019		1990-2019
		Number of deaths (95% UI)	ASDR per 100,000 (95% UI)	Number of deaths (95% UI)	ASDR per 100,000 (95% UI)	EAPC of ASDR (95% CI)
Tunisia	All dietary risks	125 (89 to 159)	2.82 (1.99 to 3.57)	352 (215 to 498)	2.98 (1.84 to 4.21)	0.02 (-0.05 to 0.09)
Tunisia	Diet low in milk	71 (32 to 94)	1.6 (0.73 to 2.11)	228 (99 to 332)	1.92 (0.84 to 2.78)	0.52 (0.45 to 0.59)
Tunisia	Diet low in fiber	2 (1 to 5)	0.05 (0.02 to 0.12)	5 (2 to 10)	0.04 (0.02 to 0.09)	-1.1 (-1.3 to -0.9)
Tunisia	Diet low in calcium	55 (37 to 77)	1.26 (0.86 to 1.75)	118 (64 to 196)	1.02 (0.56 to 1.67)	-1.08 (-1.19 to -0.97)
Tunisia	Diet high in red meat	8 (1 to 23)	0.18 (0.02 to 0.49)	25 (2 to 72)	0.21 (0.02 to 0.6)	0.36 (0.28 to 0.45)
Tunisia	Diet high in processed meat	4 (1 to 7)	0.08 (0.02 to 0.14)	12 (2 to 25)	0.1 (0.02 to 0.21)	0.84 (0.76 to 0.93)
Tunisia	Diet low in whole grains	71 (32 to 94)	1.6 (0.73 to 2.11)	228 (99 to 332)	1.92 (0.84 to 2.78)	0.52 (0.45 to 0.59)
Turkey	All dietary risks	1370 (898 to 1844)	4 (2.63 to 5.34)	3302 (2139 to 4361)	3.87 (2.51 to 5.1)	0.2 (-0.22 to 0.62)
Turkey	Diet low in milk	963 (473 to 1297)	2.8 (1.38 to 3.76)	2379 (1221 to 3237)	2.79 (1.43 to 3.79)	0.27 (-0.19 to 0.73)
Turkey	Diet low in fiber	14 (9 to 25)	0.04 (0.03 to 0.07)	35 (22 to 66)	0.04 (0.03 to 0.08)	0.78 (0.39 to 1.17)
Turkey	Diet low in calcium	371 (185 to 639)	1.1 (0.55 to 1.88)	796 (379 to 1409)	0.94 (0.45 to 1.66)	-0.15 (-0.43 to 0.13)
Turkey	Diet high in red meat	107 (9 to 297)	0.31 (0.03 to 0.85)	256 (23 to 711)	0.3 (0.03 to 0.82)	0.21 (-0.38 to 0.79)
Turkey	Diet high in processed meat	40 (10 to 74)	0.11 (0.03 to 0.21)	119 (23 to 228)	0.14 (0.03 to 0.26)	1.08 (0.47 to 1.69)
Turkey	Diet low in whole grains	963 (473 to 1297)	2.8 (1.38 to 3.76)	2379 (1221 to 3237)	2.79 (1.43 to 3.79)	0.27 (-0.19 to 0.73)
Turkmenistan	All dietary risks	56 (42 to 67)	2.87 (2.15 to 3.43)	97 (69 to 128)	2.68 (1.93 to 3.54)	-0.53 (-0.93 to -0.12)
Turkmenistan	Diet low in milk	31 (15 to 38)	1.57 (0.78 to 1.93)	54 (26 to 73)	1.51 (0.73 to 2.02)	-0.36 (-0.79 to 0.07)

Countries	Risk factors	1990 Number of deaths (95% UI)	1990 ASDR per 100,000 (95% UI)	2019 Number of deaths (95% UI)	2019 ASDR per 100,000 (95% UI)	1990-2019 EAPC of ASDR (95% CI)
Turkmenistan	Diet low in fiber	5 (2 to 9)	0.27 (0.1 to 0.48)	5 (2 to 11)	0.15 (0.05 to 0.32)	-2.81 (-3.15 to -2.46)
Turkmenistan	Diet low in calcium	17 (10 to 25)	0.88 (0.55 to 1.3)	20 (10 to 37)	0.58 (0.29 to 1.04)	-2.02 (-2.35 to -1.69)
Turkmenistan	Diet high in red meat	8 (1 to 16)	0.37 (0.07 to 0.78)	22 (7 to 40)	0.59 (0.18 to 1.06)	2.25 (1.47 to 3.04)
Turkmenistan	Diet high in processed meat	6 (1 to 9)	0.29 (0.05 to 0.47)	11 (3 to 19)	0.29 (0.06 to 0.5)	-0.07 (-0.61 to 0.47)
Turkmenistan	Diet low in whole grains	31 (15 to 38)	1.57 (0.78 to 1.93)	54 (26 to 73)	1.51 (0.73 to 2.02)	-0.36 (-0.79 to 0.07)
Tuvalu	All dietary risks	0 (0 to 0)	4.06 (3.14 to 5.09)	0 (0 to 1)	4.26 (3.01 to 5.82)	0.02 (-0.02 to 0.07)
Tuvalu	Diet low in milk	0 (0 to 0)	1.69 (0.63 to 2.32)	0 (0 to 0)	1.81 (0.65 to 2.67)	0.08 (0.03 to 0.13)
Tuvalu	Diet low in fiber	0 (0 to 0)	0.06 (0.03 to 0.16)	0 (0 to 0)	0.05 (0.02 to 0.1)	-1 (-1.13 to -0.86)
Tuvalu	Diet low in calcium	0 (0 to 0)	2.56 (1.95 to 3.25)	0 (0 to 0)	2.62 (1.88 to 3.55)	-0.04 (-0.08 to 0)
Tuvalu	Diet high in red meat	0 (0 to 0)	0.26 (0.02 to 0.71)	0 (0 to 0)	0.29 (0.02 to 0.82)	0.15 (0.09 to 0.22)
Tuvalu	Diet high in processed meat	0 (0 to 0)	0.08 (0.03 to 0.14)	0 (0 to 0)	0.09 (0.03 to 0.18)	0.39 (0.34 to 0.44)
Tuvalu	Diet low in whole grains	0 (0 to 0)	1.69 (0.63 to 2.32)	0 (0 to 0)	1.81 (0.65 to 2.67)	0.08 (0.03 to 0.13)
Uganda	All dietary risks	144 (110 to 178)	2.43 (1.88 to 3)	523 (379 to 683)	4.03 (2.96 to 5.14)	1.73 (1.52 to 1.94)
Uganda	Diet low in milk	60 (23 to 83)	1 (0.38 to 1.38)	214 (80 to 305)	1.64 (0.61 to 2.34)	1.67 (1.47 to 1.88)
Uganda	Diet low in fiber	1 (1 to 2)	0.02 (0.01 to 0.04)	5 (3 to 9)	0.04 (0.02 to 0.08)	1.93 (1.72 to 2.14)
Uganda	Diet low in calcium	88 (66 to 113)	1.5 (1.14 to 1.91)	314 (226 to 414)	2.45 (1.8 to 3.19)	1.67 (1.45 to 1.88)
Uganda	Diet high in red meat	7 (1 to 19)	0.12 (0.01 to 0.3)	31 (3 to 86)	0.23 (0.02 to 0.62)	2.38 (2.14 to 2.62)
Uganda	Diet high in processed meat	6 (1 to 11)	0.09 (0.01 to 0.18)	24 (3 to 46)	0.17 (0.03 to 0.33)	2.07 (1.85 to 2.3)

Countries	Risk factors	1990 Number of deaths (95% UI)	1990 ASDR per 100,000 (95% UI)	2019 Number of deaths (95% UI)	2019 ASDR per 100,000 (95% UI)	1990-2019 EAPC of ASDR (95% CI)
Uganda	Diet low in whole grains	60 (23 to 83)	1 (0.38 to 1.38)	214 (80 to 305)	1.64 (0.61 to 2.34)	1.67 (1.47 to 1.88)
Ukraine	All dietary risks	4983 (3551 to 6145)	6.99 (4.97 to 8.63)	4361 (2921 to 5696)	5.73 (3.86 to 7.48)	-1.64 (-2.01 to -1.27)
Ukraine	Diet low in milk	3279 (1633 to 4028)	4.59 (2.28 to 5.64)	2584 (992 to 3509)	3.39 (1.31 to 4.62)	-2.01 (-2.39 to -1.64)
Ukraine	Diet low in fiber	138 (51 to 348)	0.2 (0.07 to 0.5)	170 (59 to 412)	0.22 (0.08 to 0.55)	-1.16 (-1.81 to -0.5)
Ukraine	Diet low in calcium	1159 (575 to 2058)	1.63 (0.81 to 2.89)	1191 (618 to 2017)	1.55 (0.8 to 2.65)	-1.45 (-1.95 to -0.95)
Ukraine	Diet high in red meat	620 (81 to 1464)	0.87 (0.12 to 2.06)	477 (46 to 1302)	0.63 (0.06 to 1.72)	-1.69 (-2.17 to -1.2)
Ukraine	Diet high in processed meat	360 (42 to 626)	0.51 (0.06 to 0.88)	434 (52 to 755)	0.58 (0.07 to 1)	0.17 (-0.18 to 0.52)
Ukraine	Diet low in whole grains	3279 (1633 to 4028)	4.59 (2.28 to 5.64)	2584 (992 to 3509)	3.39 (1.31 to 4.62)	-2.01 (-2.39 to -1.64)
United Arab Emirates	All dietary risks	16 (9 to 24)	5.29 (3.06 to 7.77)	128 (73 to 188)	5.11 (2.87 to 7.71)	-0.14 (-0.52 to 0.23)
United Arab Emirates	Diet low in milk	10 (4 to 15)	3.31 (1.33 to 4.98)	79 (28 to 121)	3.08 (1.06 to 4.73)	-0.34 (-0.75 to 0.07)
United Arab Emirates	Diet low in fiber	0 (0 to 1)	0.12 (0.04 to 0.31)	3 (1 to 8)	0.15 (0.05 to 0.35)	0.91 (0.72 to 1.11)
United Arab Emirates	Diet low in calcium	3 (1 to 6)	1.17 (0.51 to 2.18)	38 (18 to 69)	1.7 (0.84 to 3.06)	1.39 (0.99 to 1.8)
United Arab Emirates	Diet high in red meat	3 (1 to 7)	0.9 (0.17 to 2.18)	14 (1 to 40)	0.5 (0.04 to 1.49)	-2.11 (-2.32 to -1.89)
United Arab Emirates	Diet high in processed meat	1 (0 to 2)	0.32 (0.04 to 0.66)	7 (1 to 14)	0.23 (0.03 to 0.49)	-1.46 (-1.98 to -0.94)
United Arab Emirates	Diet low in whole grains	10 (4 to 15)	3.31 (1.33 to 4.98)	79 (28 to 121)	3.08 (1.06 to 4.73)	-0.34 (-0.75 to 0.07)
United Kingdom	All dietary risks	7388 (5345 to 9035)	8.03 (5.82 to 9.83)	7470 (5153 to 9213)	5.58 (3.91 to 6.87)	-1.47 (-1.65 to -1.28)
United Kingdom	Diet low in milk	3781 (1420 to 4869)	4.11 (1.54 to 5.28)	4056 (1520 to 5244)	3.03 (1.13 to 3.91)	-1.28 (-1.42 to -1.15)

Countries	Risk factors	1990 Number of deaths (95% UI)	1990 ASDR per 100,000 (95% UI)	2019 Number of deaths (95% UI)	2019 ASDR per 100,000 (95% UI)	1990-2019 EAPC of ASDR (95% CI)
United Kingdom	Diet low in fiber	623 (244 to 1142)	0.67 (0.27 to 1.23)	549 (216 to 1082)	0.4 (0.16 to 0.8)	-1.89 (-2.24 to -1.54)
United Kingdom	Diet low in calcium	1448 (707 to 2583)	1.55 (0.75 to 2.78)	1180 (544 to 2069)	0.85 (0.4 to 1.5)	-2.18 (-2.6 to -1.76)
United Kingdom	Diet high in red meat	1193 (297 to 2369)	1.32 (0.33 to 2.6)	1059 (221 to 2248)	0.81 (0.18 to 1.7)	-1.96 (-2.22 to -1.69)
United Kingdom	Diet high in processed meat	1415 (504 to 2197)	1.55 (0.55 to 2.41)	1591 (560 to 2518)	1.21 (0.44 to 1.91)	-1.08 (-1.18 to -0.97)
United Kingdom	Diet low in whole grains	3781 (1420 to 4869)	4.11 (1.54 to 5.28)	4056 (1520 to 5244)	3.03 (1.13 to 3.91)	-1.28 (-1.42 to -1.15)
United Republic of Tanzania	All dietary risks	291 (221 to 371)	2.93 (2.25 to 3.67)	778 (561 to 1037)	3.43 (2.53 to 4.47)	0.57 (0.51 to 0.63)
United Republic of Tanzania	Diet low in milk	93 (35 to 140)	0.93 (0.35 to 1.41)	278 (102 to 430)	1.21 (0.45 to 1.84)	0.98 (0.93 to 1.02)
United Republic of Tanzania	Diet low in fiber	4 (2 to 9)	0.04 (0.02 to 0.1)	9 (5 to 18)	0.04 (0.02 to 0.08)	-0.25 (-0.39 to -0.11)
United Republic of Tanzania	Diet low in calcium	202 (151 to 265)	2.04 (1.54 to 2.64)	512 (377 to 685)	2.27 (1.7 to 2.98)	0.38 (0.32 to 0.43)
United Republic of Tanzania	Diet high in red meat	15 (2 to 40)	0.15 (0.02 to 0.39)	39 (4 to 103)	0.17 (0.02 to 0.44)	0.47 (0.25 to 0.69)
United Republic of Tanzania	Diet high in processed meat	13 (2 to 24)	0.12 (0.02 to 0.23)	35 (5 to 69)	0.15 (0.02 to 0.29)	0.85 (0.69 to 1.01)
United Republic of Tanzania	Diet low in whole grains	93 (35 to 140)	0.93 (0.35 to 1.41)	278 (102 to 430)	1.21 (0.45 to 1.84)	0.98 (0.93 to 1.02)
United States of America	All dietary risks	21198 (15512 to 26161)	6.48 (4.75 to 7.98)	26824 (19397 to 33359)	4.72 (3.46 to 5.84)	-1.26 (-1.36 to -1.16)
United States of America	Diet low in milk	10015 (3764 to 13318)	3.06 (1.15 to 4.07)	13049 (4889 to 17133)	2.29 (0.86 to 3.02)	-1.23 (-1.33 to -1.13)
United States of America	Diet low in fiber	2016 (787 to 3663)	0.61 (0.24 to 1.11)	1760 (673 to 3441)	0.3 (0.12 to 0.59)	-2.47 (-2.63 to -2.31)
United States of America	Diet low in calcium	3924 (2017 to 6719)	1.18 (0.6 to 2.02)	4338 (2122 to 7529)	0.74 (0.36 to 1.28)	-1.35 (-1.65 to -1.05)
United States of America	Diet high in red meat	4392 (1231 to 7939)	1.37 (0.39 to 2.45)	5738 (1601 to 10372)	1.03 (0.29 to 1.84)	-1.2 (-1.32 to -1.08)

Countries	Risk factors	1990 Number of deaths (95% UI)	1990 ASDR per 100,000 (95% UI)	2019 Number of deaths (95% UI)	2019 ASDR per 100,000 (95% UI)	1990-2019 EAPC of ASDR (95% CI)
United States of America	Diet high in processed meat	3973 (1348 to 6029)	1.22 (0.42 to 1.86)	5751 (2074 to 9197)	1.03 (0.38 to 1.64)	-0.83 (-0.93 to -0.74)
United States of America	Diet low in whole grains	10015 (3764 to 13318)	3.06 (1.15 to 4.07)	13049 (4889 to 17133)	2.29 (0.86 to 3.02)	-1.23 (-1.33 to -1.13)
United States Virgin Islands	All dietary risks	5 (3 to 6)	6.35 (4.4 to 8.04)	14 (9 to 18)	7.64 (4.88 to 10)	0.93 (0.75 to 1.1)
United States Virgin Islands	Diet low in milk	2 (1 to 3)	3.26 (1.22 to 4.39)	8 (3 to 11)	4.36 (1.6 to 5.95)	1.3 (1.09 to 1.51)
United States Virgin Islands	Diet low in fiber	0 (0 to 1)	0.46 (0.17 to 0.91)	1 (0 to 1)	0.37 (0.14 to 0.84)	-0.72 (-1.04 to -0.39)
United States Virgin Islands	Diet low in calcium	2 (1 to 3)	2.69 (1.73 to 3.89)	5 (3 to 8)	2.65 (1.47 to 4.27)	0.15 (-0.05 to 0.36)
United States Virgin Islands	Diet high in red meat	0 (0 to 1)	0.5 (0.05 to 1.36)	1 (0 to 4)	0.72 (0.07 to 1.94)	1.64 (1.37 to 1.91)
United States Virgin Islands	Diet high in processed meat	0 (0 to 0)	0.23 (0.04 to 0.44)	1 (0 to 1)	0.34 (0.06 to 0.64)	1.67 (1.41 to 1.94)
United States Virgin Islands	Diet low in whole grains	2 (1 to 3)	3.26 (1.22 to 4.39)	8 (3 to 11)	4.36 (1.6 to 5.95)	1.3 (1.09 to 1.51)
Uruguay	All dietary risks	424 (323 to 503)	10.91 (8.31 to 12.95)	535 (380 to 659)	9.19 (6.57 to 11.28)	-0.7 (-0.77 to -0.63)
Uruguay	Diet low in milk	190 (72 to 244)	4.9 (1.87 to 6.31)	275 (106 to 357)	4.72 (1.81 to 6.13)	-0.19 (-0.27 to -0.1)
Uruguay	Diet low in fiber	42 (16 to 73)	1.08 (0.41 to 1.88)	31 (11 to 65)	0.51 (0.18 to 1.08)	-2.77 (-2.88 to -2.66)
Uruguay	Diet low in calcium	116 (68 to 180)	2.98 (1.75 to 4.63)	108 (54 to 188)	1.81 (0.9 to 3.15)	-1.72 (-1.84 to -1.6)
Uruguay	Diet high in red meat	113 (45 to 175)	2.9 (1.16 to 4.49)	133 (44 to 225)	2.34 (0.78 to 3.89)	-1.09 (-1.25 to -0.92)
Uruguay	Diet high in processed meat	45 (9 to 72)	1.16 (0.23 to 1.87)	72 (15 to 115)	1.25 (0.27 to 1.98)	0.23 (0.16 to 0.29)
Uruguay	Diet low in whole grains	190 (72 to 244)	4.9 (1.87 to 6.31)	275 (106 to 357)	4.72 (1.81 to 6.13)	-0.19 (-0.27 to -0.1)
Uzbekistan	All dietary risks	297 (219 to 359)	2.59 (1.9 to 3.13)	565 (398 to 715)	3.63 (2.6 to 4.59)	1.24 (1.09 to 1.39)

Countries	Risk factors	1990 Number of deaths (95% UI)	1990 ASDR per 100,000 (95% UI)	2019 Number of deaths (95% UI)	2019 ASDR per 100,000 (95% UI)	1990-2019 EAPC of ASDR (95% CI)
Uzbekistan	Diet low in milk	171 (84 to 212)	1.49 (0.73 to 1.85)	344 (165 to 449)	2.21 (1.06 to 2.84)	1.53 (1.41 to 1.65)
Uzbekistan	Diet low in fiber	25 (9 to 47)	0.21 (0.07 to 0.41)	22 (7 to 51)	0.17 (0.06 to 0.38)	-1.23 (-1.74 to -0.73)
Uzbekistan	Diet low in calcium	89 (53 to 135)	0.79 (0.46 to 1.18)	143 (75 to 239)	0.99 (0.54 to 1.6)	0.74 (0.33 to 1.16)
Uzbekistan	Diet high in red meat	29 (3 to 71)	0.25 (0.02 to 0.61)	73 (12 to 169)	0.41 (0.06 to 0.98)	1.98 (1.71 to 2.25)
Uzbekistan	Diet high in processed meat	31 (5 to 51)	0.26 (0.04 to 0.43)	62 (12 to 107)	0.35 (0.06 to 0.62)	1.15 (0.95 to 1.36)
Uzbekistan	Diet low in whole grains	171 (84 to 212)	1.49 (0.73 to 1.85)	344 (165 to 449)	2.21 (1.06 to 2.84)	1.53 (1.41 to 1.65)
Vanuatu	All dietary risks	2 (1 to 3)	3.19 (2.27 to 4.21)	6 (4 to 8)	3.76 (2.7 to 5.01)	0.39 (0.25 to 0.54)
Vanuatu	Diet low in milk	1 (0 to 1)	1.28 (0.47 to 1.83)	2 (1 to 3)	1.53 (0.54 to 2.19)	0.47 (0.33 to 0.61)
Vanuatu	Diet low in fiber	0 (0 to 0)	0.03 (0.01 to 0.06)	0 (0 to 0)	0.03 (0.02 to 0.05)	-0.7 (-0.87 to -0.52)
Vanuatu	Diet low in calcium	1 (1 to 2)	2.02 (1.43 to 2.75)	4 (3 to 5)	2.36 (1.71 to 3.17)	0.34 (0.2 to 0.49)
Vanuatu	Diet high in red meat	0 (0 to 0)	0.29 (0.03 to 0.73)	1 (0 to 1)	0.32 (0.03 to 0.87)	0.16 (-0.03 to 0.35)
Vanuatu	Diet high in processed meat	0 (0 to 0)	0.06 (0.02 to 0.1)	0 (0 to 0)	0.08 (0.02 to 0.14)	0.85 (0.72 to 0.98)
Vanuatu	Diet low in whole grains	1 (0 to 1)	1.28 (0.47 to 1.83)	2 (1 to 3)	1.53 (0.54 to 2.19)	0.47 (0.33 to 0.61)
Venezuela (Bolivarian Republic of)	All dietary risks	276 (215 to 329)	3.01 (2.35 to 3.59)	1094 (751 to 1501)	3.88 (2.69 to 5.29)	0.38 (0.19 to 0.56)
Venezuela (Bolivarian Republic of)	Diet low in milk	111 (42 to 151)	1.21 (0.46 to 1.64)	436 (161 to 636)	1.54 (0.57 to 2.25)	0.49 (0.35 to 0.63)
Venezuela (Bolivarian Republic of)	Diet low in fiber	21 (7 to 42)	0.23 (0.08 to 0.46)	92 (31 to 186)	0.33 (0.11 to 0.66)	0.53 (0.26 to 0.8)

Countries	Risk factors	1990 Number of deaths (95% UI)	1990 ASDR per 100,000 (95% UI)	2019 Number of deaths (95% UI)	2019 ASDR per 100,000 (95% UI)	1990-2019 EAPC of ASDR (95% CI)
Venezuela (Bolivarian Republic of)	Diet low in calcium	148 (112 to 189)	1.63 (1.24 to 2.08)	571 (383 to 806)	2.04 (1.37 to 2.87)	0.07 (-0.19 to 0.33)
Venezuela (Bolivarian Republic of)	Diet high in red meat	23 (2 to 63)	0.25 (0.02 to 0.68)	99 (9 to 277)	0.35 (0.03 to 0.96)	1.02 (0.9 to 1.13)
Venezuela (Bolivarian Republic of)	Diet high in processed meat	11 (2 to 19)	0.12 (0.02 to 0.2)	49 (7 to 98)	0.17 (0.02 to 0.34)	1.26 (1.11 to 1.41)
Venezuela (Bolivarian Republic of)	Diet low in whole grains	111 (42 to 151)	1.21 (0.46 to 1.64)	436 (161 to 636)	1.54 (0.57 to 2.25)	0.49 (0.35 to 0.63)
Viet Nam	All dietary risks	1542 (1185 to 1918)	3.99 (3.07 to 4.94)	6417 (4724 to 8209)	7.22 (5.37 to 9.13)	2.33 (2.21 to 2.45)
Viet Nam	Diet low in milk	431 (163 to 645)	1.11 (0.42 to 1.66)	1897 (711 to 2902)	2.12 (0.79 to 3.24)	2.55 (2.43 to 2.67)
Viet Nam	Diet low in fiber	312 (174 to 447)	0.81 (0.45 to 1.15)	1020 (507 to 1561)	1.16 (0.59 to 1.77)	1.38 (1.25 to 1.5)
Viet Nam	Diet low in calcium	989 (728 to 1279)	2.56 (1.89 to 3.33)	3954 (2901 to 5083)	4.47 (3.29 to 5.71)	2.18 (2.06 to 2.29)
Viet Nam	Diet high in red meat	77 (7 to 209)	0.2 (0.02 to 0.54)	564 (60 to 1418)	0.61 (0.06 to 1.54)	4.5 (4.27 to 4.73)
Viet Nam	Diet high in processed meat	11 (8 to 13)	0.03 (0.02 to 0.03)	65 (45 to 90)	0.07 (0.05 to 0.1)	3.7 (3.51 to 3.89)
Viet Nam	Diet low in whole grains	431 (163 to 645)	1.11 (0.42 to 1.66)	1897 (711 to 2902)	2.12 (0.79 to 3.24)	2.55 (2.43 to 2.67)
Yemen	All dietary risks	109 (69 to 163)	2.41 (1.52 to 3.63)	368 (263 to 512)	2.97 (2.12 to 4.11)	0.99 (0.88 to 1.1)
Yemen	Diet low in milk	52 (26 to 82)	1.16 (0.58 to 1.78)	177 (83 to 257)	1.42 (0.69 to 2.04)	1.05 (0.92 to 1.18)
Yemen	Diet low in fiber	6 (2 to 14)	0.14 (0.04 to 0.32)	25 (9 to 48)	0.2 (0.07 to 0.38)	0.79 (0.61 to 0.97)
Yemen	Diet low in calcium	62 (38 to 95)	1.38 (0.85 to 2.11)	210 (147 to 299)	1.7 (1.19 to 2.39)	0.93 (0.82 to 1.03)
Yemen	Diet high in red meat	4 (0 to 12)	0.09 (0.01 to 0.26)	15 (2 to 42)	0.12 (0.01 to 0.32)	1.46 (1.24 to 1.67)

Countries	Risk factors	1990 Number of deaths (95% UI)	1990 ASDR per 100,000 (95% UI)	2019 Number of deaths (95% UI)	2019 ASDR per 100,000 (95% UI)	1990-2019 EAPC of ASDR (95% CI)
Yemen	Diet high in processed meat	2 (1 to 4)	0.04 (0.01 to 0.08)	6 (2 to 12)	0.05 (0.02 to 0.09)	1.16 (0.99 to 1.32)
Yemen	Diet low in whole grains	52 (26 to 82)	1.16 (0.58 to 1.78)	177 (83 to 257)	1.42 (0.69 to 2.04)	1.05 (0.92 to 1.18)
Zambia	All dietary risks	107 (77 to 144)	4.1 (2.97 to 5.47)	312 (221 to 423)	5.02 (3.57 to 6.69)	0.64 (0.61 to 0.67)
Zambia	Diet low in milk	32 (12 to 51)	1.22 (0.45 to 1.96)	102 (37 to 157)	1.63 (0.61 to 2.53)	0.95 (0.89 to 1.01)
Zambia	Diet low in fiber	1 (1 to 3)	0.05 (0.02 to 0.11)	4 (2 to 9)	0.06 (0.03 to 0.15)	0.97 (0.47 to 1.46)
Zambia	Diet low in calcium	78 (54 to 109)	2.98 (2.07 to 4.1)	219 (150 to 304)	3.54 (2.48 to 4.86)	0.52 (0.49 to 0.55)
Zambia	Diet high in red meat	5 (1 to 15)	0.2 (0.02 to 0.56)	16 (1 to 46)	0.25 (0.02 to 0.7)	0.69 (0.61 to 0.77)
Zambia	Diet high in processed meat	4 (1 to 8)	0.15 (0.02 to 0.3)	12 (2 to 25)	0.18 (0.03 to 0.37)	0.64 (0.54 to 0.73)
Zambia	Diet low in whole grains	32 (12 to 51)	1.22 (0.45 to 1.96)	102 (37 to 157)	1.63 (0.61 to 2.53)	0.95 (0.89 to 1.01)
Zimbabwe	All dietary risks	142 (114 to 172)	3.95 (3.2 to 4.81)	322 (231 to 425)	5.12 (3.72 to 6.7)	0.92 (0.69 to 1.16)
Zimbabwe	Diet low in milk	49 (18 to 70)	1.36 (0.5 to 1.96)	111 (39 to 170)	1.75 (0.62 to 2.69)	0.9 (0.67 to 1.14)
Zimbabwe	Diet low in fiber	3 (1 to 7)	0.09 (0.03 to 0.21)	5 (2 to 13)	0.09 (0.04 to 0.23)	-0.21 (-0.49 to 0.07)
Zimbabwe	Diet low in calcium	98 (76 to 123)	2.73 (2.12 to 3.42)	222 (154 to 296)	3.54 (2.48 to 4.71)	0.93 (0.71 to 1.16)
Zimbabwe	Diet high in red meat	7 (1 to 18)	0.19 (0.02 to 0.49)	17 (2 to 47)	0.26 (0.03 to 0.72)	1.19 (0.9 to 1.48)
Zimbabwe	Diet high in processed meat	4 (1 to 8)	0.11 (0.02 to 0.2)	9 (2 to 18)	0.14 (0.03 to 0.28)	1.14 (0.91 to 1.37)
Zimbabwe	Diet low in whole grains	49 (18 to 70)	1.36 (0.5 to 1.96)	111 (39 to 170)	1.75 (0.62 to 2.69)	0.9 (0.67 to 1.14)

Abbreviations: ASDR, age-standardized death rate; EAPC, estimated annual percentage change; UI, uncertainty interval; CI, confidence

Supplementary Table S5. The death cases and age-standardized death rates of CRC attributable to diet low in whole grains in 1990 and 2019, and their temporal trends from 1990 to 2019.

Characteristics	1990		2019		1990-2019
	Number of deaths (95% UI)	ASDR per 100,000 (95% UI)	Number of deaths (95% UI)	ASDR per 100,000 (95% UI)	EAPC of ASDR (95% CI)
**Global**	83524 (33128 to 108898)	2.31 (0.92 to 3)	171487 (66721 to 225162)	2.16 (0.84 to 2.84)	-0.29 (-0.36 to -0.23)
**Socio-demographic index**					
High SDI	33880 (12820 to 44760)	3.23 (1.22 to 4.26)	51307 (19330 to 67630)	2.55 (0.96 to 3.35)	-0.97 (-1.05 to -0.89)
High-middle SDI	29559 (12447 to 37444)	2.93 (1.23 to 3.7)	55270 (21849 to 72006)	2.75 (1.09 to 3.58)	-0.34 (-0.43 to -0.25)
Middle SDI	12506 (4820 to 16724)	1.32 (0.51 to 1.77)	42038 (16054 to 56386)	1.8 (0.69 to 2.41)	1.24 (1.1 to 1.39)
Low-middle SDI	5392 (2145 to 7140)	0.99 (0.39 to 1.31)	17246 (6672 to 22951)	1.34 (0.52 to 1.79)	1.08 (1.04 to 1.12)
Low SDI	2143 (871 to 2938)	1 (0.41 to 1.36)	5528 (2195 to 7384)	1.17 (0.46 to 1.56)	0.56 (0.51 to 0.6)
**Region**					
Andean Latin America	239 (91 to 314)	1.24 (0.47 to 1.63)	849 (311 to 1207)	1.56 (0.57 to 2.22)	0.99 (0.85 to 1.12)
Australasia	828 (312 to 1110)	3.58 (1.35 to 4.81)	1284 (478 to 1718)	2.49 (0.93 to 3.31)	-1.59 (-1.72 to -1.47)
Caribbean	537 (203 to 696)	2.15 (0.81 to 2.79)	1262 (474 to 1730)	2.44 (0.92 to 3.34)	0.46 (0.4 to 0.52)
Central Asia	1084 (537 to 1330)	2.33 (1.16 to 2.86)	1594 (786 to 1992)	2.4 (1.19 to 3.01)	0.36 (0.19 to 0.53)
Central Europe	5238 (2043 to 6717)	3.66 (1.43 to 4.7)	8363 (3077 to 11205)	3.82 (1.41 to 5.12)	0.14 (0.05 to 0.22)
Central Latin America	776 (292 to 1044)	1 (0.38 to 1.34)	3053 (1126 to 4242)	1.32 (0.49 to 1.83)	0.95 (0.91 to 0.99)
Central Sub-Saharan Africa	218 (83 to 317)	1.09 (0.41 to 1.58)	525 (188 to 772)	1.11 (0.39 to 1.66)	-0.05 (-0.26 to 0.16)
East Asia	12911 (4832 to 17333)	1.6 (0.6 to 2.15)	43889 (16196 to 60896)	2.24 (0.83 to 3.09)	1.47 (1.22 to 1.72)
Eastern Europe	9548 (4160 to 11961)	3.45 (1.5 to 4.31)	10871 (4121 to 14232)	3.13 (1.19 to 4.1)	-0.8 (-1 to -0.6)
Eastern Sub-Saharan Africa	722 (277 to 1001)	1.04 (0.4 to 1.43)	1860 (678 to 2622)	1.24 (0.46 to 1.74)	0.66 (0.6 to 0.73)

High-income Asia Pacific	4617<br>(1724 to 6391)	2.42<br>(0.91 to 3.34)	11244<br>(4217 to 15105)	2.24<br>(0.84 to 2.99)	-0.33<br>(-0.42 to -0.24)
High-income North America	10993<br>(4134 to 14609)	3.06<br>(1.15 to 4.06)	14816<br>(5557 to 19542)	2.31<br>(0.87 to 3.05)	-1.17<br>(-1.27 to -1.08)
North Africa and Middle East	2617<br>(1225 to 3420)	1.66<br>(0.79 to 2.16)	7822<br>(3657 to 10100)	1.98<br>(0.92 to 2.54)	0.8<br>(0.6 to 1)
Oceania	34<br>(13 to 47)	1.3<br>(0.5 to 1.77)	92<br>(35 to 129)	1.48<br>(0.55 to 2.04)	0.41<br>(0.33 to 0.49)
South Asia	4011<br>(1589 to 5431)	0.8<br>(0.32 to 1.08)	13845<br>(5413 to 18842)	1.07<br>(0.42 to 1.45)	0.84<br>(0.72 to 0.96)
Southeast Asia	3056<br>(1160 to 4288)	1.28<br>(0.49 to 1.78)	10359<br>(3942 to 15324)	1.82<br>(0.69 to 2.69)	1.13<br>(1.07 to 1.2)
Southern Latin America	1689<br>(711 to 2141)	3.82<br>(1.6 to 4.85)	3089<br>(1147 to 3994)	3.65<br>(1.36 to 4.72)	-0.18<br>(-0.26 to -0.1)
Southern Sub-Saharan Africa	317<br>(120 to 470)	1.25<br>(0.47 to 1.87)	752<br>(276 to 1076)	1.45<br>(0.54 to 2.09)	0.53<br>(0.32 to 0.75)
Tropical Latin America	1277<br>(476 to 1683)	1.53<br>(0.57 to 2.01)	3969<br>(1479 to 5328)	1.67<br>(0.62 to 2.25)	0.37<br>(0.23 to 0.52)
Western Europe	21986<br>(8795 to 28247)	3.74<br>(1.49 to 4.8)	29831<br>(12051 to 38310)	2.99<br>(1.2 to 3.83)	-0.97<br>(-1.13 to -0.82)
Western Sub-Saharan Africa	827<br>(322 to 1164)	1.06<br>(0.41 to 1.49)	2119<br>(801 to 2911)	1.3<br>(0.49 to 1.78)	0.91<br>(0.82 to 1)

Abbreviations: SDI, sociodemographic index; ASDR, age-standardized death rate; EAPC, estimated annual percentage change; UI, uncertainty interval; CI, confidence interval.

Supplementary Table S6. The death cases and age-standardized death rates of CRC attributable to diet low in milk in 1990 and 2019, and their temporal trends from 1990 to 2019.

Characteristics	1990		2019		1990-2019
	Number of deaths (95% UI)	ASDR per 100,000 (95% UI)	Number of deaths (95% UI)	ASDR per 100,000 (95% UI)	EAPC of ASDR (95% CI)
Global	72199 (44384 to 99966)	1.98 (1.2 to 2.74)	166456 (107221 to 226027)	2.09 (1.34 to 2.84)	0.19 (0.16 to 0.23)
Socio-demographic index					
High SDI	26999 (14174 to 39995)	2.58 (1.36 to 3.81)	39255 (21762 to 57568)	1.95 (1.08 to 2.85)	-1.14 (-1.2 to -1.09)
High-middle SDI	19832 (11648 to 28461)	1.98 (1.16 to 2.84)	46115 (28371 to 63889)	2.29 (1.41 to 3.17)	0.55 (0.49 to 0.61)
Middle SDI	15910 (11069 to 20548)	1.68 (1.17 to 2.18)	52394 (34928 to 69155)	2.24 (1.49 to 2.96)	1.13 (1 to 1.25)
Low-middle SDI	6808 (4700 to 9069)	1.23 (0.85 to 1.65)	22080 (14880 to 29018)	1.72 (1.16 to 2.25)	1.13 (1.08 to 1.18)
Low SDI	2607 (1744 to 3557)	1.21 (0.81 to 1.65)	6514 (4308 to 8719)	1.39 (0.92 to 1.86)	0.46 (0.38 to 0.54)
Region					
Andean Latin America	269 (177 to 367)	1.4 (0.92 to 1.91)	998 (629 to 1408)	1.83 (1.16 to 2.58)	1.13 (0.99 to 1.28)
Australasia	467 (167 to 780)	2.02 (0.74 to 3.38)	410 (142 to 806)	0.78 (0.26 to 1.53)	-4.1 (-4.54 to -3.65)
Caribbean	580 (376 to 785)	2.32 (1.51 to 3.15)	1431 (910 to 1993)	2.77 (1.76 to 3.85)	0.63 (0.58 to 0.68)
Central Asia	481 (232 to 755)	1.03 (0.5 to 1.62)	691 (340 to 1098)	1.04 (0.51 to 1.63)	-0.09 (-0.28 to 0.1)
Central Europe	3711 (1771 to 5620)	2.61 (1.25 to 3.94)	6315 (3246 to 9515)	2.88 (1.49 to 4.34)	0.3 (0.18 to 0.43)
Central Latin America	924 (571 to 1281)	1.19 (0.73 to 1.64)	3463 (1972 to 5057)	1.5 (0.85 to 2.19)	0.78 (0.73 to 0.82)
Central Sub-Saharan Africa	412 (299 to 546)	2.07 (1.5 to 2.75)	929 (632 to 1316)	1.98 (1.33 to 2.84)	-0.27 (-0.52 to -0.02)
East Asia	15903 (10806 to 20942)	1.98 (1.35 to 2.61)	52877 (35241 to 71142)	2.7 (1.8 to 3.61)	1.39 (1.15 to 1.62)

Eastern Europe	3464 (1195 to 6059)	1.28 (0.45 to 2.21)	5912 (2386 to 9539)	1.71 (0.7 to 2.76)	0.92 (0.57 to 1.27)
Eastern Sub-Saharan Africa	894 (571 to 1253)	1.29 (0.83 to 1.79)	2265 (1440 to 3135)	1.52 (0.97 to 2.09)	0.58 (0.51 to 0.66)
High-income Asia Pacific	5862 (3680 to 8025)	3.06 (1.92 to 4.17)	13124 (8062 to 18133)	2.61 (1.58 to 3.59)	-0.65 (-0.71 to -0.59)
High-income North America	7358 (3207 to 11460)	2.05 (0.9 to 3.2)	8476 (3297 to 13802)	1.32 (0.51 to 2.16)	-1.72 (-1.91 to -1.53)
North Africa and Middle East	2006 (1240 to 2904)	1.27 (0.78 to 1.84)	6185 (3790 to 8725)	1.55 (0.94 to 2.19)	0.81 (0.67 to 0.94)
Oceania	39 (25 to 54)	1.47 (0.95 to 2.05)	104 (66 to 145)	1.68 (1.09 to 2.3)	0.43 (0.36 to 0.5)
South Asia	5419 (3766 to 7190)	1.07 (0.74 to 1.42)	19002 (12875 to 24981)	1.47 (1 to 1.93)	0.94 (0.81 to 1.06)
Southeast Asia	5071 (3679 to 6466)	2.12 (1.54 to 2.7)	16263 (11214 to 21732)	2.86 (1.97 to 3.82)	0.91 (0.85 to 0.96)
Southern Latin America	1317 (748 to 1881)	2.98 (1.68 to 4.26)	2705 (1542 to 3833)	3.2 (1.83 to 4.54)	0.26 (0.14 to 0.37)
Southern Sub-Saharan Africa	452 (283 to 641)	1.8 (1.12 to 2.57)	1017 (637 to 1423)	1.98 (1.23 to 2.76)	0.32 (0.11 to 0.53)
Tropical Latin America	1279 (705 to 1845)	1.53 (0.84 to 2.22)	3659 (1822 to 5417)	1.54 (0.77 to 2.28)	-0.01 (-0.16 to 0.14)
Western Europe	15268 (7361 to 23213)	2.6 (1.25 to 3.95)	17956 (8091 to 28063)	1.8 (0.81 to 2.84)	-1.57 (-1.71 to -1.44)
Western Sub-Saharan Africa	1025 (681 to 1431)	1.31 (0.88 to 1.83)	2677 (1794 to 3619)	1.65 (1.1 to 2.21)	1.02 (0.91 to 1.13)

Abbreviations: SDI, sociodemographic index; ASDR, age-standardized death rate; EAPC, estimated annual percentage change; UI, uncertainty interval; CI, confidence interval.

Supplementary Table S7. The death cases and age-standardized death rates of CRC attributable to diet low in fiber in 1990 and 2019, and their temporal trends from 1990 to 2019.

Characteristics	1990		2019		1990-2019
	**Number of deaths (95% UI)**	**ASDR per 100,000 (95% UI)**	**Number of deaths (95% UI)**	**ASDR per 100,000 (95% UI)**	**EAPC of ASDR (95% CI)**
**Global**	12548 (4964 to 23334)	0.36 (0.14 to 0.66)	20499 (8205 to 39829)	0.26 (0.11 to 0.51)	-1.13 (-1.23 to -1.02)
**Socio-demographic index**					
High SDI	5663 (2154 to 10850)	0.54 (0.21 to 1.03)	7284 (2772 to 14364)	0.35 (0.13 to 0.7)	-1.5 (-1.54 to -1.47)
High-middle SDI	2725 (988 to 5696)	0.28 (0.1 to 0.57)	4442 (1740 to 9415)	0.22 (0.09 to 0.47)	-0.92 (-1.16 to -0.69)
Middle SDI	2668 (1153 to 4500)	0.28 (0.12 to 0.47)	5433 (2336 to 9959)	0.23 (0.1 to 0.43)	-0.61 (-0.75 to -0.46)
Low-middle SDI	1250 (567 to 2076)	0.23 (0.11 to 0.38)	2771 (1210 to 5054)	0.22 (0.1 to 0.4)	-0.28 (-0.42 to -0.15)
Low SDI	234 (92 to 476)	0.11 (0.05 to 0.23)	556 (219 to 1071)	0.12 (0.05 to 0.23)	0.12 (-0.03 to 0.27)
**Region**					
Andean Latin America	47 (17 to 92)	0.25 (0.09 to 0.48)	128 (49 to 242)	0.24 (0.09 to 0.45)	-0.01 (-0.13 to 0.11)
Australasia	157 (55 to 307)	0.68 (0.24 to 1.32)	187 (69 to 365)	0.36 (0.13 to 0.71)	-2.59 (-2.76 to -2.41)
Caribbean	86 (31 to 170)	0.34 (0.12 to 0.68)	110 (43 to 223)	0.21 (0.08 to 0.43)	-1.81 (-2.09 to -1.53)
Central Asia	119 (42 to 244)	0.26 (0.09 to 0.53)	115 (41 to 256)	0.18 (0.06 to 0.4)	-1.81 (-2.22 to -1.39)
Central Europe	495 (178 to 1102)	0.35 (0.13 to 0.78)	764 (280 to 1622)	0.35 (0.13 to 0.74)	-0.1 (-0.39 to 0.2)
Central Latin America	95 (38 to 180)	0.12 (0.05 to 0.23)	319 (122 to 654)	0.14 (0.05 to 0.29)	0.47 (0.38 to 0.57)

Central Sub-Saharan Africa	17 (6 to 39)	0.09 (0.03 to 0.21)	56 (19 to 121)	0.12 (0.04 to 0.27)	0.98 (0.78 to 1.18)
East Asia	2025 (798 to 3825)	0.25 (0.1 to 0.47)	2898 (1197 to 6261)	0.15 (0.06 to 0.33)	-1.32 (-1.63 to -1.01)
Eastern Europe	623 (235 to 1419)	0.23 (0.09 to 0.52)	892 (334 to 1988)	0.26 (0.1 to 0.57)	-0.7 (-1.31 to -0.08)
Eastern Sub-Saharan Africa	42 (18 to 93)	0.06 (0.03 to 0.14)	84 (41 to 164)	0.06 (0.03 to 0.11)	-0.58 (-0.65 to -0.52)
High-income Asia Pacific	638 (241 to 1310)	0.34 (0.13 to 0.69)	1912 (771 to 3631)	0.38 (0.15 to 0.71)	0.5 (0.34 to 0.65)
High-income North America	2161 (833 to 3970)	0.6 (0.23 to 1.09)	1910 (732 to 3778)	0.29 (0.11 to 0.58)	-2.51 (-2.67 to -2.34)
North Africa and Middle East	110 (48 to 242)	0.07 (0.03 to 0.16)	309 (139 to 614)	0.08 (0.04 to 0.16)	0.24 (0.17 to 0.31)
Oceania	1 (1 to 2)	0.04 (0.02 to 0.08)	2 (1 to 3)	0.03 (0.02 to 0.05)	-0.89 (-1.15 to -0.62)
South Asia	902 (379 to 1593)	0.19 (0.08 to 0.33)	2157 (872 to 4124)	0.17 (0.07 to 0.33)	-0.49 (-0.71 to -0.27)
Southeast Asia	1557 (791 to 2286)	0.66 (0.34 to 0.96)	3920 (1783 to 6398)	0.7 (0.32 to 1.14)	0.09 (0.02 to 0.16)
Southern Latin America	288 (101 to 537)	0.66 (0.24 to 1.22)	494 (174 to 955)	0.58 (0.21 to 1.13)	-0.17 (-0.3 to -0.03)
Southern Sub-Saharan Africa	21 (9 to 47)	0.09 (0.03 to 0.19)	53 (22 to 118)	0.11 (0.04 to 0.23)	0.63 (0.42 to 0.84)
Tropical Latin America	223 (87 to 422)	0.27 (0.11 to 0.51)	500 (198 to 1020)	0.21 (0.08 to 0.43)	-1.22 (-1.36 to -1.09)
Western Europe	2892 (1074 to 5896)	0.49 (0.18 to 0.99)	3624 (1343 to 7418)	0.35 (0.13 to 0.73)	-1.28 (-1.4 to -1.17)
Western Sub-Saharan Africa	49 (22 to 106)	0.07 (0.03 to 0.14)	64 (38 to 114)	0.04 (0.02 to 0.07)	-1.75 (-2.03 to -1.47)

Abbreviations: SDI, sociodemographic index; ASDR, age-standardized death rate; EAPC, estimated annual percentage change; UI, uncertainty interval; CI, confidence interval.

Supplementary Table S8. The death cases and age-standardized death rates of CRC attributable to diet low in calcium in 1990 and 2019, and their temporal trends from 1990 to 2019.

Characteristics	1990		2019		1990-2019
	Number of deaths (95% UI)	ASDR per 100,000 (95% UI)	Number of deaths (95% UI)	ASDR per 100,000 (95% UI)	EAPC of ASDR (95% CI)
**Global**	63709 (46057 to 87779)	1.73 (1.24 to 2.41)	137896 (96835 to 189221)	1.73 (1.21 to 2.38)	0.04 (-0.06 to 0.13)
**Socio-demographic index**					
High SDI	16353 (9362 to 26404)	1.56 (0.89 to 2.51)	24723 (14298 to 38861)	1.2 (0.69 to 1.88)	-0.85 (-0.89 to -0.82)
High-middle SDI	18120 (11963 to 26825)	1.8 (1.19 to 2.66)	33330 (20306 to 50206)	1.66 (1.01 to 2.5)	-0.37 (-0.54 to -0.21)
Middle SDI	18240 (14735 to 22771)	1.95 (1.57 to 2.42)	50250 (37787 to 65537)	2.16 (1.62 to 2.81)	0.51 (0.35 to 0.66)
Low-middle SDI	7730 (6177 to 9752)	1.44 (1.15 to 1.79)	21803 (16914 to 27979)	1.73 (1.35 to 2.21)	0.65 (0.58 to 0.71)
Low SDI	3228 (2501 to 4133)	1.52 (1.18 to 1.94)	7704 (6158 to 9579)	1.66 (1.34 to 2.06)	0.3 (0.25 to 0.34)
**Region**					
Andean Latin America	315 (242 to 400)	1.65 (1.27 to 2.1)	975 (684 to 1335)	1.79 (1.26 to 2.45)	0.37 (0.23 to 0.52)
Australasia	392 (199 to 685)	1.7 (0.86 to 2.96)	634 (344 to 1054)	1.21 (0.66 to 2.01)	-1.47 (-1.62 to -1.32)
Caribbean	515 (373 to 695)	2.07 (1.5 to 2.78)	1119 (761 to 1576)	2.17 (1.48 to 3.05)	-0.03 (-0.11 to 0.06)
Central Asia	527 (326 to 811)	1.14 (0.7 to 1.75)	592 (344 to 936)	0.91 (0.53 to 1.42)	-1.02 (-1.23 to -0.82)
Central Europe	2436 (1321 to 4075)	1.72 (0.93 to 2.87)	3566 (1853 to 6002)	1.61 (0.84 to 2.72)	-0.32 (-0.57 to -0.07)
Central Latin America	1007 (756 to 1303)	1.31 (0.98 to 1.69)	3369 (2286 to 4734)	1.47 (0.99 to 2.06)	0.33 (0.27 to 0.39)
Central Sub-Saharan Africa	386 (286 to 515)	1.96 (1.45 to 2.61)	907 (647 to 1244)	1.94 (1.37 to 2.72)	-0.16 (-0.37 to 0.06)
East Asia	17222 (13608 to 22106)	2.16 (1.71 to 2.77)	42151 (28995 to 58467)	2.16 (1.49 to 2.97)	0.38 (0.08 to 0.68)
Eastern Europe	4279 (2343 to 7049)	1.56 (0.86 to 2.55)	4611 (2416 to 7893)	1.32 (0.69 to 2.27)	-1.51 (-1.94 to -1.07)
Eastern Sub-Saharan Africa	1208 (930 to 1565)	1.76 (1.35 to 2.25)	2878 (2244 to 3643)	1.95 (1.54 to 2.45)	0.35 (0.29 to 0.4)
High-income Asia Pacific	4703 (3110 to 6629)	2.47 (1.63 to 3.47)	10449 (6635 to 15092)	2.03 (1.27 to 2.95)	-0.56 (-0.65 to -0.47)
High-income North America	4462 (2309 to 7663)	1.23 (0.63 to 2.1)	5202 (2578 to 9028)	0.79 (0.39 to 1.37)	-1.27 (-1.55 to -0.99)

North Africa and Middle East	2010 (1445 to 2757)	1.28 (0.92 to 1.76)	5231 (3621 to 7273)	1.32 (0.91 to 1.84)	0.18 (0.06 to 0.3)
Oceania	47 (34 to 62)	1.81 (1.33 to 2.37)	127 (94 to 170)	2.05 (1.56 to 2.7)	0.42 (0.38 to 0.47)
South Asia	5469 (4328 to 6909)	1.13 (0.89 to 1.43)	16734 (12701 to 21942)	1.34 (1.02 to 1.74)	0.5 (0.39 to 0.62)
Southeast Asia	6431 (5000 to 7928)	2.7 (2.09 to 3.31)	20593 (15534 to 26291)	3.63 (2.74 to 4.61)	0.94 (0.88 to 1.01)
Southern Latin America	1096 (705 to 1593)	2.5 (1.61 to 3.62)	1963 (1194 to 3008)	2.31 (1.41 to 3.55)	-0.08 (-0.18 to 0.03)
Southern Sub-Saharan Africa	615 (475 to 778)	2.45 (1.88 to 3.13)	1390 (1118 to 1705)	2.72 (2.18 to 3.34)	0.31 (0.09 to 0.54)
Tropical Latin America	1332 (962 to 1785)	1.62 (1.17 to 2.17)	2893 (1714 to 4445)	1.23 (0.73 to 1.89)	-1.22 (-1.34 to -1.09)
Western Europe	7941 (3973 to 13940)	1.33 (0.66 to 2.33)	9416 (4700 to 16258)	0.91 (0.45 to 1.58)	-1.45 (-1.55 to -1.35)
Western Sub-Saharan Africa	1315 (1005 to 1725)	1.7 (1.3 to 2.22)	3098 (2366 to 3964)	1.93 (1.48 to 2.45)	0.57 (0.49 to 0.65)

Abbreviations: SDI, sociodemographic index; ASDR, age-standardized death rate; EAPC, estimated annual percentage change; UI, uncertainty interval; CI, confidence interval.

Supplementary Table S9. The death cases and age-standardized death rates of CRC attributable to diet high in red meat in 1990 and 2019, and their temporal trends from 1990 to 2019.

Characteristics	1990		2019		1990-2019
	Number of deaths (95% UI)	ASDR per 100,000 (95% UI)	Number of deaths (95% UI)	ASDR per 100,000 (95% UI)	EAPC of ASDR (95% CI)
**Global**	26087 (6690 to 50231)	0.71 (0.18 to 1.38)	52811 (13598 to 100688)	0.66 (0.17 to 1.26)	-0.32 (-0.37 to -0.28)
**Socio-demographic index**					
High SDI	14063 (3944 to 25419)	1.34 (0.38 to 2.42)	18849 (4893 to 35349)	0.97 (0.26 to 1.79)	-1.29 (-1.37 to -1.21)
High-middle SDI	8864 (2276 to 17225)	0.87 (0.22 to 1.7)	18793 (5033 to 35132)	0.93 (0.25 to 1.74)	0.09 (0.01 to 0.17)
Middle SDI	2278 (297 to 5662)	0.23 (0.03 to 0.59)	11723 (2560 to 23587)	0.49 (0.1 to 0.99)	2.95 (2.77 to 3.12)
Low-middle SDI	649 (122 to 1497)	0.12 (0.02 to 0.27)	2826 (617 to 5904)	0.22 (0.05 to 0.45)	2.38 (2.28 to 2.48)
Low SDI	221 (32 to 565)	0.1 (0.01 to 0.26)	598 (98 to 1464)	0.12 (0.02 to 0.3)	0.81 (0.72 to 0.9)
**Region**					
Andean Latin America	36 (4 to 92)	0.18 (0.02 to 0.47)	154 (17 to 392)	0.28 (0.03 to 0.71)	1.81 (1.68 to 1.95)
Australasia	630 (276 to 959)	2.72 (1.19 to 4.15)	888 (367 to 1387)	1.75 (0.74 to 2.71)	-1.8 (-1.97 to -1.63)
Caribbean	83 (8 to 220)	0.33 (0.03 to 0.87)	209 (20 to 560)	0.4 (0.04 to 1.08)	0.9 (0.79 to 1.01)
Central Asia	271 (70 to 524)	0.57 (0.14 to 1.11)	389 (98 to 756)	0.56 (0.14 to 1.11)	0.39 (-0.13 to 0.91)
Central Europe	1538 (322 to 3190)	1.06 (0.22 to 2.23)	2862 (699 to 5678)	1.33 (0.33 to 2.61)	1.04 (0.92 to 1.17)
Central Latin America	189 (24 to 461)	0.24 (0.03 to 0.58)	807 (130 to 1946)	0.34 (0.05 to 0.84)	1.38 (1.33 to 1.42)
Central Sub-Saharan Africa	27 (5 to 67)	0.13 (0.02 to 0.32)	61 (10 to 150)	0.12 (0.02 to 0.3)	-0.18 (-0.51 to 0.15)
East Asia	2851 (408 to 6970)	0.34 (0.05 to 0.84)	16498 (4056 to 31569)	0.82 (0.2 to 1.58)	3.6 (3.35 to 3.86)
Eastern Europe	2995 (772 to 5768)	1.07 (0.28 to 2.07)	2708 (468 to 5947)	0.79 (0.14 to 1.72)	-1.91 (-2.21 to -1.62)
Eastern Sub-Saharan Africa	88 (10 to 231)	0.12 (0.01 to 0.32)	241 (27 to 626)	0.16 (0.02 to 0.4)	0.9 (0.79 to 1.02)
High-income Asia Pacific	910 (87 to 2421)	0.47 (0.05 to 1.25)	2196 (275 to 5546)	0.47 (0.06 to 1.14)	-0.25 (-0.35 to -0.15)
High-income North America	4798 (1332 to 8719)	1.36 (0.38 to 2.44)	6386 (1712 to 11567)	1.02 (0.28 to 1.82)	-1.21 (-1.33 to -1.09)

North Africa and Middle East	301<br>(29 to 820)	0.18<br>(0.02 to 0.5)	882<br>(86 to 2352)	0.22<br>(0.02 to 0.57)	0.76<br>(0.51 to 1.02)
Oceania	6<br>(1 to 16)	0.22<br>(0.03 to 0.59)	15<br>(2 to 41)	0.24<br>(0.03 to 0.64)	0.11<br>(-0.02 to 0.24)
South Asia	267<br>(70 to 583)	0.05<br>(0.01 to 0.12)	947<br>(228 to 2161)	0.07<br>(0.02 to 0.16)	0.84<br>(0.71 to 0.97)
Southeast Asia	406<br>(51 to 1036)	0.17<br>(0.02 to 0.42)	1822<br>(220 to 4550)	0.31<br>(0.04 to 0.77)	2.15<br>(2.08 to 2.21)
Southern Latin America	896<br>(376 to 1387)	2<br>(0.82 to 3.09)	1768<br>(705 to 2777)	2.1<br>(0.84 to 3.3)	0.15<br>(0.06 to 0.23)
Southern Sub-Saharan Africa	83<br>(11 to 211)	0.32<br>(0.04 to 0.84)	209<br>(30 to 498)	0.4<br>(0.06 to 0.95)	1.01<br>(0.84 to 1.18)
Tropical Latin America	388<br>(78 to 834)	0.45<br>(0.09 to 0.98)	2403<br>(890 to 3925)	1<br>(0.36 to 1.64)	3.02<br>(2.41 to 3.64)
Western Europe	9232<br>(2731 to 16476)	1.59<br>(0.48 to 2.82)	11104<br>(2953 to 20565)	1.16<br>(0.32 to 2.11)	-1.33<br>(-1.44 to -1.22)
Western Sub-Saharan Africa	92<br>(10 to 244)	0.11<br>(0.01 to 0.3)	262<br>(29 to 684)	0.16<br>(0.02 to 0.41)	1.35<br>(1.23 to 1.48)

Abbreviations: SDI, sociodemographic index; ASDR, age-standardized death rate; EAPC, estimated annual percentage change; UI, uncertainty interval; CI, confidence interval.

Supplementary Table S10. The death cases and age-standardized death rates of CRC attributable to diet high in processed meat in 1990 and 2019, and their temporal trends from 1990 to 2019.

Characteristics	1990		2019		1990-2019
	Number of deaths (95% UI)	ASDR per 100,000 (95% UI)	Number of deaths (95% UI)	ASDR per 100,000 (95% UI)	EAPC of ASDR (95% CI)
Global	20185 (7515 to 30917)	0.56 (0.2 to 0.87)	33928 (11645 to 52096)	0.43 (0.15 to 0.66)	-1.08 (-1.2 to -0.96)
Socio-demographic index					
High-middle SDI	6473 (2283 to 10459)	0.63 (0.22 to 1.02)	10456 (2857 to 16319)	0.52 (0.14 to 0.81)	-0.88 (-1.01 to -0.74)
High SDI	12344 (4087 to 18743)	1.18 (0.39 to 1.79)	17957 (6063 to 27711)	0.91 (0.32 to 1.41)	-1.04 (-1.19 to -0.89)
Low-middle SDI	426 (172 to 661)	0.08 (0.03 to 0.12)	1620 (654 to 2475)	0.12 (0.05 to 0.19)	1.79 (1.71 to 1.86)
Low SDI	208 (47 to 364)	0.09 (0.02 to 0.16)	587 (139 to 987)	0.12 (0.03 to 0.2)	0.89 (0.84 to 0.94)
Middle SDI	725 (307 to 1102)	0.08 (0.03 to 0.11)	3293 (1028 to 5402)	0.14 (0.04 to 0.22)	2.47 (2.32 to 2.62)
Region					
Andean Latin America	10 (5 to 15)	0.05 (0.03 to 0.07)	44 (17 to 75)	0.08 (0.03 to 0.13)	2.03 (1.88 to 2.18)
Australasia	279 (69 to 435)	1.21 (0.31 to 1.89)	450 (120 to 693)	0.89 (0.24 to 1.36)	-1.27 (-1.4 to -1.14)
Caribbean	39 (7 to 67)	0.15 (0.03 to 0.26)	100 (18 to 181)	0.19 (0.04 to 0.35)	1.02 (0.95 to 1.09)
Central Asia	190 (33 to 306)	0.4 (0.07 to 0.65)	280 (50 to 457)	0.4 (0.07 to 0.66)	0.31 (0.03 to 0.6)
Central Europe	1002 (178 to 1615)	0.7 (0.12 to 1.13)	2007 (436 to 3289)	0.93 (0.21 to 1.52)	1.35 (1.18 to 1.51)
Central Latin America	78 (15 to 131)	0.1 (0.02 to 0.16)	342 (65 to 603)	0.15 (0.03 to 0.26)	1.55 (1.49 to 1.6)
Central Sub-Saharan Africa	24 (3 to 47)	0.11 (0.02 to 0.22)	54 (8 to 105)	0.11 (0.02 to 0.21)	-0.22 (-0.54 to 0.11)
East Asia	622 (277 to 987)	0.08 (0.03 to 0.12)	3378 (701 to 6071)	0.17 (0.04 to 0.3)	3.41 (3.13 to 3.69)

Eastern Europe	3313 (1175 to 5893)	1.19 (0.42 to 2.11)	3347 (1058 to 5208)	0.96 (0.3 to 1.5)	-1.2 (-1.49 to -0.91)
Eastern Sub-Saharan Africa	73 (11 to 134)	0.1 (0.02 to 0.18)	200 (30 to 370)	0.13 (0.02 to 0.23)	0.95 (0.85 to 1.06)
High-income Asia Pacific	1480 (384 to 2310)	0.74 (0.19 to 1.16)	3003 (761 to 4758)	0.66 (0.18 to 1.02)	-0.5 (-0.75 to -0.24)
High-income North America	4261 (1414 to 6494)	1.19 (0.4 to 1.82)	6316 (2234 to 10039)	1 (0.36 to 1.6)	-0.82 (-0.91 to -0.73)
North Africa and Middle East	116 (32 to 208)	0.07 (0.02 to 0.13)	411 (95 to 725)	0.1 (0.02 to 0.17)	1.45 (1.21 to 1.7)
Oceania	2 (1 to 3)	0.06 (0.02 to 0.1)	5 (1 to 8)	0.07 (0.02 to 0.13)	0.58 (0.49 to 0.66)
South Asia	339 (177 to 507)	0.07 (0.03 to 0.1)	1421 (852 to 2054)	0.11 (0.06 to 0.15)	1.6 (1.52 to 1.69)
Southeast Asia	137 (73 to 195)	0.06 (0.03 to 0.08)	592 (264 to 926)	0.1 (0.05 to 0.16)	2.06 (1.95 to 2.18)
Southern Latin America	280 (47 to 456)	0.63 (0.1 to 1.02)	725 (163 to 1152)	0.86 (0.19 to 1.37)	1.16 (1.09 to 1.23)
Southern Sub-Saharan Africa	32 (6 to 60)	0.12 (0.02 to 0.23)	85 (14 to 152)	0.16 (0.03 to 0.28)	1.08 (0.91 to 1.26)
Tropical Latin America	111 (20 to 194)	0.13 (0.02 to 0.22)	501 (68 to 872)	0.21 (0.03 to 0.36)	2.04 (1.92 to 2.16)
Western Europe	7676 (2454 to 11669)	1.31 (0.42 to 2)	10299 (3467 to 15699)	1.05 (0.36 to 1.61)	-0.94 (-1.14 to -0.75)
Western Sub-Saharan Africa	120 (16 to 222)	0.15 (0.02 to 0.27)	368 (55 to 644)	0.21 (0.03 to 0.37)	1.57 (1.46 to 1.68)

Abbreviations: SDI, sociodemographic index; ASDR, age-standardized death rate; EAPC, estimated annual percentage change; UI, uncertainty interval; CI, confidence interval.
